# Synergistic Brønsted/Lewis acid catalyzed aromatic alkylation with unactivated tertiary alcohols or di-*tert*-butylperoxide to synthesize quaternary carbon centers†

**DOI:** 10.1039/d1sc06422c

**Published:** 2022-03-08

**Authors:** Aaron Pan, Maja Chojnacka, Robert Crowley, Lucas Göttemann, Brandon E. Haines, Kevin G. M. Kou

**Affiliations:** Department of Chemistry, University of California, Riverside 501 Big Springs Road Riverside CA 92521 USA kevin.kou@ucr.edu; Department of Chemistry, Westmont College 955 La Paz Road Santa Barbara CA 93108 USA bhaines@westmont.edu

## Abstract

Dual Brønsted/Lewis acid catalysis involving environmentally benign, readily accessible protic acid and iron promotes site-selective *tert*-butylation of electron-rich arenes using di-*tert*-butylperoxide. This transformation inspired the development of a synergistic Brønsted/Lewis acid catalyzed aromatic alkylation that fills a gap in the Friedel–Crafts reaction literature by employing unactivated tertiary alcohols as alkylating agents, leading to new quaternary carbon centers. Corroborated by DFT calculations, the Lewis acid serves a role in enhancing the acidity of the Brønsted acid. The use of non-allylic, non-benzylic, and non-propargylic tertiary alcohols represents an underexplored area in Friedel–Crafts reactivity.

## Abstract 2

Dual Brønsted/Lewis acid catalysis involving environmentally benign, readily accessible protic acid and iron promotes site-selective *tert*-alkylation of arenes using di-*tert*-butylperoxide and tertiary alcohols.

## Graphical abstract

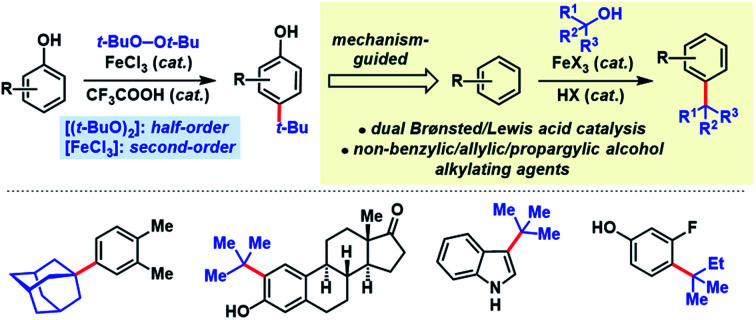

## Introduction

**Fig. 1:**
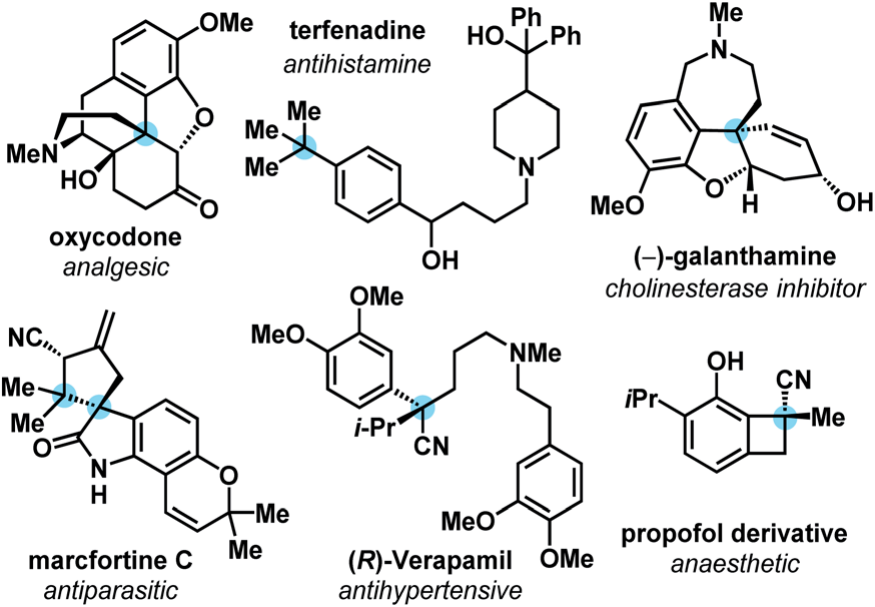
Bioactive molecules bearing all-carbon quaternary carbon centers.

The simplicity and efficiency of sp^2^–sp^2^ cross-coupling technologies have driven its widespread adoption by the synthetic community, influencing synthesis strategies and the types of molecules that are most readily synthesized by the pharmaceutical industry. However, as a community, we are realizing trends that indicate enhanced developability and clinical success of organic molecules that exhibit greater degrees of saturation, which is often correlated with increasing numbers of sp^3^-hybridized carbons.^1^ This ‘molecular complexity’ tends to improve a compound's aqueous solubility, crystallinity, and binding specificity.^2^ All-carbon quaternary centers are frequently encountered in bioactive natural products, pharmaceuticals, and drug candidates (Fig. 1).^3^

In a recent analysis of modern Negishi, Suzuki, and various nickel-catalyzed photoredox cross-coupling methods for constructing C(sp^2^)–C(sp^3^) aryl–alkyl bonds by Abbvie scientists, none were able to install a *tert*-butyl group.^2*b*^ This highlights the challenges inherent in synthesizing quaternary carbon centers, as well as the limitations that exist even with state-of-the-art catalysis. As such, considerable efforts have been devoted to their catalytic synthesis with precious metals, where palladium, rhodium, and iridium demonstrate the greatest utility.^4^

**Scheme 1:**
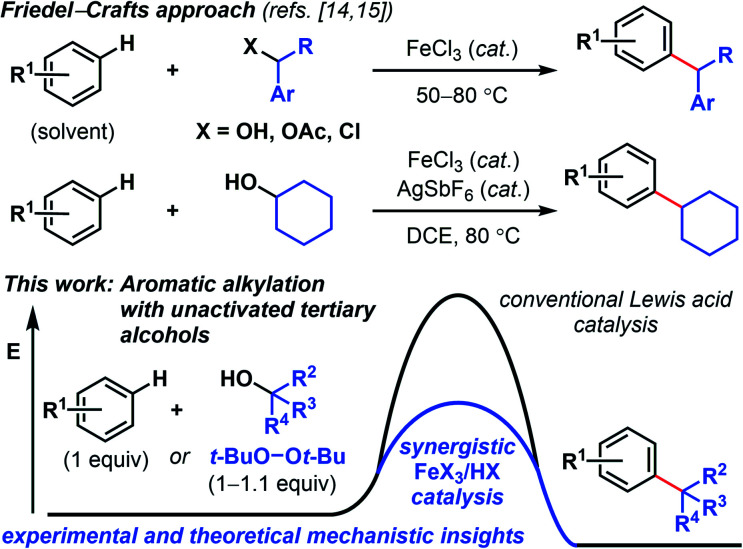
Synergistic iron/TFA-catalyzed *tert*-butylation of phenol using peroxide reagents with and without an acid co-catalyst.

Modern variations of Kumada^5^ and Suzuki reactions,^6^ including photoredox-mediated,^7^ reductive,^8^ and redox-active ester-mediated cross-couplings^9^ have demonstrated success in merging C(sp^2^) and C(sp^3^) units to forge new all-carbon quaternary centers. While the development of nickel^5–11^ and copper^12^ catalyses for synthesizing quaternary carbon centers have progressed in recent years, examples with other abundant transition metals such as iron, are scarce.^13^ In considering new solutions to quaternary carbon synthesis, we surmised that a Friedel–Crafts approach would effectively permit direct C–H functionalization. In this respect, Beller and coworkers reported primary and secondary benzylic halides/acetates/alcohols coupling with arenes under iron catalysis (Scheme 1).^14^ The Cook group found that in conjunction with a silver salt, FeCl_3_ promotes Friedel–Crafts reactions between arenes and unactivated secondary alcohols.^15^ The use of triflic acid in hexafluoroisopropanol solvent can also promote arene alkylation with unactivated alcohols.^16^ Herein, we disclose a Fenton-inspired, synergistic Brønsted/Lewis acid-catalysis^17^ that enables aromatic alkylation with unactivated tertiary alcohols. Successful *tert*-butylation has been reported with superstoichiometric amounts of strong acid^18^ or superacidic heterogeneous catalysts.^19^ Our findings provide a general, complementary approach and represent underexplored examples of using non-benzylic, non-propargylic, and non-allylic alcohols for Friedel–Crafts-type alkylations.^14,20,21^ The use of environmentally benign, readily accessible reagents and catalysts provides a green approach to quaternary carbon synthesis.

## Results and discussion

**Scheme 2:**
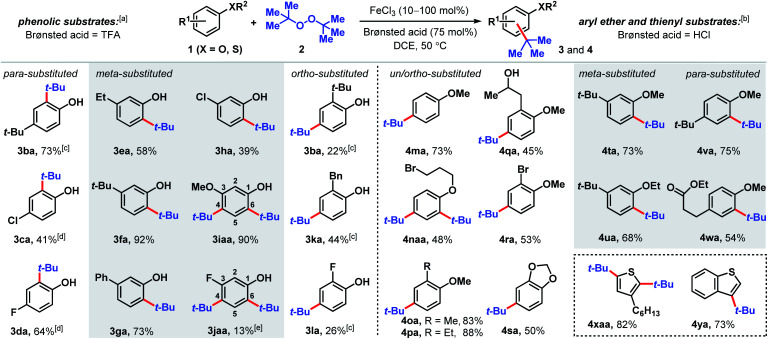
Scope of *tert*-butylation of phenolic, aryl ether, and thiophene derivatives. [a] Reaction conditions: arene 1 (0.2 mmol), DTBP (2, 0.2 mmol), FeCl_3_ (10 mol%), TFA (0.15 mmol), DCE (0.8 mmol), 50 °C, 2 h. [b] Reaction conditions: arene 1 (0.2 mmol), DTBP (2, 0.2 mmol), FeCl_3_ (10 mol%), HCl_(aq)_ (0.15 mmol), DCE (0.8 mmol), 50 °C, 2 h. [c] FeCl_3_ (20 mol%), 18 h. [d] FeCl_3_ (1 equiv.), HCl_(aq)_ (0.15 mmol), 48 h. [e] 2-*tert*-Butyl-5-fluorophenol isolated in 5% yield.

The Fenton reaction is a classic iron-catalyzed oxidation that employs peroxide reagents and a strong acid.^22^ Its reactivity has been elegantly exploited for aliphatic C–H functionalization to synthesize C–O^23^ and C–S^24^ bonds. In our investigations of the reactivity of aromatic C–H bonds under Fenton-inspired conditions, we observed that the treatment of phenolic substrates (1) with equimolar di-*tert*-butyl peroxide (DTBP, 2), trifluoroacetic acid, and catalytic FeCl_3_ led to site-selective C–C bond formations (Scheme 2, see ESI† for optimization data). This dual Brønsted/Lewis acid catalysis exerts considerably enhanced reactivity compared to a related iron-mediated system where the arene reagent was employed as the solvent.^25^

Substituted phenolic and anisolic substrates generally alkylate to yield one major isomeric product. Exposing 4-*tert*-butylphenol to DTBP (2) in the presence of iron(iii) and HCl catalysts yields 73% of 2,4-di-*tert*-butylphenol (3ba). 4-Chloro- and 4-fluorophenols require stoichiometric iron salts to proceed and are transformed into their alkylated counterparts 3ca and 3da in 41% and 64% yields, respectively. Under these reaction conditions, overoxidation to benzoquinone-type side-products accounts for some of the mass balance. *Meta* substituted phenols are alkylated exclusively at the less hindered position(s) *ortho* to the phenolic group. Both 3-ethyl and 3-*tert*-butylphenol are converted to *tert*-butylated 3ea and 3fa in 58% and 92% yields, respectively, the latter of which is confirmed by X-ray crystallography (see ESI†). The higher isolated yield obtained for 3fa is presumably due to the lack of benzylic hydrogens that can participate in hydrogen atom abstractions. 3-Phenylphenol, which also does not contain benzylic hydrogens, is transformed into the corresponding alkylated product (3ga) in 73% yield. The phenolic derivative bearing a *meta*-chloro substituent undergoes *tert*-alkylation to yield phenolic 3ha in a modest 39% yield. Contrary to phenolic substrates 1a–h that are monoalkylated at the less hindered *ortho* site, 3-methoxy- and 3-fluorophenol are *tert*-butylated at both the 4- and 6-positions to furnish tetrasubstituted phenols 3iaa and 3jaa, in 90% and 13% yields, respectively, with 1 equivalent of DTBP (2). *Ortho*-substituted phenolic substrates are considerably less reactive but are selectively *tert*-butylated *para* to the hydroxy group to yield 3ba, 3ka and 3la in 22–44% yields using higher iron loadings and extended reaction times.

Aryl ether and thiophene derivatives are better behaved in the dual iron(iii)/HCl catalyzed *tert*-butylation reaction (Scheme 2). Anisole is converted to 4-*tert*-butylanisole (4ma) in 73% yield. 2,4-Dialkylation occurs with bromopropyl phenyl ether to afford trisubstituted arene 4naa in 48% yield, with no monoalkylation product observed. *Ortho*-substituted anisole precursors are site-selectively functionalized *para* to the methoxy group. Unlike the 2-alkylphenolic derivatives, which are poorly reactive, 2-methyl- and 2-ethylanisoles undergo *tert*-butylation to give 4oa and 4pa in 83% and 88% yields, respectively. Anisole derivatives with an aliphatic alcohol or bromo group at the 2-position are transformed to their corresponding *tert*-butylated products in moderate yields (45% for 4qa and 53% for 4ra). New C(sp^2^)–C(sp^3^) bond formation occurs with benzodioxole, albeit less effectively than with anisole, producing 4sa in 50% yield. 3-Substituted aryl ethers are functionalized selectively to products 4ta and 4ua with alkylation at the *ortho* positions in 68–73% yields. Selective mono-*tert*-butylation proceeds with 4-*tert*-butylanisole to deliver 2,4-di-*tert*-butylanisole (4va) in 75% yield. An anisole derivative bearing a pendant ester group is accommodated and 54% of the alkylated product (4wa) is formed. In addition to anisole derivatives, thiophene derivatives react effectively. Treating 3-hexylthiophene with DTBP (2) under iron(iii)/HCl catalysis favors di-*tert*-butylation at both the 2- and 5-positions (4xaa, 82%), whereas the analogous reaction with benzothiophene leads to selective *tert*-butylation at the 3-position in 73% yield (4ya). In contrast, the phosphoric acid-mediated direct alkylation of thiophene derivatives with *tert*-butanol requires 200 °C to achieve modest yields.^19*a*^

**Scheme 3:**
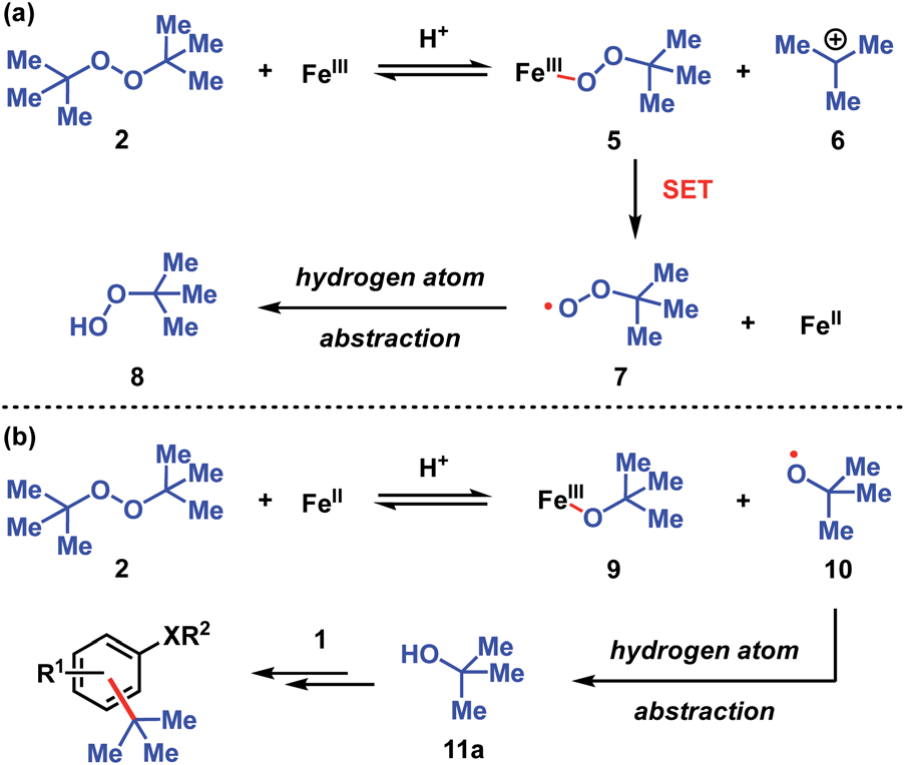
Proposed pathways for the decomposition of DTBP (2). (a) Fe(iii) initiated pathway. (b) Fe(ii) initiated pathway.

**Fig. 2:**
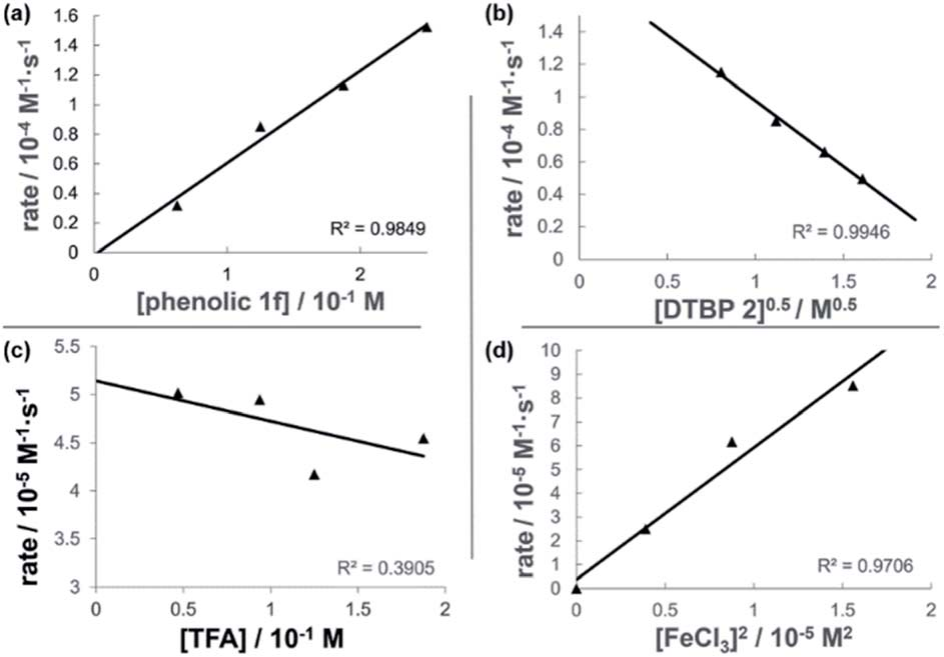
Plots of initial rates with respect to (a) [3-*tert*-butylphenol 1f] indicating approximate first-order dependence, [DTBP 2] = 0.13 M, [FeCl_3_] = 0.012 M, [TFA] = 0.094 M; (b) [DTBP 2]^0.5^ indicating half-order dependence [1f], = 0.12 M, [FeCl_3_] 0.012 M, [TFA] = 0.094 M; (c) [TFA] suggestive of zero-order dependence [1f], = 0.12 M, [DTBP 2] = 0.13 M, [FeCl_3_] = 0.012 M; (d) [FeCl_3_]^2^ indicating second-order dependence [1f], = 0.12 M, [DTBP] = 0.13 M, [TFA] = 0.094 M. Each data point was measured in triplicate.

**Fig. 3:**
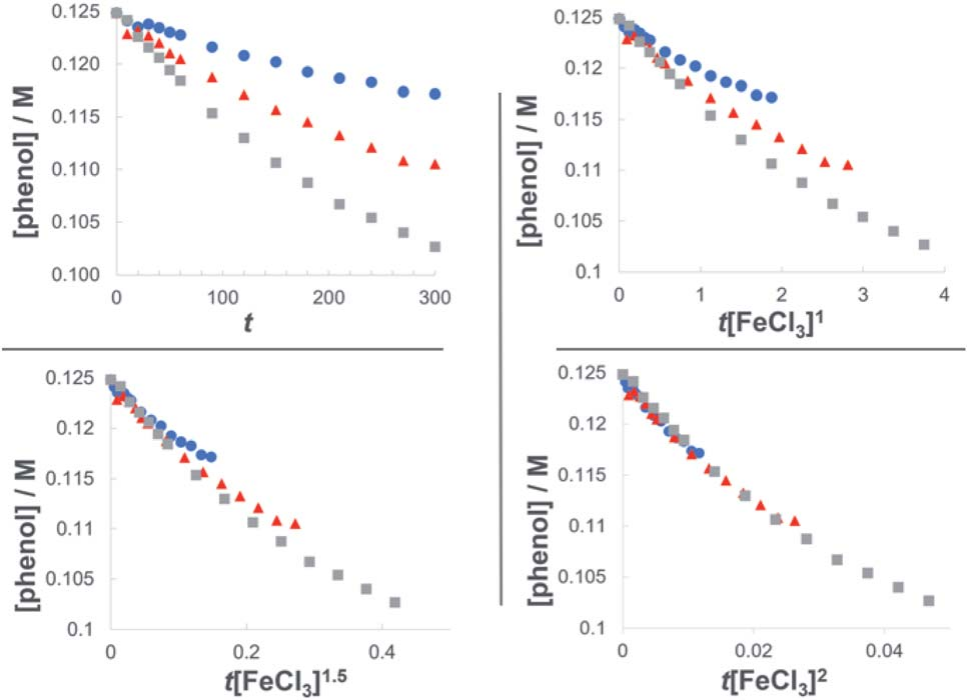
Plots of the normalized time scale method for determining catalyst order; blue = 0.0062 M FeCl_3_, orange = 0.0094 M FeCl_3_, grey = 0.013 M FeCl_3_.

The dual Brønsted/Lewis acid catalyzed cross-coupling between electron-rich arenes and DTBP (2) represents an underexplored site-selective Friedel–Crafts alkylation process. However, the modest reactivity experienced by several substrates and the reliance on DTBP (2) limit synthetic practicality. We speculate side reaction pathways arising from radical species compromise reactivity and product yields. In a proposed pathway, analogous to that with hydrogen peroxide (Scheme 3a),^26^ DTBP (2) can react with iron(iii) to form iron(iii) *tert*-butylperoxide (5) and *tert*-butyl cation (6), the latter of which can participate in the desired electrophilic alkylation. Single electron transfer with the former would lead to iron(ii) and *tert*-butylperoxyl radical (7), which could abstract a hydrogen atom from the solvent or substrate to give *tert*-butyl hydroperoxide (8), which also promotes this reaction, albeit less effectively than DTBP (2). Alternatively, iron(ii) produced in this manner, or through reduction of iron(iii) by phenol and anisole derivatives,^27,28^ can reduce DTBP (2) in a Fenton-like fashion to generate iron(iii) (9) and *tert*-butoxyl radical (10, Scheme 3b). Subsequent hydrogen atom abstraction by the oxygen-centered radical may initiate undesirable side reactions while producing *tert*-butanol (11a), a potential precursor to the desired Friedel–Crafts reaction. We find catalysis with FeCl_2_ proceeds similarly to FeCl_3_, which is consistent with a Fenton-initiation process. A kinetic analysis was undertaken to derive insight into optimizing the C(sp^2^)–C(sp^3^) cross-coupling reaction. 3-*tert*-Butylphenol (1f) was selected as the model substrate to react with DTBP (2) because little-to-no side products form over the course of the reaction, thus simplifying the data analysis and interpretation. Initial rates for *tert*-butylation were then measured by varying the concentrations 1f, DTBP (2), TFA, and FeCl_3_ catalyst. A first-order rate dependence on the concentration of phenolic 1f was observed (Fig. 2a). The kinetics experiments revealed a half-order dependence with respect to the concentration of DTBP (2) (Fig. 2b), suggestive of 2 dissociating into two active fragments and consistent with the mechanistic hypotheses presented in Scheme 3. Little change in initial rates were observed with varying TFA concentrations, which we interpret as zero-order rate dependence (Fig. 2c). TFA may play a role in forming the active catalyst, potentially as a ligand. With respect to FeCl_3_, a relatively uncommon second order dependence on rate was observed (Fig. 2d).^29^ Additional evidence for the catalyst order was sought by treating the reaction profile data to graphical analysis using the normalized time scale method.^30^ Rather than converting the raw data to rates, the raw concentration data of the entire data sets (*i.e.* [1f],) were plotted against normalized time scales, *t*[FeCl_3_]^*n*^, where *t* = time and *n* corresponds to the catalyst order when all the curves overlay on one another (Fig. 3). Using the data sets obtained from varying the catalyst loadings, the curves overlay when *n* = 2, which support a second order dependence in [FeCl_3_] and is consistent with a tandem iron-catalyzed process.^31,32^ Therein, the catalyst plays distinct roles in transforming DTBP (2) into the reactive alkylating agent, potentially *tert*-butanol (11a), and further activates it for merger with the arene coupling partner. The latter activation of *tert*-butanol for arene alkylation is potentially the turnover-limiting step and would be consistent with the rate law, *k*[phenol]^1^[DTBP]^0.5^[FeCl_3_]^2^[TFA]^0^. Based on this mechanistic conjecture, DTBP (2) could be substituted with *tert*-alkanols. While catalytic *tert*-alkylations using allylic, propargylic, and benzylic alcohols are well precedented,^18^ few examples exist with unactivated *tert*-alkanols, especially in the context of site-selectivity.^18,19^ We envisage that the process involving a synergistic combination of Fe(iii) and Brønsted acid catalysts would address the synthetic limitations imposed by using peroxides as coupling reagents, and would provide a simple approach for directly forging C(sp^2^)–C(sp^3^) bonds with quaternary carbon centers.

**Scheme 4:**
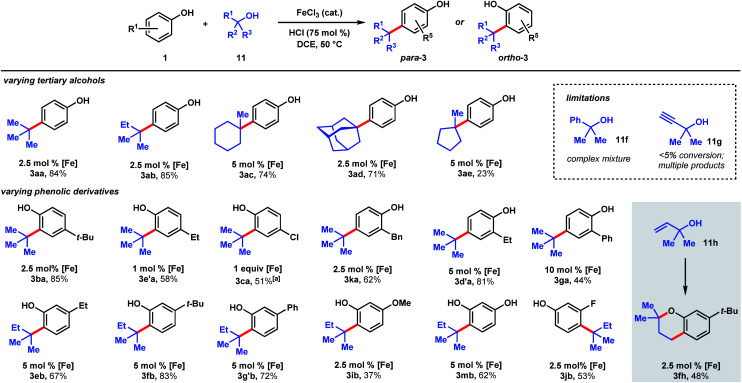
Scope of dual Brønsted/Lewis acid catalyzed, C(sp^2^)–C(sp^3^) coupling between phenolic and tertiary alcohol derivatives. [a] 2 equiv. 11a, 48 h.

We targeted the joining of 2-methyl-2-butanol (11b) and 3-*tert*-butylphenol (1f) to investigate our hypothesis (Table 1). The use of 2.5 mol% FeCl_3_ and 75 mol% HCl in DCE solvent afforded 72% yield of target 3fb (entry 1). Only 10% product was formed in the absence of HCl. In contrast to the reactions with DTBP (2), *tert*-alkylation does not occur with trifluoroacetic acid as the co-catalyst (entry 2), while 66% NMR yield was obtained with HBr (entry 3). Using FeCl_2_ instead of FeCl_3_ resulted in a significant drop in conversion to 15% (entry 4). FeBr_3_ (entry 5) and FeBr_2_ (entry 6) performed similarly to FeCl_3_ (70% yields). The use of Fe(OTf)_2_ provided modest reactivity when combined with HCl (46%, entry 7), and no reactivity without HCl. Increasing or decreasing the amounts of acid led to inferior 63% and 60% yields, respectively (entries 8 and 9). Exchanging the solvent for HFIP resulted in only 13% conversion (entry 10). The reaction proceeded similarly in chlorobenzene solvent (75%, entry 11). When performed in toluene, moderate levels of product formation were observed (43%, entry 12); the lower yield is attributed to toluene being reactive, which consumes a significant proportion of the alcohol. Isopropanol and THF solvents do not promote the desired alkylation (entries 13 and 14). Considering reagent cost and operation simplicity, we elected to use FeCl_3_, HCl, and DCE solvent as the optimal conditions to explore the substrate scope. These reactions can be set up under air. Moisture does not affect reactivity and aqueous HCl can be used as the source of Brønsted acid. The unique reactivity arising from the combination of FeCl_3_ and HCl previously observed in a cation–π polycyclization has been attributed to the formation of HFeCl_4_.^33^ The desired *tert*-alkylation reactions are not restricted to phenolic compounds, but also to anisolic and electron-neutral arenes, in which cases the combination of FeBr_3_ and HBr catalysts were found to be the optimal catalysts (see ESI† for optimization studies). Product formation was not observed in the absence of iron catalyst and the use of AlCl_3_ in place of FeX_3_/HX resulted in complex mixtures.

**Table 1:** Survey of conditions for direct Friedel–Crafts alkylation with phenolic 1f and tertiary alcohol 11b^a^

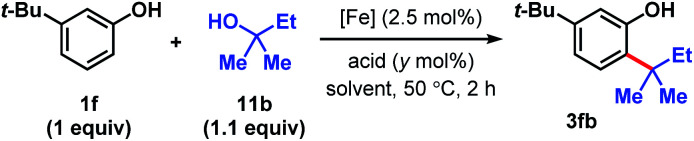					
	[Fe]	Acid	*y*	Solvent	% Yield^b^
1	FeCl_3_	HCl_(aq)_	75	DCE	72 (10)^c^
2	FeCl_3_	CF_3_COOH	75	DCE	0
3	FeCl_3_	HBr_(aq)_	75	DCE	66
4	FeCl_2_	HCl_(aq)_	75	DCE	15
5	FeBr_3_	HCl_(aq)_	75	DCE	70
6	FeBr_2_	HCl_(aq)_	75	DCE	70
7	Fe(OTf)_2_	HCl_(aq)_	75	DCE	46 (<5)^c^
8	FeCl_3_	HCl_(aq)_	50	DCE	63
9	FeCl_3_	HCl_(aq)_	100	DCE	60
10	FeCl_3_	HCl_(aq)_	75	HFIP	13
11	FeCl_3_	HCl_(aq)_	75	PhCl	75
12	FeCl_3_	HCl_(aq)_	75	PhMe	43
13	FeCl_3_	HCl_(aq)_	75	IPA	0
14	FeCl_3_	HCl_(aq)_	75	THF	0

a Conditions: all reactions performed on 0.2 mmol scale, phenol (1 equiv.), alcohol (1.1 equiv.), 0.25 M, 50 °C, 24 h.

b Determined by NMR analysis of the crude reaction mixture using 1,3,5-trimethoxybenzene as internal standard.

c Without Brønsted acid.

The ability to use tertiary alcohols enables various alkyl groups to be added (Scheme 4). Alkylation of phenol (1) occurred selectively at the *para* position, affording 3aa–3ad in 71–85% yields. Adamantane is a privileged structure that has earned the reputation of being a “lipophilic bullet” for enhancing pharmacological activity^34^ and various methods have been devised for their derivatization,^35^ including a Friedel–Crafts strategy that requires trifluoroacetic acid as the solvent.^36^ Here, dual FeCl_3_/HCl catalysis allows arylation of 1-adamantanol under mild reaction conditions. Surprisingly, 1-methylcyclopentanol (11e) turned out to be a poor alkylating agent that only gave 23% yield of *para*-methylcyclopentyl phenol (3ae) even with a higher catalyst loading. Analysis of the reaction mixture revealed the major side product to be cyclopentene. Presumably, the dehydration pathway is facile, and the reverse hydration step is unfavorable under the reaction conditions. Using *tert*-butanol (11a), alkylation of 4-*tert*-butylphenol furnishes di-*tert*-butylphenol (3ba) in 85% yield, while 4-ethylphenol was alkylated to yield 3e′a in 58% yield at 1 mol% FeCl_3_ loading. 4-Chlorophenol required 1 equivalent of FeCl_3_ to achieve 51% yield of 3ca. 2-Benzyl-, 2-ethyl-, and 2-phenylphenol were alkylated in moderate-to-good yields (44–81%) to give 3ka, 3d′a, and 3ga, respectively. Some substrates require higher catalyst loadings (*e.g.*, 2-ethylphenol and 2-phenylphenol) to achieve high reactivity, but absent of a trend. Minor amounts of dialkylation side products were isolated (see ESI†). *tert*-Alkylation of *m*eta-substituted phenols were examined using 2-methyl-2-propanol (11b). At 5 mol% catalyst loading, 3-ethyl-, 3-*tert*-butyl-, and 3-phenylphenol are converted to disubstituted phenols 3eb, 3fb, 3g′b in 67–83% yields. 3-Methoxyphenol is converted to 3ib in 37% yield and alkylated resorcinol 3mb is synthesized in 62% yield. Unlike other *meta*-substituted phenols, 3-fluorophenol is *tert*-alkylated *para* to the hydroxy group in 53% yield (3jb). When reacted with phenol (1a), tertiary benzylic (11f) and propargylic (11g) alcohols, normally successful in Friedel–Crafts alkylations, converted to multiple products that could not be purified to homogeneity. With dimethylvinylcarbinol (11h), *C*-alkylation followed by cyclization was observed with 3-*tert*-butylphenol to produce chromane 3fh in 48% yield.

**Scheme 5:**
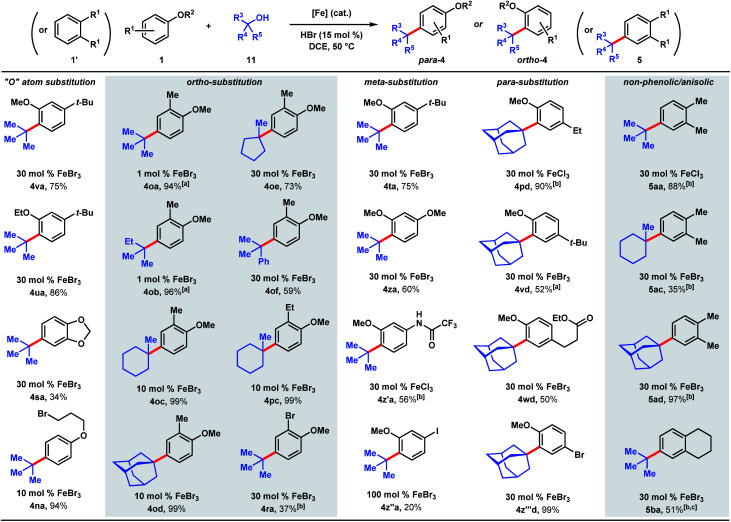
Scope of dual Brønsted/Lewis acid-catalyzed, C(sp^2^)–C(sp^3^) coupling of arene and tertiary alcohol derivatives. [a] 10 mol% HBr. [b] 75 mol% HCl. [c] Isolated as a 2.6 : 1 mixture of product/starting material.

We next examined the alkylation of aryl ethers and simple arenes (Scheme 5). 3-*tert*-Butylanisole is selectively alkylated at the less sterically encumbered *ortho* position with respect to the methoxy group (4va, 75%). Swapping the methyl ether with an ethyl ether yields product 4ua in 86%. However, 1,2-benzodioxole (4sa) is *tert*-butylated in a modest 34% yield. A primary halide tethered off the ether linkage does not hinder the reaction and results in 94% yield of 4na. A variety of tertiary alcohols were tested to alkylate 2-methylanisole (1o). Most of the alcohols deliver the alkylated products (4oa–4od) in near quantitative yields (94–99%) with low catalyst loadings: 1 mol% for *tert*-butanol (11a) and *tert*-amyl alcohol (11b), and 10 mol% for methylcyclohexanol (11c) and adamantanol (11d). Methylcyclopentanol (11e) and cumyl alcohol (11f), substrates that reacted poorly with phenol (*cf.*Scheme 5), requires 30 mol% iron and yields alkylated 4oe and 4of in 73% and 59%, respectively. Alkylation of 2-ethylanisole with methyl-cyclohexanol provides 4pc in 99% yield. 2-Bromoanisole is considerably less reactive, leading to alkylated 4ra in 37% yield with a full equivalent of FeBr_3_. While most of the *meta*-substituted anisole derivatives are alkylated to 4ta, 4za, and 4z′a in moderate yields (56–75%) with catalytic FeBr_3_, 3-iodo-anisole requires a full equivalent of FeBr_3_, and furnished the product (4z′′a) in 20% yield. *tert*-Alkylation of 4-ethylanisole led to product 4pd in 90% yield, but 4-*tert*-butylanisole turned out to be a more challenging substrate, likely owing to the added steric bulk, forming alkylation product 4vd in 52% yield. The reaction accommodates esters, providing product 4wd in 50% yield. In contrast to previously studied halogenated arenes, 4-bromoanisole was converted to product 4z′′′d in quantitative yield. This *tert*-alkylation reaction is not confined to phenolic and aryl ether substrates. *Ortho*-xylene and tetralin are alkylated to provide arenes 5aa, 5ac, 5ad, and 5ba in 35–97% yields. In contrast to the TFA/FeCl_3_ system where the kinetics are well-behaved (see Scheme 2 & Fig. 2), the occurrence of induction periods that complicate the kinetics analysis are observed with the HCl/FeCl_3_ pair. The reaction rates during the acceleration periods following the induction periods are invariably constant and do not appear to be affected by concentrations of FeCl_3_, HCl, phenolic substrate, or *t*-butanol, thereby resembling zero-order behaviors in all cases (see ESI†).

**Fig. 4:**
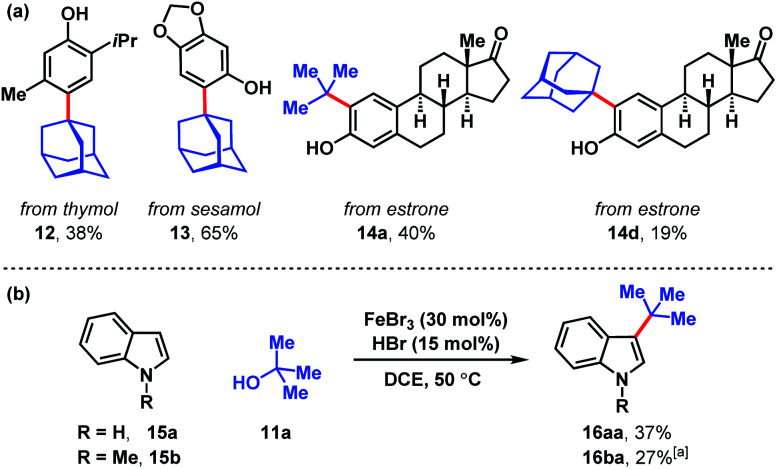
(a) Late-stage *tert*-alkylation of natural products. Conditions: FeCl_3_ (5 mol%), *tert*-alcohol (1.1 equiv.), HCl (75 mol%), PhCl, 100 °C. (b) *tert*-Alkylation of indoles. Conditions: FeBr_3_ (5 mol%), *tert*-butanol (1 equiv.), HBr (15 mol%), DCE, 50 °C. [a] Isolated together with 14% *N*-methyl-3,5-di-*tert*-butylindole (see ESI†).

Several naturally occurring compounds were subjected to late-stage *tert*-alkylation (Fig. 4a). Initially, the compounds tested performed poorly due to low solubility in DCE at 50 °C. However, useful yields resulted by changing the solvent to chlorobenzene and heating to 100 °C. Thymol and sesamol are adamantylated to produce functionalized 12 and 13 in 38% and 65% yields, respectively. The relatively more complex molecule, estrone, undergoes *tert*-butylation in 40% yield (14a) and adamantylation in 19% yield (14d). Free indole (15a) and *N*-methylindole (15b) can be *tert*-butylated with catalytic FeBr_3_/HBr to produce 3-*tert*-butylindoles 16aa and 16ba in modest yields (27–37%, Fig. 4b).

**Scheme 6:**
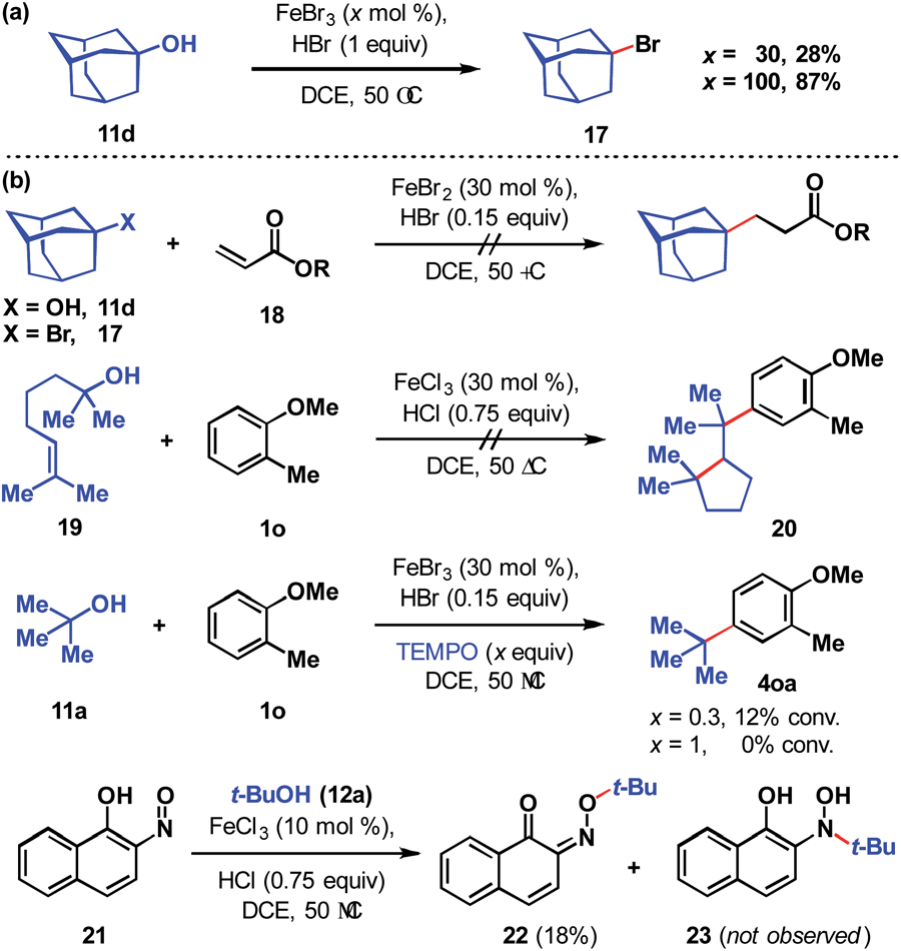
(a) Fate of the alcohol. (b) Probing for a radical *vs.* polar pathway.

To assess the stability of the tertiary alcohol under the reaction conditions, we exposed 1-adamantanol (11d) to dual Brønsted/Lewis acid catalysis conditions (Scheme 6a). In the absence of the arene substrate, 1-bromoadamantane (17) was isolated in 28% yield. Subjecting the same reaction to 1 equivalent of FeBr_3_ increased the yield to 87%. To probe whether the reaction proceeds through a closed- or open-shell pathway, we investigated the capturing of putative radical intermediates using various Michael acceptors 18 (Scheme 6b). The potential for a 1-electron reduction of the newly formed carbon–halogen bond using an iron(ii) catalyst was examined. However, attempts to generate radical species from both 1-adamantanol (11d) and 1-bromoadamantane (17) were deemed unsuccessful as alkyl addition to the Michael acceptors was not observed. Initially, methyl acrylate and phenyl acrylate were tested, however both proved ineffective, as did others that were investigated (see ESI†). If a radical intermediate forms from alkenol 19, the resulting tertiary radical could cyclize onto the alkene, but attempts to react it with 2-methylanisole (1o) resulted in a mixture of products with no indication of cyclization to cyclopentyl 20. Addition of substoichiometric TEMPO reduced reactivity to 12% conversion and stoichiometric TEMPO halted reactivity. However, in the absence of other compelling data, we interpret this as a competitive interaction between TEMPO and the iron reagent that leads to catalyst arrest.^37^ This is supported by the lack of TEMPO-adducts observed, which are otherwise expected to form from the quenching of arene or tertiary alkyl radical species. While less common than TEMPO, nitroso compounds exert radical scavenging properties.^38^ As such, we rationalized that 2-nitroso-1-naphthol (21) could potentially differentiate radical and polar pathways. The donating capacity of the phenolic group could render the nitroso functionality reactive towards polar electrophiles to give oxime ether 22. Alternatively, radical intermediates would engage the nitroso group to arrive at hydroxylamine 23. Under the reaction conditions, only oxime ether 22 was formed in 18% yield, with the remainder of the mass balance attributed to unreacted starting materials. Amine 23 was not detected in the reaction mixture. In addition, we employed density functional theory (DFT) calculations with energies refined at the B2PLYP-D3/def2-TZVPPD level of theory^39^ to assess the thermodynamics of closed- and open-shell pathways for activation of *t*-BuOH by FeCl_3_ through a polar pathway or FeCl_2_ through a radical pathway using eqn (1) (Δ*G*°/Δ*H*° = 15.8/18.2 kcal mol^−1^) and (2) (Δ*G*°/Δ*H*° = 42.5/46.7 kcal mol^−1^), respectively:

1 *t*-BuOH + FeCl_3_ → *t*-Bu^+^ + [FeCl_3_OH]^−^

2 *t*-BuOH + FeCl_2_ → *t*-Bu˙ + FeCl_2_OH

The reaction between *t*-BuOH and FeCl_3_ to form *tert*-butyl cation is computed to be lower in free energy by 26.7 kcal mol^−1^, suggesting it is far more likely to occur. Considering the reaction is run in the presence of a strong Brønsted acid, we also examined how protonation of the alcohol group affects these energetics. First, protonation of the alcohol group by HCl is predicted to be significantly thermodynamically uphill (Δ*G*° = 28.8 kcal mol^−1^). The free energy for subsequent cleavage of the C–O bond in the presence of FeCl_3_ and FeCl_2_ are computed using eqn (3) (Δ*G*°/Δ*H*° = −5.8/−4.0 kcal mol^−1^) and (4) (Δ*G*°/Δ*H*° = 40.1/44.1 kcal mol^−1^):

3 *t*-BuOH_2_^+^ + FeCl_3_ → *t*Bu^+^ + FeCl_3_OH_2_

4 *t*-BuOH_2_^+^ + FeCl_2_ → *t*Bu˙ + [FeCl_2_OH_2_]^+^

The reaction in eqn (3) is lower in free energy than the reaction in eqn (4) by 45.9 kcal mol^−1^, suggesting that the effect of protonating the alcohol renders the polar pathway even more likely. Based on these studies, we propose this reaction proceeds *via* a polar Friedel–Crafts type mechanism.

**Fig. 5:**
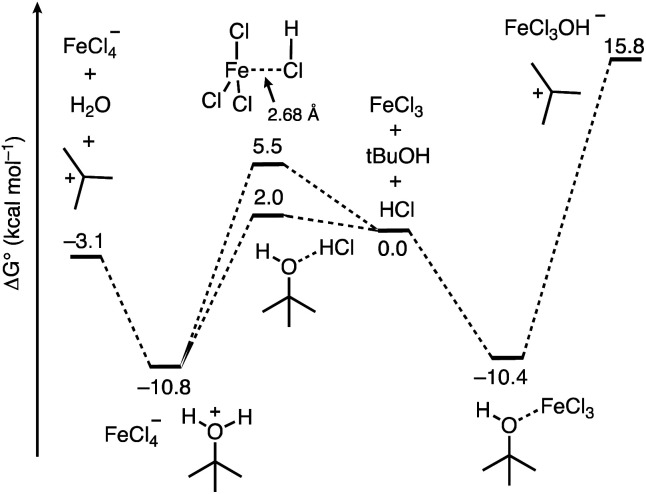
Free energy profile computed using DFT calculations for the course of ionization of *t*-BuOH in the presence of the FeCl_3_/HCl acid pair and FeCl_3_.

From here, we next sought to gain insight into the course of the reaction (Fig. 5). We first computed the association complexes between FeCl_3_ and other components in the reaction. All attempts to locate a structure for “HFeCl_4_” through coordination of HCl to the iron center of FeCl_3_ led to dissociation of the HCl upon optimization. This indicates that HFeCl_4_ is not a well-defined minimum on the potential energy surface at this level of theory. In addition, the formation of the HCl⋯FeCl_3_ association complex is uphill (Δ*G*°/Δ*H*° = 5.5/−1.8 kcal mol^−1^). We found that the most stable 1 : 1 complex is between *t*-BuOH and FeCl_3_ (*t*-BuOH + FeCl_3_ → *t*-BuOH–FeCl_3_) where Δ*G*°/Δ*H*° = −10.4/−21.7 kcal mol^−1^. Direct ionization from this complex to form the *tert*-butyl cation is significantly thermodynamically uphill (Δ*G*°/Δ*H*° = 26.2/39.9 kcal mol^−1^), which is consistent with how FeCl_3_ has not been successful in catalyzing transformations with unactivated *tert*-alkanols.^20,21,40^ Additionally, the role of HCl in this process is unclear. Alternatively, HCl association with *t*-BuOH to form a hydrogen bonded complex is slightly unfavored (*t*-BuOH + HCl → *t*-BuOH–HCl) where Δ*G*°/Δ*H*° = 2.0/−5.4 kcal mol^−1^. However, putting FeCl_3_ near the HCl and optimizing the geometry results in spontaneous protonation of the alcohol to form the *t*-BuOH_2_^+^⋯[FeCl_4_]^−^ ion pair. The ion pair is lower in free energy than the hydrogen bonded complex by 12.8 kcal mol^−1^, indicating that FeCl_3_-facilitated protonation of the alcohol is competitive with direct coordination of FeCl_3_ to *t*-BuOH. From the ion pair, ionization to the *tert*-butyl cation is only 7.7 kcal mol^−1^ uphill. Thus, the combination of FeCl_3_ and HCl provides a low energy pathway to the formation of the reactive *tert*-butyl cation.

The results in Fig. 5 imply that the basis for the FeCl_3_ Lewis acid additive increases the Brønsted acidity of HCl despite the lack of a discrete structure for “HFeCl_4_”. This is reminiscent of the HF/BF_3_ pair that is sometimes referred to as HBF_4_, for which there is no expected discrete structure.^41^ We next sought to quantify the extent of increased Brønsted acidity imparted by the inclusion of the Lewis acid additive for several Brønsted acid/Lewis acid (HA/L) pairs. We used the reaction shown in eqn (5), where HA is the Brønsted acid, L represents the Lewis acid additive, and HA-L represents a complex formed between them:

5 HA–L + A^−^ → HA + A–L^−^

The HA/L pairs studied are HCl/FeCl_3_, HBr/FeBr_3_, CF_3_COOH/FeCl_3_, as well as HF/BF_3_ (Table 2). It should be noted that for the binary mineral acids studied, the HA–L is not stable relative to the separated HA and L species and so the energy calculated from eqn (5) corresponds with the complexation energy between A^−^ and L.

**Table 2:** Free energy calculated from eqn (5) to estimate the increased Brønsted acidity for Brønsted acid/Lewis acid pairs (HA/L) discussed in this study

HA/L	Δ*G*°, eqn (5) (kcal mol^−1^)	Δp*K*_a_
HCl/FeCl_3_	−31.4	23
HBr/FeBr_3_	−28.6	21
CF_3_COOH/FeCl_3_	−24.7	18
HF/BF_3_	−47.5	35

The data in Table 2 show that the added Lewis acid has a substantial effect on the acidity of the Brønsted acids. FeCl_3_ provides more stabilization to the chloride ion than to trifluoroacetate (ΔΔ*G*° = −6.7 kcal mol^−1^) and more stabilization than FeBr_3_ provides to the bromide ion (ΔΔ*G*° = −2.8 kcal mol^−1^). In addition, the largest increase is achieved for the HF/BF_3_ pair. These results suggest a synergistic effect between the conjugate base and Lewis acid may be a significant factor for determining the increase in Brønsted acidity.

We next used eqn (6) to gain a better sense for the acidity of the HA/L pairs relative to HCl/FeCl_3_ to assess their overall reactivity:

6 HA–L + FeCl_4_^−^ → [A–L]^−^ + HCl + FeCl_3_.

For the HBr/FeBr_3_ pair, Δ*G*°/Δ*H*° = −2.9/−3.2 kcal mol^−1^, which is consistent with our experimental results suggesting this pair to be more reactive. However, this value is about half as much as one would expect based on the relative p*K*_a_ values of HCl and HBr in DCE, (Δp*K*_a_[DCE], HBr–HCl = 4.5).^42^ The other two combinations are predicted to be less reactive than HCl/FeCl_3_, where the CF_3_COOH/FeCl_3_ and HF/BF_3_ combinations give Δ*G*°/Δ*H*° = 8.4/16.8 and 7.5/7.1 kcal mol^−1^, respectively. The former case is consistent with experimental results from Table 1 (entry 2) showing no product formation with the CF_3_COOH/FeCl_3_ pair. The use of HBF_4_ (2.5 mol%) as the catalyst resulted in only trace product formation (<5% by ^1^H NMR analysis). These results suggest that the pairing of a Lewis acid with a Brønsted acid generally increases the Brønsted acidity significantly in organic media, and that careful choice of the pairing could provide a level of control over the overall reactivity of the pair.

## Conclusions

We have detailed mild and operationally simple reaction conditions to achieve *tert*-alkylations of aromatic systems with tertiary alkylperoxides and alcohols in forming all-carbon quaternary centers through synergistic Brønsted/Lewis acid catalysis. These reactions fill a gap in the Friedel–Crafts alkylation literature by enabling the use of tertiary aliphatic alcohols that lack stabilizing aryl, alkenyl, and alkynyl substituents. We expect that this approach will prove to be practical in installing quaternary carbon centers when orchestrated into synthesis plans that take advantage of C–O bonds (*e.g.*, triflyl and methoxy groups) for cross-coupling applications.^43^ The use of cost-effective and readily-available iron, alcohol and arene reagents render this methodology advantageous for all-carbon quaternary center and C(sp^2^)–C(sp^3^) bond synthesis.

## Data availability

Procedures including preparation of substrates, optimization of catalysis conditions, characterization data, kinetics and DFT data, X-Ray crystallographic data, and NMR spectra.

## Author contributions

A. P., M. C., R. C., and L. G. conducted the catalysis experiments and the characterization of the compounds. B. E. H. designed and performed the computational experiments. K. G. M. K conceived, designed, and oversaw the project. All authors contributed to writing and editing the manuscript.

## Conflicts of interest

There are no conflicts to declare.

## Supplementary Material

SC-013-D1SC06422C-s001

SC-013-D1SC06422C-s002

## References

[cit1] Lovering F., Bikker J., Humblet C. (2009). J. Med. Chem..

[cit2] Méndez-Lucio O., Medina-Franco J. L. (2017). Drug Discovery Today.

[cit3] Long R., Huang J., Gong J., Yang Z. (2015). Nat. Prod. Rep..

[cit4] Douglas C. J., Overman L. E. (2004). Proc. Natl. Acad. Sci. U. S. A..

[cit5] Lohre C., Dröge T., Wang C., Glorius F. (2011). Chem.–Eur. J..

[cit6] Zultanski S. L., Fu G. C. (2013). J. Am. Chem. Soc..

[cit7] Primer D. N., Molander G. A. (2017). J. Am. Chem. Soc..

[cit8] Wang X., Wang S., Xue W., Gong H. (2015). J. Am. Chem. Soc..

[cit9] Chen T.-G., Zhang H., Mykhailiuk P. K., Merchant R. R., Smith C. A., Qin T., Baran P. S. (2019). Angew. Chem., Int. Ed..

[cit10] Green S. A., Huffman T. R., McCourt R. O., van der Puyl V., Shenvi R. A. (2019). J. Am. Chem. Soc..

[cit11] Dorsheimer J. R., Ashley M. A., Rovis T. (2021). J. Am. Chem. Soc..

[cit12] Luchaco-Cullis C. A., Mizutani H., Murphy K. E., Hoveyda A. H. (2001). Angew. Chem., Int. Ed..

[cit13] Shimizu Y., Shi S.-L., Usuda H., Kanai M., Shibasaki M. (2010). Angew. Chem., Int. Ed..

[cit14] Iovel I., Mertins K., Kischel J., Zapf A., Beller M. (2005). Angew. Chem., Int. Ed..

[cit15] Jefferies L. R., Cook S. P. (2014). Org. Lett..

[cit16] Zhang S., Vayer M., Noël F., Vuković V. D., Golushko A., Rezajooei N., Rowley C. N., Lebœuf D., Moran J. (2021). Chem.

[cit17] Rueping M., Koenigs R. M., Atodiresei I. (2010). Chem.–Eur. J..

[cit18] Liu Y., Kim B., Taylor S. D. (2007). J. Org. Chem..

[cit19] Kutz W. M., Corson B. B. (1946). J. Am. Chem. Soc..

[cit20] Rueping M., Nachtsheim B. J. (2010). Beilstein J. Org. Chem..

[cit21] Ricardo C. L., Mo X., McCubbin J. A., Hall D. G. (2015). Chem.–Eur. J..

[cit22] Wardman P., Candeias L. P. (1996). Radiat. Res..

[cit23] Kim C., Chen K., Kim J., Que Jr L. (1997). J. Am. Chem. Soc..

[cit24] Groendyke B. J., Modak A., Cook S. P. (2019). J. Org. Chem..

[cit25] Liguori L., Bjørsvik H.-R., Fontana F., Bosco D., Galimberti L., Minisci F. (1999). J. Org. Chem..

[cit26] Perez-Benito J. F. (2004). J. Phys. Chem. A.

[cit27] Shalit H., Libman A., Pappo D. (2017). J. Am. Chem. Soc..

[cit28] Horibe T., Ohmura S., Ishihara K. (2019). J. Am. Chem. Soc..

[cit29] Albright H., Riehl P. S., McAtee C. C., Reid J. P., Ludwig J. R., Karp L. A., Zimmerman P. M., Sigman M. S., Schindler C. S. (2019). J. Am. Chem. Soc..

[cit30] Burés J. (2016). Angew. Chem., Int. Ed..

[cit31] Fogg D. E., dos Santos E. N. (2004). Coord. Chem. Rev..

[cit32] Kou K. G. M., Dong V. M. (2015). Org. Biomol. Chem..

[cit33] Elkin M., Szewczyk S. M., Scruse A. C., Newhouse T. R. (2017). J. Am. Chem. Soc..

[cit34] Wanka L., Iqbal K., Schreiner P. R. (2013). Chem. Rev..

[cit35] Lao Y.-X., Wu J.-Q., Chen Y., Zhang S.-S., Li Q., Wang H. (2015). Org. Chem. Front..

[cit36] Lu D., Meng Z., Thakur G. A., Fan P., Steed J., Tartal C. L., Hurst D. P., Reggio P. H., Deschamps J. R., Parrish D. A., George C., Järbe T. U. C., Lamb R. J., Makriyannis A. (2005). J. Med. Chem..

[cit37] Van Humbeck J. F., Simonovich S. P., Knowles R. R., MacMillan D. W. C. (2010). J. Am. Chem. Soc..

[cit38] de Boer Th. J. (1982). Can. J. Chem..

[cit39] Energies are computed at the B2PLYPD3/def2-TZVPPD//M15L/def2-SVP level of theory. Refined energies include solvation effects with the IEF-PCM model with 1,2-dichloroethane as the solvent and are corrected to a 1 M standard state. See ESI† for full description of computational methods

[cit40] Bauer I., Knölker H.-J. (2015). Chem. Rev..

[cit41] Juhasz M., Hoffmann S., Stoyanov E., Kim K.-C., Reed C. A. (2004). Angew. Chem., Int. Ed..

[cit42] Kütt A., Rodima T., Saame J., Raamat E., Mäemets V., Kaljurand I., Koppel I. A., Garlyauskayte R. Y., Yagupolskii Y. L., Yagupolskii L. M., Bernhardt E., Willner H., Leito I. (2011). J. Org. Chem..

[cit43] Rosen B. M., Quasdorf K. W., Wilson D. A., Zhang N., Resmerita A. M., Garg N. K., Percec V. (2011). Chem. Rev..

