# Supplementary material for: Synergistic Brønsted/Lewis acid catalyzed aromatic alkylation with unactivated tertiary alcohols or di-tert-butylperoxide to synthesize quaternary carbon centers
Source: Chem Sci. 2022 Mar 8;13(12):3539–48. doi: 10.1039/d1sc06422c (PMC8943850; doi:10.1039/d1sc06422c)
Supplement: SC-013-D1SC06422C-s001 [file SC-013-D1SC06422C-s001.pdf]

Electronic Supplementary Material (ESI) for Chemical Science.
This journal is © The Royal Society of Chemistry 2022

Supporting Information

Synergistic Brønsted/Lewis Acid Catalyzed Aromatic Alkylation with Unactivated Tertiary Alcohols or Di-*tert*-Butylperoxide to Synthesize Quaternary Carbon Centers

Aaron Pan,[+] Maja Chojnacka,[+] Robert Crowley III, Lucas Göttemann, Brandon E. Haines[*] and Kevin G. M. Kou[*]

Table of Contents

1. General Information 2
i) Solvents and reagents 2
ii) Reaction setup, progress monitoring, and product purification 2
2. Selected Reaction Optimization Data for Arene *tert*-Butoxylation with DTBP 3
3. Reactions Yielding Minor Dialkylation Products 4
4. Reaction Optimization for direct Friedel–Crafts Alkylation of Anisolic Compounds 5
5. Dual Brønsted/Lewis acid-Catalyzed Friedel–Crafts *tert*-Alkylation 6
i) General Procedure A: Alkylations with di-tert-butylperoxide (DTBP) 6
ii) General Procedure B: Alkylations with tertiary alcohols 7
iii) General Procedure C: Alkylations with 1-adamantanol in chlorobenzene 7
iv) Product Characterization 7
6. Mechanistic Studies 39
i) Kinetic Experiments 39
ii) Michael acceptors used to probe formation of radical intermediates 45
iii) Computational Details 46
7. X-Ray Data 52
8. References 59
9. NMR Spectra 61

1. General Information

i) Solvents and reagents

Commercial reagents were purchased from MilliporeSigma, Acros Organics, Chem-Impex, TCI, Oakwood, and Alfa Aesar, and used without additional purification. Solvents were purchased from Fisher Scientific, Acros Organics, Alfa Aesar, and Sigma Aldrich. Tetrahydrofuran (THF), diethyl ether (Et_2O), acetonitrile (MeCN), dichloromethane (CH_2Cl_2), benzene, 1,4-dioxane, and triethylamine (Et_3N) were sparged with argon and dried by passing through alumina columns using argon in a Glass Contour (Pure Process Technology) solvent purification system. Dimethylformamide (DMF), dimethyl sulfoxide (DMSO), and dichloroethane (DCE) were purchased in Sure/Seal or AcroSeal bottling and dispensed under N_2. Deuterated solvents were obtained from Cambridge Isotope Laboratories, Inc. or MilliporeSigma.

ii) Reaction setup, progress monitoring, and product purification

In general, the catalytic reactions are not air- or moisture-sensitive; however, the iron salts are hygroscopic and quickly changes color when being weighed and added to the reaction vessel. This influences how much metal catalyst is being added because their molecular weights increase on hydration. For consistency and rigor, the iron salts were weighed and added to vials inside a nitrogen-filled glovebox. All other reagents, including the solvent were added outside the glovebox under open air. Reaction progresses were monitored using thin-layer chromatography (TLC) on EMD Silica Gel 60 F254 or Macherey–Nagel SIL HD (60 Å mean pore size, 0.75 mL/g specific pore volume, 5–17 µm particle size, with fluorescent indicator) silica gel plates. Visualization of the developed plates was performed under UV light (254 nm). Purification and isolation of products were performed via silica gel chromatography (both column and preparative thin-layer chromatography). Organic solutions were concentrated under reduced pressure on IKA® temperature-controlled rotary evaporator equipped with an ethylene glycol/water condenser.

iii) Analytical instrumentation

Melting points were measured with the MEL-TEMP melting point apparatus.

Proton nuclear magnetic resonance (^1H NMR) spectra, carbon nuclear magnetic resonance (^{13}C NMR) spectra and fluorine nuclear magnetic resonance (^{19}F NMR) spectra were recorded on Bruker Avance NEO 400 (not ^1H decoupled) or Bruker Avance 600 MHz spectrometers (^1H decoupled). Chemical shifts (δ) are reported in ppm relative to the residual solvent signal (δ 7.26 for ^1H NMR, δ 77.16 for ^{13}C NMR in $CDCl_3$).[1] Data for ^1H NMR spectroscopy are reported as

follows: chemical shift (δ ppm), multiplicity (s = singlet, d = doublet, t = triplet, q = quartet, m = multiplet, br = broad, dd = doublet of doublets, dt = doublet of triplets), coupling constant (Hz), integration. Data for ^{13}C and ^{19}F NMR spectroscopy are reported in terms of chemical shift (δ ppm).

IR spectroscopic data were recorded on a NICOLET 6700 FT-IR spectrophotometer using a diamond attenuated total reflectance (ATR) accessory. Samples are loaded onto the diamond surface either neat or as a solution in organic solvent and the data acquired after the solvent had evaporated.

High resolution accurate mass (ESI) spectral data were obtained from the Analytical Chemistry Instrumentation Facility at the University of California, Riverside, on an Agilent 6545 Q-TOF LC/MS instrument (supported by NSF grant CHE-1828782). High resolution accurate mass (EI) spectral data were obtained from the Mass Spectrometry Facility at the University of California, Irvine, on a ThermoFinnegan TraceMS+ GC EI/CI instrument.

X-ray diffraction data were collected on a Bruker-AXS Apex II diffractometer with an Apex II CCD detector using Mo K_α radiation (λ = 0.71073 Å) from a fine-focus sealed tube source. CYLview and ORTEP3 were used for graphic rendering.[2,3]

2. Selected Reaction Optimization Data for Arene *tert*-Butoxylation with DTBP

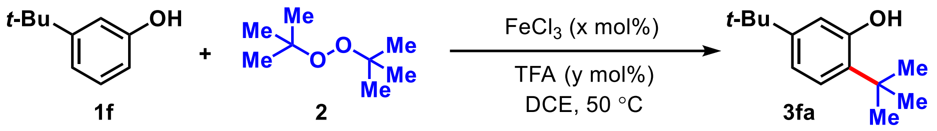

Entry	$FeCl_3$ (*x* mol%)	TFA (*y* mol%)	NMR Yield (%)
1	0	75	< 2
2	10	0	42
3	10	25	71
4	10	50	77
5	10	75	99
6	10	100	93
7	10	125	88
8	10	200	75

3. Reactions Yielding Minor Dialkylation Products

In general, monoalkylation of phenolic and anisolic compounds occurs in a site-selective fashion. In the cases with phenolic substrates (reported in Scheme 5) noted below, minor dialkylation products were isolated and characterized:

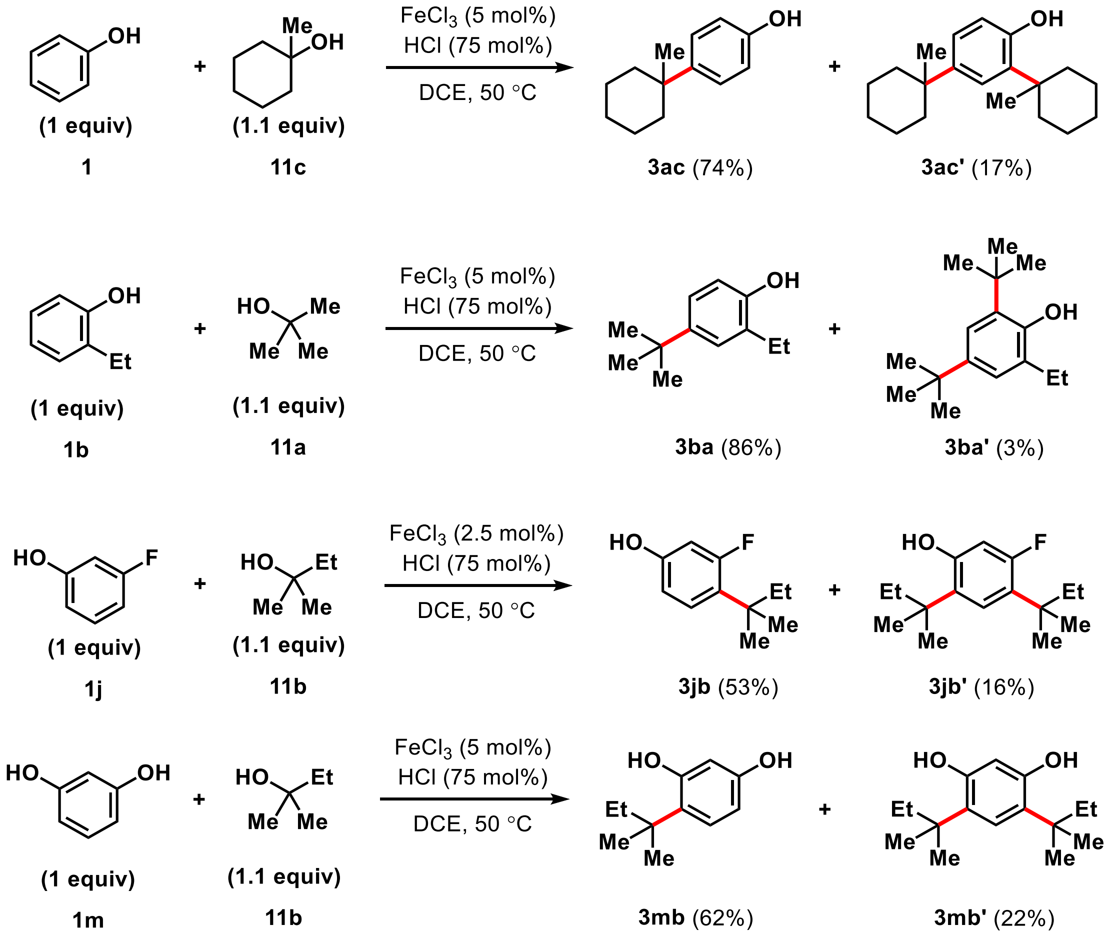

4. Reaction Optimization for direct Friedel–Crafts Alkylation of Anisolic Compounds

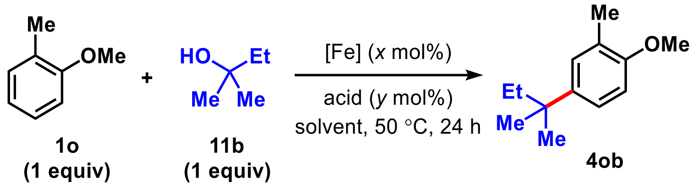

1o (1 equiv) + **11b** (1 equiv) → **4ob**

	[Fe]	*x*	acid	*y*	solvent	% yield[b]
1	$FeBr_3$	30	$HBr_{(aq)}$	15	DCE	68 (33)[c]
2	$FeCl_3$	30	$HCl_{(aq)}$	15	DCE	47
3	$FeBr_3$	30	$HBr_{(aq)}$	75	DCE	44
4	$FeBr_3$	30	$HBr_{(aq)}$	15	DCE	31
5	$FeBr_3$	30	$HBr_{(aq)}$	0	DCE	66
6	$FeBr_3$	30	$HBr_{(aq)}$	15	*i*-PrOH	0
7	$FeBr_3$	30	$HBr_{(aq)}$	15	HFIP	6
8	$FeBr_3$	30	$HBr_{(aq)}$	15	ClC_6H_5	63
9	$FeBr_3$	30	$HBr_{(aq)}$	15	PhMe	25
10	—	—	$HBr_{(aq)}$	100	DCE	0
11	—	—	$AlCl_3$	100	DCE	—[d]

[a] Conditions: All reactions performed on 0.2 mmol scale, anisole (1 equiv), alcohol (1 equiv), 0.2 M, 50 °C, 24 h. [b] Determined by NMR analysis of the crude reaction mixture using 1,3,5-trimethoxybenzene as the internal standard. [c] 20 °C. [d] Low conversion to a mixture of products.

Using 2-methylanisole (**1o**) for optimization studies, it was discovered that the optimal condition involved $FeBr_3$ and HBr in DCE solvent, which afforded the product (**4ob**) in 68% NMR yield (entry 1). While the use of $FeCl_3$ is optimal with phenolic substrates, it decreased the conversion to 47% (entry 2), and $FeBr_2$ proved even less effective (31%, entry 4). Adding more HBr co-catalyst reduced product formation to 44% (entry 3). The reaction still proceeded to 66% conversion when the acid additive was absent (entry 5). Chlorobenzene was the only other effective solvent (63%, entries 6–9). Finally, the reaction was run with only the Brønsted (entry

10) or $AlCl_3$ (entry 11). Both Friedel–Crafts reactions failed, with $AlCl_3$ inducing low conversion to a complex mixture.

In this example, omitting HBr from the reaction conditions only resulted in a 2% decrease in conversion to product (entry 5 vs entry 1). This reaction is particularly well-behaved and there are other examples where the addition of HBr had noticeably positive effects. For example:

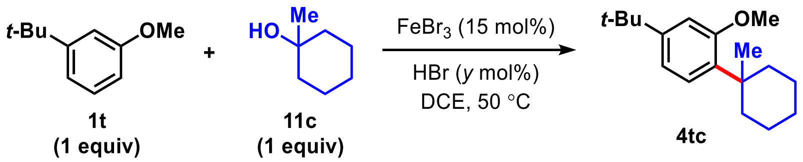

y	time (h)	NMR conv. (%)
0.15	2.5	4
0.15	48	61
0.75	48	>99

The addition of catalytic Brønsted acid for anisolic substrates generally improves conversions and isolated yields. We do believe that the combination of FeX_3 plus a strong Bronsted acid will always result in activation of the Bronsted acid. This will affect the equilibrium between the tertiary alcohol and its activated form. There will be arene substrates that are better behaved and capable of reacting with the minor reactive species, and other more challenging substrates that are less capable of doing so and will require Lewis acid-assisted Bronsted acidity enhancement. The optimization studies described here was used to determine a starting point for our substrate scope studies in Scheme 6. We have optimized conditions for several substrates, some of which require 10 mol% HBr, some 15 mol% HBr, and some 75 mol% HCl. The important finding from these studies is that even though 2-methylanisole may not require a strong acid, the use of it is not detrimental, and as such would make a good starting point for *tert*-alkylation conditions to test.

5. Dual Brønsted/Lewis acid-Catalyzed Friedel–Crafts *tert*-Alkylation

i) General Procedure A: Alkylations with di-tert-butylperoxide (DTBP)

A one-dram vial equipped with a stirring bar was sequentially added $FeCl_3$ (0.02–0.06 mmol, 10–30 mol%),* arene derivative (0.2 mmol, 1 equiv), DCE (0.8 mL, 0.25 M), DTBP (37 µL, 0.2 mmol,

* $FeCl_3$ is hygroscopic and quickly changes to an orange color as it becomes hydrated. As such, we weigh $FeCl_3$ into a vial in a nitrogen-filled glovebox to be rigorous with the weight(s) that we report. However, the reactions do not need to be anhydrous and the use of $FeCl_3$ that has turned orange will not have a significant effect beyond an underestimation of the Fe actually added to the reaction mixture.

1 equiv), and TFA (11.5 µL, 0.15 mmol, 75 mol%). The reaction mixture was heated at 50 °C for 2 h, at which time the solution was filtered through a 5” pipette plug of silica gel (approximately half-filled) and eluted with hexanes/EtOAc (1:1) or hexanes/Et_2O (1:1). The solution was concentrated *in vacuo* and purified via silica gel chromatography to obtain the alkylation product.

ii) General Procedure B: Alkylations with tertiary alcohols

A one-dram vial equipped with a stirring bar was sequentially added iron (1–100 mol%),† arene derivative (0.2 mmol, 1 equiv), DCE (0.8 mL, 0.25 M), tertiary alcohol (0.22 mmol, 1.1 equiv), and conc. $HCl_{(aq)}$/$HBr_{(aq)}$ (10–75 mol%). The reaction mixture was heated at 50 °C for 24 h, at which time the solution was filtered through a 5” pipette plug of silica gel (approximately half-filled) and eluted with hexanes/EtOAc (1:1). The solution was concentrated *in vacuo* and purified via silica gel chromatography to obtain the alkylation product.

iii) General Procedure C: Alkylations with 1-adamantanol in chlorobenzene

A one-dram vial equipped with a stirring bar was sequentially added iron (0.01 mmol, 5 mol%), arene derivative (0.2 mmol, 1 equiv), chlorobenzene (0.8 mL, 0.25 M), 1-adamantanol (0.22 mmol, 1.1 equiv), and conc. $HCl_{(aq)}$ (0.15 mmol, 75 mol%). The reaction mixture was heated at 100 °C for 24 h, at which time the solution was filtered through a 5” pipette plug of silica gel (approximately half-filled) and eluted with hexanes/EtOAc (1:1). The solution was concentrated *in vacuo* and purified via silica gel chromatography to obtain the alkylation product.

iv) Product Characterization

2,4-Di-*tert*-butylphenol (3ba)

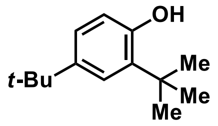

Prepared using General Procedure A with 4-*tert*-butylphenol (34.0 mg, 0.200 mmol, 1 equiv), $FeCl_3$ (6.5 mg, 0.04 mmol, 0.02 equiv), DCE (0.8 mL, 0.25 M), DTBP (37 µL, 0.20 mmol, 1 equiv), and TFA (11.5 µL, 0.15 mmol, 0.75 equiv) for 18 h. Purification by preparative TLC (eluting with 19:1 hexanes/EtOAc) afforded **3ba** (9.1 mg, 22%) as a light-orange solid. ^1H NMR ($CDCl_3$, 500 MHz): δ 7.30 (d, J = 2.4 Hz, 1H), 7.08 (dd, J = 8.2, 2.4 Hz, 1H), 6.60 (d, J = 8.2 Hz, 1H), 4.63 (s, 1H), 1.42 (s, 9H), 1.29 (s, 9H). HRMS (ESI–): m/z $[M–H]^–$ calculated for $C_{14}H_{21}O$: 205.1598; found: 205.1608. The spectral data recorded are consistent with those previously reported.[4]

† $FeCl_3$ is hygroscopic and quickly changes to an orange color as it becomes hydrated. As such, we weigh $FeCl_3$ into a vial in a nitrogen-filled glovebox to be rigorous with the weight(s) that we report. However, the reactions do not need to be anhydrous and the use of $FeCl_3$ that has turned orange will not have a significant effect beyond an underestimation of the Fe actually added to the reaction mixture.

2-*tert*-Butyl-4-chlorophenol (3ca)

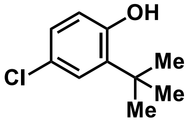

Prepared using General Procedure A with 4-chlorophenol (25.7 mg, 0.200 mmol, 1 equiv), $FeCl_3$ (32.4 mg, 0.20 mmol, 1 equiv), DCE (0.8 mL, 0.25 M), DTBP (37 µL, 0.20 mmol, 1 equiv), and TFA (11.5 µL, 0.15 mmol, 0.75 equiv) for 48 h. Purified via preparative TLC (eluting with 19:1 hexanes/EtOAc) afforded **3ca** (15.1 mg, 41%) as colorless oil. R_f: 0.23 (19:1 hexanes/EtOAc). ^{1}H NMR ($CDCl_3$, 600 MHz): δ 7.21 (d, *J* = 3.0 Hz, 1H), 7.02 (dd, *J* = 8.4, 2.4, 1H), 6.60 (d, *J* = 8.4 Hz, 1H), 4.77 (br s, 1H), 1.38 (s, 9H); ^{13}C NMR ($CDCl_3$, 101 MHz): δ 152.9, 138.2, 127.5, 126.7, 125.58, 117.7, 34.9, 29.5. HRMS (ESI–): m/z $[M–H]^–$ calculated for $C_{10}H_{12}ClO$: 183.0582; found: 183.0590. The spectral data recorded are consistent with those previously reported.[5]

2-*tert*-Butyl-4-fluorophenol (3da)

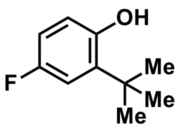

Prepared using General Procedure A with 4-fluorophenol (22.4 mg, 0.200 mmol, 1 equiv), $FeCl_3$ (32.4 mg, 0.20 mmol, 1 equiv), DCE (0.8 mL, 0.25 M), DTBP (37 µL, 0.20 mmol, 1 equiv), and TFA (11.5 µL, 0.15 mmol, 0.75 equiv) for 48 h. Purified via preparative TLC (eluting with 19:1 hexanes/EtOAc) afforded **3da** (21.5 mg, 64%) as yellow oil. R_f: 0.43 (19.1 hexanes/EtOAc). ^{1}H NMR ($CDCl_3$, 600 MHz): δ 6.97 (dd, *J* = 10.8, 3.0 Hz, 1H), 6.75 (td, *J* = 8.4, 3.0 Hz, 1H), 6.59 (dd, *J* = 8.4, 3.0 Hz, 1H), 4.68 (br s, 1H), 1.39 (s, 9H). The spectral data recorded are consistent with those previously reported.[5]

2-*tert*-Butyl-5-ethylphenol (3ea)

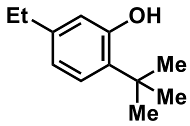

Prepared using General Procedure A with 3-ethylphenol (24.8 µL, 0.200 mmol, 1 equiv), $FeCl_3$ (3.2 mg, 0.020 mmol, 0.1 equiv), DCE (0.8 mL, 0.25 M), DTBP (37 µL, 0.20 mmol, 1 equiv), and TFA (11.5 µL, 0.15 mmol, 0.75 equiv). Purification by preparative TLC (eluting with 19:1 hexanes/EtOAc) afforded **3ea** (20.7 mg, 58%) as a yellow oil. R_f: 0.36 (19:1 hexanes/EtOAc). ^{1}H NMR ($CDCl_3$, 500 MHz): δ 7.12 (d, *J* = 2.3 Hz, 1H), 6.93 (dd, *J* = 7.9, 2.2 Hz, 1H), 6.61 (d, *J* = 7.9 Hz, 1H), 4.69 (br s, 1H), 2.60 (q, *J* = 7.6 Hz, 3H), 1.44 (s, 9H), 1.24 (t, *J* = 7.6 Hz, 3H); ^{13}C NMR ($CDCl_3$, 126 MHz): δ 152.2, 136.2, 135.9, 126.8, 126.0, 116.5, 34.6, 29.8, 28.4, 16.1. IR (ATR): 3515, 2960, 1651, 1461, 1362, 728 $cm^{–1}$. HRMS (ESI–): *m/z* $[M–H]^–$ calculated for $C_{12}H_{17}O$: 177.1285; found: 177.1293.

2,5-Di-*tert*-butylphenol (3fa)

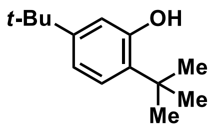

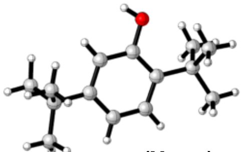

(X-ray)

Prepared using General Procedure A with 3-*tert*-butylphenol (30.0 mg, 0.200 mmol, 1 equiv), $FeCl_3$ (3.2 mg, 0.020 mmol, 0.1 equiv), DCE (0.8 mL, 0.25 M), DTBP (37 µL, 0.2 mmol, 1 equiv), and TFA (11.5 µL, 0.15 mmol, 0.75 equiv). Purification by preparative TLC (eluting with 19:1 hexanes/EtOAc) afforded **3fa** (37.9 mg, 92%) as a light orange-white solid. R_f: 0.44 (19:1 hexanes/EtOAc). M.p. 103–106 °C. ^1H NMR ($CDCl_3$, 600 MHz): δ 7.23 (d, J = 8.2 Hz, 1H), 6.92 (dd, J = 8.1, 2.0 Hz, 1H), 6.71 (d, J = 2.0 Hz, 1H), 4.80 (br s, 1H), 1.44 (s, 9H), 1.32 (s, 9H); ^{13}C NMR ($CDCl_3$, 151 MHz): δ 153.8, 150.5, 133.1, 126.75, 117.5, 114.0, 34.2, 31.4, 29.8. IR (ATR): 3509, 2954, 1611, 1360, 700 cm^{-1}. HRMS (ESI–): m/z $[M–H]^-$ calculated for $C_{14}H_{21}O$: 205.1598; found: 205.1608. The site-selectivity is unambiguously confirmed by single crystal X-ray diffraction.

2-*tert*-Butyl-5-phenylphenol (3ga)

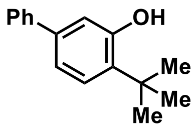

Prepared using General Procedure A with 3-phenylphenol (34.0 mg, 0.200 mmol, 1 equiv), $FeCl_3$ (3.2 mg, 0.020 mmol, 0.1 equiv), DCE (0.8 mL, 0.25 M), DTBP (37 µL, 0.20 mmol, 1 equiv), and TFA (11.5 µL, 0.15 mmol, 0.75 equiv). Purification by preparative TLC (eluting with 19:1 hexanes/EtOAc) afforded **3ga** (33.0 mg, 73%) as a red solid. R_f: 0.26 (19:1 hexanes/EtOAc). M.p. 74–77 °C. ^1H NMR ($CDCl_3$, 500 MHz): δ 7.62–7.53 (m, 2H), 7.44 (t, J = 7.7 Hz, 2H), 7.36 (dd, J = 7.8, 3.2 Hz, 2H), 7.14 (dd, J = 8.0, 1.9 Hz, 1H), 6.90 (d, J = 1.9 Hz, 1H), 4.96 (br s, 1H), 1.48 (s, 9H); ^{13}C NMR ($CDCl_3$, 126 MHz): δ 154.5, 140.5, 140.4, 135.4, 128.8, 127.7, 127.4, 127.0, 119.4, 115.3, 34.54, 29.8. IR (ATR): 3531, 2953, 1614, 1447, 1360, 700 cm^{-1}. HRMS (ESI–): m/z $[M–H]^-$ calculated for $C_{16}H_{17}O$: 225.1285; found: 225.1285.

2-*tert*-Butyl-5-chlorophenol (3ha)

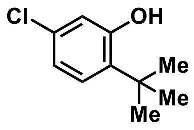

Prepared using General Procedure A with 3-chlorophenol (21.1 µL, 0.200 mmol, 1 equiv), $FeCl_3$ (3.2 mg, 0.020 mmol, 0.1 equiv), DCE (0.8 mL, 0.25 M), DTBP (37 µL, 0.20 mmol, 1 equiv), and TFA (11.5 µL, 0.15 mmol, 0.75 equiv). Purification by preparative TLC (3 × elutions with 49:1 hexanes/EtOAc) afforded **3ha** (15.1 mg, 39%) as a yellow oil. R_f: 0.49 (9:1 hexanes/EtOAc). ^1H NMR ($CDCl_3$, 500 MHz): δ 7.17 (d, J =

8.4 Hz, 1H), 6.84 (dd, *J* = 8.5, 2.2 Hz, 1H), 6.69 (d, *J* = 2.2 Hz, 1H), 4.94 (br s, 1H), 1.38 (s, 9H); ^{13}C NMR ($CDCl_3$, 126 MHz): δ 154.9, 135.0, 131.9, 128.2, 120.7, 116.7, 34.5, 29.6. IR (ATR): 3547, 2958, 1653, 1465, 1363, 704 cm^{-1}. HRMS (ESI–): *m/z* [M–H]$^-$ calculated for $C_{10}H_{12}ClO$: 183.0582; found: 183.0584.

2,4-Di-*tert*-butyl-5-methoxyphenol (3iaa)

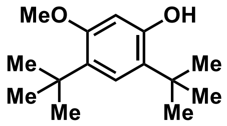

Prepared using General Procedure A with 3-methoxyphenol (21.7 µL, 0.200 mmol, 1 equiv), $FeCl_3$ (3.2 mg, 0.020 mmol, 0.1 equiv), DCE (0.8 mL, 0.25 M), DTBP (37 µL, 0.20 mmol, 1 equiv),‡ and TFA (11.5 µL, 0.15 mmol, 0.75 equiv). Purification by preparative TLC (3 elutions with 4:1 hexanes/EtOAc) afforded **3iaa** (42.5 mg, 90%) as an orange solid. R_f: 0.39 (7:3 hexanes/EtOAc). M.p. 95–98 °C. ^{1}H NMR ($CDCl_3$, 500 MHz): δ 7.15 (s, 1H), 6.26 (s, 1H), 4.61 (br s, 1H), 3.78 (s, 3H), 1.39 (s, 9H), 1.34 (s, 9H); ^{13}C NMR ($CDCl_3$, 126 MHz): δ 157.2, 152.7, 129.7, 126.6, 125.4, 101.5, 55.2, 34.6, 34.2, 30.2. IR (ATR): 3386, 2948, 1598, 1443, 1358, 724 cm^{-1}. HRMS (ESI–): *m/z* [M–H]$^-$ calculated for $C_{15}H_{23}O_2$: 235.1704; found: 235.1711.

2,4-Di-*tert*-butyl-5-fluorophenol (3jaa)

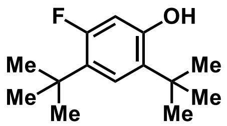

Prepared using General Procedure A with 3-fluorophenol (21.7 µL, 0.200 mmol, 1 equiv), $FeCl_3$ (3.2 mg, 0.020 mmol, 0.1 equiv), DCE (0.8 mL, 0.25 M), DTBP (37 µL, 0.20 mmol, 1 equiv),§ and TFA (11.5 µL, 0.15 mmol, 0.75 equiv). Without filtering through a silica plug, purification by preparative TLC (eluting with 9:1 hexanes/EtOAc) afforded **3jaa** (5.8 mg, 13%) as a colorless oil. R_f: 0.44 (9:1 hexanes/EtOAc). ^{1}H NMR ($CDCl_3$, 500 MHz): δ 7.15 (d, *J* = 9.7 Hz, 1H), 6.38 (d, *J* = 12.8 Hz, 1H), 4.75 (br s, 1H), 1.39 (s, 9H), 1.34 (s, 9H); ^{13}C NMR ($CDCl_3$, 126 MHz): δ 160.0 (d, *J* = 252 Hz), 152.9 (d, *J* = 10.3 Hz), 130.9, 128.1, 125.5 (d, *J* = 7.4 Hz), 105.0 (d, *J* = 27.0 Hz), 34.54, 34.0 (d, *J* = 3.0 Hz), 30.3 (d, *J* = 3.3 Hz), 29.9; ^{19}F NMR ($CDCl_3$, 376 MHz): 114.2 (t, *J* = 11.1 Hz). IR (ATR): 3544, 2957, 1618, 1469, 1363, 717 cm^{-1}. HRMS (ESI–): *m/z* [M–H]$^-$ calculated for $C_{14}H_{20}FO$: 223.1504;

‡ Even with 0.5 equiv DTBP, the dialkylation product was isolated as the major product along with unreacted starting material.

§ Even with 0.5 equiv DTBP, the dialkylation product was isolated as the major product along with unreacted starting material.

found: 223.1512. Monoalkylation products were isolated in low yields: 2-*tert*-butyl-5-fluorophenol (1.7 mg, 5%) and 4-*tert*-butyl-5-fluorophenol (3.2 mg, 10%).

2-Benzyl-4-*tert*-butylphenol (3ka)

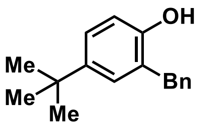

Prepared using General Procedure A with 2-benzylphenol (36.8 mg, 0.200 mmol, 1 equiv), $FeCl_3$ (6.5 mg, 0.040 mmol, 0.2 equiv), DCE (0.8 mL, 0.25 M), DTBP (37 µL, 0.20 mmol, 1 equiv), and TFA (11.5 µL, 0.15 mmol, 0.75 equiv) for 18 h. Purification by preparative TLC (eluting with 19:1 hexanes/EtOAc) afforded **3ka** (21.1 mg, 44%) as a yellow oil. R_f: 0.42 (19:1 hexanes/EtOAc). ^1H NMR ($CDCl_3$, 500 MHz): δ 7.30 (t, *J* = 7.6 Hz, 2H), 7.23 (d, *J* = 7.4 Hz, 2H), 7.20 (d, *J* = 7.3 Hz, 2H), 7.16–7.10 (m, 2H), 6.75–6.68 (m, 1H), 4.52 (br s, 1H), 4.00 (s, 1H), 1.28 (s, 9H); ^{13}C NMR ($CDCl_3$, 126 MHz): δ 151.6, 143.8, 140.2, 128.74, 128.2, 126.4, 126.2, 124.7, 115.4, 37.0, 34.2, 31.7. IR (ATR): 3525, 3027, 2960, 1602, 1453, 1363, 728 cm^{-1}. HRMS (ESI–): *m/z* $[M–H]^-$ calculated for $C_{17}H_{19}O$: 239.1441; found: 239.1447.

4-*tert*-Butyl-2-fluorophenol (3la)

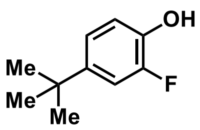

Prepared using General Procedure A with 2-fluorophenol (18.0 µL, 0.200 mmol, 1 equiv), $FeCl_3$ (32.4 mg, 0.20 mmol, 1 equiv), DCE (0.8 mL, 0.25 M), DTBP (37 µL, 0.20 mmol, 1 equiv), and TFA (11.5 µL, 0.15 mmol, 0.75 equiv) for 48 h. Purified via preparative TLC (eluting with 9:1 hexanes/EtOAc) afforded **3la** (8.7 mg, 26%) as a light-yellow oil. R_f: 0.56 (9:1 hexanes/EtOAc). ^1H NMR ($CDCl_3$, 500 MHz): δ 7.12 (dd, *J* = 12.6, 2.4 Hz, 1H), 7.06 (d, *J* = 7.2 Hz, 1H), 6.95 (t, *J* = 9.6, 1H), 4.98 (br s, 1H), 1.31 (s, 9H); ^{19}F NMR ($CDCl_3$, 564 MHz): δ –141.2 (s). The spectral data recorded are consistent with those previously reported.[6]

4-(*tert*-Butyl)-1-methoxybenzene (4ma) Prepared using General Procedure A with anisole (21.6 mg, 0.200 mmol, 1 equiv), $FeCl_3$ (9.7 mg, 0.060 mmol, 0.3 equiv), DCE (0.8 mL, 0.25 M), DTBP (37 µL, 0.20 mmol, 1 equiv), and conc. $HCl_{(aq)}$ (14 µL, 0.15 mmol, 0.75 equiv). Purification by preparative TLC (eluting with 19:1 hexanes/EtOAc) afforded **4ma** (24.0 mg, 73%) as a colorless oil. R_f: 0.38 (19:1 hexanes/EtOAc). ^1H NMR ($CDCl_3$, 600 MHz): δ 7.32 (d, *J* = 8.7 Hz, 2H), 6.86 (d, *J* = 8.7 Hz, 2H), 3.80 (s, 3H),

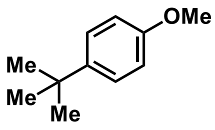

1.31 (s, 9H); ^{13}C NMR ($CDCl_3$, 101 MHz): δ 157.4, 143.5, 126.4, 113.5, 55.4, 34.2, 31.7. The spectral data recorded are consistent with those previously reported.[7]

1-(3-Bromopropoxy)-2,4-di-*tert*-butylbenzene (4naa)

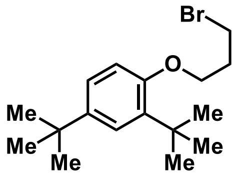

Prepared using General Procedure A with 3-phenoxypropyl bromide (43.0 mg, 0.200 mmol, 1 equiv), $FeCl_3$ (9.7 mg, 0.060 mmol, 0.3 equiv), DCE (0.8 mL, 0.25 M), DTBP (37 µL, 0.20 mmol, 1 equiv), and conc. $HCl_{(aq)}$ (14 µL, 0.15 mmol, 0.75 equiv). Purification by preparative TLC (eluting with hexanes) afforded **4naa** (31.6 mg, 48%) as a pale-yellow oil. R_f: 0.66 (hexanes). ^{1}H NMR ($CDCl_3$, 400 MHz): δ 7.34 (d, J = 2.3 Hz, 1H), 7.19 (dd, J = 8.4, 2.3 Hz, 1H), 6.82 (d, J = 8.5 Hz, 1H), 4.13 (t, J = 5.7 Hz, 2H), 3.66 (t, J = 6.5 Hz, 2H), 2.38 (p, J = 6.1 Hz, 2H), 1.40 (s, 9H), 1.31 (s, 9H); ^{13}C NMR ($CDCl_3$, 101 MHz): δ 155.2, 142.9, 137.2, 124.2, 123.5, 111.3, 65.2, 35.2, 34.4, 32.8, 31.7, 30.6, 30.1. IR (ATR): 2955, 2867, 1498, 1361, 1233, 1201, 1094, 1033, 810, 669 cm^{-1}. HRMS (CI+) *m*/*z* calculated for $C_{17}H_{27}BrO$ $[M]^+$: 326.1245; found: 326.1235.

4-(*tert*-Butyl)-1-methoxy-2-methylbenzene (4oa)

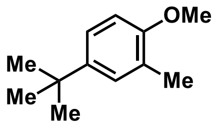

Prepared using General Procedure A with 2-methylanisole (24.4 mg, 0.200 mmol, 1 equiv), $FeCl_3$ (9.7 mg, 0.060 mmol, 0.3 equiv), DCE (0.8 mL, 0.25 M), DTBP (37 µL, 0.20 mmol, 1 equiv), and conc. $HCl_{(aq)}$ (14 µL, 0.15 mmol, 0.75 equiv). Purification by preparative TLC (eluting with 19:1 hexanes/EtOAc) afforded **4oa** (29.5 mg, 83%) as a colorless oil. R_f: 0.62 (19:1 hexanes/EtOAc). ^{1}H NMR ($CDCl_3$, 600 MHz): δ 7.21–7.16 (m, 2H), 6.80–6.76 (m, 1H), 3.83 (s, 3H), 2.24 (s, 3H), 1.31 (s, 9H); ^{13}C NMR ($CDCl_3$, 101 MHz): δ 155.6, 143.0, 128.0, 126.0, 123.3, 109.5, 55.4, 34.1, 31.7, 16.6. The spectral data recorded are consistent with those previously reported.[8]

4-(*tert*-Butyl)-1-methoxy-2-ethylbenzene (4pa)

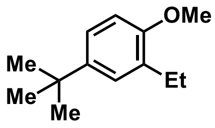

Prepared using General Procedure A with 2-ethyl-methoxybenzene (27.2 mg, 0.200 mmol, 1 equiv), $FeCl_3$ (9.7 mg, 0.060 mmol, 0.3 equiv), DCE (0.8 mL, 0.25 M), DTBP (37 µL, 0.20 mmol, 1 equiv), and conc. $HCl_{(aq)}$ (14 µL, 0.15 mmol, 0.75 equiv). Purification by preparative TLC (eluting with 19:1 hexanes/EtOAc) afforded **4pa** (33.8 mg, 88%) as a light-yellow oil. R_f: 0.63 (19:1 hexanes/ EtOAc). ^{1}H NMR ($CDCl_3$, 600 MHz): δ 7.23–7.14 (m, 2H), 6.82–6.76 (m, 1H), 3.82 (s, 3H), 2.65 (q, J = 7.5 Hz, 2H), 1.32 (s,

9H), 1.21 (t, *J* = 7.5 Hz, 3H); ^{13}C NMR ($CDCl_3$, 101 MHz): δ 155.3, 143.1, 132.0, 126.4, 123.3, 109.8, 55.4, 34.2, 31.7, 23.8, 14.5. IR (ATR): 2960, 2833, 1503, 1462, 1362, 1244, 1177, 1145, 1035, 889, 808, 639 cm^{-1}. HRMS (ESI+): *m/z* $[M+H]^+$ calculated for $C_{13}H_{21}O$: 193.1587; found: 193.1575.

1-(5-(*tert*-Butyl)-2-methoxyphenyl)propan-2-ol (4qa)

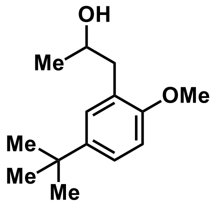

Prepared using General Procedure A with 1-(2-methoxyphenyl)propan-2-ol (33.2 mg, 0.200 mmol, 1 equiv), $FeCl_3$ (9.7 mg, 0.060 mmol, 0.3 equiv), DCE (0.8 mL, 0.25 M), DTBP (37 µL, 0.20 mmol, 1 equiv), and conc. $HCl_{(aq)}$ (14 µL, 0.15 mmol, 0.75 equiv). Purification by preparative TLC (eluting with 19:1 hexanes/EtOAc) afforded **4qa** (20.0 mg, 45%) as a colorless oil. R_f: 0.34 (19:1 hexanes/ EtOAc). ^{1}H NMR ($CDCl_3$, 400 MHz): δ 7.24 (dd, *J* = 8.5, 2.4 Hz, 1H), 7.18 (d, *J* = 2.3 Hz, 1H), 6.79 (d, *J* = 8.5 Hz, 1H), 4.33 (q, *J* = 6.7 Hz, 1H), 3.81 (s, 3H), 3.09 (dd, *J* = 13.4, 6.9 Hz, 1H), 3.00 (dd, *J* = 13.4, 7.1 Hz, 1H), 1.50 (d, *J* = 6.5 Hz, 3H), 1.30 (s, 9H); ^{13}C NMR ($CDCl_3$, 101 MHz): δ 155.5, 143.1, 128.9, 125.9, 124.6, 109.8, 57.9, 55.4, 42.1, 34.1, 31.7, 25.0. IR (ATR): 2956, 2835, 1503, 1464, 1248, 1143, 1034, 810, 617, 530 cm^{-1}. HRMS (ESI+): *m/z* $[M–OH]^+$ calculated for $C_{14}H_{21}O$: 205.1587; found: 205.1578.

2-Bromo-4-(*tert*-butyl)-1-methoxybenzene (4ra)

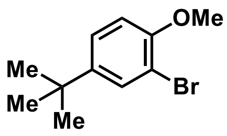

Prepared using General Procedure A with 2-bromo-methoxybenzene (37.4 mg, 0.200 mmol, 1 equiv), $FeCl_3$ (9.7 mg, 0.060 mmol, 0.3 equiv), DCE (0.8 mL, 0.25 M), DTBP (37 µL, 0.20 mmol, 1 equiv), and conc. $HCl_{(aq)}$ (14 µL, 0.15 mmol, 0.75 equiv). Purification by preparative TLC (eluting with 19:1 hexanes/EtOAc) afforded **4ra** (25.8 mg, 53%) as a pale-yellow oil. R_f: 0.55 (19:1 hexanes/ EtOAc). ^{1}H NMR ($CDCl_3$, 600 MHz): δ 7.54 (d, *J* = 2.2 Hz, 1H), 7.27 (dd, *J* = 8.6, 2.2 Hz, 1H), 6.84 (d, *J* = 8.6 Hz, 1H), 3.88 (s, 3H), 1.29 (s, 9H); ^{13}C NMR ($CDCl_3$, 101 MHz): δ 153.7, 145.2, 130.6, 125.4, 111.7, 111.4, 56.4, 34.3, 31.5. IR (ATR): 2959, 2838, 1602, 1501, 1481, 1461, 1439, 1393, 1362, 1289, 1260, 1203, 1182, 1162, 1118, 1054, 1020, 909, 880, 862, 808, 748, 733, 719, 692 cm^{-1}. HRMS (CI+) *m/z* calculated for $C_{11}H_{15}BrO$ $[M]^+$: 242.0306; found: 242.0301.

5-(*tert*-Butyl)benzo[*d*][1,3]dioxole (4sa)

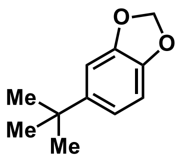

Prepared using General Procedure A with 1,3-benzodioxole (24.4 mg, 0.200 mmol, 1 equiv), $FeCl_3$ (9.7 mg, 0.060 mmol, 0.3 equiv), DCE (0.8 mL, 0.25 M), DTBP (37 µL, 0.20 mmol, 1 equiv), and conc. $HCl_{(aq)}$ (14 µL, 0.15 mmol, 0.75 equiv). Purification by preparative TLC (eluting with 19:1 hexanes/EtOAc) afforded **4sa** (17.8 mg, 50%) as a colorless oil. R_f: 0.46 (19:1 hexanes/EtOAc). ^{1}H NMR ($CDCl_3$, 400 MHz): δ 6.90 (d, *J* = 1.8 Hz, 1H), 6.83 (dd, *J* = 8.1, 1.8 Hz, 1H), 6.74 (d, *J* = 8.2 Hz, 1H), 5.92 (s, 2H), 1.28 (s, 9H); ^{13}C NMR ($CDCl_3$, 101 MHz): δ 147.5, 145.6, 145.2, 118.0, 107.8, 106.5, 100.9, 34.8, 31.7. IR (ATR): 2957, 2869, 1507, 1488, 1364, 1255, 1230, 1112, 1041, 939, 909, 859, 807, 640 cm^{-1}. HRMS (ESI+): *m*/*z* $[M+H]^+$ calculated for $C_{11}H_{15}O_2$: 179.1067; found: 179.1056.

1,4-Di-*tert*-Butyl-2-methoxybenzene (4ta)

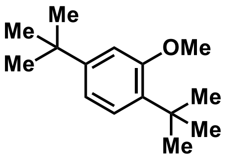

Prepared using General Procedure A with 3-(*tert*-butyl)-1-methoxybenzene (32.8 mg, 0.200 mmol, 1 equiv), $FeCl_3$ (9.7 mg, 0.060 mmol, 0.3 equiv), DCE (0.8 mL, 0.25 M), DTBP (37 µL, 0.20 mmol, 1 equiv), and conc. $HCl_{(aq)}$ (14 µL, 0.15 mmol, 0.75 equiv). Purification by preparative TLC (eluting with 19:1 hexanes/EtOAc) afforded **4ta** (32.2 mg, 73%) as a colorless oil. R_f: 0.77 (19:1 hexanes/EtOAc). ^{1}H NMR ($CDCl_3$,600 MHz): δ 7.22–7.15 (m, 1H), 7.01–6.81 (m, 2H), 3.85 (s, 3H), 1.36 (s, 9H), 1.32 (s, 9H); ^{13}C NMR ($CDCl_3$, 101 MHz): δ 158.4, 150.2, 135.4, 126.2, 117.1, 109.3, 55.1, 34.6, 34.5, 31.5, 30.0. IR (ATR): 2955, 2830, 1611, 1563, 1462, 1399, 1360, 1268, 1228, 1158, 1079, 1039, 912, 853, 818, 734, 658 cm^{-1}. HRMS (ESI+): *m*/*z* $[M+H]^+$ calculated for $C_{15}H_{25}O$: 221.1900; found: 221.1892.

1,4-Di-*tert*-Butyl-2-ethoxybenzene (4ua)

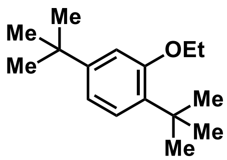

Prepared using General Procedure A with 3-(*tert*-butyl)-1-ethoxybenzene (35.7 mg, 0.200 mmol, 1 equiv), $FeCl_3$ (9.7 mg, 0.060 mmol, 0.3 equiv), DCE (0.8 mL, 0.25 M), DTBP (37 µL, 0.20 mmol, 1 equiv), and conc. $HCl_{(aq)}$ (14 µL, 0.15 mmol, 0.75 equiv). Purification by preparative TLC (eluting with hexanes) afforded **4ua** (31.8 mg, 68%) as a pale-yellow oil. R_f: 0.78 (19:1 hexanes/EtOAc). ^{1}H NMR ($CDCl_3$, 400 MHz): δ 7.19 (d, *J* = 8.6 Hz, 1H), 6.92–6.86 (m, 2H), 4.08 (q, *J* = 6.9 Hz, 2H), 1.47 (t, *J* = 7.0 Hz, 3H),

1.39 (s, 9H), 1.31 (s, 9H); ^{13}C NMR ($CDCl_3$, 101 MHz): δ 157.6, 150.1, 135.3, 126.2, 116.8, 109.8, 63.4, 34.6, 34.6, 31.8, 31.5, 30.0, 15.2. IR (ATR): 3454, 2978, 2858, 1736, 1503, 1369, 1310, 1255, 1159, 1131, 1097, 1071, 958, 836, 779 cm^{-1}. HRMS (ESI+): *m*/*z* $[M+H]^+$ calculated for $C_{16}H_{27}O$: 235.2056; found: 235.2052.

2,4-Di-*tert*-Butyl-1-methoxybenzene (4va)

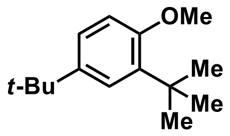

Prepared using General Procedure A with 4-(*tert*-butyl)-1-methoxybenzene (32.8 mg, 0.200 mmol, 1 equiv), $FeCl_3$ (9.7 mg, 0.060 mmol, 0.3 equiv), DCE (0.8 mL, 0.25 M), DTBP (37 µL, 0.20 mmol, 1 equiv), and conc. $HCl_{(aq)}$ (14 µL, 0.15 mmol, 0.75 equiv). Purification by preparative TLC (eluting with 19:1 hexanes/EtOAc) afforded **4va** (33.1 mg, 75%) as a colorless oil. R_f: 0.72 (19:1 hexanes/EtOAc). ^{1}H NMR ($CDCl_3$, 600 MHz): δ 7.32 (br s, 1H), 7.19 (d, *J* = 8.0 Hz, 1H), 6.81 (d, *J* = 8.0 Hz, 1H), 3.82 (s, 3H), 1.38 (s, 9H), 1.31 (s, 9H); ^{13}C NMR ($CDCl_3$, 101 MHz): δ 156.4, 142.6, 137.5, 124.0, 123.4, 111.0, 55.1, 35.2, 34.4, 31.8, 29.9. The spectral data recorded are consistent with those previously reported.[9]

Ethyl 3-(3-(*tert*-butyl)-4-methoxyphenyl)propanoate (4wa)

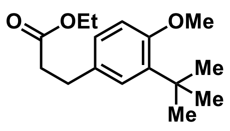

Prepared using General Procedure A with ethyl-3-(4-methoxyphenyl) propanoate (41.7 mg, 0.200 mmol, 1 equiv), $FeCl_3$ (9.7 mg, 0.060 mmol, 0.3 equiv), DCE (0.8 mL, 0.25 M), DTBP (37 µL, 0.20 mmol, 1 equiv), and conc. $HCl_{(aq)}$ (14 µL, 0.15 mmol, 0.75 equiv). Purification by preparative TLC (eluting with 19:1 hexanes/EtOAc) afforded **4wa** (28.6 mg, 54%) as a yellow oil. R_f: 0.42 (19:1 hexanes/ EtOAc). ^{1}H NMR ($CDCl_3$, 400 MHz): δ 7.10 (s, 1H), 7.01 (d, *J* = 8.2 Hz, 1H), 6.80 (d, *J* = 8.2 Hz, 1H), 4.14 (q, *J* = 7.1 Hz, 2H), 3.82 (s, 3H), 3.68 (s, 1H), .89 (t, *J* = 7.6 Hz, 2H), 2.60 (q, *J* = 7.5, 6.8 Hz, 2H), 1.37 (s, 9H), 1.25 (t, *J* = 7.0 Hz, 3H); ^{13}C NMR ($CDCl_3$, 101 MHz): δ 173.7, 173.3, 157.1, 138.3, 132.2, 126.8, 126.5, 111.7, 60.5, 55.2, 51.7, 36.5, 36.3, 34.9, 30.7, 29.9, 14.4. IR (ATR): 2953, 2858, 1733, 1496, 1457, 1359, 1235, 1178, 1095, 1031, 887, 811, 732, 669 cm^{-1}. HRMS (ESI+): *m*/*z* $[M+H]^+$ calculated for $C_{16}H_{25}O_3$: 265.1798; found: 265.1781.

1-(2,5-Di-*tert*-Butylthiophen-3-yl)hexan-1-one (4xaa)

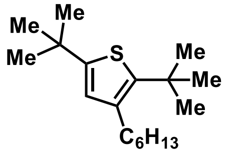

Prepared using General Procedure A with 3-hexylthiophene (33.7 mg, 0.200 mmol, 1 equiv), $FeCl_3$ (9.7 mg, 0.060 mmol, 0.3 equiv), DCE (0.8 mL, 0.25 M), DTBP (37 µL, 0.20 mmol, 1 equiv),** and conc. $HCl_{(aq)}$ (14 µL, 0.15 mmol, 0.75 equiv). Purification by preparative TLC (eluting with hexanes) afforded **4xaa** (46.0 mg, 82%) as a colorless oil. R_f: 0.61 (hexanes). ^1H NMR ($CDCl_3$, 600 MHz): δ 6.53 (s, 1H), 2.64–2.54 (m, 2H), 1.60–1.54 (m, 2H), 1.40 (s, 9H), 1.43–1.29 (m, 6H), 1.33 (s, 9H), 0.90 (t, *J* = 6.9 Hz, 3H); ^{13}C NMR ($CDCl_3$, 101 MHz): δ 150.5, 144.2, 136.2, 125.1, 34.7, 34.2, 32.5, 32.0, 31.9, 31.7, 30.5, 29.9, 22.8, 14.2. IR (ATR): 2956, 2859, 1465, 1362, 1255, 1203, 835 cm^{-1}. HRMS (ESI+): *m*/*z* $[M+H]^+$ calculated for $C_{18}H_{33}S$: 281.2297; found: 281.2315.

3-(*tert*-Butyl)benzo[*b*]thiophene (4ya)

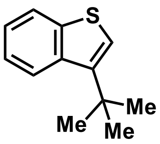

Prepared using General Procedure A with benzo[*b*]thiophene (26.8 mg, 0.200 mmol, 1 equiv), $FeCl_3$ (9.7 mg, 0.060 mmol, 0.3 equiv), DCE (0.8 mL, 0.25 M), DTBP (37 µL, 0.20 mmol, 1 equiv), and conc. $HCl_{(aq)}$ (14 µL, 0.15 mmol, 0.75 equiv). Purification by preparative TLC (eluting with 19:1 hexanes/EtOAc) afforded **4ya** (27.8 mg, 73%) as a colorless oil. R_f: 0.80 (19:1 hexanes/EtOAc). ^1H NMR ($CDCl_3$, 500 MHz): δ 8.07 (d, *J* = 8.2 Hz, 1H), 7.87 (d, *J* = 7.9 Hz, 1H), 7.38–7.28 (m, 2H), 7.11 (s, 1H), 1.51 (s, 9H); ^{13}C NMR ($CDCl_3$, 101 MHz): δ 145.9, 141.8, 138.0, 124.6, 123.5, 123.4, 119.7, 34.7, 30.4. IR (ATR): 2961, 2830, 1457, 1425, 1393, 1364, 1235, 1068, 861, 762, 735, 708 cm^{-1}. HRMS (CI+) *m*/*z* calculated for $C_{12}H_{14}S$ $[M]^+$: 190.0816; found: 190.0824.

General Procedure B – Friedel–Crafts Alkylations with tertiary alcohols

A one-dram vial was charged with a stirring bar, phenol derivative (0.2 mmol, 1 equiv) and brought into a glovebox before adding iron(III) chloride (0.005 mmol, 0.025 equiv), and DCE (0.8 mL, 0.25 M). The vial was closed with a septum screw-cap and removed from the glovebox before sequentially adding alcohol (0.22 mmol, 1.1 equiv) and conc. $HCl_{(aq)}$ (37 %, 12.5 µL, 0.15 mmol, 0.75 equiv), and heated at 50 °C for 24 h, at which time the solution was filtered through a 5” pipette silica plug (approximately half-filled) and eluted with 1:1 hexanes/EtOAc. The solution

** Even with 0.5 equiv DTBP, the dialkylation product was isolated as the major product along with unreacted starting material.

was concentrated in vacuo before purification via preparative TLC (19:1 or 9:1 hexanes/EtOAc) to afford the desired product.

4-*tert*-Butylphenol (3aa)

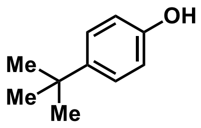

Prepared using General Procedure B with phenol (18.8 mg, 0.200 mmol, 1 equiv), $FeCl_3$ (0.8 mg, 0.005 mmol, 0.025 equiv), DCE (0.8 mL, 0.25 M), *tert*-butanol (21 µL, 0.22 mmol, 1.1 equiv), and conc. $HCl_{(aq)}$ (37%, 12.5 µL, 0.15 mmol, 0.75 equiv). Purification by preparative TLC (eluting with 19:1 hexanes/EtOAc) afforded **3aa** (25.2 mg, 84%) as a light yellow-white solid. R_f: 0.12 (19:1 hexanes/EtOAc). M.p. 90–92 °C. ^{1}H NMR ($CDCl_3$, 500 MHz): δ 7.26 (d, *J* = 7.6 Hz, 2H), 6.77 (d, *J* = 8.6 Hz, 2H), 4.54 (br s, 1H), 1.29 (s, 9H); ^{13}C NMR ($CDCl_3$, 126 MHz): δ 153.2, 143.7, 126.6, 114.9, 34.2, 31.7. IR (ATR): 3230, 2959, 1613, 1447, 1361, 722 cm^{-1}. HRMS (ESI–): *m/z* $[M–H]^-$ calculated for $C_{10}H_{13}O$: 149.0972; found: 149.0978. The spectral data recorded are consistent with those previously reported.[10]

4-*tert*-Amylphenol (3ab)

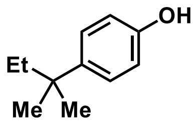

Prepared using General Procedure B with phenol (18.8 mg, 0.200 mmol, 1 equiv), $FeCl_3$ (0.8 mg, 0.005 mmol, 0.025 equiv), DCE (0.8 mL, 0.25 M), 2-methyl-2-butanol (24 µL, 0.22 mmol, 1.1 equiv), and conc. $HCl_{(aq)}$ (37%, 12.5 µL, 0.15 mmol, 0.75 equiv). Purification by preparative TLC (eluting with 19:1 hexanes/EtOAc) afforded **3ab** (27.9 mg, 85%) as a pale yellow-white solid. R_f: 0.18 (19:1 hexanes/EtOAc). M.p. 91–92 °C. ^{1}H NMR ($CDCl_3$, 400 MHz): δ 7.27–7.07 (m, 2H), 6.85–6.67 (m, 2H), 4.84 (br s, 1H), 1.61 (q, *J* = 7.5 Hz, 2H), 1.26 (s, 9H), 0.68 (t, *J* = 7.5 Hz, 3H); ^{13}C NMR ($CDCl_3$, 101 MHz): δ 153.1, 141.9, 127.3, 114.9, 37.4, 37.1, 28.7, 9.3. IR (ATR): 3251, 2963, 1599, 1448, 1375, 705 cm^{-1}. HRMS (ESI–): *m/z* $[M–H]^-$ calculated for $C_{11}H_{15}O$: 163.1128; found: 163.1134.

3ac and 3ac'

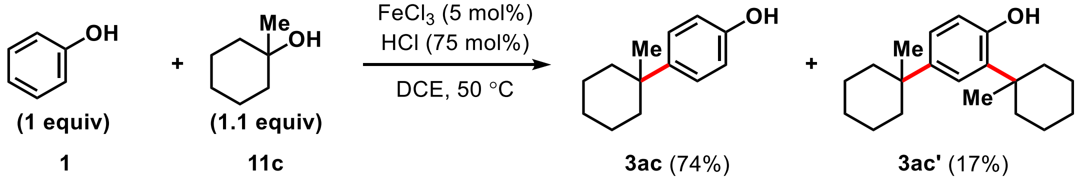

The reaction was performed following General Procedure B with phenol (18.8 mg, 0.200 mmol, 1 equiv), $FeCl_3$ (0.8 mg, 0.005 mmol, 0.025 equiv), DCE (0.8 mL, 0.25 M), 1-methylcyclohexanol (**11c**, 25.1 mg, 0.22 mmol, 1.1 equiv), and conc. $HCl_{(aq)}$ (37%, 12.5 µL, 0.15 mmol, 0.75 equiv). Purification by preparative TLC (eluting with 19:1hexanes/EtOAc) afforded **3ac** (28.2 mg, 74%) as a pale orange-white solid and **2,4-bis(1-methylcyclohexyl)phenol** (**3ac'**, 10.0 mg, 17%) as an orange oil.

4-(1-Methylcyclohexyl)phenol (3ac)

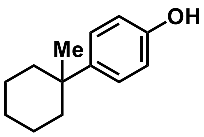

R_f: 0.15 (19:1 hexanes/EtOAc). M.p. 106–108 °C. ^{1}H NMR ($CDCl_3$, 500 MHz): δ 7.24 (d, *J* = 8.2 Hz, 1H), 6.80 (d, *J* = 8.6 Hz, 1H), 4.81 (br s, 1H), 1.99–1.91 (m, 2H), 1.54 (td, *J* = 7.6, 3.8 Hz, 4H), 1.47–1.36 (m, 4H), 1.16 (s, 3H); ^{13}C NMR ($CDCl_3$, 126 MHz): δ 153.0, 142.5, 127.2, 115.1, 38.2, 37.4, 30.8, 26.5, 22.8. IR (ATR): 3228, 2927, 1598, 1444, 1370, 724. HRMS (ESI–): *m/z* [M–H]$^-$ calculated for $C_{13}H_{17}O$: 189.1285; found: 189.1294.

2,4-Bis(1-methylcyclohexyl)phenol (**3ac'**)

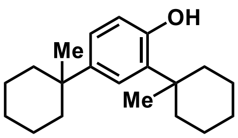

R_f: 0.38 (19:1 hexanes/EtOAc). ^{1}H NMR ($CDCl_3$, 500 MHz): δ 7.30 (d, *J* = 2.4 Hz, 1H), 7.05 (dd, *J* = 8.2, 2.4 Hz, 1H), 6.61 (d, *J* = 8.2 Hz, 1H), 4.72 (br s, 1H), 2.22–2.15 (m, 2H), 1.97–1.90 (m, 2H), 1.73 1.37 (m, 16H), 1.34 (s, 3H), 1.18 (s, 3H); ^{13}C NMR ($CDCl_3$, 126 MHz): δ 151.9, 142.0, 134.6, 125.9, 123.9, 116.5, 38.3, 37.5, 37.2, 26.8, 26.6, 23.0, 22.8. IR (ATR): 3529, 2922, 1605, 1447, 1374, 706 cm^{-1}. HRMS (ESI–): *m/z* [M–H]$^-$ calculated for $C_{20}H_{29}O$: 285.2224; found: 285.2233.

4-(1-Adamantyl)phenol (3ad)

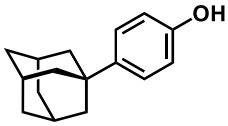

Prepared using General Procedure B with phenol (18.8 mg, 0.200 mmol, 1 equiv), $FeCl_3$ (0.8 mg, 0.005 mmol, 0.025 equiv), DCE (0.8 mL, 0.25 M), 1-adamantanol (33.5 mg, 0.22 mmol, 1.1 equiv), and conc. $HCl_{(aq)}$ (37%, 12.5 µL, 0.15 mmol, 0.75 equiv). Purification by preparative TLC (eluting with 19:1 hexanes/EtOAc) afforded **3ad** (32.4 mg, 71%) as a white solid. R_f: 0.18 (19:1 hexanes/EtOAc). M.p. 176–179 °C. ^{1}H NMR ($CDCl_3$, 400 MHz): δ 7.24 (dd, *J* = 9.2, 2.8 Hz, 2H), 6.80 (d, *J* = 8.6 Hz, 2H), 4.84 (br s, 1H), 2.12–2.06 (m, 3H), 1.89 (d, *J* = 3.1 Hz, 6H), 1.83–1.70 (m, 6H); ^{13}C NMR ($CDCl_3$, 101 MHz): δ 153.3, 144.1, 126.2, 115.0, 43.5, 36.9, 35.7, 29.1. IR (ATR): 3248, 2900, 1597, 1446, 1367, 721

cm^{-1}. HRMS (ESI–): *m/z* [M–H]$^-$ calculated for $C_{16}H_{19}O$: 227.1441; found: 227.1448. The spectral data recorded are consistent with those previously reported.[11]

4-(1-Methylcyclopentyl)phenol (3ae)

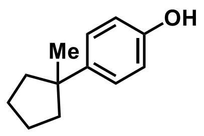

Prepared using General Procedure B with phenol (18.8 mg, 0.20 mmol, 1 equiv), $FeCl_3$ (0.8 mg, 0.005 mmol, 0.025 equiv), DCE (0.8 mL, 0.25 M), 1-methylcyclopentanol (22.0 mg, 0.22 mmol, 1.1 equiv), and conc. $HCl_{(aq)}$ (37%, 12.5 µL, 0.15 mmol, 0.75 equiv). Purification by preparative TLC (eluting with 19:1 hexanes/EtOAc) afforded a 1:3 mixture of **3ae**/phenol (22.6 mg, 23% yield of **3ae**) as a yellow oil. ^{1}H NMR ($CDCl_3$, 500 MHz): 7.20 (d, *J* = 8.5 Hz, 2H), 6.77 (d, *J* = 8.5 Hz, 2H), 5.01 (br s, 1H), 4.93 (br s, 1H), 1.91–1.63 (m, 8H), 1.22 (s, 3H); ^{13}C NMR ($CDCl_3$, 126 MHz): δ 153.2, 143.9, 127.3, 114.9, 40.0, 38.9, 29.7, 23.8; PhOH 155.7, 129.8, 120.8, 115.5.

2,4-Di-*tert*-butylphenol (3ba)

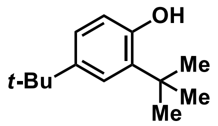

Prepared using General Procedure B with 4-*tert*-butylphenol (34.0 mg, 0.200 mmol, 1 equiv), $FeCl_3$ (0.8 mg, 0.005 mmol, 0.025 equiv), DCE (0.8 mL, 0.25 M), *tert*-butanol (21 µL, 0.22 mmol, 1.1 equiv), and conc. $HCl_{(aq)}$ (37%, 12.5 µL, 0.15 mmol, 0.75 equiv). Purification by preparative TLC (eluting with 19:1 hexanes/EtOAc) afforded **3ba** (35.1 mg, 85%) as a light-orange solid. ^{1}H NMR ($CDCl_3$, 500 MHz): δ 7.30 (d, *J* = 2.4 Hz, 1H), 7.08 (dd, *J* = 8.2, 2.4 Hz, 1H), 6.60 (d, *J* = 8.2 Hz, 1H), 4.63 (s, 1H), 1.42 (s, 9H), 1.29 (s, 9H). HRMS (ESI–): m/z [M–H]$^-$ calculated for $C_{14}H_{21}O$: 206.1671; found: 206.1680. The spectral data recorded are consistent with those previously reported.[4]

2-*tert*-Butyl-4-ethylphenol (3e'a)

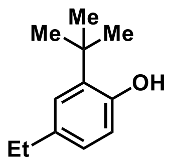

Prepared using General Procedure B with 4-ethylphenol (24.4 mg, 0.200 mmol, 1 equiv), $FeCl_3$ (0.3 mg, 0.002 mmol, 0.01 equiv), DCE (0.8 mL, 0.25 M), *tert*-butanol (21 µL, 0.22 mmol, 1.1 equiv), and conc. $HCl_{(aq)}$ (37%, 12.5 µL, 0.15 mmol, 0.75 equiv). Purification by preparative TLC (eluting with 19:1 hexanes/EtOAc) afforded **3e'a** (20.7 mg, 58%) as an orange oil. R_f: 0.28 (19:1 hexanes/EtOAc). ^{1}H NMR ($CDCl_3$, 500 MHz): δ 7.12 (d, *J* = 2.2 Hz, 1H), 6.93 (dd, *J* = 7.9, 2.2 Hz, 1H), 6.61 (d, *J* = 7.8 Hz, 1H), 4,70 (br s, 1H), 2.60 (q, *J* = 7.6 Hz, 2H), 1.44 (s, 9H), 1.24 (t, *J* = 7.6 Hz, 3H); ^{13}C NMR ($CDCl_3$, 151 MHz): δ 152.3, 136.1, 135.9, 126.8, 126.0, 116.5, 34.6, 29.8, 28.4, 16.1. IR

(ATR): 3528 (br.), 2960, 1608, 1460, 1362, 688 cm^{-1}. HRMS (ESI–): *m/z* [M–H]$^-$ calculated for $C_{12}H_{17}O$: 178.1285; found: 177.1292.

2-*tert*-Butyl-4-chlorophenol (3ca)

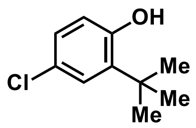

Prepared using General Procedure B with 4-chlorophenol (25.7 mg, 0.200 mmol, 1 equiv), $FeCl_3$ (32.4 mg, 0.2 mmol, 1 equiv), DCE (0.8 mL, 0.25 M), *tert*-butanol (21 µL, 0.22 mmol, 1.1 equiv), and conc. $HCl_{(aq)}$ (37%, 12.5 µL, 0.15 mmol, 0.75 equiv). Purification by preparative TLC (eluting with 19:1 hexanes/EtOAc) afforded **3ca** (18.8 mg, 51%) as a pale-yellow oil. R_f: 0.23 (19:1 hexanes/EtOAc). ^{1}H NMR ($CDCl_3$, 600 MHz): δ 7.21 (d, *J* = 3.0 Hz, 1H), 7.02 (dd, *J* = 8.4, 2.4, 1H), 6.60 (d, *J* = 8.4 Hz, 1H), 4.77 (br s, 1H), 1.38 (s, 9H); ^{13}C NMR ($CDCl_3$, 101 MHz): δ 152.9, 138.2, 127.5, 126.7, 125.58, 117.7, 34.9, 29.5. HRMS (ESI–): m/z [M–H]$^-$ calculated for $C_{10}H_{12}ClO$: 183.0582; found: 183.0590. The spectral data recorded are consistent with those previously reported.[5]

4-*tert*-Butyl-2-benzylphenol (3ka)

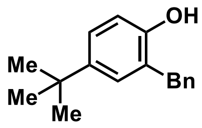

Prepared using General Procedure B with 2-benzylphenol (36.8 mg, 0.200 mmol, 1 equiv), $FeCl_3$ (0.8 mg, 0.005 mmol, 0.025 equiv), DCE (0.8 mL, 0.25 M), *tert*-butanol (21 µL, 0.22 mmol, 1.1 equiv), and conc. $HCl_{(aq)}$ (37%, 12.5 µL, 0.15 mmol, 0.75 equiv). Purification by preparative TLC (eluting with 19:1 hexanes/EtOAc) afforded **3ka** (29.8 mg, 62%) as an orange oil. R_f: 0.34 (19:1 hexanes/EtOAc). ^{1}H NMR (500 MHz, $CDCl_3$) δ 7.33–7.26 (m, 2H), 7.23 (d, *J* = 7.4 Hz, 2H), 7.20 (d, *J* = 7.3 Hz, 1H), 7.17–7.11 (m, 2H), 4.52 (s, 1H), 4.00 (s, 2H), 1.28 (s, 9H). ^{13}C NMR (101 MHz, $CDCl_3$) δ 151.6, 143.8, 140.2, 128.7, 128.2, 126.4, 126.2, 124.7, 115.4, 37.0, 34.2, 31.7. IR (ATR): 3425 (br.), 2960, 1602, 1452, 1363, 697 cm^{-1}. HRMS (ESI–): *m/z* [M–H]$^-$ calculated for $C_{17}H_{19}O$: 239.1441; found: 239.1447.

3d'a and 3d'aa

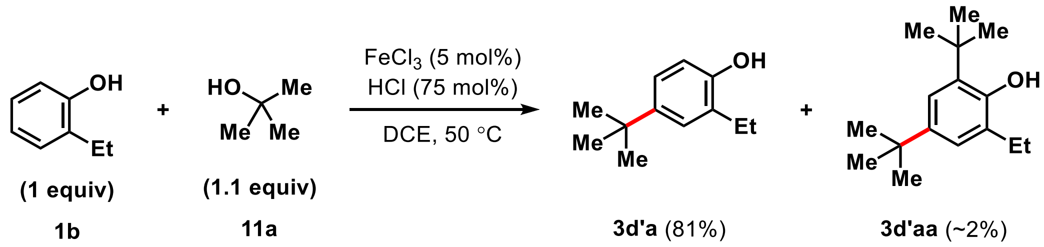

The reaction was performed following General Procedure B with 2-ethylphenol (23.6 µL, 0.200 mmol, 1 equiv), $FeCl_3$ (1.6 mg, 0.010 mmol, 0.05 equiv), DCE (0.8 mL, 0.25 M), *tert*-butanol (**11a**, 21 µL, 0.22 mmol, 1.1 equiv), and conc. $HCl_{(aq)}$ (37%, 12.5 µL, 0.15 mmol, 0.75 equiv). Purification by preparative TLC (eluting with 19:1 hexanes/EtOAc) afforded **3d'a** (28.8 mg, 81%) as a light yellow oil and **2,4-di-*tert*-butyl-6-ethylphenol 3d'aa** (1.4 mg, 2%) as a yellow oil.

4-*tert*-Butyl-2-ethylphenol (3d'a)

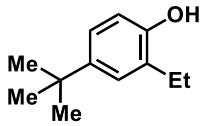

R_f: 0.28 (19:1 hexanes/EtOAc). ^{1}H NMR (500 MHz, $CDCl_3$) δ 7.18 (d, *J* = 2.5 Hz, 1H), 7.12 (dd, *J* = 8.2, 2.5 Hz, 1H), 6.72 (d, *J* = 8.3 Hz, 1H), 4.66 (br s, 1H), 2.66 (q, *J* = 7.6 Hz, 2H), 1.32 (s, 9H), 1.27 (t, *J* = 7.6 Hz, 3H); ^{13}C NMR ($CDCl_3$, 151 MHz): δ 151.1, 143.7, 129.3, 126.5, 123.8, 114.8, 34.2, 31.9, 31.8, 31.7, 23.5, 14.4. IR (ATR): 3397, 2962, 1610, 1462, 1363, 752 cm^{-1}. HRMS (ESI–): *m/z* $[M–H]^-$ calculated for $C_{12}H_{17}O$: 177.1285; found: 177.1292.

2,4-Di-*tert*-butyl-6-ethylphenol (3d'aa)

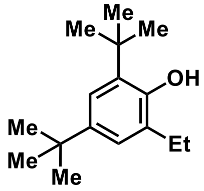

R_f: 0.49 (19:1 hexanes/EtOAc). ^{1}H NMR (500 MHz, $CDCl_3$) δ 7.19 (d, *J* = 2.5 Hz, 1H), 7.03 (d, *J* = 2.5 Hz, 1H), 4.71 (br s, 1H), 2.60 (q, *J* = 7.6 Hz, 2H), 1.43 (s, 8H), 1.30 (s, 8H), 1.28 (t, *J* = 7.6 Hz, 3H); ^{13}C NMR ($CDCl_3$, 151 MHz): 150.0, 142.4, 135.0, 128.4, 123.6, 122.1, 34.9, 34.5, 31.8, 30.1, 23.5, 14.1. IR (ATR): 2956, 1653, 1457, 1445, 1361, 721 cm^{-1}. HRMS (ESI–): *m/z* $[M–H]^-$ calculated for $C_{26}H_{25}O$: 233.1911; found: 234.1918.

4-*tert*-Butyl-2-phenylphenol (3ga)

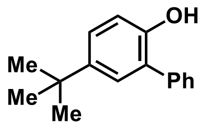

Prepared using General Procedure B with 2-phenylphenol (34.0 mg, 0.200 mmol, 1 equiv), $FeCl_3$ (3.2 mg, 0.02 mmol, 0.1 equiv), DCE (0.8 mL, 0.25 M), *tert*-butanol (21 µL, 0.22 mmol, 1.1 equiv), and conc. $HCl_{(aq)}$ (37%, 12.5 µL, 0.15 mmol, 0.75 equiv). Purification by preparative TLC (eluting with 97:3 hexanes/EtOAc × 4) afforded **3ga** (15.0 mg, 44%) as an orange oil. R_f: 0.14 (97:3 hexanes/EtOAc). ^{1}H NMR (500 MHz, $CDCl_3$) δ 7.53–7.46 (m, 4H), 7.43–7.38 (m, 1H), 7.30 (dd, *J* = 8.5, 2.4 Hz, 1H), 7.25 (d, *J* = 2.5 Hz, 1H), 6.93 (d, *J* = 8.5 Hz, 1H), 5.08 (s, 1H), 1.33 (s, 9H). ^{13}C NMR (126 MHz, $CDCl_3$) δ 150.2, 143.7, 137.8, 129.4, 129.3, 127.9, 127.5, 127.3, 126.2, 115.4, 34.3, 31.7. IR (ATR): 3415 (br.),

2956, 1600, 1463, 1363, 699 cm^{-1}. HRMS (ESI–): *m/z* [M–H]$^-$ calculated for $C_{16}H_{17}O$: 225.1285; found: 225.1292.

2-*tert*-Amyl-5-ethylphenol (3eb)

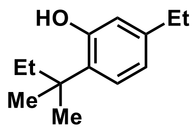

Prepared using General Procedure B with 3-ethylphenol (24.4 µL, 0.200 mmol, 1 equiv), $FeCl_3$ (1.6 mg, 0.010 mmol, 0.05 equiv), DCE (0.8 mL, 0.25 M), 2-methyl-2-butanol (24 µL, 0.22 mmol, 1.1 equiv), and conc. $HCl_{(aq)}$ (37%, 12.5 µL, 0.15 mmol, 0.75 equiv). Purification by preparative TLC (eluting with 19:1 hexanes/EtOAc) afforded **3eb** (25.8 mg, 67%) as a yellow oil. R_f: 0.45 (19:1 hexanes/EtOAc). ^{1}H NMR ($CDCl_3$, 600 MHz): δ 7.13 (d, *J* = 7.9 Hz, 1H), 6.73 (dd, *J* = 7.9, 1.8 Hz, 1H), 6.51 (d, *J* = 1.8 Hz, 1H), 4.76 (br s, 1H), 2.59 (d, *J* = 7.6 Hz, 2H), 1.86 (d, *J* = 7.5 Hz, 2H), 1.37 (s, 6H), 1.23 (t, *J* = 7.6 Hz, 3H), 0.70 (t, *J* = 7.5 Hz, 3H); ^{13}C NMR ($CDCl_3$, 151 MHz): δ 154.1, 143.3, 131.6, 128.4, 120.0, 116.1, 37.9, 33.5, 28.1, 27.9, 15.3, 9.7. IR (ATR): 3530, 2963, 1617, 1460, 1362, 727 cm^{-1}. HRMS (ESI–): *m/z* [M–H]$^-$ calculated for $C_{13}H_{19}O$: 191.1441; found: 191.1450.

2-*tert*-Amyl-5-*tert*-butylphenol (3fb)

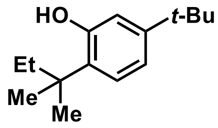

Prepared using General Procedure B with 3-*tert*-butylphenol (30.0 mg, 0.200 mmol, 1 equiv), FeCl*3* (1.6 mg, 0.010 mmol, 0.05 equiv), DCE (0.8 mL, 0.25 M), 2-methyl-2-butanol (24 µL, 0.22 mmol, 1.1 equiv), and conc. $HCl_{(aq)}$ (37%, 12.5 µL, 0.15 mmol, 0.75 equiv). Purification by preparative TLC (eluting with 19:1 hexanes/EtOAc) afforded **3fb** (36.6 mg, 83%) as a yellow-white solid. R_f: 0.42 (19:1 hexanes/EtOAc). M.p. 56–57 °C. ^{1}H NMR ($CDCl_3$, 600 MHz): δ 7.15 (d, *J* = 8.2 Hz, 1H), 6.90 (dd, *J* = 8.2, 2.1 Hz, 1H), 6.68 (d, *J* = 2.1 Hz, 1H), 4.76 (br s, 1H), 1.87 (q, *J* = 7.5 Hz, 2H), 1.38 (s, 6H), 1.31 (d, *J* = 2.2 Hz, 9H), 0.71 (t, *J* = 7.5 Hz, 3H); ^{13}C NMR ($CDCl_3$, 151 MHz): δ 153.8, 150.4, 131.4, 128.0, 117.4, 113.9, 37.8, 34.2, 33.5, 31.4, 27.8, 9.7. IR (ATR): 3473 (br.), 2961, 1608, 1460, 1361, 706 cm^{-1}. HRMS (ESI–): *m/z* [M–H]$^-$ calculated for $C_{15}H_{23}O$: 219.1754; found: 219.1764.

2-*tert*-Amyl-5-phenylphenol (3gb)

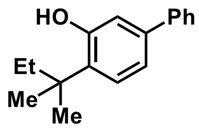

Prepared using General Procedure B with 3-phenylphenol (34.0 mg, 0.200 mmol, 1 equiv), $FeCl_3$ (1.6 mg, 0.010 mmol, 0.05 equiv), DCE (0.8 mL, 0.25 M), 2-methyl-2-butanol (24 µL, 0.22 mmol, 1.1 equiv), and conc. $HCl_{(aq)}$ (37%, 12.5

μL, 0.15 mmol, 0.75 equiv). Purification by preparative TLC (eluting with 19:1 hexanes/EtOAc) afforded **3gb** (34.6 mg, 72%) as an orange-white solid. R_f: 0.29 (19:1 hexanes/EtOAc). M.p. 63–65 °C. ^{1}H NMR ($CDCl_3$, 600 MHz): δ 7.59 (dd, *J* = 8.1, 1.4 Hz, 2H), 7.44 (t, *J* = 7.6 Hz, 2H), 7.38–7.34 (m, 1H), 7.31 (d, *J* = 8.0 Hz, 1H), 7.15 (dd, *J* = 8.1, 1.9 Hz, 1H), 6.89 (d, *J* = 1.9 Hz, 1H), 4.91 (br s, 1H), 1.93 (d, *J* = 7.5 Hz, 2H), 1.44 (s, 6H), 0.75 (t, *J* = 7.5 Hz, 3H); ^{13}C NMR ($CDCl_3$, 151 MHz): δ 154.5, 140.5, 140.2, 133.7, 129.0, 128.8, 127.3, 127.0, 119.3, 115.1, 38.2, 33.4, 27.8, 9.7. IR (ATR): 3541 (br.), 2960, 1601, 1461, 1360, 721 cm^{-1}. HRMS (ESI–): *m/z* [M–H]$^-$ calculated for $C_{17}H_{19}O$: 239.1441; found: 239.1450.

2-*tert*-Amyl-5-methoxyphenol (3ib)

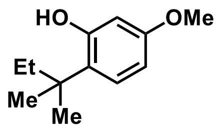

Prepared using General Procedure B with 3-methoxyphenol (24.8 mg, 0.200 mmol, 1 equiv), $FeCl_3$ (0.8 mg, 0.005 mmol, 0.025 equiv), DCE (0.8 mL, 0.25 M), 2-methyl-2-butanol (24 μL, 0.22 mmol, 1.1 equiv), and conc. $HCl_{(aq)}$ (37%, 12.5 μL, 0.15 mmol, 0.75 equiv). Purification by preparative TLC (eluting with 19:1 hexanes/EtOAc) afforded **3ib** (14.4 mg, 37%) as orange-peach crystals. R_f: 0.29 (9:1 hexanes/EtOAc). M.p. 33–34 °C. ^{1}H NMR ($CDCl_3$, 400 MHz): δ 7.10 (d, *J* = 8.6 Hz, 1H), 6.43 (dd, *J* = 8.6, 2.6 Hz, 1H), 6.26 (d, *J* = 2.6 Hz, 1H), 4.84 (br s, 1H), 3.76 (s, 3H), 1.82 (q, *J* = 7.5 Hz, 2H), 1.31 (s, 6H), 0.67 (t, *J* = 7.5 Hz, 3H); ^{13}C NMR ($CDCl_3$, 151 MHz): δ 158.7, 155.1, 129.1, 127.0, 105.0, 103.1, 55.4, 37.7, 33.6, 29.9, 28.1, 9.7. IR (ATR): 3411, 2960, 1613, 1416, 1376, 737 cm^{-1}. HRMS (ESI–): *m/z* [M–H]$^-$ calculated for $C_{12}H_{17}O_2$: 193.1234; found: 193.1241.

3mb and 3mb’

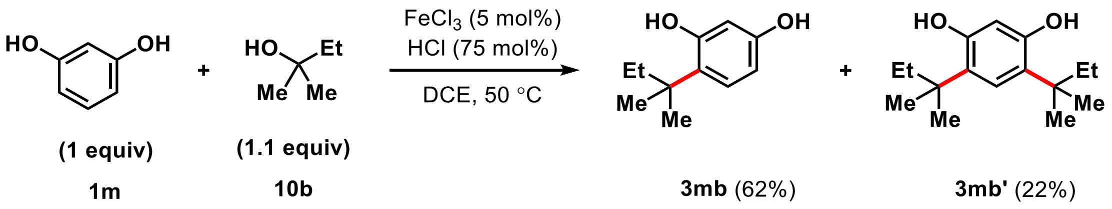

The reaction was performed following General Procedure B with resorcinol (22.0 mg, 0.200 mmol, 1 equiv), $FeCl_3$ (1.6 mg, 0.010 mmol, 0.05 equiv), DCE (0.8 mL, 0.25 M), 2-methyl-2-butanol (24 μL, 0.22 mmol, 1.1 equiv), and conc. $HCl_{(aq)}$ (37%, 12.5 μL, 0.15 mmol, 0.75 equiv). Purification by preparative TLC (eluting with 19:1 hexanes/EtOAc) afforded **3mb** (22.2 mg, 62%) as an orange oil and **4,6-di-*tert*-amyl-resorcinol** (**3mb’**, 10.9 mg, 22%) as a colorless oil.

4-*tert*-Amyl-resorcinol (3mb)

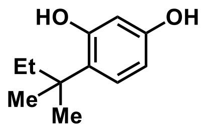

R_f: 0.39 (7:3 hexanes/EtOAc). ^{1}H NMR ($CDCl_3$, 600 MHz): δ 7.03 (d, *J* = 8.5 Hz, 1H), 6.35 (dd, *J* = 8.5, 2.6 Hz, 1H), 6.23 (d, *J* = 2.6 Hz, 1H), 5.20 (br s, 1H), 1.80 (q, *J* = 7.5 Hz, 2H), 1.32 (s, 6H), 0.65 (t, *J* = 7.5 Hz, 3H); ^{13}C NMR ($CDCl_3$, 151 MHz): δ 155.3, 154.4, 129.3, 127.2, 107.1, 104.1, 37.7, 33.5, 28.0, 9.6. IR (ATR): 3365, 2963, 1600, 1439, 1374, 704 cm^{-1}. HRMS (ESI–): *m/z* [M–H]$^-$ calculated for $C_{11}H_{15}O_2$: 179.1078; found: 179.1082.

4,6-Di-*tert*-amyl-resorcinol (3mb')

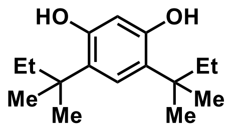

R_f: 0.37 (7:3 hexanes/EtOAc). ^{1}H NMR (500 MHz, $CDCl_3$) δ 6.99 (s, 1H), 6.05 (s, 1H), 4.60 (br s, 2H), 1.78 (q, *J* = 7.5 Hz, 4H), 1.34 (s, 12H), 0.67 (t, *J* = 7.4 Hz, 6H); ^{13}C NMR ($CDCl_3$, 151 MHz): δ 152.6, 128.4, 125.6, 105.4, 37.8, 33.9, 28.1, 9.6. IR (ATR): 3523, 3354, 2959, 1611, 1403, 1376, 703 cm^{-1}. HRMS (ESI–): *m/z* [M–H]$^-$ calculated for $C_{16}H_{25}O_2$: 249.1860; found: 249.1865.

3jb and 3jb'

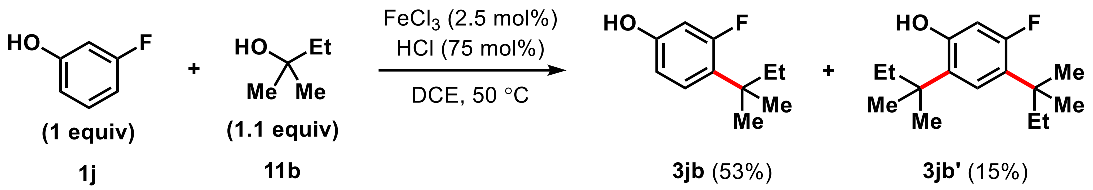

The reaction was performed following General Procedure B with 3-fluorophenol (18.1 µL, 0.200 mmol, 1 equiv), $FeCl_3$ (0.8 mg, 0.005 mmol, 0.025 equiv), DCE (0.8 mL, 0.25 M), 2-methyl-2-butanol (24 µL, 0.22 mmol, 1.1 equiv), and conc. $HCl_{(aq)}$ (37%, 12.5 µL, 0.15 mmol, 0.75 equiv). Purification by preparative TLC (eluting with 19:1 hexanes/EtOAc) afforded **3jb** (19.3 mg, 53%) as a white solid and **2,4-di-*tert*-amyl-5-fluorophenol** (**3jb'**, 7.8 mg, 15%) as a colorless oil.

4-*tert*-Amyl-3-fluorophenol (3jb)

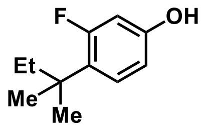

R_f: 0.26 (9:1 hexanes/EtOAc). M.p. 72–74 °C. IR (ATR): 3256, 2966, 1623, 1444, 1377, 738 cm^{-1}. ^{1}H NMR ($CDCl_3$, 500 MHz): δ 7.07 (t, *J* = 9.1 Hz, 1H), 6.59–6.46 (m, 2H), 4.72 (br s, 1H), 1.76–1.67 (m, 2H), 1.29 (s, 6H), 0.66 (t, *J* = 7.5 Hz, 3H); ^{13}C NMR ($CDCl_3$, 126 MHz): δ 163.2, 161.2, 154.8 (d, *J* = 12.0 Hz), 129.1 (d, *J* =

8.1 Hz), 110.4 (d, *J* = 3.0 Hz), 104.1 (d, *J* = 27.9 Hz), 37.5 (d, *J* = 3.4 Hz), 34.4 (d, *J* = 4.1 Hz), 28.0 (d, *J* = 2.9 Hz), 9.5; ^{19}F NMR ($CDCl_3$, 564 MHz): δ 107.6 (t, *J* = 12.1 Hz). IR (ATR): 3256, 2966, 1623, 1444, 1377, 738 cm^{-1}. HRMS (ESI–): *m/z* $[M–H]^-$ calculated for $C_{11}H_{14}FO$: 181.1034; found: 181.1040.

2,4-Di-*tert*-amyl-5-fluorophenol (3jb')

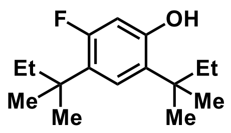

R_f: 0.44 (9:1 hexanes/EtOAc). IR (ATR): 3262, 1617, 1590, 1462, 1377, 703 cm^{-1}. ^{1}H NMR (500 MHz, $CDCl_3$) δ 7.01 (d, *J* = 9.6 Hz, 1H), 6.34 (d, *J* = 13.1 Hz, 1H), 4.65 (br s, 1H), 1.80 (q, *J* = 7.5 Hz, 2H), 1.70 (q, *J* = 7.5 Hz, 2H), 1.34 (s, 6H), 1.30 (s, 6H), 0.66 (qd, *J* = 7.5, 1.9 Hz, 6H); ^{13}C NMR ($CDCl_3$, 126 MHz): δ 158.9, 152.8 (d, *J* = 10.2 Hz), 129.0, 128.1 (d, *J* = 7.6 Hz), 126.2, 104.8 (d, *J* = 27.2 Hz), 38.1, 37.6 (d, *J* = 3.5 Hz), 34.7 (d, *J* = 3.7 Hz), 33.5, 28.1 (d, *J* = 3.0 Hz), 28.0, 9.5 (d, *J* = 10.2 Hz); ^{19}F NMR ($CDCl_3$, 564 MHz): δ 114.3 (t, *J* = 11.3 Hz). IR (ATR): 3262, 1617, 1590, 1462, 1377, 703 cm^{-1}. HRMS (ESI–): *m/z* $[M–H]^-$ calculated for $C_{16}H_{24}FO$: 251.1817; found: 251.1824.

7-(*tert*-Butyl)-2,2-dimethylchromane (3fh)

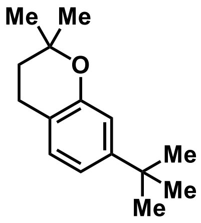

Prepared using General Procedure A with 3-*tert*-butylphenol (30.1 mg, 0.201 mmol, 1 equiv), $FeCl_3$ (1.0 mg, 6.2 µmol, 0.025 equiv), DCE (0.8 mL, 0.25 M), 2-methyl-3-buten-2-ol (23 µL, 0.22 mmol, 1.1 equiv), and conc. $HCl_{(aq)}$ (37%, 12.5 µL, 0.15 mmol, 0.75 equiv). Purification by preparative TLC (eluting with 9:1 hexanes/EtOAc) afforded **3fh** (20.8 mg, 48%) as a pale-yellow oil. R_f: 0.32 (49:1 hexanes/EtOAc). ^{1}H NMR (500 MHz, $CDCl_3$) δ 7.01 (d, *J* = 8.0 Hz, 1H), 6.88 (dd, *J* = 8.0, 2.0 Hz, 1H), 6.83 (d, *J* = 2.0 Hz, 1H), 2.75 (t, *J* = 6.7 Hz, 2H), 1.80 (t, *J* = 6.8 Hz, 2H), 1.35 (s, 6H), 1.30 (s, 9H).; ^{13}C NMR (151 MHz, $CDCl_3$) δ 153.6, 150.9, 129.1, 117.9, 117.0, 114.3, 74.2, 34.6, 33.0, 31.5, 30.5, 27.1, 27.0, 22.2. IR (ATR): 2962, 2867, 1620, 1569, 1502, 1452, 1412, 1382, 1367, 1344, 1306, 1277, 1253, 1233, 1199, 1157, 1121, 1093, 1055, 1026, 1011, 987, 973, 941, 907, 893, 868, 807, 730, 715, 686, 645 cm^{-1}. HRMS (ESI+): *m/z* $[M+H]^+$ calculated for $C_{15}H_{23}O$: 219.1743; found: 219.1751.

1,4-Di-*tert*-butyl-2-methoxybenzene (4va)

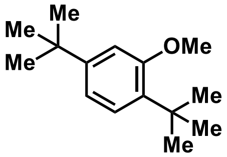

Prepared using General Procedure B with 3-(*tert*-butyl)-1-methoxybenzene (32.8 mg, 0.200 mmol, 1 equiv), $FeCl_3$ (9.7 mg, 0.060 mmol, 0.3 equiv), DCE

(0.8 mL, 0.25 M), *tert*-butanol (19 µL, 0.20 mmol, 1 equiv), and conc. $HBr_{(aq)}$ (3 µL, 0.03 mmol, 0.15 equiv). Purification by preparative TLC (eluting with 19:1 hexanes/EtOAc) afforded **4va** (33.1 mg, 75%) as a colorless oil. R_f: 0.77 (19:1 hexanes/EtOAc). ^{1}H NMR ($CDCl_3$,600 MHz): δ 7.22–7.15 (m, 1H), 7.01–6.81 (m, 2H), 3.85 (s, 3H), 1.36 (s, 9H), 1.32 (s, 9H); ^{13}C NMR ($CDCl_3$, 101 MHz): δ 158.4, 150.2, 135.4, 126.2, 117.1, 109.3, 55.1, 34.6, 34.5, 31.5, 30.0. IR (ATR): 2955, 2830, 1611, 1563, 1462, 1399, 1360, 1268, 1228, 1158, 1079, 1039, 912, 853, 818, 734, 658 cm^{-1}. HRMS (ESI+): *m*/*z* $[M+H]^+$ calculated for $C_{15}H_{25}O$: 221.1900; found: 221.1892.

1,4-Di-*tert*-Butyl-2-ethoxybenzene (4ua)

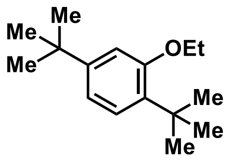

Prepared using General Procedure B with 3-(*tert*-butyl)-1-ethoxybenzene (35.7 mg, 0.200 mmol, 1 equiv), $FeCl_3$ (9.7 mg, 0.060 mmol, 0.3 equiv), DCE (0.8 mL, 0.25 M), *tert*-butanol (19 µL, 0.20 mmol, 1 equiv), and conc. $HBr_{(aq)}$ (3 µL, 0.03 mmol, 0.15 equiv). Purification by preparative TLC (eluting with hexanes) afforded **4ua** (40.3 mg, 86%) as a pale-yellow oil. R_f: 0.78 (19:1 hexanes/EtOAc). ^{1}H NMR ($CDCl_3$, 400 MHz): δ 7.19 (d, *J* = 8.6 Hz, 1H), 6.92–6.86 (m, 2H), 4.08 (q, *J* = 6.9 Hz, 2H), 1.47 (t, *J* = 7.0 Hz, 3H), 1.39 (s, 9H), 1.31 (s, 9H); ^{13}C NMR ($CDCl_3$, 101 MHz): δ 157.6, 150.1, 135.3, 126.2, 116.8, 109.8, 63.4, 34.6, 34.6, 31.8, 31.5, 30.0, 15.2. IR (ATR): 3454, 2978, 2858, 1736, 1503, 1369, 1310, 1255, 1159, 1131, 1097, 1071, 958, 836, 779 cm^{-1}. HRMS (ESI+): *m*/*z* $[M+H]^+$ calculated for $C_{16}H_{27}O$: 235.2056; found: 235.2052.

5-(*tert*-Butyl)benzo[*d*][1,3]dioxole (4sa)

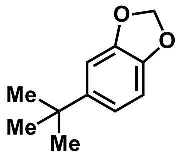

Prepared using General Procedure B with 1,3-benzodioxole (24.4 mg, 0.200 mmol, 1 equiv), $FeCl_3$ (9.7 mg, 0.060 mmol, 0.3 equiv), DCE (0.8 mL, 0.25 M), *tert*-butanol (19 µL, 0.20 mmol, 1 equiv), and conc. $HBr_{(aq)}$ (3 µL, 0.03 mmol, 0.15 equiv). Purification by preparative TLC (eluting with 19:1 hexanes/EtOAc) afforded **4sa** (12.1 mg, 34%) as a colorless oil. R_f: 0.46 (19:1 hexanes/EtOAc). ^{1}H NMR ($CDCl_3$, 400 MHz): δ 6.90 (d, *J* = 1.8 Hz, 1H), 6.83 (dd, *J* = 8.1, 1.8 Hz, 1H), 6.74 (d, *J* = 8.2 Hz, 1H), 5.92 (s, 2H), 1.28 (s, 9H); ^{13}C NMR ($CDCl_3$, 101 MHz): δ 147.5, 145.6, 145.2, 118.0, 107.8, 106.5, 100.9, 34.8, 31.7. IR (ATR): 2957, 2869, 1507, 1488, 1364, 1255, 1230, 1112, 1041, 939, 909, 859, 807, 640 cm^{-1}. HRMS (ESI+): *m*/*z* $[M+H]^+$ calculated for $C_{11}H_{15}O_2$: 179.1067; found: 179.1056.

1-(3-Bromopropoxy)-4-*tert*-butylbenzene (4na)

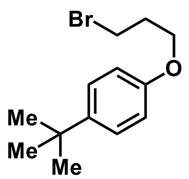

Prepared using General Procedure B with 3-phenoxypropyl bromide (43.0 mg, 0.200 mmol, 1 equiv), $FeBr_3$ (5.9 mg, 0.020 mmol, 0.1 equiv), DCE (0.8 mL, 0.25 M), *tert*-butanol (19 µL, 0.20 mmol, 1 equiv), and conc. $HBr_{(aq)}$ (3 µL, 0.03 mmol, 0.15 equiv). Purification by preparative TLC (eluting with 19:1 hexanes/EtOAc) afforded **4na** (51.0 mg, 94%) as a colorless oil. R_f: 0.66 (hexanes). ^{1}H NMR ($CDCl_3$, 600 MHz): δ 7.31 (d, J = 9.0 Hz, 2H), 6.85 (d, J = 9.0 Hz, 2H), 4.12 (t, J = 6.0 Hz, 2H), 3.64 (t, J = 6.0 Hz, 2H), 2.31 (quint, J = 6.6 Hz, 2H), 1.30 (s, 9H); ^{13}C NMR ($CDCl_3$, 101 MHz): δ 157.1, 156.5, 143.7, 129.6, 126.4, 115.1, 114.1, 65.3, 34.2, 32.6, 31.7, 30.3. IR (ATR): 3041, 2960, 2868, 2369, 1609, 1582, 1512, 1469, 1435, 1420, 1389, 1363, 1294, 1240, 1183, 1117, 1032, 931, 827, 774, 753, 652, 552 cm^{-1}. HRMS (CI+): m/z $[M]^+$ calculated for $C_{13}H_{19}BrO$: 270.0619; found: 270.0619; $[M+2]^+$ calculated for $C_{13}H_{19}BrO$: 272.0600; found 272.0584; 1:1 intensity for $[M]^+/[M+2]^+$ signals.

4-(*tert*-Butyl)-1-methoxy-2-methylbenzene (4oa)

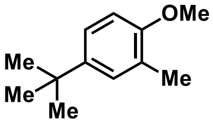

Prepared using General Procedure B with 2-methyl-methoxybenzene (24.4 mg, 0.200 mmol, 1 equiv), $FeCl_3$ (9.7 mg, 0.060 mmol, 0.3 equiv), DCE (0.8 mL, 0.25 M), *tert*-butanol (19 µL, 0.20 mmol, 1 equiv), and conc. $HBr_{(aq)}$ (3 µL, 0.03 mmol, 0.15 equiv). Purification by preparative TLC (eluting with 19:1 hexanes/EtOAc) afforded **4oa** (33.5 mg, 94%) as a colorless oil. R_f: 0.62 (19:1 hexanes/EtOAc). ^{1}H NMR ($CDCl_3$, 600 MHz): δ 7.21–7.16 (m, 2H), 6.80–6.76 (m, 1H), 3.83 (s, 3H), 2.24 (s, 3H), 1.31 (s, 9H); ^{13}C NMR ($CDCl_3$, 101 MHz): δ 155.6, 143.0, 128.0, 126.0, 123.3, 109.5, 55.4, 34.1, 31.7, 16.6. The spectral data recorded are consistent with those previously reported.[8]

4-*tert*-Amyl-2-methylanisole (4ob)

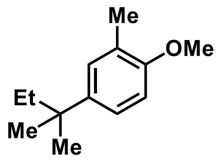

Prepared using General Procedure B with 2-methylanisole (24.4 mg, 0.200 mmol, 1 equiv), $FeBr_3$ (0.59 mg, 0.0020 mmol, 0.01 equiv), DCE (0.8 mL, 0.25 M), amyl alcohol (21.6 µL, 0.2 mmol, 1 equiv), and conc. $HBr_{(aq)}$ (2 µL, 0.02 mmol, 0.1 equiv). Purification by preparative TLC (eluting with 19:1 hexanes/EtOAc) afforded **4ob** (36.9 mg, 96%) as a colorless oil. R_f: 0.69 (19:1 hexanes/EtOAc). ^{1}H NMR ($CDCl_3$, 400 MHz): δ 7.17–7.09 (m, 2H), 6.81–6.76 (m, 1H), 3.84 (s, 3H), 2.25 (s, 3H),

1.63 (q, J = 7.4 Hz, 2H), 1.28 (s, 6H), 0.71 (t, J = 7.4 Hz, 3H); ^{13}C NMR ($CDCl_3$, 400 MHz): δ 155.6, 141.3, 128.6, 125.8, 124.1, 109.4, 55.4, 37.0, 31.7, 28.8, 16.7, 9.3. IR (ATR): 2961, 2856, 1608, 1506, 1463, 1441, 1377, 1304, 1261, 1246, 1142, 1114, 1036, 994, 881, 807, 776, 775, 731, 654, 612, 573 cm^{-1}. HRMS (CI+): m/z $[M]^+$ calculated for $C_{13}H_{20}O$: 192.1514; found: 192.1516.

1-Methoxy-2-methyl-4-(1-methylcyclohexyl)benzene (4oc)

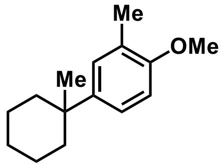

Prepared using General Procedure B with 2-methylanisole (24.4 mg, 0.200 mmol, 1 equiv), $FeBr_3$ (5.9 mg, 0.020 mmol, 0.1 equiv), DCE (0.8 mL, 0.25 M), 1-methylcyclohexanol (24.9 µL, 0.20 mmol, 1 equiv), and conc. $HBr_{(aq)}$ (3 µL, 0.03 mmol, 0.15 equiv). Purification by preparative TLC (eluting with 19:1 hexanes/EtOAc) afforded **4oc** (43.2 mg, 99%) as a pale-yellow oil. R_f: 0.61 (19:1 hexanes/EtOAc). ^{1}H NMR ($CDCl_3$, 400 MHz): δ 7.22–7.12 (m, 2H), 6.85–6.78 (m, 1H), 3.85 (s, 3H), 2.27 (s, 3H), 2.07–1.92 (m, 2H), 1.65–1.52 (m, 4H), 1.52–1.37 (m, 4H), 1.20 (s, 3H); ^{13}C NMR ($CDCl_3$, 101 MHz): δ 155.5, 141.8, 128.5, 126.1, 124.0, 109.7, 55.4, 38.2, 37.3, 30.7, 26.6, 22.8, 16.7. IR (ATR): 2924, 2855, 1609, 1507, 1465, 1453, 1374, 1302, 1243, 1173, 1145, 1116, 1034, 996, 965, 883, 806, 755, 731, 650, 617, 608, 573, 564, 552, 529 cm^{-1}. HRMS (ESI+): m/z $[M+H]^+$ calculated for $C_{15}H_{23}O$: 219.1743; found: 219.1735.

1-(4-Methoxy-3-methyl-phenyl)-adamantane (4od)

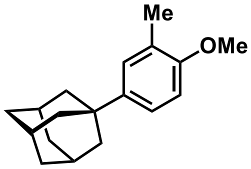

Prepared using General Procedure B with 2-methylanisole (24.4 mg, 0.200 mmol, 1 equiv), $FeBr_3$ (5.9 mg, 0.020 mmol, 0.1 equiv), DCE (0.8 mL, 0.25 M), adamantanol (30.6 mg, 0.200 mmol, 1 equiv), and conc. $HBr_{(aq)}$ (3 µL, 0.03 mmol, 0.75 equiv). Purification by preparative TLC (eluting with 19:1 hexanes/EtOAc) afforded **4od** (50.8 mg, 99%) as a white solid. R_f: 0.49 (19:1 hexanes/EtOAc). M.p. 88–89°C. ^{1}H NMR ($CDCl_3$, 400 MHz): δ 7.17–7.11 (m, 2H), 6.81–6.76 (m, 1H), 3.81 (s, 3H), 2.23 (s, 3H), 2.08 (s, 3H), 1.94–1.86 (m, 6H), 1.81–1.70 (m, 6H); ^{13}C NMR ($CDCl_3$, 101 MHz): δ 155.7, 143.5, 127.5, 126.0, 122.9, 109.6, 55.4, 43.6, 37.0, 35.6, 29.2, 16.7. The spectral data recorded are consistent with those previously reported.[12]

1-Methoxy-2-methyl-4-(1-methylcyclopentyl)benzene (4oe)

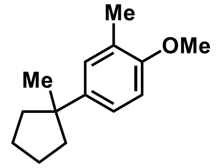

Prepared using General Procedure B with 2-methylanisole (24.4 mg, 0.200 mmol, 1 equiv), $FeBr_3$ (17.7 mg, 0.060 mmol, 0.3 equiv), DCE (0.8 mL, 0.25 M), 1-methylcyclopentanol (25.2 µL, 0.20 mmol, 1 equiv), and conc. $HBr_{(aq)}$ (3 µL, 0.03 mmol, 0.15 equiv). Purification by preparative TLC (eluting with 19:1 hexanes/EtOAc) afforded **4oe** (29.8 mg, 73%) as a colorless oil. R_f: 0.66 (19:1 hexanes/EtOAc). ^1H NMR ($CDCl_3$, 400 MHz): δ 7.16–7.07 (m, 2H), 6.80–6.72 (m, 1H), 3.82 (s, 3H), 2.23 (s, 3H), 1.92–1.83 (m, 2H), 1.83–1.66 (m, 6H), 1.23 (s, 3H); ^{13}C NMR ($CDCl_3$, 101 MHz): δ 155.6, 143.4, 128.8, 126.0, 124.0, 109.6, 55.5, 46.5, 40.0, 29.7, 23.9, 16.6. IR (ATR): 2952, 2832, 1610, 1505, 1464, 1371, 1328, 1296, 1244, 1138, 1035, 994, 882, 806, 754, 714, 609, 575, 559, 547, 530 cm^{-1}. HRMS (ESI+): *m/z* $[M+H]^+$ calculated for $C_{14}H_{21}O$: 205.1587; found: 205.1579.

1-Methoxy-2-methyl-4-(2-phenylpropan-2-yl)benzene (4of)

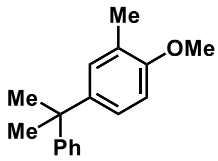

Prepared using General Procedure B with 2-methylanisole (24.4 mg, 0.200 mmol, 1 equiv), $FeBr_3$ (17.7 mg, 0.060 mmol, 0.3 equiv), DCE (0.8 mL, 0.25 M), cumyl alcohol (28 µL, 0.2 mmol, 1 equiv), and conc. $HBr_{(aq)}$ (3 µL, 0.03 mmol, 0.15 equiv). Purification by preparative TLC (eluting with 19:1 hexanes/EtOAc) afforded **4of** (28.4 mg, 59%) as a pale-yellow oil. R_f: 0.63 (19:1 hexanes/EtOAc). ^1H NMR ($CDCl_3$, 400 MHz): δ 7.31–7.22 (m, 4H), 7.20–7.15 (m, 1H), 7.04 (dd, J = 8.4, 3.0 Hz, 1H), 7.00 (d, J = 2.5 Hz, 1H), 6.74 (d, J = 8.4 Hz, 1H), 3.82 (s, 3H), 2.19 (s, 3H), 1.67 (s, 6H); ^{13}C NMR ($CDCl_3$, 101 MHz): δ 155.8, 151.2, 142.5, 129.5, 128.1, 126.9, 126.0, 125.6, 124.9, 109.4, 55.4, 42.3, 31.1, 16.6. IR (ATR): 2964, 2833, 1608, 1502, 1464, 1442, 1362, 1306, 1247, 1175, 1151, 1135, 1112, 1031, 995, 893, 809, 771, 154, 731, 617, 594, 560, 539 cm^{-1}. HRMS (ESI+): *m/z* $[M+H]^+$ calculated for $C_{17}H_{21}O$: 241.1587; found: 240.1524.

1-Methoxy-2-ethyl-4-(1-methylcyclohexyl)benzene (4pc)

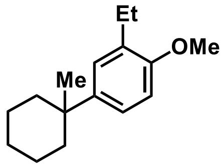

Prepared using General Procedure B with 2-ethylanisole (27.2 mg, 0.200 mmol, 1 equiv), $FeBr_3$ (5.9 mg, 0.020 mmol, 0.1 equiv), DCE (0.8 mL, 0.25 M), 1-methylcyclohexanol (24.9 µL, 0.20 mmol, 1 equiv), and conc. $HBr_{(aq)}$ (3 µL, 0.03 mmol, 0.75 equiv). Purification by preparative TLC (eluting with 19:1 hexanes/EtOAc) afforded **4pc** (46.0 mg, 99%) as a pale-yellow oil. R_f: 0.68 (19:1 hexanes/EtOAc).

^{1}H NMR ($CDCl_3$, 600 MHz): δ 7.16 (m, 2H), 6.79 (d, J = 9.0 Hz, 1H), 3.81 (s, 3H), 2.64 (q, J = 7.2 Hz, 2H), 1.96 (m, 2H), 1.55 (m, 5H), 1.45 (m, 3H), 1.20 (t, J = 7.2 Hz, 3H), 1.169 (s, 3H); ^{13}C NMR ($CDCl_3$, 150 MHz): δ 155.0, 141.9, 132.0, 126.8, 123.9, 109.8, 55.3, 38.1, 37.2, 26.5, 23.7, 22.7, 14.5. IR (ATR): 2926, 2856, 1607, 1503, 1453, 1373, 1306, 1245, 1147, 1134, 1117, 1052, 1030, 962, 891, 854, 807, 750, 650, 605, 553 cm^{-1}. HRMS (ESI+): m/z [M+H]$^+$ calculated for $C_{16}H_{25}O$: 233.1900; found: 233.1913.

4-(*tert*-Butyl)-3-methoxyanisole (4za)

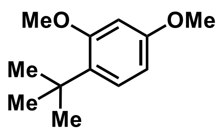

Prepared using General Procedure B with 1,3-dimethoxybenzene (27.6 mg, 0.200 mmol, 1 equiv), $FeBr_3$ (17.7 mg, 0.060 mmol, 0.3 equiv), DCE (0.8 mL, 0.25 M), *tert*-butanol (19 µL, 0.20 mmol, 1 equiv), and conc. $HBr_{(aq)}$ (3 µL, 0.03 mmol, 0.15 equiv). Purification by preparative TLC (eluting with 19:1 hexanes/EtOAc) afforded **4za** (23.3 mg, 60%) as a colorless oil. R_f: 0.47 (19:1 hexanes/EtOAc). ^{1}H NMR ($CDCl_3$, 600 MHz): δ 7.21 (d, J = 8.4 Hz, 1H), 6.51 (s, 1H), 6.45 (d, J = 8.4 Hz), 3.82 (s, 3H), 3.80 (s, 3H), 1.35 (s, 9H); ^{13}C NMR ($CDCl_3$, 101 MHz): δ 159.5, 159.1, 131.0, 126.9, 103.3, 99.8, 55.4, 55.1, 34.4, 30.0. IR (ATR): 2996, 2953, 2834, 1611, 1581, 1502, 1484, 1461, 1438, 1412, 1389, 1358, 1305, 1274, 1257, 1210, 1151, 1145, 1096, 1035, 939, 925, 833, 795, 732, 659, 634, 601, 543 cm^{-1}. HRMS (ESI+): m/z [M+H]$^+$ calculated for $C_{12}H_{19}O_2$: 195.1380; found: 195.1371.

***N*-(4-(*tert*-Butyl)-3-methoxyphenyl)-2,2,2-trifluoroacetamide (4z'a)**

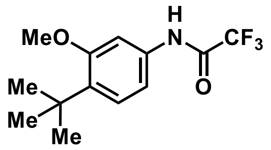

Prepared using General Procedure B with *N*-(3-methoxyphenyl)-2,2,2-trifluoroacetamide (43.8 mg, 0.200 mmol, 1 equiv), $FeBr_3$ (17.7 mg, 0.060 mmol, 0.3 equiv), DCE (0.8 mL, 0.25 M), *tert*-butanol (19 µL, 0.20 mmol, 1 equiv), and conc. $HCl_{(aq)}$ (14 µL, 0.15 mmol, 0.75 equiv). Purification by preparative TLC (eluting with 19:1 hexanes/EtOAc) afforded **4z'a** (30.8 mg, 56%) as a light orange oil. R_f: 0.32 (19:1 hexanes/EtOAc). ^{1}H NMR ($CDCl_3$, 600 MHz): δ 7.86 (br s, 1H), 7.32 (d, J = 1.8 Hz, 1H), 7.25 (d, J = 8.4 Hz, 1H), 6.91 (dd, J = 8.4, 2.4 Hz, 1H), 3.85 (s, 3H), 1.35 (s, 9H); ^{13}C NMR ($CDCl_3$, 101 MHz): δ 159.1, 155.0 (q, J = 37.3 Hz), 136.9, 134.2, 127.1, 116.1 (q, J = 288.8 Hz), 111.9, 104.4, 55.3, 34.9, 29.7. IR (ATR): 3292, 3141, 2958, 2836, 1701, 1607, 1541, 1505, 1464, 1361, 1297, 1215, 1153, 1086, 1038, 968, 814, 716, 622 cm^{-1}. HRMS (ESI+): m/z [M+H]$^+$ calculated for $C_{13}H_{17}F_3NO_2$: 275.1206; found: 275.1219.

1-*tert*-Butyl-4-iodo-2-methoxybenzene (4z”a)

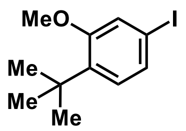

Prepared using General Procedure B with 3-iodoanisole (46.8 mg, 0.200 mmol, 1 equiv), $FeBr_3$ (45.6 mg, 0.20 mmol, 1 equiv), DCE (0.8 mL, 0.25 M), *tert*-butanol (19 µL, 0.20 mmol, 1 equiv), and conc. $HBr_{(aq)}$ (3 µL, 0.03 mmol, 0.15 equiv). Purification by preparative TLC (eluting with 19:1 hexanes/EtOAc) afforded **4z”a** (11.6 mg, 20%) as an orange oil. R_f: 0.72 (19:1 hexanes/EtOAc). ^{1}H NMR ($CDCl_3$, 400 MHz): δ 7.15 (dd, *J* = 8.2, 1.8 Hz, 1H), 7.06 (d, *J* = 1.8 Hz, 1H), 6.90 (d, *J* = 8.2 Hz, 1H), 3.75 (s, 3H), 1.26 (s, 9H); ^{13}C NMR ($CDCl_3$, 101 MHz): δ 159.2, 138.3, 129.6, 128.4, 120.8, 91.4, 55.4, 34.9, 29.6. IR (ATR): 2997, 2866, 1579, 1558, 1485, 1461, 1382, 1359, 1290, 1233, 1201, 1180, 1142, 1105, 1075, 1027, 932, 849, 801, 611, 583, 548, 533 cm^{-1}. HRMS (CI+): *m*/*z* [M]$^+$ calculated for $C_{11}H_{15}IO$: 290.0168; found: 290.0170.

1-(5-Ethyl-2-methoxyphenyl)-adamantane (4pd)

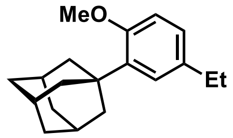

Prepared using General Procedure B with 4-ethylanisole (27.2 mg, 0.200 mmol, 1 equiv), $FeBr_3$ (17.7 mg, 0.06 mmol, 0.3 equiv), DCE (0.8 mL, 0.25 M), adamantanol (30.6 mg, 0.200 mmol, 1 equiv), and conc. $HCl_{(aq)}$ (14 µL, 0.15 mmol, 0.75 equiv). Purification by preparative TLC (eluting with 19:1 hexanes/EtOAc) afforded **4pd** (48.7 mg, 90%) as a cream-colored solid. R_f: 0.67 (19:1 hexanes/EtOAc). M.p. 58–59 °C. ^{1}H NMR ($CDCl_3$, 400 MHz): δ 7.05 (d, *J* = 2.3 Hz, 1H), 7.00 (dd, *J* = 8.2, 2.3 Hz, 1H), 6.80 (d, *J* = 8.2 Hz, 1H), 3.81 (s, 3H), 2.59 (q, *J* = 7.6 Hz, 2H), 2.11 (br d, *J* = 3.0 Hz, 6H), 2.08–2.03 (m, 3H), 1.77 (br t, *J* = 2.9 Hz, 6H), 1.22 (t, *J* = 7.6 Hz, 3H); ^{13}C NMR ($CDCl_3$, 101 MHz): δ 157.0, 138.4, 136.0, 126.4, 125.7, 111.8, 55.3, 40.8, 37.3, 29.3, 28.5, 16.0. IR (ATR): 2899, 2849, 1494, 1447, 1230, 1179, 1136, 1061, 1037, 1025, 990, 890, 818, 810, 718, 620, 602, 590, 579, 542 cm^{-1}. HRMS (CI+): *m*/*z* [M]$^+$ calculated for $C_{19}H_{26}O$: 270.1984; found: 270.1984.

1-(5-*tert*-Butyl-2-methoxyphenyl)-adamantane (4vd)

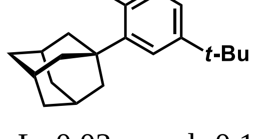

Prepared using General Procedure B with 4-*tert*-butylanisole (32.8 mg, 0.200 mmol, 1 equiv), $FeBr_3$ (17.7 mg, 0.06 mmol, 0.3 equiv), DCE (0.8 mL, 0.25 M), adamantanol (30.6 mg, 0.2 mmol, 1 equiv), and conc. $HBr_{(aq)}$ (2 µL, 0.02 mmol, 0.1 equiv). Purification by preparative TLC (eluting with 19:1 hexanes/EtOAc) afforded **4vd** (31.0 mg, 52%) as a clear oil. R_f: 0.82 (19:1 hexanes/EtOAc). ^{1}H NMR ($CDCl_3$, 400

MHz): δ 7.28 (d, *J* = 2.5 Hz, 1H), 7.19 (dd, *J* = 8.5, 2.5 Hz, 1H), 6.82 (d, *J* = 8.5 Hz, 1H), 3.83 (s, 3H), 2.13 (br d, *J* = 3.0 Hz, 6H), 2.11–2.05 (m, 3H), 1.79 (br t, *J* = 3.1 Hz, 5H), 1.75 (br t, *J* = 3.2 Hz, 1H), 1.33 (s, 9H); ^{13}C NMR ($CDCl_3$, 101 MHz): δ 156.5, 142.6, 137.7, 123.8, 123.1, 111.0, 55.0, 40.7, 37.2, 35.6, 34.3, 31.7, 29.2. IR (ATR): 2902, 2848, 2361, 1604, 1495, 1454, 1361, 1343, 1316, 1291, 1268, 1234, 1202, 1179, 1144, 1123, 1102, 1039, 1028, 978, 907, 884, 818, 809, 795, 731, 677, 666, 633, 574, 558, 547, 539, 530 cm^{-1}. HRMS (ESI+): *m/z* $[M+H]^+$ calculated for $C_{21}H_{31}O$: 299.2369; found: 298.2373.

Ethyl-3-(3-adamantyl-4-methoxyphenyl)propanoate (4wd)

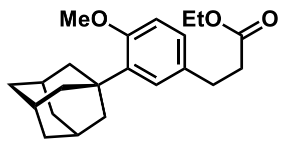

Prepared using General Procedure B with ethyl-3-(4-methoxyphenyl)propanoate (41.7 mg, 0.200 mmol, 1 equiv), $FeBr_3$ (17.7 mg, 0.060 mmol, 0.3 equiv), DCE (0.8 mL, 0.25 M), adamantanol (30.6 mg, 0.20 mmol, 1 equiv), and conc. $HBr_{(aq)}$ (3 µL, 0.03 mmol, 0.15 equiv). Purification by preparative TLC (eluting with 19:1 hexanes/EtOAc) afforded **4wd** (34.2 mg, 50%) as a waxy cream-colored solid. R_f: 0.41 (19:1 hexanes/EtOAc). M.p. 90–91 °C. ^{1}H NMR ($CDCl_3$, 400 MHz): δ 6.96 (d, *J* = 2.2 Hz, 1H), 6.92 (dd, *J* = 8.2, 2.3 Hz, 1H), 6.71 (d, *J* = 8.2 Hz, 1H), 4.06 (q, *J* = 7.1 Hz, 2H), 3.73 (s, 3H), 2.81 (t, *J* = 8.2 Hz, 2H), 2.51 (t, *J* = 8.2 Hz, 2H), 2.06–1.94 (m, 9H), 1.69 (br t, *J* = 3.0 Hz, 6H), 1.17 (t, *J* = 7.1 Hz, 3H); ^{13}C NMR ($CDCl_3$, 101 MHz): δ 173.3, 157.4, 138.6, 132.3, 126.8, 126.3, 111.8, 60.5, 55.2, 40.7, 37.3, 37.0, 36.5, 30.7, 29.2, 14.4. IR (ATR): 3010, 2972, 2901, 2888, 2843, 2366, 1735, 1604, 1491, 1454, 1414, 1368, 1298, 1260, 1200, 1181, 1162, 1147, 1035, 1024, 976, 865, 877, 816, 779, 718, 690, 638, 591, 568, 561, 543, 530 cm^{-1}. HRMS (ESI): *m/z* $[M+H]^+$ calculated for $C_{22}H_{31}O_3$: 343.2268; found: 343.2242.

1-(5-Bromo-2-methoxyphenyl)-adamantane (4z'''d)

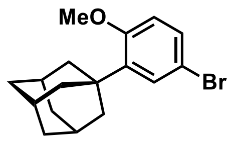

Prepared using General Procedure B with 4-bromoanisole (37.4 mg, 0.200 mmol, 1 equiv), $FeBr_3$ (17.7 mg, 0.060 mmol, 0.3 equiv), DCE (0.8 mL, 0.25 M), adamantanol (30.6 mg, 0.200 mmol, 1 equiv), and conc. $HBr_{(aq)}$ (3 µL, 0.03 mmol, 0.15 equiv). Purification by preparative TLC (eluting with 19:1 hexanes/EtOAc) afforded **4z'''d** (63.6 mg, 99%) as a peach-colored solid. R_f: 0.42 (19:1 hexanes/EtOAc). M.p. 141–142 °C. ^{1}H NMR ($CDCl_3$, 600 MHz): δ 7.28 (d, *J* = 2.5 Hz, 1H), 7.26 (dd, *J* = 8.6, 2.5 Hz, 1H), 6.73 (d, *J* = 8.6 Hz, 1H), 3.81 (s, 3H), 2.05 (br s, 9H), 1.76 (br s, 6H); ^{13}C NMR ($CDCl_3$, 151 MHz): δ 157.9, 140.8, 129.8, 129.3, 113.3, 55.2, 40.3, 37.2, 37.0, 29.0. IR (ATR): 3007, 2898,

2844, 2358, 1598, 1499, 1450, 1438, 1356, 1275, 1260, 1215, 1178, 1145, 1101, 1058, 1037, 1021, 975, 876, 785, 771, 719, 696, 673, 645, 589, 574, 556, 539, 530 cm^{-1}. HRMS (CI+): *m*/*z* [M]$^+$ calculated for $C_{17}H_{21}BrO$: 320.0776; found: 320.0761.

4-*tert*-Butyl-*o*-xylene (5aa)

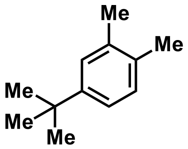

Prepared using General Procedure B with *o*-xylene (21.2 mg, 0.200 mmol, 1 equiv), $FeCl_3$ (9.7 mg, 0.060 mmol, 0.3 equiv), DCE (0.8 mL, 0.25 M), *tert*-butanol (14.8 mg, 0.200 mmol, 1 equiv), and conc. $HCl_{(aq)}$ (14 µL, 0.15 mmol, 0.75 equiv). Purification by preparative TLC (eluting with hexanes) afforded **5aa** (28.5 mg, 88%) as a colorless oil. R_f: 0.73 (hexanes). ^{1}H NMR ($CDCl_3$, 600 MHz): δ 7.19 (s, 1H), 7.16 (d, *J* = 7.8, 1H), 7.13 (d, *J* = 8.4 Hz, 1H), 2.29 (s, 3H), 2.26 (s, 3H), 1.33 (s, 9H); ^{13}C NMR ($CDCl_3$, 151 MHz): δ 148.8, 136.1, 133.7, 129.5, 126.8, 122.8, 34.4, 31.6, 20.2, 19.4. The spectral data recorded are consistent with those previously reported.[13]

1,2-Dimethyl-4-(1-methylcyclohexyl)benzene (5ac)

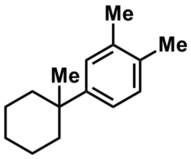

Prepared using General Procedure B with *o*-xylene (21.2 mg, 0.200 mmol, 1 equiv), $FeCl_3$ (9.7 mg, 0.060 mmol, 0.3 equiv), DCE (0.8 mL, 0.25 M), 1-methylcyclohexanol (22.8 mg, 0.200 mmol, 1 equiv), and conc. $HCl_{(aq)}$ (14 µL, 0.15 mmol, 0.75 equiv). Purification by preparative TLC (eluting with hexanes) afforded **5ac** (14.3 mg, 35%) as a yellow oil. R_f: 0.62 (hexanes). ^{1}H NMR ($CDCl_3$, 600 MHz): δ 7.15 (s, 1H), 7.10 (q, *J* = 7.8 Hz, 2H), 2.27 (s, 3H), 2.24 (s, 3H), 1.98 (m, 2H), 1.54 (m, 5H), 1.45 (m, 3H), 1.17 (s, 3H); ^{13}C NMR ($CDCl_3$, 151 MHz): δ 147.7, 136.3, 133.5, 129.6, 127.4, 123.4, 38.1, 37.6, 26.6, 22.9, 20.3, 19.4. IR (ATR): 3020, 2922, 2855, 2361, 1611, 1507, 1467, 1450, 1374, 1306, 1133, 1110, 1020, 995, 964, 928, 910, 880, 733, 719, 610, 594, 547, 534 cm^{-1}. HRMS (CI+): *m*/*z* [M]$^+$ calculated for $C_{15}H_{22}$: 202.1722; found: 202.1713.

1-(1-Adamantyl)-3,4-dimethylbenzene (5ad)

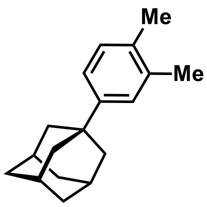

Prepared using General Procedure B with *o*-xylene (21.2 mg, 0.200 mmol, 1 equiv), $FeCl_3$ (9.7 mg, 0.060 mmol, 0.3 equiv), DCE (0.8 mL, 0.25 M), 1-adamantanol (30.4 mg, 0.200 mmol, 1 equiv), and conc. $HCl_{(aq)}$ (14 µL, 0.15 mmol, 0.75 equiv). Purification by preparative TLC (eluting with hexanes) afforded **5ad** (46.5 mg, 97%) as a white solid. R_f: 0.54 (hexanes). M.p. 108–109 °C. ^{1}H NMR

($CDCl_3$, 600 MHz): δ 7.14 (s, 1H), 7.10 (m, 2H), 2.27 (s, 3H), 2.23 (s, 3H), 2.08 (s, 3H), 1.91 (s, 6H), 1.76 (m, 6H); ^{13}C NMR ($CDCl_3$, 151 MHz): δ 149.2, 136.2, 133.7, 129.5, 126.4, 122.3, 43.4, 37.0, 29.1, 20.2, 19.4. IR (ATR): 2999, 2898, 2847, 1617, 1572, 1504, 1445, 1382, 1356, 1342, 1317, 1159, 1126, 1101, 1024, 993, 909, 889, 803, 731, 717, 688, 645, 563, 556, 546, 536 cm^{-1}. HRMS (CI+): *m*/*z* $[M]^+$ calculated for $C_{18}H_{24}$: 240.1878; found: 240.1880.

6-*tert*-Butyl-1,2,3,4-tetrahydronapthalene (5ba)

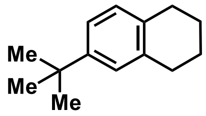

Prepared using General Procedure B with tetralin (26.4 mg, 0.200 mmol, 1 equiv), $FeCl_3$ (9.7 mg, 0.060 mmol, 0.3 equiv), DCE (0.8 mL, 0.25 M), 1-*tert*-butanol (14.8 mg, 0.200 mmol, 1 equiv), and conc. $HCl_{(aq)}$ (14 µL, 0.15 mmol, 0.75 equiv). Purification by preparative TLC (eluting with hexanes) afforded an inseparable mixture (30.9 mg, 51% **5ba** + 20% tetralin) as a yellow oil. R_f: 0.72 (hexanes). ^{1}H NMR ($CDCl_3$, 600 MHz): δ 7.14 (d, *J* = 9.6 Hz, 1H), 7.09 (s, 1H), 7.02 (d, *J* = 9.6 Hz, 1H), 2.75 (m, 4H), 1.80 (s, 4H), 1.31 (s, 9H); ^{13}C NMR ($CDCl_3$, 151 MHz): δ 148.4, 136.7, 134.3, 129.0, 126.0, 122.7, 34.4, 31.6, 29.8, 29.0, 23.5. IR (ATR): 2932, 2859, 2836, 1503, 1477, 1459, 1437, 1411, 1392, 1362, 1270, 1245, 1201, 1189, 1136, 910, 893, 877, 827, 807, 714, 648, 615, 583, 550, 535 cm^{-1}. HRMS (CI+): *m*/*z* $[M]^+$ calculated for $C_{14}H_{20}$: 188.1565; found: 188.1564.

4-(1-Adamantyl)-2-isopropyl-5-methylphenol (12)

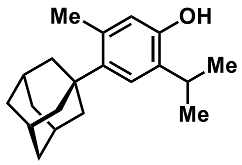

Prepared using General Procedure C with thymol (30.8 mg, 0.205 mmol, 1 equiv), $FeCl_3$ (1.5 mg, 9.2 µmol, 0.05 equiv), chlorobenzene (0.8 mL, 0.25 M), 1-adamantanol (33.3 mg, 0.219 mmol, 1.1 equiv), and conc. $HCl_{(aq)}$ (12.5 µL, 0.15 mmol, 0.75 equiv). Purification by preparative TLC (eluting with 9:1 hexanes/EtOAc) followed by re-subjection to reaction conditions afforded a 2.7:1 mixture of **12** and thymol starting material (25.9 mg, 38%) as a yellow oil. R_f: 0.37 (15:4:1 hexanes/toluene/Et_2O). ^{1}H NMR (600 MHz, $CDCl_3$) δ 7.16 (s, 1H, **12**), 7.10 (d, *J* = 7.8 Hz, 0.3H, **thymol**), 6.75 (d, *J* = 7.8 Hz, 0.3H, **thymol**), 6.59 (s, 0.3H, **thymol**), 6.53 (s, 1H, **12**), 4.71 (br s, 0.3H, **thymol**), 4.58 (br s, 1H, **12**), 3.24–3.09 (m, 1.3H, **12** + **thymol**), 2.54 (s, 3H, **12**), 2.29 (s, 0.9H, **thymol**), 2.13–2.10 (m, 3H, **12**), 2.08 (d, *J* = 2.9 Hz, 6H, **12**), 1.79 (d, *J* = 3.1 Hz, 6H, **12**), 1.28 (d, *J* = 6.9 Hz, 6H, 12**),** 1.26 (d, *J* = 7.1 Hz, 1.8H, **thymol**). ^{13}C NMR (151 MHz, $CDCl_3$) δ 152.6 (**thymol**), 150.2 (**12**), 140.6 (**12**), 136.7 (thymol), 134.8 (**12**), 131.4 (**thymol**), 130.8 (**12**),

126.4 (**thymol**), 124.6 (12), 121.8 (**thymol**), 119.9 (**12**), 116.1 (**thymol**), 41.8 (**12**), 37.7 (**12**), 37.1 (**12**), 29.4 (**12**), 27.4 (**12**), 26.8 (**thymol**), 22.9 (**12**), 22.8 (**thymol**), 21.0 (**thymol**).

6-(1-Adamantyl)benzo[*d*][1,3]dioxol-5-ol (13)

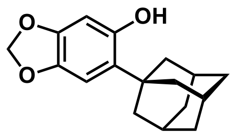

Prepared using General Procedure C with sesamol (34.8 mg, 0.2067 mmol, 1 equiv), $FeCl_3$ (1.6 mg, 0.01 mmol, 0.05 equiv), chlorobenzene (0.8 mL, 0.25 M), 1-adamantanol (33.8 mg, 0.222 mmol, 1.1 equiv), and conc. $HCl_{(aq)}$ (12.5 µL, 0.15 mmol, 0.75 equiv). Purification by flash chromatography (eluting with 5–20% EtOAc in hexanes) afforded **13** (36.7 mg, 65%) as a white solid. R_f: 0.37 (15:4:1 hexanes/toluene/Et_2O). M.p. 197–198 °C. ^1H NMR (500 MHz, $CDCl_3$) δ 6.75 (s, 1H), 6.28 (s, 1H), 5.87 (s, 2H), 4.44 (br s, 1H), 2.06 (s, 9H), 1.76 (s, 6H). ^{13}C NMR (151 MHz, $CDCl_3$) δ 149.0, 145.4, 141.5, 129.0, 107.0, 101.1, 99.4, 41.0, 37.1, 36.6, 29.2. IR (ATR): 3514, 2897, 2850, 1631, 1485, 1380, 1236, 723 cm^{-1}. HRMS (ESI–): *m/z* $[M–H]^-$ calculated for $C_{17}H_{21}O_3$: 273.1485; found: 273.1466.

(8*R*,9*S*,13*S*,14*S*)-2-(*tert*-Butyl)-3-hydroxy-13-methyl-7,8,9,11,12,13,15,16-octahydro-6*H*-cyclopenta[*a*]phenanthren-17(14*H*)-one (14)

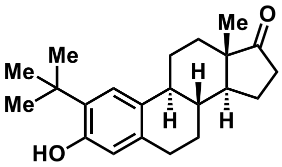

Prepared using General Procedure C with estrone (29.4 mg, 0.109 mmol, 1 equiv), $FeCl_3$ (0.8 mg, 0.005 mmol, 0.05 equiv), chlorobenzene (0.4 mL, 0.25 M), *tert*-butanol (21.0 µL, 0.22 mmol, 2.2 equiv), and conc. $HCl_{(aq)}$ (6.3 µL, 0.075 mmol, 0.75 equiv). Purification by preparative TLC (eluting with 7:3 hexanes/EtOAc) afforded **14** (13.2 mg, 40%) as a white solid. R_f: 0.21 (4:1 hexanes/EtOAc). M.p. 236–238 °C. ^1H NMR (500 MHz, $CDCl_3$) δ 7.20 (s, 1H), 6.43 (s, 1H), 4.62 (s, 1H), 2.89–2.75 (m, 2H), 2.50 (dd, J = 19.1, 8.7 Hz, 1H), 2.47–2.39 (m, 1H), 2.30–2.20 (m, 1H), 2.14 (dt, J = 18.6, 8.9 Hz, 1H), 2.09–2.01 (m, 1H), 2.01–1.91 (m, 2H), 1.67–1.47 (m, 6H), 1.40 (s, 9H), 0.91 (s, 3H). ^{13}C NMR (151 MHz, $CDCl_3$) δ 221.6, 152.3, 135.3, 133.7, 131.4, 124.2, 116.7, 50.5, 48.2, 44.4, 38.6, 36.1, 34.6, 31.8, 29.8, 28.9, 26.7, 26.1, 21.7, 14.0. IR (ATR): 3285, 2920, 2866, 1715, 1611, 1411, 1387, 1202, 825 cm^{-1}. HRMS (ESI–): *m/z* $[M–H]^-$ calculated for $C_{22}H_{31}O_2$: 327.2319; found: 327.2324.

(8*R*,9*S*,13*S*,14*S*)-2-(1-adamantyl)-3-hydroxy-13-methyl-7,8,9,11,12,13,15,16-octahydro-6*H*-cyclopenta[*a*]phenanthren-17(14*H*)-one (15)

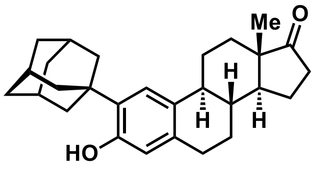

Prepared using General Procedure C with estrone (54.4 mg, 0.201 mmol, 1 equiv), $FeCl_3$ (1.6 mg, 0.01 mmol, 0.05 equiv), chlorobenzene (0.8 mL, 0.25 M), 1-adamantanol (33.7 mg, 0.222 mmol, 1.1 equiv), and conc. $HCl_{(aq)}$ (12.5 µL, 0.15 mmol, 0.75 equiv). Purification by flash chromatography (eluting with 10–20% EtOAc in hexanes) followed by preparative TLC (eluting with 4:1 hexanes/EtOAc) afforded **15** (11.3 mg, 19%) as a white solid. R_f: 0.45 (4:1 hexanes/EtOAc). M.p. 312–313 °C. ^{1}H NMR (500 MHz, $CDCl_3$) δ 7.15 (s, 1H), 6.42 (s, 1H), 4.72 (br s, 1H), 2.89–2.74 (m, 2H), 2.51 (dd, *J* = 19.1, 8.6 Hz, 1H), 2.47–2.40 (m, 1H), 2.26 (td, *J* = 10.9, 4.1 Hz, 1H), 2.20–2.00 (m, 10H), 2.02–1.92 (m, 2H), 1.79–1.74 (m, 6H), 1.67–1.47 (m, 6H), 1.47–1.34 (m, 1H), 0.91 (s, 3H). ^{13}C NMR (151 MHz, $CDCl_3$) δ 221.4, 152.4, 135.1, 134.0, 131.6, 124.1, 116.9, 115.1, 53.6, 50.5, 48.2, 44.4, 40.8, 38.6, 37.2, 36.7, 36.0, 31.7, 29.2, 28.9, 26.6, 26.1, 21.7, 14.0. IR (ATR): 3445, 2901, 2851, 1729, 1616, 1413, 1369, 1211, 825 cm^{-1}. HRMS (ESI–): *m/z* $[M–H]^-$ calculated for $C_{28}H_{36}O_2$: 405.2788; found: 405.2793.

3-(*tert*-Butyl)-1*H*-indole (16aa)

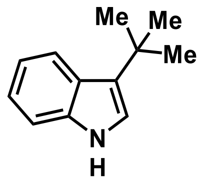

Prepared using General Procedure B with indole (**15a**, 23.4 mg, 0.200 mmol, 1 equiv), $FeBr_3$ (17.7 mg, 0.060 mmol, 0.3 equiv), DCE (0.8 mL, 0.25 M), *tert*-butanol (14.8 mg, 0.200 mmol, 1 equiv), and HBr (3 µL, 0.03 mmol, 0.15 equiv). Purification via 2 cycles of preparative TLC (eluting with 19:1 hexanes/EtOAc) afforded **16aa** (12.9 mg, 37%) as a yellow oil. R_f: 0.58 (19:1 hexanes/EtOAc, then 7:3 hexanes/CH_2Cl_2). ^{1}H NMR ($CDCl_3$, 600 MHz): δ 7.83 (d, *J* = 7.8 Hz, 1H), 7.36 (d, *J* = 7.8 Hz 1H), 7.18 (t, *J* = 7.2 Hz, 1H), 7.10 (t, *J* = 7.2 Hz 1H), 6.94 (s, 1H), 1.47 (s, 9H); ^{13}C NMR ($CDCl_3$, 151 MHz): δ 137.3, 126.9, 126.8, 126.0, 121.6, 121.4, 119.3, 118.9, 111.4, 32.1, 30.9. The spectral data recorded are consistent with those previously reported.[14]

3-(*tert*-Butyl)-1-methyl-1*H*-indole (16ba)

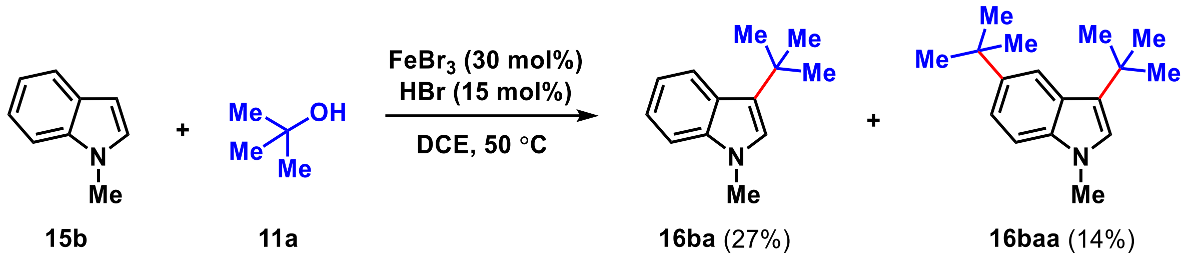

Prepared using General Procedure B with *N*-methylindole (**15b**, 26.2 mg, 0.200 mmol, 1 equiv), $FeBr_3$ (17.7 mg, 0.060 mmol, 0.3 equiv), DCE (0.8 mL, 0.25 M), *tert*-butanol (14.8 mg, 0.200 mmol, 1 equiv), and HBr (3 µL, 0.03 mmol, 0.15 equiv). Purification by preparative TLC (eluting with 19:1 hexanes/EtOAc) afforded **16ba** and **16bb** as a coeluted mixture (16.9 mg, 27% **16ba** + 14% **16baa**) as a yellow oil. R_f: 0.26 (19:1 hexanes/EtOAc). A small amount of pure **16baa** (~2 mg) was obtained by preparative TLC (eluting with 5% CH_2Cl_2 in hexanes × 3 elutions) for characterization purposes.

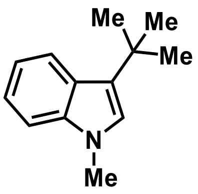

^{1}H NMR ($CDCl_3$, 500 MHz): δ 7.81 (d, *J* = 8.0 Hz, 1H), 7.30 (d, *J* = 8.2 Hz 1H), 7.21 (t, *J* = 7.5 Hz, 1H), 7.10 (t, *J* = 7.5 Hz 1H), 6.80 (s, 1H), 3.74 (s, 3H), 1.46 (s, 9H); ^{13}C NMR ($CDCl_3$, 151 MHz): δ 137.3, 126.9, 126.1, 121.6, 121.4, 119.4, 118.9, 111.4, 32.1, 31.7, 30.9. IR (ATR): 3145, 2952, 2865, 1546, 1482, 1464, 1423, 1390, 1375, 1360, 1327, 1259, 1233, 1202, 1153, 1135, 1110, 1053, 102, 987, 795, 723, 672, 573, 564 cm^{-1}. HRMS (ESI+): *m/z* $[M+H]^+$ calculated for $C_{13}H_{18}N$: 188.1439; found: 188.1437.

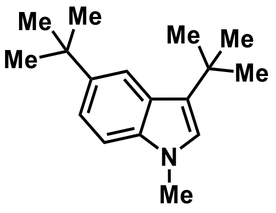

^{1}H NMR ($CDCl_3$, 500 MHz): δ 7.72 (d, *J* = 8.4 Hz, 1H), 7.24 (d, *J* = 1.7 Hz 1H), 7.17 (dd, *J* = 8.4, 1.8 Hz, 1H), 6.73 (s, 1H), 3.72 (s, 3H), 1.43 (s, 9H), 1.40 (s, 9H); ^{13}C NMR ($CDCl_3$, 151 MHz): δ 144.5, 137.9, 125.0, 124.2, 124.1, 121.4, 120.9, 116.6, 105.6, 34.9, 32.6, 32.0, 31.7, 31.1. HRMS (ESI+): *m/z* $[M+H]^+$ calculated for $C_{17}H_{27}N$: 244.2060; found: 244.2067.

1-Bromoadamantane (17)

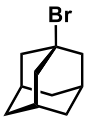

A one-dram vial equipped with a stirring bar was sequentially added $FeBr_3$ (59.1 mg, 0.2 mmol, 1 equiv), 1-adamantanol (30.4 mg, 0.2 mmol, 1 equiv), DCE (0.8 mL, 0.25 M), HBr (10.0 µL, 0.09 mmol, 45 mol%). The reaction mixture was heated at 50 °C for 24 h,

at which time the solution was filtered through a 5" pipette plug of silica gel (approximately half-filled) and eluted with DCM. The solution was concentrated *in vacuo* to yield the product **17** (37.4 mg, 87%) as a white solid. ^{1}H NMR ($CDCl_3$, 400 MHz): δ 2.36 (m, 6H), 2.10 (s, 3H), 1.73 (m, 6H). The spectroscopic data obtained are consistent with the previously reported literature.[15]

(*E*)-2-(*tert*-Butoxyimino)naphthalen-1(2H)-one (22)

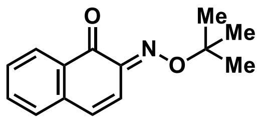

Prepared using General Procedure B with 1-naphthol (34.7 mg, 0.201 mmol, 1 equiv), $FeCl_3$ (3.4 mg, 0.021 mmol, 0.10 equiv), DCE (0.8 mL, 0.25 M), *tert*-butanol (21 µL, 0.22 mmol, 1.1 equiv), and conc. $HCl_{(aq)}$ (37%, 12.5 µL, 0.15 mmol, 0.75 equiv). Purification by preparative TLC (eluting with 4:1 hexanes/EtOAc × 2) afforded **22** (8.2 mg, 18%) as a bright yellow oil. R_f: 0.50 (17:3 hexanes/EtOAc). ^{1}H NMR ($CDCl_3$, 500 MHz): δ = 8.20 (d, *J* = 7.8 Hz, 1H), 7.56 (t, *J* = 7.4 Hz, 1H), 7.41 (t, *J* = 7.6 Hz, 1H), 7.30 (d, *J* = 7.7 Hz, 1H), 7.10 (d, *J* = 10.1 Hz, 1H), 6.81 (d, *J* = 10.0 Hz, 1H), 1.45 (s, 9H); ^{13}C NMR ($CDCl_3$, 151 MHz): δ = 182.1, 147.2, 136.7, 134.3, 132.1, 130.7, 129.0, 128.5, 128.2, 117.1, 83.5, 27.8. IR (ATR): 2977, 1672, 1613, 1592, 1450, 964, 680 cm^{-1}. HRMS (ESI): *m/z* $[M+H]^+$ calculated for $C_{14}H_{16}NO_2$: 230.1176; found: 230.1174.

6. Mechanistic Studies

i) Kinetic Experiments

with DTBP ***2****:*

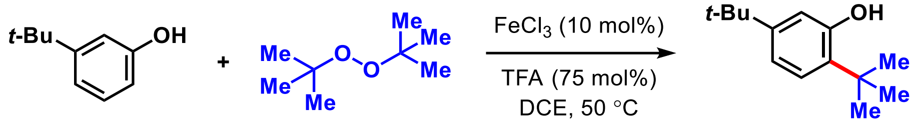

Kinetic experiments were carried out using the system depicted above. 3-*tert*-Butylphenol (**1f**) was chosen because little-to-no side products form over the course of the reaction. No products of decomposition were observed, therefore simplifying the data analysis. The kinetic profile of the reaction was examined using the method of initial rates and by varying the concentrations of **1f**, **2**, $FeCl_3$ catalyst, and TFA. The conversions to product **3fa** were monitored by GC–FID analysis.

Table S1. Kinetic Data for Arene Alkylation with Di-*tert*-Butylperoxide

Entry	[**1f**] / M	[**2**] / M	[$FeCl_3$] / M	[TFA] / M	initial rate[a] / $M \cdot s^{-1}$
1	6.2×10^{-2}	1.3×10^{-1}	1.3×10^{-2}	9.4×10^{-2}	$0.31_8 \times 10^{-4}$
2	$1.2_5 \times 10^{-1}$	1.3×10^{-1}	1.3×10^{-2}	9.4×10^{-2}	$0.85_1 \times 10^{-4}$
3	1.9×10^{-1}	1.3×10^{-1}	1.3×10^{-2}	9.4×10^{-2}	$1.1_3 \times 10^{-4}$
4	2.5×10^{-1}	1.3×10^{-1}	1.3×10^{-2}	9.4×10^{-2}	$1.5_2 \times 10^{-4}$
5	$1.2_5 \times 10^{-1}$	6.5×10^{-2}	1.3×10^{-2}	9.4×10^{-2}	$1.1_5 \times 10^{-4}$
6	$1.2_5 \times 10^{-1}$	1.9×10^{-1}	1.3×10^{-2}	9.4×10^{-2}	$0.66_1 \times 10^{-4}$
7	$1.2_5 \times 10^{-1}$	2.6×10^{-1}	1.3×10^{-2}	9.4×10^{-2}	$0.49_5 \times 10^{-4}$
8	$1.2_5 \times 10^{-1}$	1.3×10^{-1}	6.2×10^{-3}	9.4×10^{-2}	$0.25_0 \times 10^{-4}$
9	$1.2_5 \times 10^{-1}$	1.3×10^{-1}	9.4×10^{-3}	9.4×10^{-2}	$0.61_7 \times 10^{-4}$
10	$1.2_5 \times 10^{-1}$	1.3×10^{-1}	1.3×10^{-2}	4.7×10^{-2}	$0.50_2 \times 10^{-4}$[b]
11	$1.2_5 \times 10^{-1}$	1.3×10^{-1}	1.3×10^{-2}	1.3×10^{-1}	$0.49_5 \times 10^{-4}$[c]
12	$1.2_5 \times 10^{-1}$	1.3×10^{-1}	1.3×10^{-2}	1.9×10^{-1}	$0.50_2 \times 10^{-4}$[b]

[a] Average value from 3 independent experiments. [b] Average value from 4 independent experiments. [c] Average value from 2 independent experiments. See plots on next few pages.

Initial rates when varying [phenolic **1f**]:

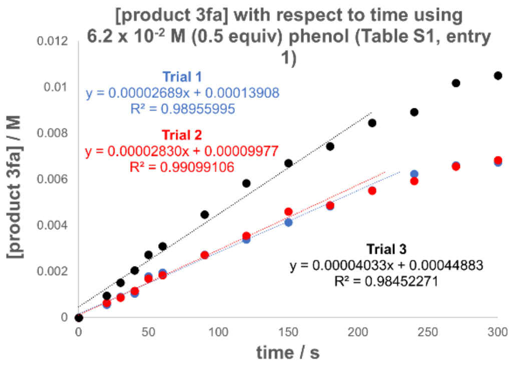

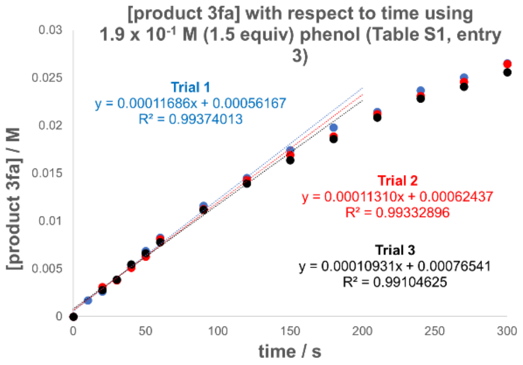

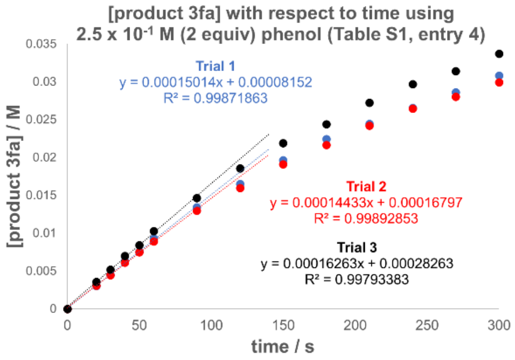

Initial rates when varying [oxidant **2**]:

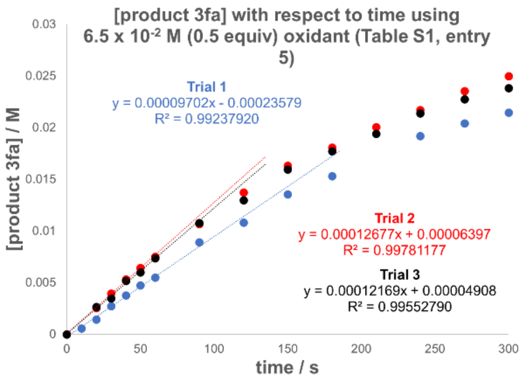

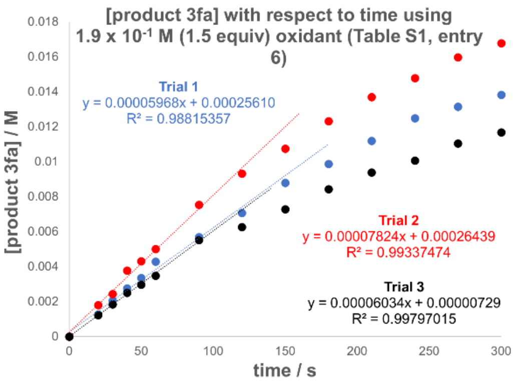

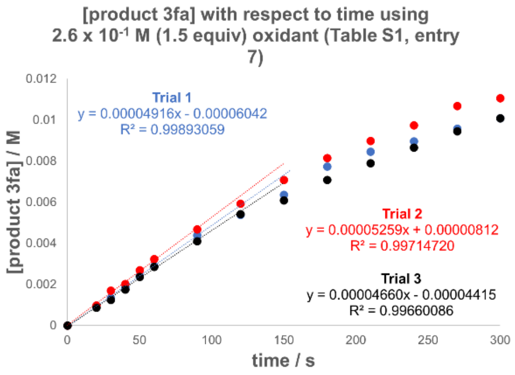

Initial rates when varying [$FeCl_3$]:

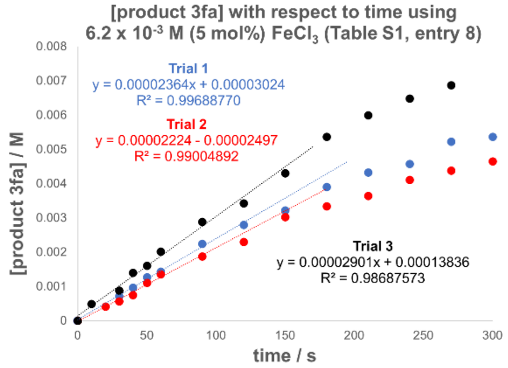

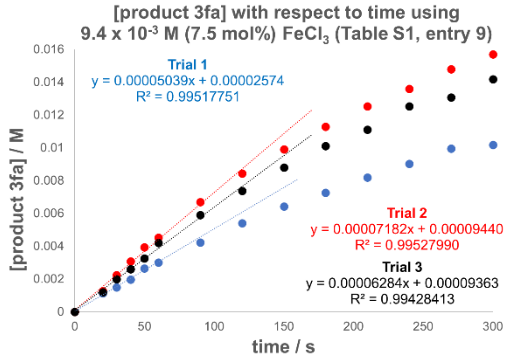

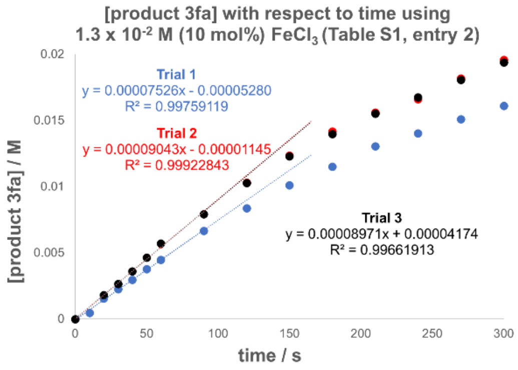

Initial rates when varying [TFA]:

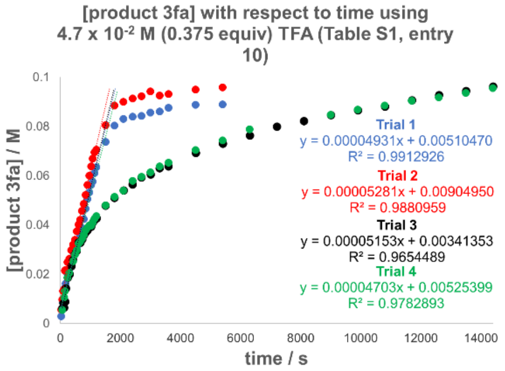

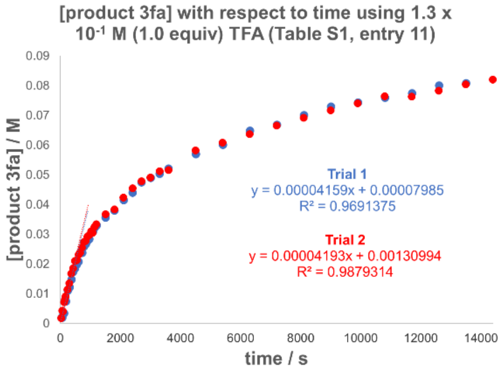

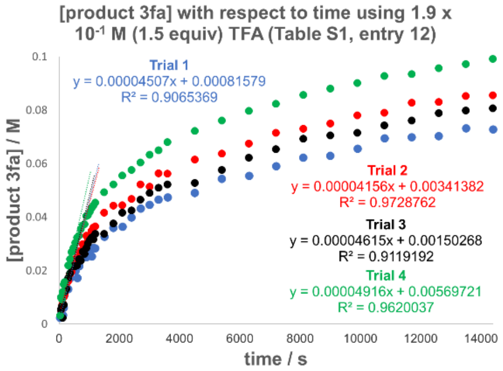

With *tert*-butanol:

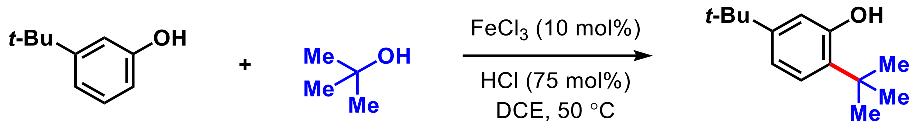

1f **11a** **3fa**

Kinetic experiments were carried out using the system depicted above. 3-*tert*-Butylphenol (**1f**) was chosen because little-to-no side products form over the course of the reaction. No products of decomposition were observed, therefore simplifying the data analysis. The kinetic profile of the reaction was examined using the method of initial and exponential rates and by varying the concentrations of **1f**, **11a**, $FeCl_3$ catalyst, and conc. $HCl_{(aq)}$. The conversions to product **3fa** were monitored by SFC analysis.

While the kinetics for the DTBP (**2**) system were well-behaved, the kinetics data obtained for the alkylation process with *tert*-butanol were all complicated by induction periods. We believe that this may be due to the heterogeneous nature of the reaction mixtures; the iron catalyst does not completely solubilize. The induction periods were characterized by slow reaction rates and lasted for 2000–4000 s (~30–60 min). They did not follow a trend and were unpredictable. Even those induction periods measured within replicate runs differed 2–3 fold in reaction rates (see plots below). We did observe that the acceleration phases following the induction periods all displayed constant reaction rates (apparent zero-order dependences), regardless of varying arene, alcohol, iron, or HCl concentrations.

Table S2. Kinetic Data for Arene Alkylation with *tert*-Butanol

Entry	[**1f**] / M	[**11a**] / M	[$FeCl_3$] / M	[HCl] / M	Induction Period[a] / $M \cdot s^{-1}$	Acceleration Phase[a] / $M \cdot s^{-1}$
1	6.3×10^{-2}	1.3×10^{-1}	1.3×10^{-2}	9.4×10^{-2}	$2.5_5 \times 10^{-6}$	$1.5_9 \times 10^{-5}$
2	1.3×10^{-1}	1.3×10^{-1}	1.3×10^{-2}	9.4×10^{-2}	$5.5_6 \times 10^{-6}$	$3.0_4 \times 10^{-5}$
3	2.5×10^{-1}	1.3×10^{-1}	1.3×10^{-2}	9.4×10^{-2}	$1.9_3 \times 10^{-6}$	$7.4_5 \times 10^{-5}$
4	1.3×10^{-1}	6.3×10^{-2}	1.3×10^{-2}	9.4×10^{-2}	$9.8_3 \times 10^{-6}$	$2.1_0 \times 10^{-5}$
5	1.3×10^{-1}	1.9×10^{-1}	1.3×10^{-2}	9.4×10^{-2}	$4.2_0 \times 10^{-6}$	$2.5_9 \times 10^{-5}$[b]
6	1.3×10^{-1}	2.5×10^{-1}	1.3×10^{-2}	9.4×10^{-2}	$1.0_8 \times 10^{-6}$	$1.3_5 \times 10^{-5}$[b]
7	1.3×10^{-1}	1.3×10^{-1}	3.1×10^{-3}	9.4×10^{-2}	$2.2_3 \times 10^{-6}$	$2.1_6 \times 10^{-5}$
8	1.3×10^{-1}	1.3×10^{-1}	6.3×10^{-3}	9.4×10^{-2}	$1.7_8 \times 10^{-6}$	$1.6_9 \times 10^{-5}$
9	1.3×10^{-1}	1.3×10^{-1}	9.4×10^{-3}	9.4×10^{-2}	$4.3_7 \times 10^{-6}$	$1.7_8 \times 10^{-5}$
10	1.3×10^{-1}	1.3×10^{-1}	1.9×10^{-2}	9.4×10^{-2}	$3.9_3 \times 10^{-6}$	$2.8_5 \times 10^{-5}$[b]
11	1.3×10^{-1}	1.3×10^{-1}	1.3×10^{-2}	4.7×10^{-2}	$18._4 \times 10^{-6}$	$3.3_8 \times 10^{-5}$
12	1.3×10^{-1}	1.3×10^{-1}	1.3×10^{-2}	1.3×10^{-1}	$1.9_2 \times 10^{-6}$	$2.0_7 \times 10^{-5}$
13	1.3×10^{-1}	1.3×10^{-1}	1.3×10^{-2}	1.9×10^{-1}	$1.9_8 \times 10^{-6}$	$2.3_6 \times 10^{-5}$

[a] Average value from 2 independent experiments. [b] Average value from 3 independent experiments. See plots on next few pages.

Rates of induction period and exponential phase when varying [phenolic **1f**]:

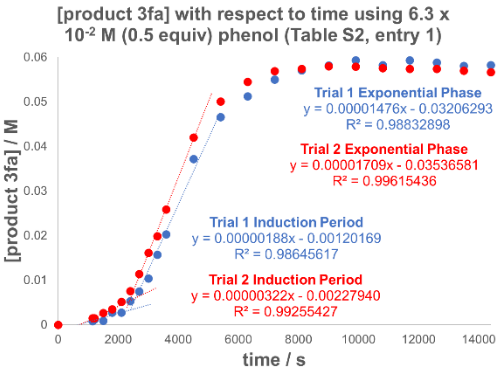

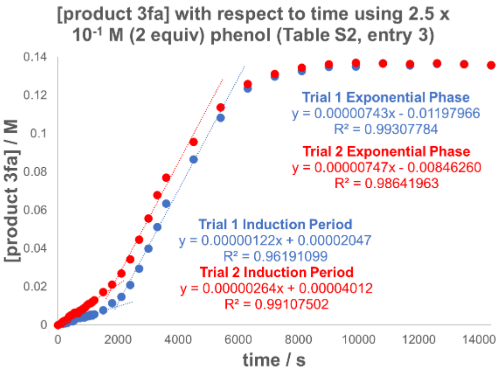

Rates of induction period and exponential phase when varying [alcohol **11a**]:

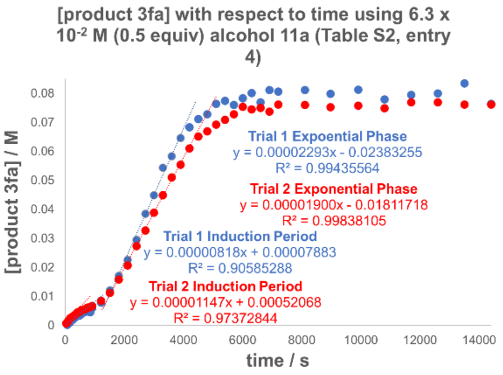

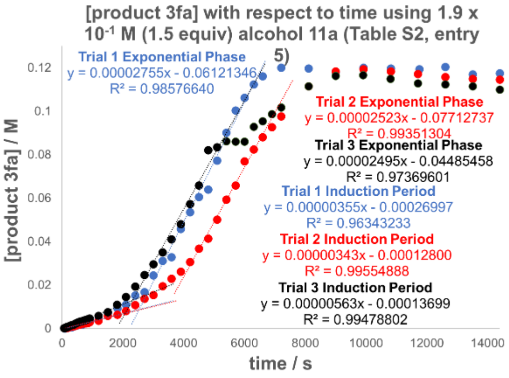

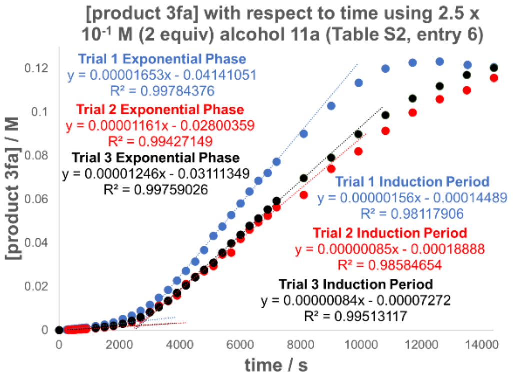

Rates of induction period and exponential phase when varying [$FeCl_3$]:

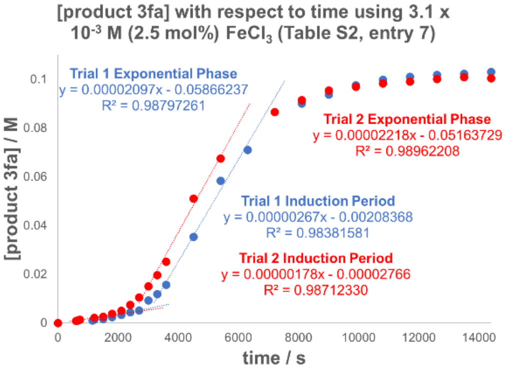

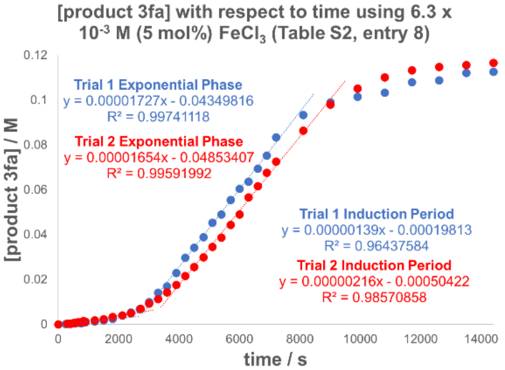

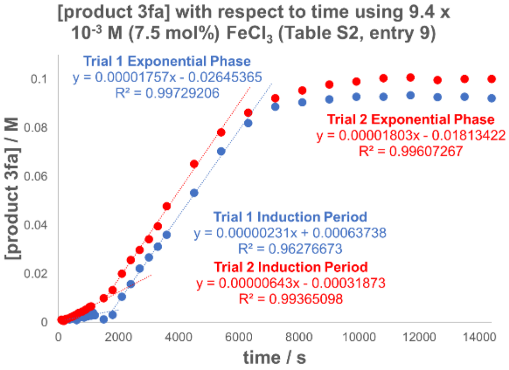

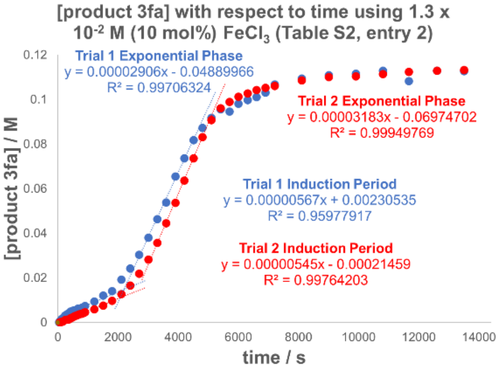

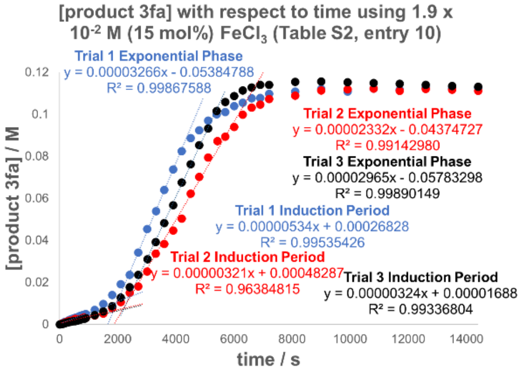

Rates of induction period and exponential phase when varying [HCl]:

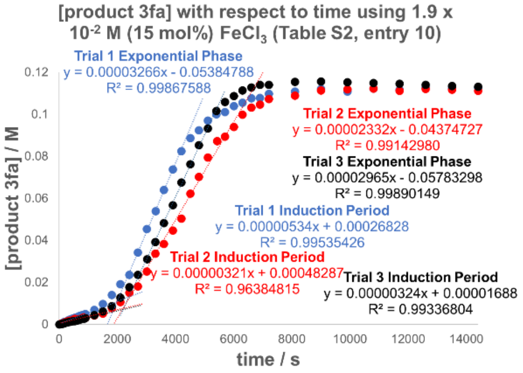

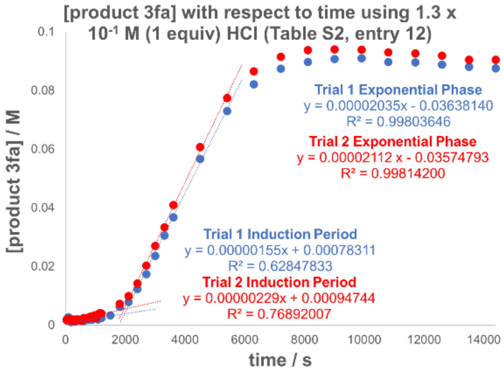

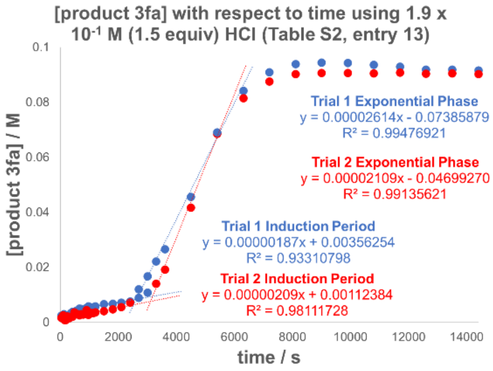

ii) Michael acceptors used to probe formation of radical intermediates

Referring to Scheme 8b, a panel of Michael acceptors **13a–13j** were tested to trap putative radical intermediates that could arise from tertiary alcohol **11d** or tertiary alkyl bromide **12** in the presence of $FeBr_2$. In all cases, Michael addition products were not observed with or without addition of a hydrogen atom donor (*e.g.*, Hantzsch ester). The lack of reactivity suggests that $FeBr_2$ is largely incapable of reducing **11d** or **12** through single electron transfer pathways.

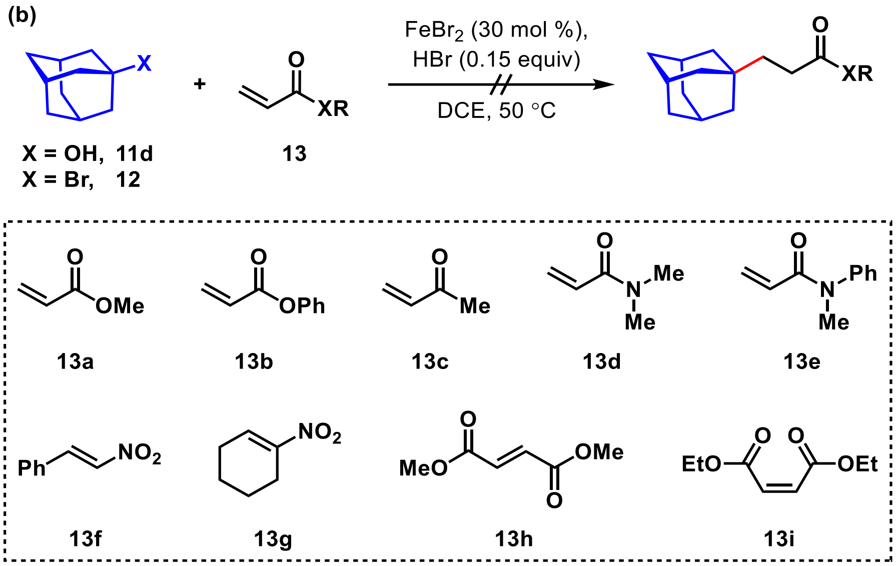

iii) Computational Details

The Gaussian 16 suite of programs[16] was used for the described calculations. Geometry optimizations and frequency calculations for all reported structures were performed using unrestricted density functional theory (DFT) at the M15L/def2-svp level of theory in the gas phase (E_{opt}). The identity of each optimized structure as a minimum energy structure is confirmed by inspecting vibrational analysis for imaginary frequencies. The energies are refined with the double hybrid density functional B2PLYP[17] with Grimme's D3 empirical dispersion correction with Becke-Johnson damping[18,19] and the def2-TZVPPD basis set[20,21] (obtained from the basis set exchange[22]). Solvent effects are incorporated with the self-consistent reaction field polarizable continuum model (IEF-PCM) in 1,2-dichloroethane (DCE) as the solvent.[23] The Goodvibes program[24] is used to calculate the thermal corrections for the enthalpy and free energy with the quasi-harmonic approximation described by Grimme[25] and to a standard state of 1M. (Any gas phase energies referenced are calculated in the standard state of 1 atm.) Thus, the final reported energies are calculated by adding the corrected thermal corrections (calculated at the M15L/def2-svp level of theory) to the B2PLYPD3/def2-TZVPPD single point energies (E_{SP}). The methods used here have been shown to be accurate in reproducing bond dissociation energies for 3d transition metals.[26]

In addition, gas phase acidities and bond dissociation energies were computed with the selected methods as shown in Tables X and X. The computational methods are overall able to adequately reproduce the experimental values for the examined parameters. Of particular interest to this study,

the computed BDEs for Cl_2Fe–Cl and Br_2Fe–Br are overestimated by approximately 10 kcal·mol^{-1}.

Table X. Comparison gas phase acidities computed at the B2PLYPD3/def2-TZVPPD// M15L/def2-SVP level of theory with experimental values.

Molecule	Exptl.[27] (kcal·mol^{-1})	Computed (kcal·mol^{-1})
HCl	332.5	**332.5**
HBr	322.6	**322.9**

Table X. Comparison selected bond dissociation enthalpies computed at the B2PLYPD3/def2-TZVPPD// M15L/def2-SVP level of theory (gas phase) with experimental values.

Molecule	Exptl. (kcal·mol^{-1})	Computed (kcal·mol^{-1})
H–Cl	103.15[28]	114.0
H–Br	87.59[28]	89.6
Cl_2Fe–Cl	54 ± 2[29]	64.3
Br_2Fe–Br	45 ± 5[29]	58.1
tBu–OH	95.8[28]	92.2

XYZ coordinates and energies in hartrees.

Cl atom
E_{opt} -459.916900
E_{SP} -460.052063
H_{corr} 0.002361
G_{corr} -0.012658
Cl -5.508006 -0.454545 0.000000

Br atom
E_{opt} -2573.850375
E_{SP} -2573.751925
H_{corr} 0.002361
G_{corr} -0.013811
Br -5.508006 -0.454545 0.000000

H atom
E_{opt} -0.494147
E_{SP} -0.498630
H_{corr} 0.002361
G_{corr} -0.007635
H -5.508006 -0.454545 0.000000

HCl
E_{opt} -460.582564
E_{SP} -460.739517
H_{corr} 0.010294
G_{corr} -0.007875
Cl -5.513793 -0.454545 0.000000
H -6.797187 -0.454545 0.000000

HBr
E_{opt} -2574.493609
E_{SP} -2574.398999
H_{corr} 0.009478
G_{corr} -0.010026
Br -5.446116 -0.454545 0.000000
H -6.864864 -0.454545 0.000000

$FeCl_3$ (sextet)
E_{opt} -2643.594090
E_{SP} -2644.121785
H_{corr} 0.009761
G_{corr} -0.027791
Fe 0.000000 -0.000248 0.001677
Cl -0.000000 0.002699 -2.159286
Cl -0.000000 1.870341 1.083640
Cl -0.000000 -1.872791 1.079772

$FeCl_2$ (quintet)
E_{opt} -2183.551791
E_{SP} -2183.981145
H_{corr} 0.007015
G_{corr} -0.023512
Fe 0.000000 0.653150 -0.422176
Cl -0.000000 -0.338618 -2.342417
Cl -0.000000 1.644924 1.498084

$FeBr_3$ (sextet)
E_{opt} -8985.334948
E_{SP} -8985.115177
H_{corr} 0.008835
G_{corr} -0.032011

Fe	-0.000000	0.011789	-0.003442
Br	0.000000	2.001383	1.149834
Br	0.000000	0.015740	-2.303010
Br	0.000000	-1.981779	1.142958

$FeBr_2$ (quintet)
E_{opt} -6411.364390
E_{SP} -6411.298521
H_{corr} 0.006566
G_{corr} -0.026374

Fe	0.000000	0.776347	-0.477290
Br	0.000000	1.835530	1.572107
Br	0.000000	-0.283055	-2.527112

OH radical
E_{opt} -75.580521
E_{SP} -75.730145
H_{corr} 0.012011
G_{corr} -0.005208

O	-0.997697	0.446429	0.000000
H	-0.019344	0.446429	0.000000

tBu radical
E_{opt} -157.473924
E_{SP} -157.719748
H_{corr} 0.123240
G_{corr} 0.089740

C	-0.579733	-0.845477	0.289530
C	0.063560	0.064111	1.291195
H	1.170775	0.069136	1.191296
H	-0.301128	1.109591	1.191807
H	-0.153815	-0.244007	2.347206
C	0.063220	-2.167983	0.002995
H	-0.154620	-2.928236	0.797919
C	0.063220	-2.167983	0.002995
H	-0.154620	-2.928236	0.797919
H	-0.301382	-2.604719	-0.952116
H	1.170470	-2.084341	-0.051048
C	-2.040729	-0.679619	0.002340
H	-2.330697	0.392268	-0.051320
H	-2.330517	-1.168424	-0.953182
H	-2.685446	-1.138709	0.796652

tBuOH
E_{opt} -233.213046
E_{SP} -233.604252
H_{corr} 0.142958
G_{corr} 0.109460

O	-2.241436	0.527907	-0.147989
C	-2.746841	1.855828	-0.014642
C	-2.260621	2.332466	1.357377
H	-1.151965	2.306170	1.394051
H	-2.603560	3.367369	1.562336
H	-2.650158	1.660141	2.149463
C	-4.283463	1.831250	-0.072589
H	-4.715432	2.845103	0.065852
H	-4.626885	1.448162	-1.059362
H	-4.681906	1.161500	0.717546
C	-2.175403	2.740456	-1.135387
H	-2.503386	2.364209	-2.130005
H	-2.517755	3.792948	-1.041961
H	-1.066187	2.720955	-1.105413
H	-2.534193	0.198690	-1.010518

Cl^-
E_{opt} -460.014177
E_{SP} -460.302253
H_{corr} 0.002361
G_{corr} -0.012003

Cl	-5.508006	-0.454545	0.000000

Br^-
E_{opt} -2573.951737
E_{SP} -2573.971831
H_{corr} 0.002361
G_{corr} -0.013157

Br	-5.508006	-0.454545	0.000000

H^+
E_{opt} 0.000000
E_{SP} -0.150228
H_{corr} 0.002361
G_{corr} -0.006981

H	-5.508006	-0.454545	0.000000

$[FeCl_3OH]^-$ (sextet)
E_{opt} -2719.411797
E_{SP} -2720.139735
H_{corr} 0.024465
G_{corr} -0.017602

Fe	-3.414478	0.018701	-0.017075
Cl	-2.104241	-0.198033	1.814256
Cl	-4.922334	-1.685532	-0.152744
Cl	-4.457537	2.025567	0.020589
O	-2.438700	-0.201950	-1.569849
H	-2.803314	-0.960772	-2.048133

$FeCl_2OH$ (sextet)
E_{opt} -2259.260299
E_{SP} -2259.786322
H_{corr} 0.021902
G_{corr} -0.015451

Fe	0.095703	-0.016875	-0.126045
Cl	-0.214717	-0.053708	-2.276056
Cl	0.043567	-1.816584	1.097952
O	0.405073	1.552071	0.662304
H	0.561680	1.725974	1.600190

tBuOH$_2^+$
E_{opt} -233.533709
E_{SP} -234.001880
H_{corr} 0.155059
G_{corr} 0.119812

O	-2.202615	0.448807	-0.119623
C	-2.773607	2.023354	0.028211
C	-2.278161	2.384756	1.409694
H	-1.169931	2.406105	1.463279
H	-2.634687	3.411582	1.633731
H	-2.687007	1.703288	2.181111
C	-4.264574	1.805328	-0.090229
H	-4.756574	2.792665	0.031368
H	-4.551825	1.419546	-1.090304
H	-4.643085	1.132851	0.704215
C	-2.128080	2.772971	-1.116315
H	-2.436566	2.376270	-2.105242
H	-2.474721	3.826669	-1.064498
H	-1.021760	2.789168	-1.036917
H	-2.454862	0.040257	-0.972343
H	-1.228040	0.398197	-0.046689

tBu$^+$
E_{opt} -157.232735
E_{SP} -157.552042
H_{corr} 0.122956
G_{corr} 0.090189

C	-0.588996	0.168817	0.033646
C	-2.037503	0.180303	0.280583
H	-2.402845	1.144258	0.688125
H	-2.483312	0.110636	-0.747266
H	-2.407132	-0.697891	0.843663
C	0.120872	1.435551	-0.178711
H	-0.528718	2.259221	-0.535658
H	0.428675	1.722119	0.863137
H	1.061479	1.333696	-0.755098
C	0.149592	-1.099307	-0.044558
H	-0.322853	-1.935539	0.504684
H	0.113403	-1.363830	-1.134692
H	1.229115	-0.985310	0.182142

$FeCl_3OH_2$ (sextet)
E_{opt} -2719.911379
E_{SP} -2720.573816
H_{corr} 0.037756
G_{corr} -0.005233

Fe	-3.599532	-0.037032	-0.239379
Cl	-4.896020	-1.710810	0.355803
Cl	-4.467414	1.954683	-0.277351
Cl	-1.949745	-0.465130	-1.629567
O	-2.362412	-0.199759	1.493516
H	-2.673003	-0.942243	2.039258
H	-1.457622	-0.426004	1.218261

$[FeCl_2OH_2]^+$ (sextet)
E_{opt} -2259.571047
E_{SP} -2260.188658
H_{corr} 0.034531
G_{corr} -0.004210

Fe	0.030198	-0.289991	-0.083060
Cl	-0.113131	0.038706	-2.169377
Cl	0.377646	-1.998913	1.117041
O	-0.214552	1.403092	1.004428
H	-0.184566	1.473400	1.976642
H	-0.382672	2.296910	0.652438

$FeCl_3$--HCl (sextet)
E_{opt} -3104.199140
E_{SP} -3104.865110
H_{corr} 0.020970
G_{corr} -0.023158

Fe	-0.086103	0.060715	-0.152231
Cl	-1.540628	-1.272917	-1.123271
Cl	-0.410676	0.583904	1.927130
Cl	1.014309	1.450680	-1.400999
Cl	1.635279	-1.976526	0.056430
H	0.725627	-2.699177	-0.518162

$FeCl_3$---tBuOH (sextet)
E_{opt} -2876.869752
E_{SP} -2877.761904
H_{corr} 0.153983
G_{corr} 0.100897

Fe	0.067238	0.059441	-0.087024
Cl	-1.866962	-0.345291	-1.067712
Cl	-0.076040	0.818966	1.962374
Cl	1.647522	0.872863	-1.388779
O	1.007651	-1.776992	0.066255
C	0.483245	-3.088718	0.491850
H	1.705836	-1.879991	-0.604819
C	-0.162876	-3.752536	-0.725337
H	-0.596447	-4.733195	-0.441030
H	-0.968534	-3.107973	-1.131938
H	0.591495	-3.931739	-1.521923
C	-0.530431	-2.770080	1.589876
H	-0.958101	-3.713712	1.983983
H	-0.044218	-2.217823	2.419890
H	-1.363859	-2.152822	1.190833
C	1.676717	-3.882049	1.026891
H	2.152194	-3.345583	1.872940
H	1.344331	-4.880185	1.378024
H	2.435442	-4.039466	0.229600

tBuOH---HCl
E_{opt} -693.812256
E_{SP} -694.3539392
H_{corr} 0.154887
G_{corr} 0.114892

O	-2.238255	0.466288	0.196673
C	-2.708035	1.829514	0.133626
C	-2.374161	2.429412	1.502305

H	-1.278820	2.400225	1.677734
H	-2.714615	3.483405	1.556534
H	-2.876140	1.848711	2.303491
C	-4.222715	1.813262	-0.112313
H	-4.635394	2.843499	-0.134438
H	-4.454132	1.334680	-1.089717
H	-4.734159	1.240177	0.688378
C	-1.968464	2.567549	-0.992285
H	-2.188337	2.096098	-1.975947
H	-2.283425	3.630521	-1.048947
H	-0.872696	2.528818	-0.820214
H	-2.435184	0.051597	-0.657848
H	-0.342016	0.494467	0.523916
Cl	0.933601	0.722735	0.676495

$tBuOH_2^+$---$FeCl_4^-$ (sextet)

E_{opt} -3337.467012
E_{SP} -3338.519461
H_{corr} 0.168217
G_{corr} 0.110506

O	-1.825138	0.435582	0.507969
C	-2.229757	1.891520	0.178721
C	-1.629550	2.758528	1.279347
C	-2.229757	1.891520	0.178721
C	-1.629550	2.758528	1.279347
H	-0.521388	2.733536	1.252749
H	-1.962046	3.803940	1.119204
H	-1.981037	2.434254	2.281695
C	-3.752671	1.855790	0.200489
H	-4.140512	2.869153	-0.027670
H	-4.142711	1.150335	-0.560777
H	-4.127036	1.552503	1.199083
C	-1.651881	2.192368	-1.199362
H	-2.017774	1.465328	-1.955001
H	-1.987490	3.204110	-1.504085
H	-0.543282	2.178931	-1.183301
H	-1.246176	0.029848	-0.208587
H	-1.230297	0.379227	1.318034
Cl	0.469264	-0.651531	-1.117532
Fe	1.810173	0.287287	0.504145
Cl	2.036981	2.440094	0.013453
Cl	0.506334	0.148826	2.398460
Cl	3.686539	-0.792071	0.728590

H_2O

E_{opt} -76.267713
E_{SP} -76.427595
H_{corr} 0.025690
G_{corr} 0.006613

O	0.078464	-0.216318	0.000000
H	1.042398	-0.157230	0.000000
H	-0.187610	0.712050	0.000000

$FeCl_4$ (sextet)

E_{opt} -3103.760047
E_{SP} -3104.484277
H_{corr} 0.012361
G_{corr} -0.029606

Fe	-3.403275	-0.131926	-0.006954
Cl	-2.147513	-0.163806	1.852179
Cl	-4.974780	-1.728334	0.125284
Cl	-4.389901	1.873221	-0.210047
Cl	-2.100131	-0.508977	-1.794444

$FeBr_3$---HBr (sextet)

E_{opt} -11559.847779
E_{SP} -11559.520920
H_{corr} 0.018857
G_{corr} -0.029533

Fe	-0.227855	0.001698	-0.406253
Br	-0.379621	-0.101779	1.918409
Br	1.125600	1.625853	-1.340538
Br	-2.037355	-0.725540	-1.647202
Br	1.475752	-2.265451	-0.585237
H	1.406710	-2.381652	0.835234

$FeBr_4^-$ (sextet)

E_{opt} -11559.414438
E_{SP} -11559.142540
H_{corr} 0.010943
G_{corr} -0.035179

Fe	0.000267	0.000331	0.000381
Br	0.846490	-0.970423	-2.014228
Br	-1.098390	2.061937	-0.509608
Br	-1.557830	-1.503054	1.015066
Br	1.809449	0.411219	1.508426

$FeCl_3$---CF_3COOH (sextet)

E_{opt} -3169.526755
E_{SP} -3170.918958
H_{corr} 0.057285
G_{corr} 0.002603

Fe	-3.612667	-0.362195	-0.364809
Cl	-4.644487	-2.248487	-0.123988
Cl	-4.657448	1.565692	0.004015
Cl	-4.644487	-2.248487	-0.123988
Cl	-4.657448	1.565692	0.004015
Cl	-2.149746	-0.037141	-1.976170
O	-2.208650	-0.103687	1.177621
C	-1.477077	0.870381	1.330906
O	-1.507637	1.998029	0.691967
C	-0.352363	0.851988	2.402264
F	-0.296790	-0.327509	2.985482
F	-0.608188	1.787169	3.310174
F	0.810226	1.120074	1.822161
H	-2.262258	2.034290	0.056125

CF_3COOH

E_{opt} -525.884537
E_{SP} -526.778197
H_{corr} 0.046941
G_{corr} 0.012469

H -0.586909 0.574049 0.741624
O 0.338431 0.712474 1.014559
C 0.389034 0.601120 2.341767
O -0.528905 0.371545 3.080251
C 1.849879 0.818700 2.815199
F 2.268879 2.033560 2.459350
F 2.653622 -0.082307 2.249742
F 1.928791 0.702930 4.129064

CF_3COO^-
E_{opt} -525.345472
E_{SP} -526.328684
H_{corr} 0.033238
G_{corr} -0.001091
O 0.311315 0.695688 1.017995
C 0.349644 0.598826 2.258715
O -0.509495 0.376375 3.128503
C 1.817711 0.813308 2.805934
F 2.304862 2.039062 2.487913
F 2.695593 -0.074238 2.275535
F 1.950912 0.703314 4.145687

$[FeCl_3\text{---}CF_3COO]^-$ (sextet)
E_{opt} -3169.058912
E_{SP} -3170.509344
H_{corr} 0.043998
G_{corr} -0.010318
Fe -3.515924 -0.170494 -0.215987
Cl -4.730152 -1.985013 0.251910
Cl -4.772541 1.664840 -0.134265
Cl -2.397685 -0.439187 -2.121898
O -2.204467 -0.055355 1.240220
C -1.365818 0.926042 1.210163
O -1.254440 1.817765 0.391565
C -0.410546 0.866883 2.436960
F 0.280619 -0.282593 2.456866
F -1.096935 0.938027 3.587270
F 0.472461 1.867294 2.432806

$BF_3\text{---}HF$
E_{opt} -424.277967
E_{SP} -425.015798
H_{corr} 0.031342
G_{corr} -0.00268
B 0.568418 -0.369898 0.448115
F -0.076797 0.775662 0.550506
F 1.900292 -0.369898 0.430432
F -0.076797 -1.515458 0.550506
F 0.653020 -0.369898 -1.822965
H 1.573271 -0.369898 -1.909926

HF
E_{opt} -100.253151
E_{SP} -100.450575
H_{corr} 0.012987
G_{corr} -0.003697
F -5.427023 -0.454545 0.000000
H -6.345209 -0.454545 0.000000

BF_3
E_{opt} -324.010377
E_{SP} -324.562406
H_{corr} 0.017120
G_{corr} -0.010437
B -5.969077 -0.821557 -0.000000
F -6.627618 0.319002 0.000000
F -6.627618 -1.962117 0.000000
F -4.652042 -0.821557 0.000000

F^-
E_{opt} -99.595539
E_{SP} -99.972367
H_{corr} 0.002361
G_{corr} -0.01114
F 1.052296 -0.153061 0.000000

BF_4^-
E_{opt} -423.796399
E_{SP} -424.622161
H_{corr} 0.019941
G_{corr} -0.009897
B 0.618655 -0.369898 -0.000009
F -0.046368 0.781884 0.470187
F 1.948649 -0.369898 0.470286
F -0.046368 -1.521680 0.470187
F 0.618544 -0.369898 -1.410650

7. X-Ray Data

X-ray crystallographic data for phenolic **3fa** (or **kk3mc**, along with its .cif file) is provided with this supporting information.

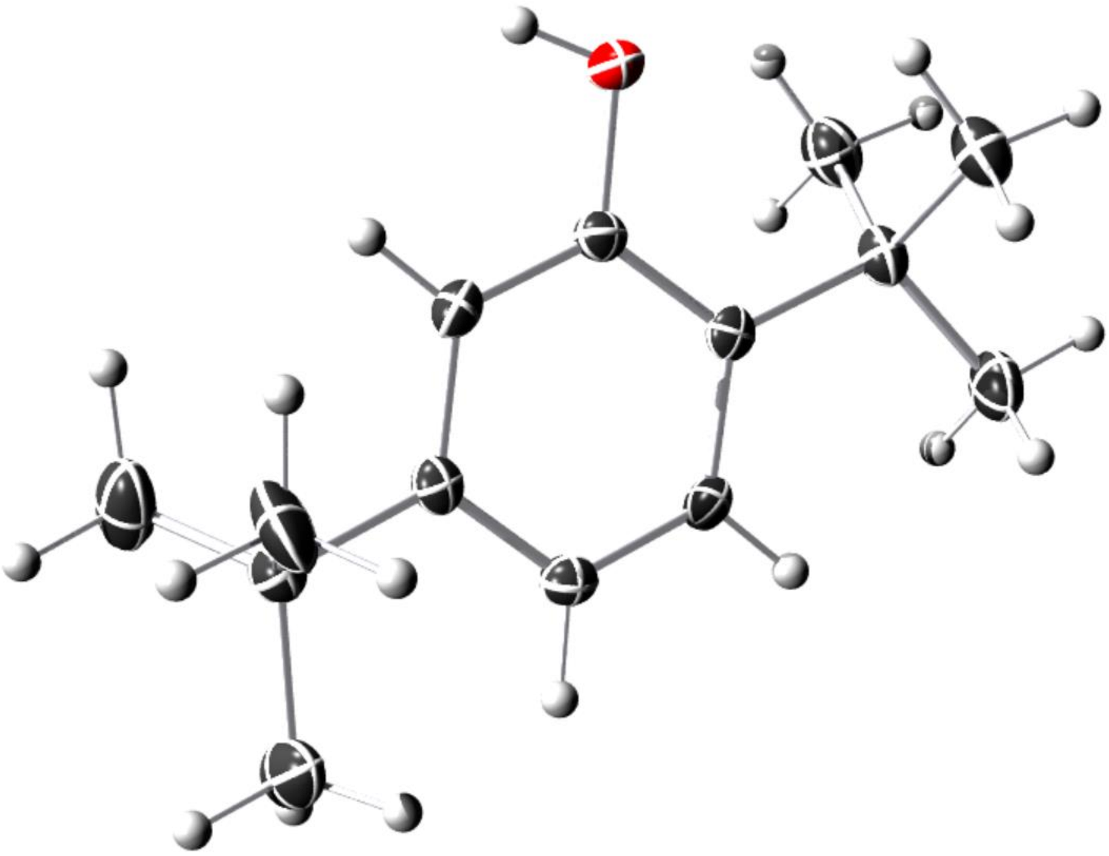

Figure S1. POV-Ray/ORTEP rendering of phenolic **3fa** with ellipsoids at 50% probability.

This crystal structure has been deposited at the Cambridge Crystallographic Data Center under CCDC 2045362.

Diffraction data were collected on a Bruker-AXS Apex II diffractometer with an Apex II CCD detector using Mo K_α radiation (λ = 0.71073 Å) from a fine-focus sealed tube source. Data were collected at 100 K by performing 0.5° φ- and ω-scans, integrated using SAINT[30], and absorption corrected using SADABS[31]. The structure was solved by direct methods using SHELXT[32] and refined against F^2 on all data by full-matrix least squares with SHELXL-2018/3[33] following established refinement strategies[34]. All non-hydrogen atoms were refined anisotropically. Except where specified below for the phenolic hydrogen, all hydrogen atoms were included into the model at geometrically calculated positions and refined using a riding model. The isotropic displacement parameters of all hydrogen atoms were fixed to 1.2 times the U value of the atoms they are linked to (1.5 times for methyl groups). Crystal and data quality details, as well as a summary of the residual refinement values, are listed in the accompanying table.

Compound **kk3mc** crystallizes in the tetragonal chiral space group $P4_1$ with one molecule of **kk3mc** per asymmetric unit. The enantiomorphic space group, $P4_3$, is equally valid and cannot be ruled out, as there was insufficient anomalous signal for this compound under molybdenum radiation.

The phenolic hydrogen, which is oriented toward the pi-system of the neighboring aromatic ring, was located in the Fourier synthesis and refined freely.

One ligand *tert*-butyl group exhibited disorder that was modeled over two positions; the disorder ratio was refined freely and converged at 87:13. This disorder was refined with the help of similarity restraints on 1,2- and 1,3- distances. The minor position of the disorder is in a roughly staggered orientation with respect to the major position, and the anisotropic displacement parameters of opposing carbon atoms were constrained to be equivalent.

Table 1. Crystal data and structure refinement for kk3mc.

Identification code	kk3mc	
Empirical formula	C14 H22 O	
Formula weight	206.31	
Temperature	100(2) K	
Wavelength	0.71073 Å	
Crystal system	Tetragonal	
Space group	$P4_1$	
Unit cell dimensions	a = 10.0087(2) Å	α = 90°.
	b = 10.0087(2) Å	β = 90°.
	c = 12.7456(4) Å	γ = 90°.
Volume	1276.78(6) $Å^3$	
Z	4	
Density (calculated)	1.073 Mg/m^3	
Absorption coefficient	0.065 mm^{-1}	
F(000)	456	
Crystal color	colourless	
Crystal size	0.283 x 0.034 x 0.024 mm^3	
Theta range for data collection	2.035 to 26.367°	
Index ranges	-12 <= *h* <= 12, -12 <= *k* <= 12, -15 <= *l* <= 15	
Reflections collected	12704	
Independent reflections	2616 [R(int) = 0.0669]	
Completeness to theta = 25.242°	100.0 %	
Absorption correction	Semi-empirical from equivalents	
Refinement method	Full-matrix least-squares on F^2	

Data / restraints / parameters	2616 / 7 / 156
Goodness-of-fit on F^2	1.022
Final R indices [I>2sigma(I) = 2044 data]	R1 = 0.0459, wR2 = 0.0942
R indices (all data, 0.80 Å)	R1 = 0.0690, wR2 = 0.1022
Absolute structure parameter	-1.4(10)
Largest diff. peak and hole	0.142 and -0.181 e.$Å^{-3}$

Table 2. Atomic coordinates (x 10^4) and equivalent isotropic displacement parameters ($Å^2$ x 10^3) for kk3mc. U(eq) is defined as one third of the trace of the orthogonalized U^{ij} tensor.

	x	y	z	U(eq)
O(1)	5888(2)	4775(2)	6563(2)	28(1)
C(1)	6760(3)	5171(3)	5794(2)	19(1)
C(2)	7844(3)	4332(3)	5559(2)	17(1)
C(3)	8683(3)	4796(3)	4770(2)	18(1)
C(4)	8486(3)	5984(3)	4238(2)	20(1)
C(5)	7390(3)	6792(3)	4460(2)	19(1)
C(6)	6543(3)	6365(3)	5255(2)	20(1)
C(7)	8068(3)	2997(3)	6124(2)	22(1)
C(8)	9327(3)	2296(3)	5720(3)	32(1)
C(9)	8234(3)	3220(3)	7304(2)	31(1)
C(10)	6872(3)	2066(3)	5906(3)	29(1)
C(11)	7132(3)	8071(3)	3834(2)	25(1)
C(12)	6957(5)	7750(4)	2690(3)	41(1)
C(13)	5906(4)	8842(4)	4234(3)	35(1)
C(14)	8344(4)	9021(4)	3975(3)	41(1)
C(12A)	6960(30)	9190(20)	4526(17)	41(1)
C(13A)	8190(20)	8280(20)	2955(18)	35(1)
C(14A)	5780(20)	7750(20)	3200(20)	41(1)

Table 3. Bond lengths [Å] and angles [°] for kk3mc.

O(1)-C(1)	1.371(3)
O(1)-H(1)	0.85(5)
C(1)-C(6)	1.396(4)
C(1)-C(2)	1.404(4)
C(2)-C(3)	1.390(4)
C(2)-C(7)	1.534(4)
C(3)-C(4)	1.382(4)
C(3)-H(3)	0.9500
C(4)-C(5)	1.392(4)
C(4)-H(4)	0.9500
C(5)-C(6)	1.388(4)
C(5)-C(11)	1.530(4)
C(6)-H(6)	0.9500
C(7)-C(9)	1.529(4)
C(7)-C(8)	1.532(4)
C(7)-C(10)	1.542(4)
C(8)-H(8A)	0.9800
C(8)-H(8B)	0.9800
C(8)-H(8C)	0.9800
C(9)-H(9A)	0.9800
C(9)-H(9B)	0.9800
C(9)-H(9C)	0.9800
C(10)-H(10A)	0.9800
C(10)-H(10B)	0.9800
C(10)-H(10C)	0.9800
C(11)-C(12A)	1.434(18)
C(11)-C(12)	1.503(5)
C(11)-C(13)	1.537(5)
C(11)-C(14)	1.552(5)
C(11)-C(13A)	1.552(18)
C(11)-C(14A)	1.614(19)
C(12)-H(12A)	0.9800
C(12)-H(12B)	0.9800
C(12)-H(12C)	0.9800
C(13)-H(13A)	0.9800
C(13)-H(13B)	0.9800
C(13)-H(13C)	0.9800
C(14)-H(14A)	0.9800
C(14)-H(14B)	0.9800
C(14)-H(14C)	0.9800
C(12A)-H(12D)	0.9800
C(12A)-H(12E)	0.9800
C(12A)-H(12F)	0.9800
C(13A)-H(13D)	0.9800
C(13A)-H(13E)	0.9800
C(13A)-H(13F)	0.9800
C(14A)-H(14D)	0.9800
C(14A)-H(14E)	0.9800
C(14A)-H(14F)	0.9800
C(1)-O(1)-H(1)	106(3)
O(1)-C(1)-C(6)	120.0(2)
O(1)-C(1)-C(2)	118.1(2)
C(6)-C(1)-C(2)	121.9(2)
C(3)-C(2)-C(1)	114.9(2)
C(3)-C(2)-C(7)	122.9(2)
C(1)-C(2)-C(7)	122.3(2)
C(4)-C(3)-C(2)	123.8(3)
C(4)-C(3)-H(3)	118.1
C(2)-C(3)-H(3)	118.1
C(3)-C(4)-C(5)	120.8(3)
C(3)-C(4)-H(4)	119.6
C(5)-C(4)-H(4)	119.6
C(6)-C(5)-C(4)	116.8(3)
C(6)-C(5)-C(11)	122.4(2)
C(4)-C(5)-C(11)	120.8(3)
C(5)-C(6)-C(1)	121.8(3)
C(5)-C(6)-H(6)	119.1
C(1)-C(6)-H(6)	119.1

C(9)-C(7)-C(8)	107.9(3)	C(11)-C(12)-H(12A)	109.5
C(9)-C(7)-C(2)	110.5(2)	C(11)-C(12)-H(12B)	109.5
C(8)-C(7)-C(2)	111.2(2)	H(12A)-C(12)-H(12B)	109.5
C(9)-C(7)-C(10)	110.5(3)	C(11)-C(12)-H(12C)	109.5
C(8)-C(7)-C(10)	107.5(2)	H(12A)-C(12)-H(12C)	109.5
C(2)-C(7)-C(10)	109.1(2)	H(12B)-C(12)-H(12C)	109.5
C(7)-C(8)-H(8A)	109.5	C(11)-C(13)-H(13A)	109.5
C(7)-C(8)-H(8B)	109.5	C(11)-C(13)-H(13B)	109.5
H(8A)-C(8)-H(8B)	109.5	H(13A)-C(13)-H(13B)	109.5
C(7)-C(8)-H(8C)	109.5	C(11)-C(13)-H(13C)	109.5
H(8A)-C(8)-H(8C)	109.5	H(13A)-C(13)-H(13C)	109.5
H(8B)-C(8)-H(8C)	109.5	H(13B)-C(13)-H(13C)	109.5
C(7)-C(9)-H(9A)	109.5	C(11)-C(14)-H(14A)	109.5
C(7)-C(9)-H(9B)	109.5	C(11)-C(14)-H(14B)	109.5
H(9A)-C(9)-H(9B)	109.5	H(14A)-C(14)-H(14B)	109.5
C(7)-C(9)-H(9C)	109.5	C(11)-C(14)-H(14C)	109.5
H(9A)-C(9)-H(9C)	109.5	H(14A)-C(14)-H(14C)	109.5
H(9B)-C(9)-H(9C)	109.5	H(14B)-C(14)-H(14C)	109.5
C(7)-C(10)-H(10A)	109.5	C(11)-C(12A)-H(12D)	109.5
C(7)-C(10)-H(10B)	109.5	C(11)-C(12A)-H(12E)	109.5
H(10A)-C(10)-H(10B)	109.5	H(12D)-C(12A)-H(12E)	109.5
C(7)-C(10)-H(10C)	109.5	C(11)-C(12A)-H(12F)	109.5
H(10A)-C(10)-H(10C)	109.5	H(12D)-C(12A)-H(12F)	109.5
H(10B)-C(10)-H(10C)	109.5	H(12E)-C(12A)-H(12F)	109.5
C(12A)-C(11)-C(5)	110.5(10)	C(11)-C(13A)-H(13D)	109.5
C(12)-C(11)-C(5)	110.3(2)	C(11)-C(13A)-H(13E)	109.5
C(12)-C(11)-C(13)	109.6(3)	H(13D)-C(13A)-H(13E)	109.5
C(5)-C(11)-C(13)	112.4(2)	C(11)-C(13A)-H(13F)	109.5
C(12)-C(11)-C(14)	109.6(3)	H(13D)-C(13A)-H(13F)	109.5
C(5)-C(11)-C(14)	108.7(3)	H(13E)-C(13A)-H(13F)	109.5
C(13)-C(11)-C(14)	106.1(3)	C(11)-C(14A)-H(14D)	109.5
C(12A)-C(11)-C(13A)	114.9(15)	C(11)-C(14A)-H(14E)	109.5
C(5)-C(11)-C(13A)	111.9(8)	H(14D)-C(14A)-H(14E)	109.5
C(12A)-C(11)-C(14A)	111.5(14)	C(11)-C(14A)-H(14F)	109.5
C(5)-C(11)-C(14A)	103.7(9)	H(14D)-C(14A)-H(14F)	109.5
C(13A)-C(11)-C(14A)	103.6(14)	H(14E)-C(14A)-H(14F)	109.5

Table 4. Anisotropic displacement parameters ($Å^2$ x 10^3) for kk3mc. The anisotropic displacement factor exponent takes the form: $-2\pi^2[h^2 a^{*2}U^{11} + ... + 2 h k a^* b^* U^{12}]$

	U^{11}	U^{22}	U^{33}	U^{23}	U^{13}	U^{12}
O(1)	30(1)	24(1)	29(1)	3(1)	13(1)	4(1)
C(1)	20(2)	19(1)	18(1)	-2(1)	2(1)	-3(1)
C(2)	18(1)	17(1)	16(1)	-4(1)	-4(1)	-2(1)
C(3)	14(1)	18(1)	22(1)	-4(1)	1(1)	2(1)
C(4)	19(1)	23(2)	19(1)	-1(1)	1(1)	-2(1)
C(5)	20(2)	17(1)	19(1)	-1(1)	-4(1)	-2(1)
C(6)	20(2)	19(2)	22(2)	-4(1)	0(1)	2(1)
C(7)	22(2)	17(2)	26(2)	2(1)	-3(1)	-1(1)
C(8)	33(2)	21(2)	40(2)	5(1)	-1(2)	5(1)
C(9)	38(2)	26(2)	29(2)	6(1)	-6(2)	-1(1)
C(10)	30(2)	23(2)	36(2)	3(1)	-4(1)	-4(1)
C(11)	27(2)	22(2)	26(2)	4(1)	4(1)	2(1)
C(12)	67(3)	28(2)	30(2)	4(2)	-6(2)	15(2)
C(13)	42(2)	31(2)	33(2)	12(2)	5(2)	13(2)
C(14)	45(2)	20(2)	58(3)	11(2)	-4(2)	-7(2)
C(12A)	67(3)	28(2)	30(2)	4(2)	-6(2)	15(2)
C(13A)	42(2)	31(2)	33(2)	12(2)	5(2)	13(2)
C(14A)	45(2)	20(2)	58(3)	11(2)	-4(2)	-7(2)

Table 5. Hydrogen coordinates (x 10^4) and isotropic displacement parameters (Å^2 x 10^3) for kk3mc.

	x	y	z	U(eq)
H(1)	5380(50)	5440(50)	6670(30)	76(15)
H(3)	9435	4267	4585	22
H(4)	9106	6251	3713	24
H(6)	5794	6900	5436	24
H(8A)	9234	2119	4967	47
H(8B)	9448	1450	6095	47
H(8C)	10106	2870	5839	47
H(9A)	8969	3848	7428	47
H(9B)	8435	2366	7646	47
H(9C)	7405	3587	7594	47
H(10A)	6059	2458	6203	44
H(10B)	7036	1193	6229	44
H(10C)	6762	1956	5147	44
H(12A)	7747	7271	2434	62
H(12B)	6847	8580	2292	62
H(12C)	6164	7188	2598	62
H(13A)	5805	9670	3831	53
H(13B)	6027	9057	4978	53
H(13C)	5105	8290	4147	53
H(14A)	9159	8573	3734	61
H(14B)	8437	9255	4718	61
H(14C)	8202	9835	3563	61
H(12D)	6799	9997	4113	62
H(12E)	6200	9023	4991	62
H(12F)	7773	9306	4948	62
H(13D)	8238	7469	2521	53
H(13E)	7927	9037	2517	53
H(13F)	9062	8450	3272	53
H(14D)	5926	6982	2731	61
H(14E)	5060	7537	3693	61
H(14F)	5522	8528	2777	61

8. References

1 a) H. E. Gottlieb, V. Kotlyar, A. Nudelman, *J. Org. Chem.* **1997**, *62*, 7512–7515; b) G. R. Fulmer, A. J. M. Miller, N. H. Sherden, H. E. Gottlieb, A. Nudelman, B. M. Stoltz, J. E. Bercaw, K. I. Goldberg, *Organometallics* **2010**, *29*, 2176–2179.

2 CYLview, 1.0b, C. Y. Legault, Université de Sherbrooke, 2009 (http://www.cylview.org)

3 L. J. Farrugia, *J. Appl. Cryst.* **2012**, *45*, 849–854.

4 a) M. Rolff, J. Schottenheim, G. Peters, F. Tuczek, *Angew. Chem. Int. Ed.* **2010**, *49*, 6438–6442; *Angew. Chem.* **2010**, *122*, 6583–6587. b) Kalaichelvan, S.; N. Sundaraganesan, O. Dereli, U. Sayin, *Spectrochim. Acta A* **2012**, *85*, 198–209.

6 H. Nemoto, T. Nishiyama, S. Akai, *Org. Lett.* **2011**, *13*, 2714–2717.

5 A.V. Nizovtsev, A. Scheurer, B. Kosog, F. W. Heinemann, K. Meyer, *Eur. J. Inorg. Chem.* **2013**, *14*, 2538–2548.

7 C. L. Lohre, T. Dröge, C. Wang, F. Glorius, *Chem. Eur. J.* **2011**, *17*, 6052–6055.

8 M. Tashiro, T. Yamato, *Org. Prep. Proced. Int.* **1978**, *10*, 143–148.

9 Ö. Dilek, M. A. Tezeren, T. Tilki, E. Ertürk, *Tetrahedron*. **2018**, *74*, 268–286.

10 H.-L. Qi, D.-S. Chen, J.-S. Ye, J.-M. Huang, *J. Org. Chem.* **2013**, *78*, 7482–7487.

11 a) Y. Grell, N. Demirel, K. Harms, E. Meggers, *Organometallics* **2019**, *38*, 3852–3859. b) R. I. Khusnutdinov, N. A. Shchadneva, L. F. Khisamova, *Russ. J. Org. Chem.* **2015**, *51*, 1545–1550.

12 R. Naik, M. Won, B. Kim, Y. Xia, H. K. Choi, G. Jin, Y. Jung, H. M. Kim, K. Lee, *J. Med. Chem.* **2012**, *55*, 23, 10564–10571.

13 A. Fischer, K. C. Teo, *Can, J. Chem.* **1978**, *56*, 258–266.

14 V. Arredondo, S. C. Hiew, E. S. Gutman, I. D. U. A. Premachandra, D. L. Van Vranken, *Angew. Chem. Int. Ed.* **2017**, *56*, 4156–4159.

15 Y. Mizukami, Z. Song, T. Takahashi, *Org. Lett.* **2015**, *17*, 5942–5945.

16 Gaussian 16, Revision A.01, Frisch, M. J.; Trucks, G. W.; Schlegel, H. B.; Scuseria, G. E.; Robb, M. A.; Cheeseman, J. R.; Scalmani, G.; Barone, V.; Petersson, G. A.; Nakatsuji, H.; Li, X.; Caricato, M.; Marenich, A. V.; Bloino, J.; Janesko, B. G.; Gomperts, R.; Mennucci, B.; Hratchian, H. P.; Ortiz, J. V.; Izmaylov, A. F.; Sonnenberg, J. L.; Williams-Young, D.; Ding, F.; Lipparini, F.; Egidi, F.; Goings, J.; Peng, B.; Petrone, A.; Henderson, T.; Ranasinghe, D.; Zakrzewski, V. G.; Gao, J.; Rega, N.; Zheng, G.; Liang, W.; Hada, M.; Ehara, M.; Toyota, K.; Fukuda, R.; Hasegawa, J.; Ishida, M.; Nakajima, T.; Honda, Y.; Kitao, O.; Nakai, H.; Vreven, T.; Throssell, K.; Montgomery, J. A., Jr.; Peralta, J. E.; Ogliaro, F.; Bearpark, M. J.; Heyd, J. J.; Brothers, E. N.; Kudin, K. N.; Staroverov, V. N.; Keith, T. A.; Kobayashi, R.; Normand, J.; Raghavachari, K.; Rendell, A. P.; Burant, J. C.; Iyengar, S. S.; Tomasi, J.; Cossi, M.; Millam, J. M.; Klene, M.; Adamo, C.; Cammi, R.; Ochterski, J. W.; Martin, R. L.; Morokuma, K.; Farkas, O.; Foresman, J. B.; Fox, D. J. Gaussian, Inc., Wallingford CT, 2016.

17 S. Grimme, *J. Chem. Phys*. **2006**, *124*, 034108.

18 S. Grimme, S. Ehrlich, L. Goerigk, *J. Comp. Chem*. **2011**, *32*, 1456–1465.

19 L. Goerigk, S. Grimme, *J. Chem. Theory Comput*. **2011**, *7*, 291–309.

20 F. Weigend, R. Ahlrichs, *Phys. Chem. Chem. Phys*. **2005**, *7*, 3297–3305.

21 A. Schaefer, C. Huber, R. Ahlrichs, *J. Chem. Phys*. **1994**, *100*, 5829–5835.

22 B. P. Pritchard, D. Altarawy, B. Didier, T. D. Gibson, T. L. Windus. *J. Chem. Inf. Model.* **2019**, *59*, 4814-4820.

23 J. Tomasi, B. Mennucci, R. Cammi, *Chem. Rev*. **2005**, *105*, 2999-3093.

24 G. Luchini, J. V. Alegre-Requena, I. Funes-Ardoiz, R. S. Paton, *F1000Research*, **2020**, *9*, 291.

25 S. Grimme, *Chem. Eur. J*. **2012**, *18*, 9955–9964.

26 K. A. Moltved, K. P. Kepp, *J. Chem. Theory Comput*. **2018**, *14*, 3479−3492.

27 K. M. Ervin, V. F. DeTuri, *J. Phys. Chem. A*, **2002**, *106*, 9947–9956.

28 S. J. Blanksby, G. B. Ellison, *Acc. Chem. Res.* **2003**, *36*, 255–263.

29 B. deB. Darwent, Nat. Bur. Stand. (U.S.), *Nat. Stand. Ref. Data Ser*. **1970**, *31*, 1–52.

30 SAINT, version 8.34A, Bruker (2012), Bruker AXS Inc., Madison, Wisconsin, USA.

31 SADABS, version 2012/1, Bruker (2012), Bruker AXS Inc., Madison, Wisconsin, USA.

32 G. M. Sheldrick, *Acta Cryst.* **2015**, *A71*, 3–8.

33 G. M. Sheldrick, *Acta Cryst.* **2015**, *C71*, 3–8.

34 P. Müller, *Crystallography Reviews* **2009**, *15*, 57–83.

9. NMR Spectra

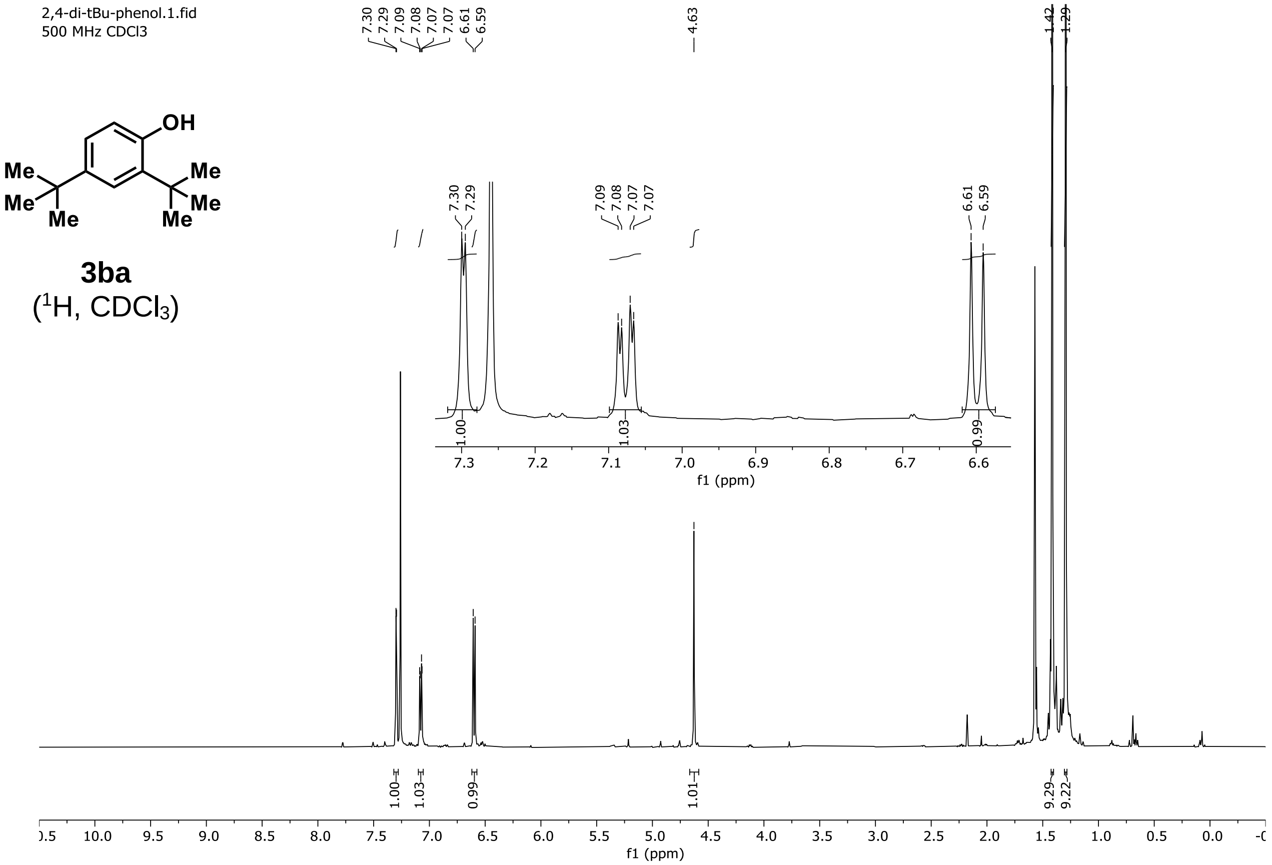

3ba
(^{1}H, $CDCl_3$)

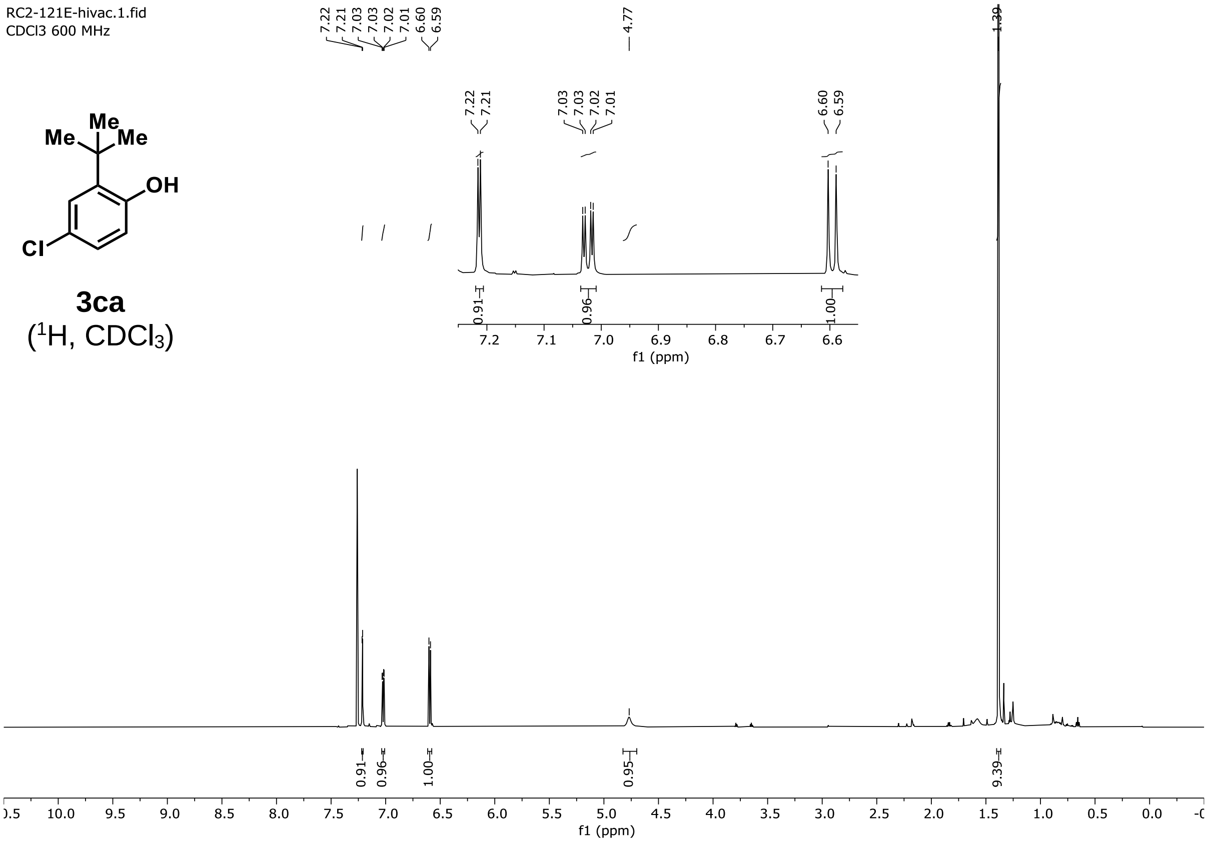

3ca
(^{1}H, $CDCl_3$)

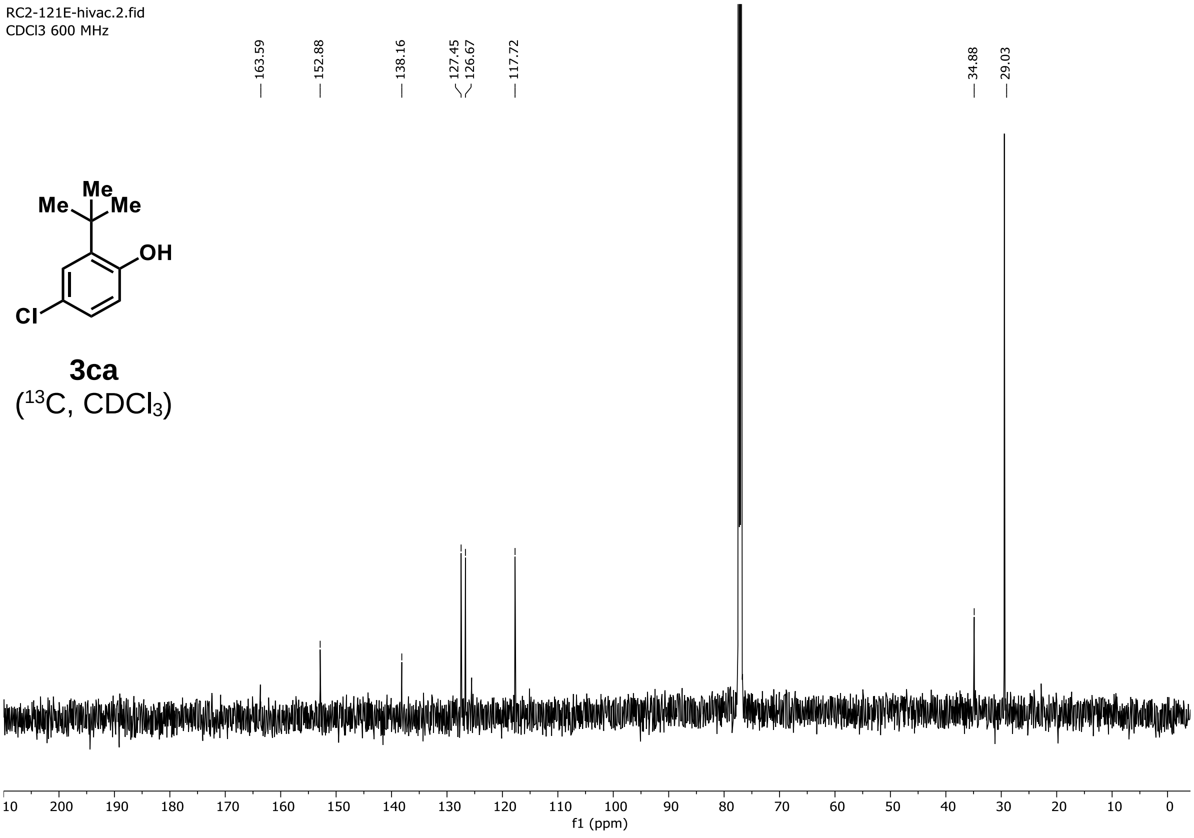

3ca
(^{13}C, $CDCl_3$)

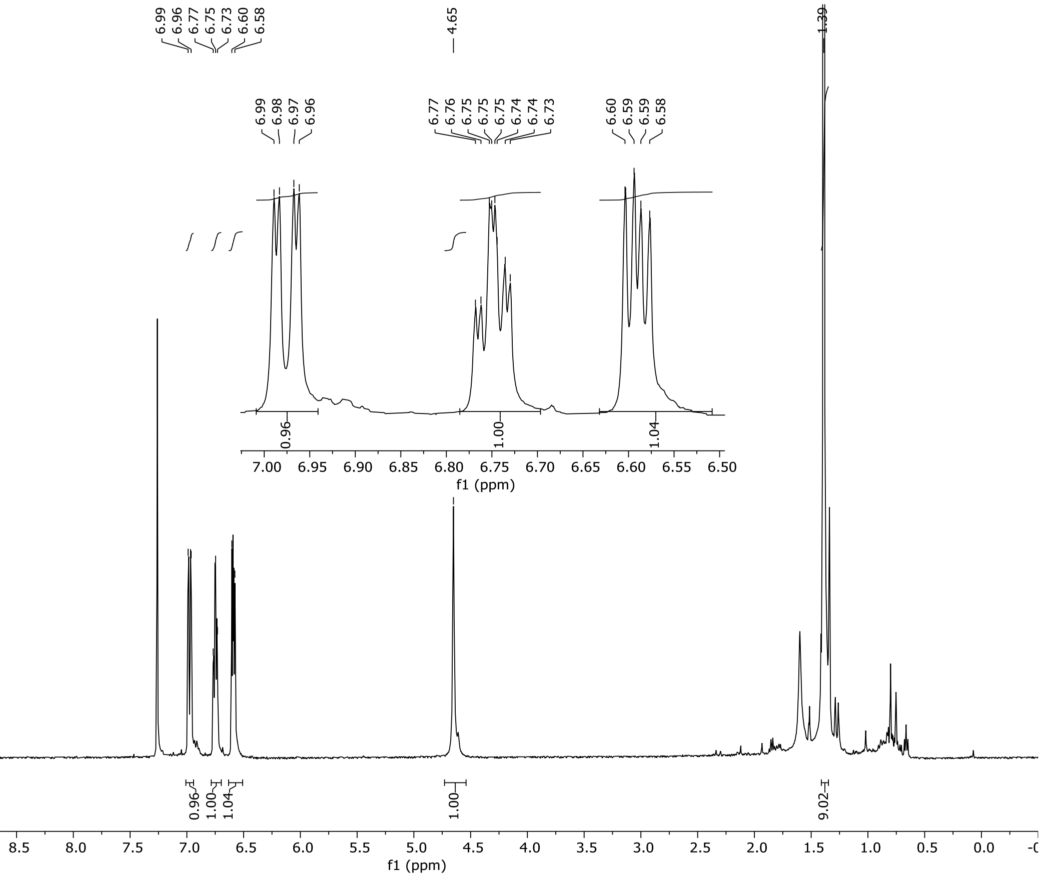

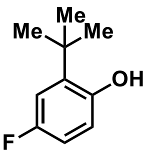

3da
(^{1}H, $CDCl_3$)

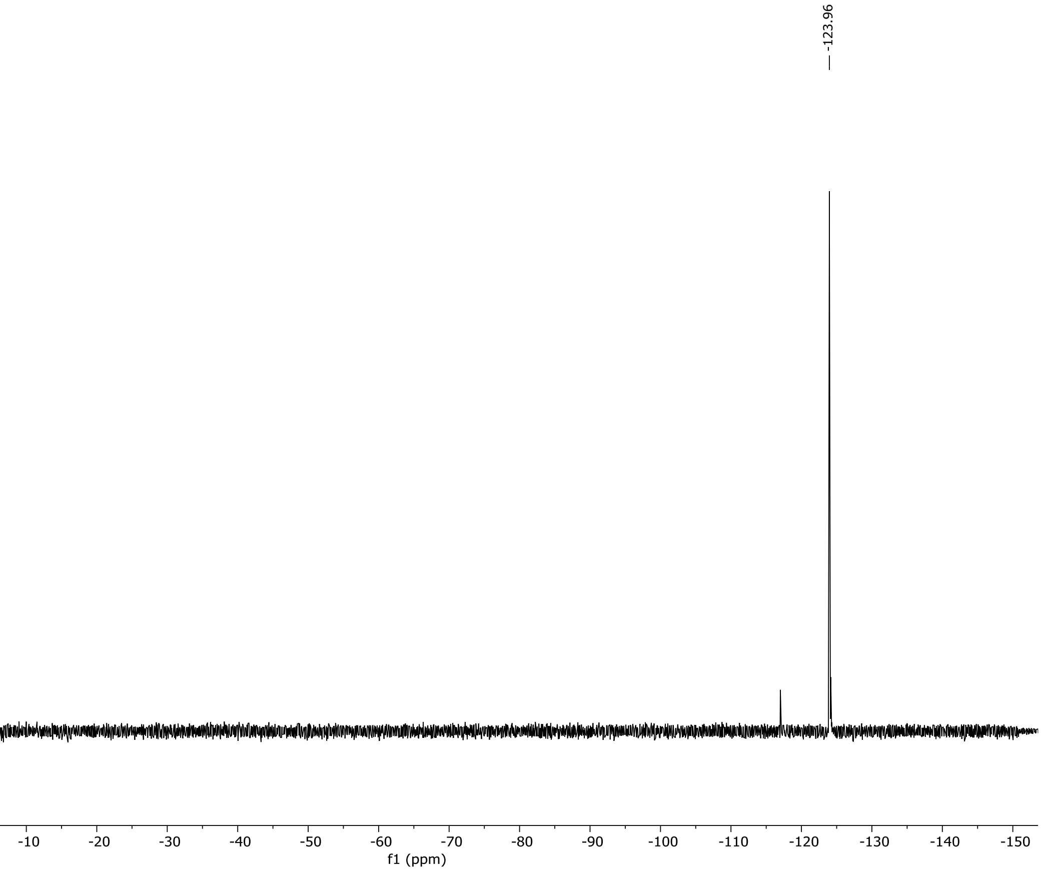

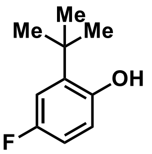

3da
(^{19}F, $CDCl_3$)

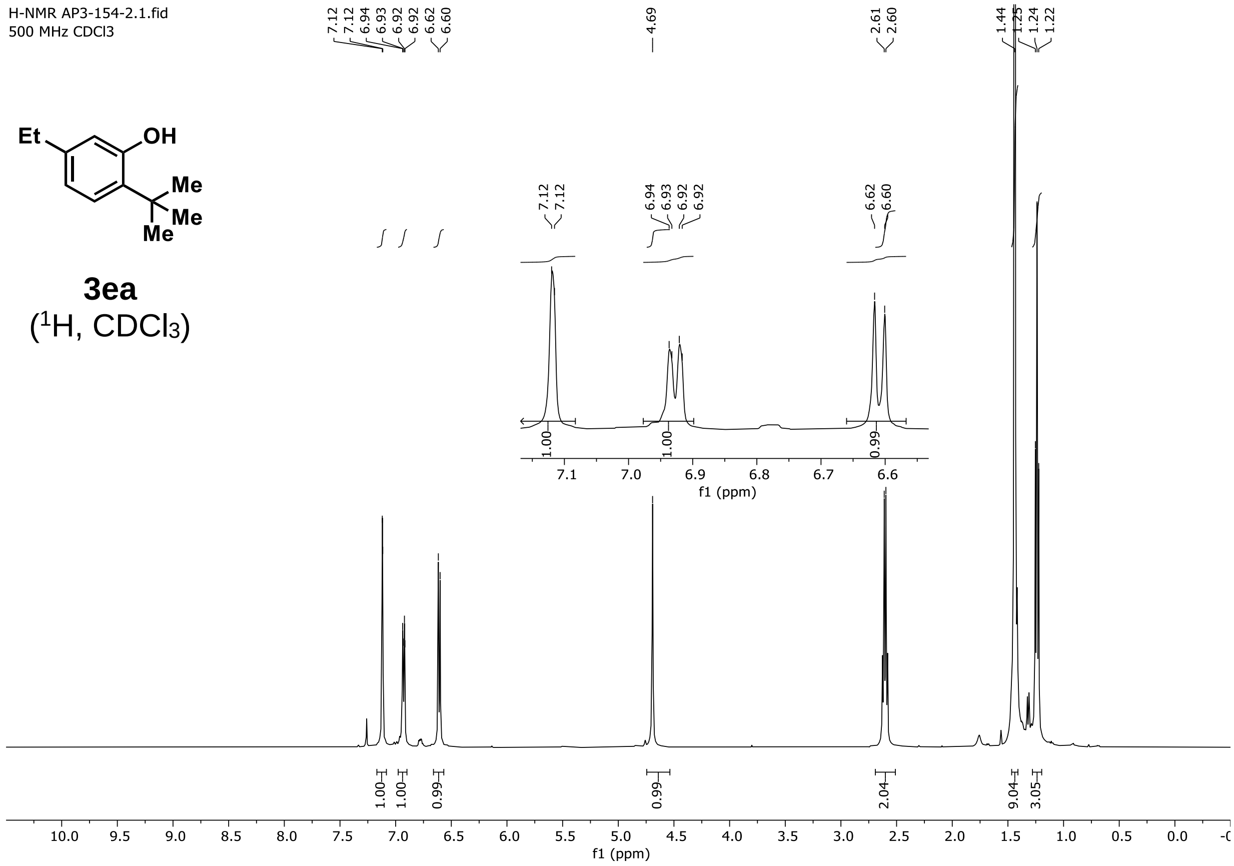
H-NMR AP3-154-2.1.fid
500 MHz CDCl3
Et
OH
Me
Me
Me
3ea
(^{1}H, CDCl3)
7.12
7.12
6.94
6.93
6.92
6.92
6.62
6.60
4.69
2.61
2.60
1.44
1.25
1.24
1.22
1.00
1.00
0.99
0.99
2.04
9.04
3.05
f1 (ppm)

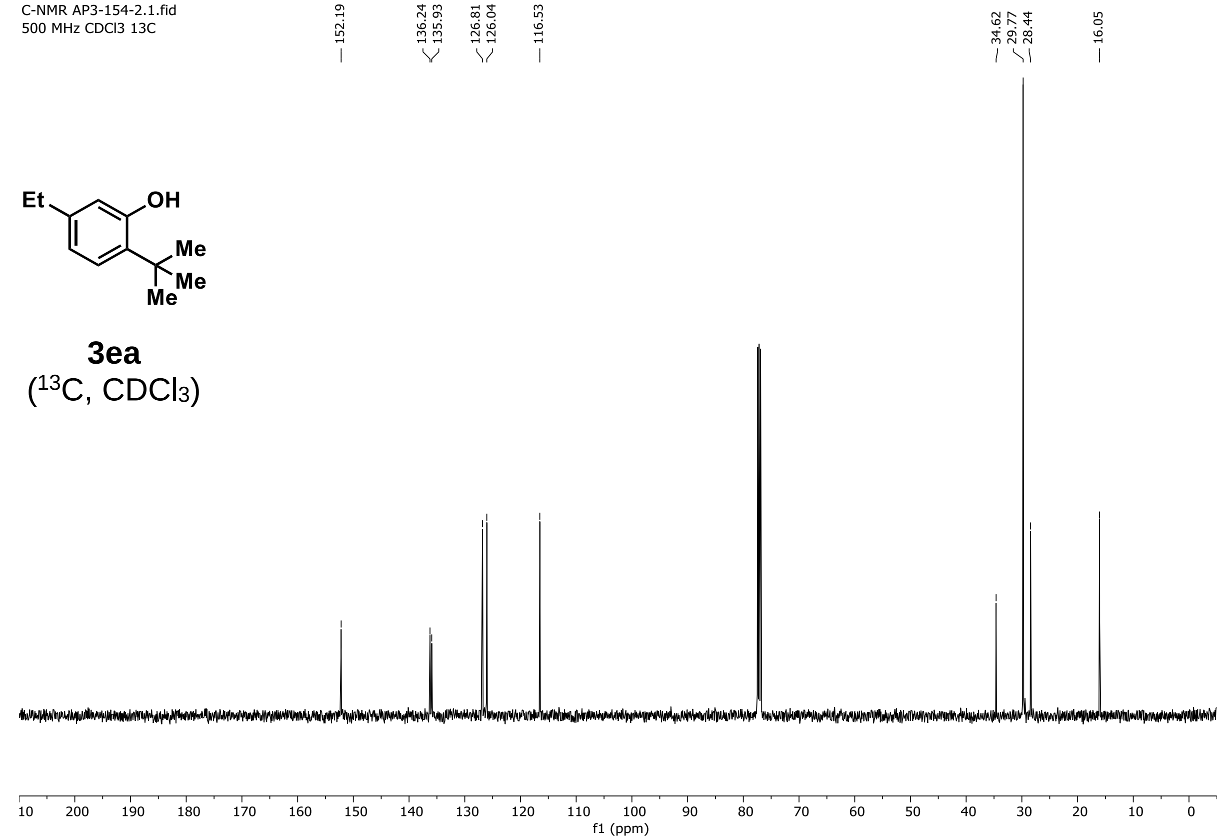
C-NMR AP3-154-2.1.fid
500 MHz CDCl3 13C
Et
OH
Me
Me
Me
3ea
(^{13}C, CDCl3)
152.19
136.24
135.93
126.81
126.04
116.53
34.62
29.77
28.44
16.05
f1 (ppm)

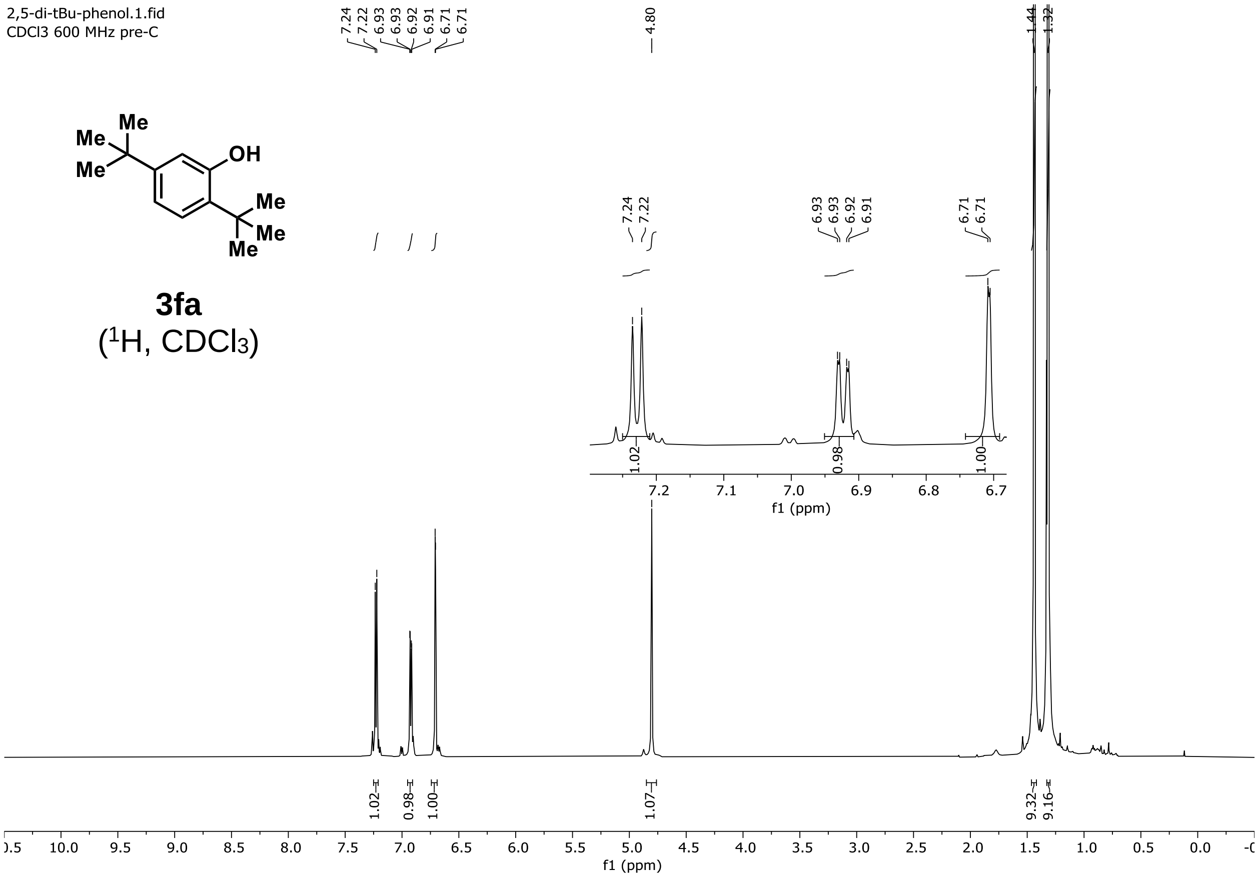
2,5-di-tBu-phenol.1.fid
CDCl3 600 MHz pre-C
3fa
(1H, CDCl3)
f1 (ppm)

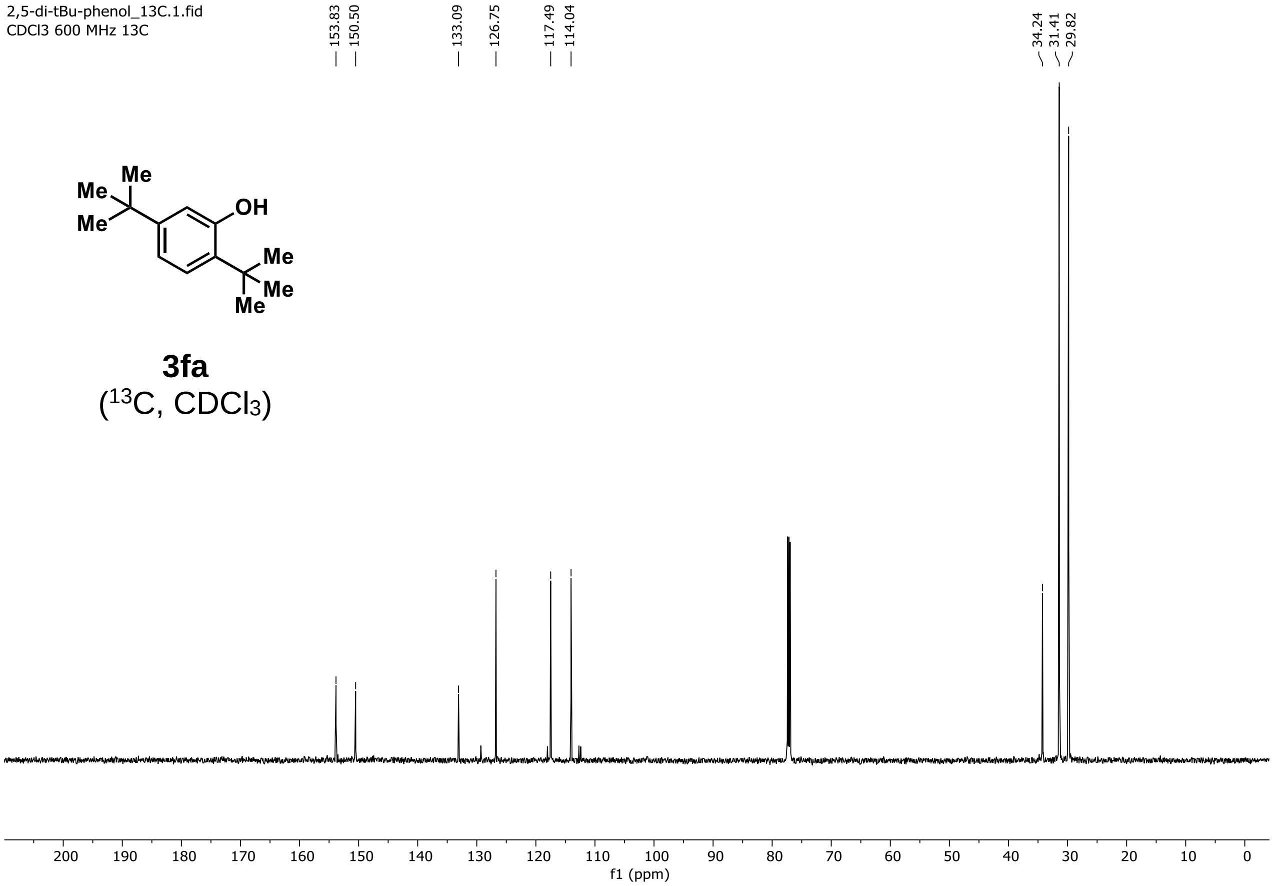
2,5-di-tBu-phenol_13C.1.fid
CDCl3 600 MHz 13C
3fa
(13C, CDCl3)
f1 (ppm)

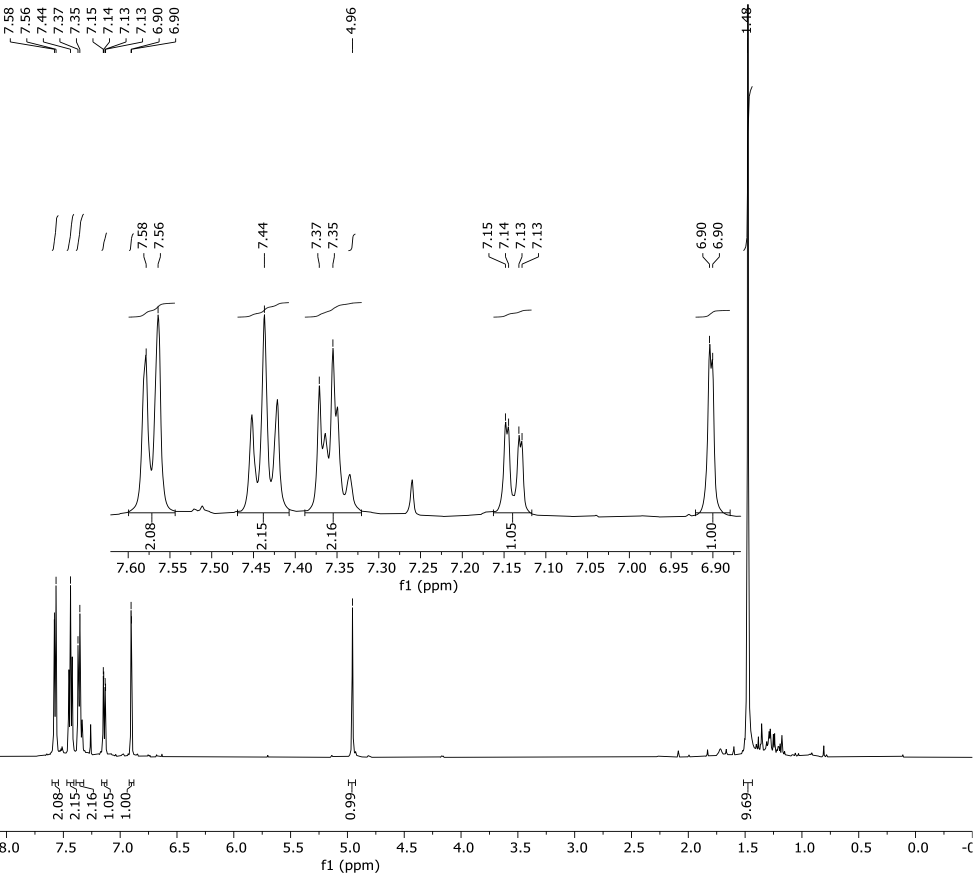

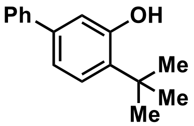

3ga
(^{1}H, $CDCl_3$)

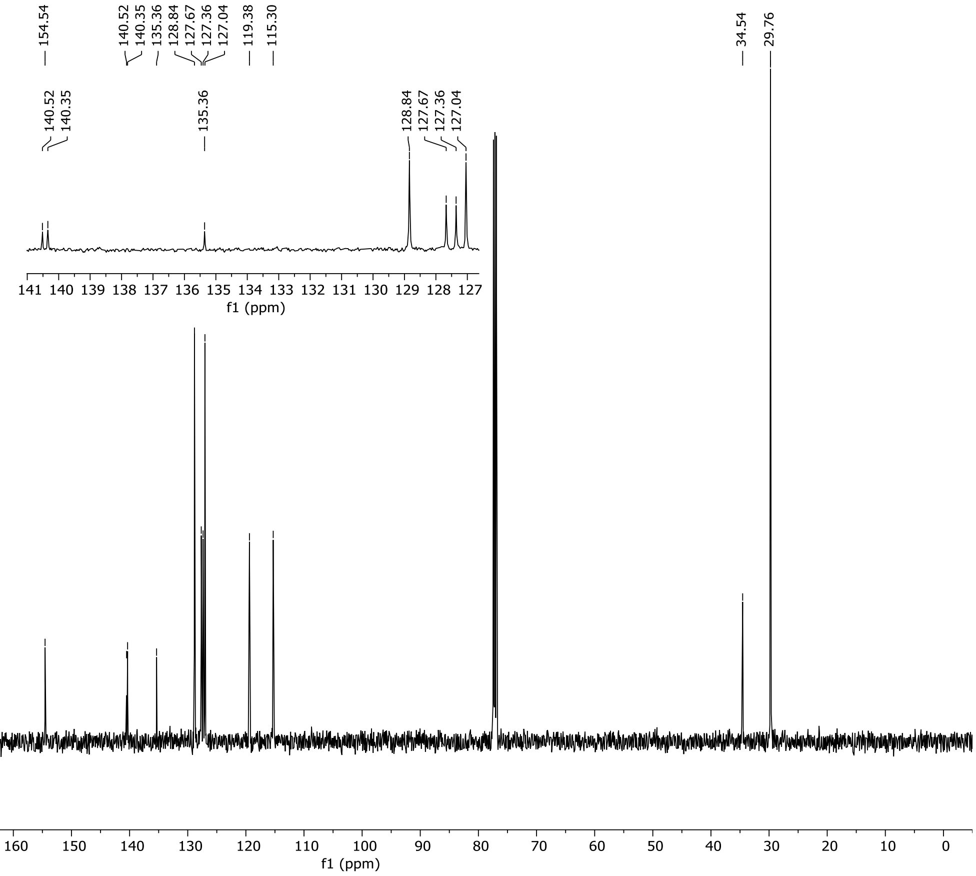

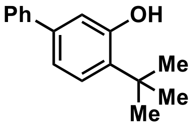

3ga
(^{13}C, $CDCl_3$)

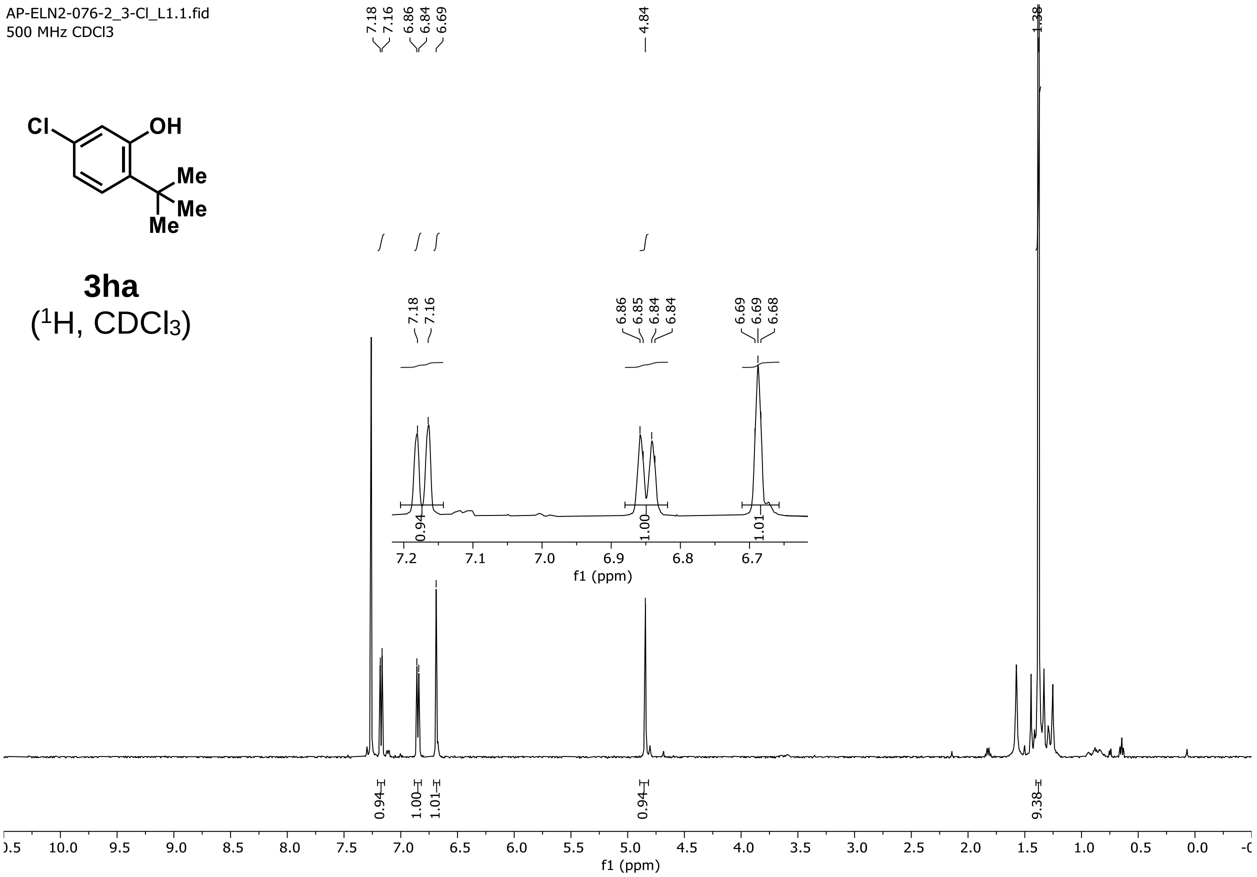
AP-ELN2-076-2_3-Cl_L1.1.fid
500 MHz CDCl3
7.18
7.16
6.86
6.84
6.69
4.84
1.38
Cl
OH
Me
Me
Me
3ha
(1H, CDCl3)
6.85
6.84
6.69
6.68
0.94
1.00
1.01
0.94
9.38
f1 (ppm)

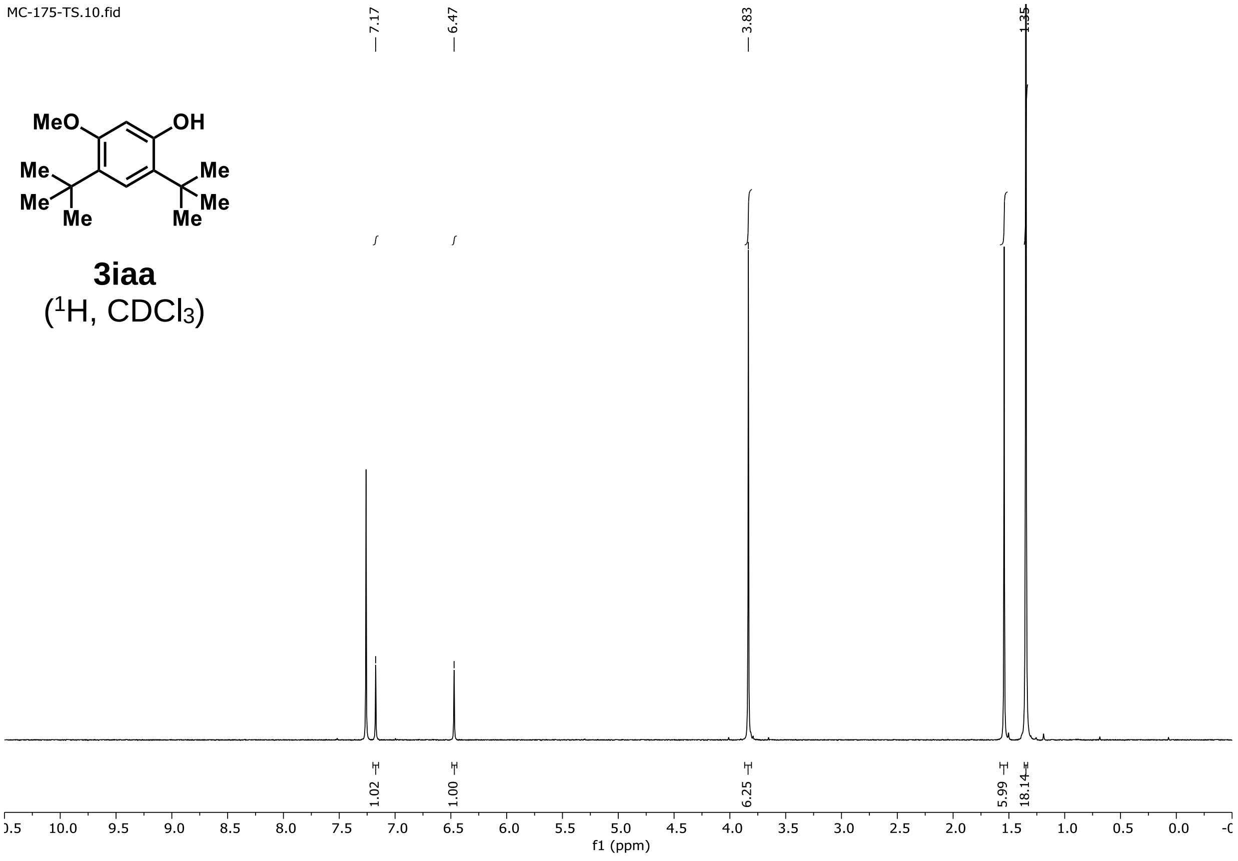
MC-175-TS.10.fid
7.17
6.47
3.83
1.35
MeO
OH
Me
Me
Me
Me
Me
Me
3iaa
(1H, CDCl3)
1.02
1.00
6.25
5.99
18.14
f1 (ppm)

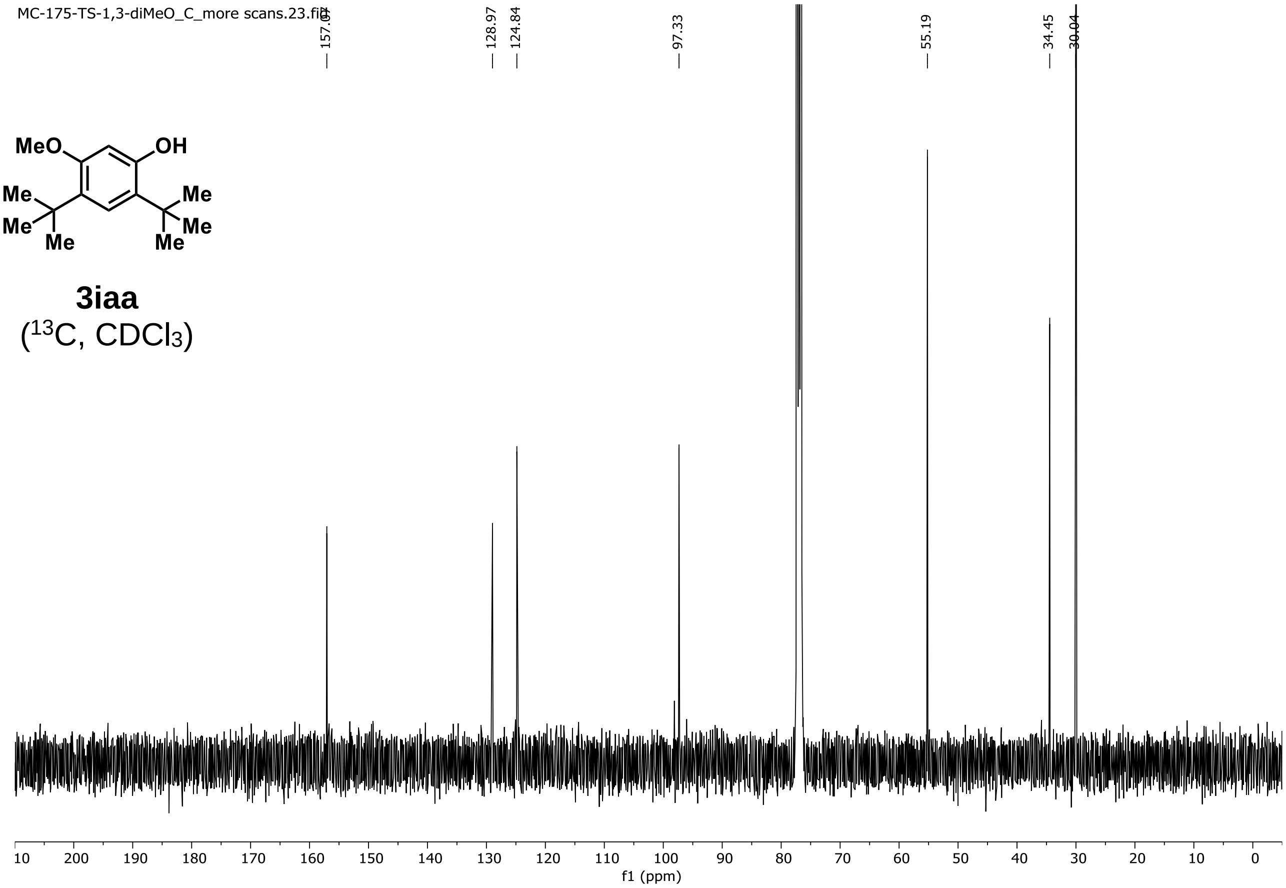
MC-175-TS-1,3-diMeO_C_more scans.23.fid
128.97
124.84
97.33
55.19
34.45
30.04
MeO
OH
Me
Me
Me
Me
Me
Me
3iaa
(13C, CDCl3)
10 200 190 180 170 160 150 140 130 120 110 100 90 80 70 60 50 40 30 20 10 0
f1 (ppm)

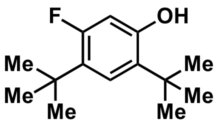

3jaa
(^{1}H, $CDCl_3$)

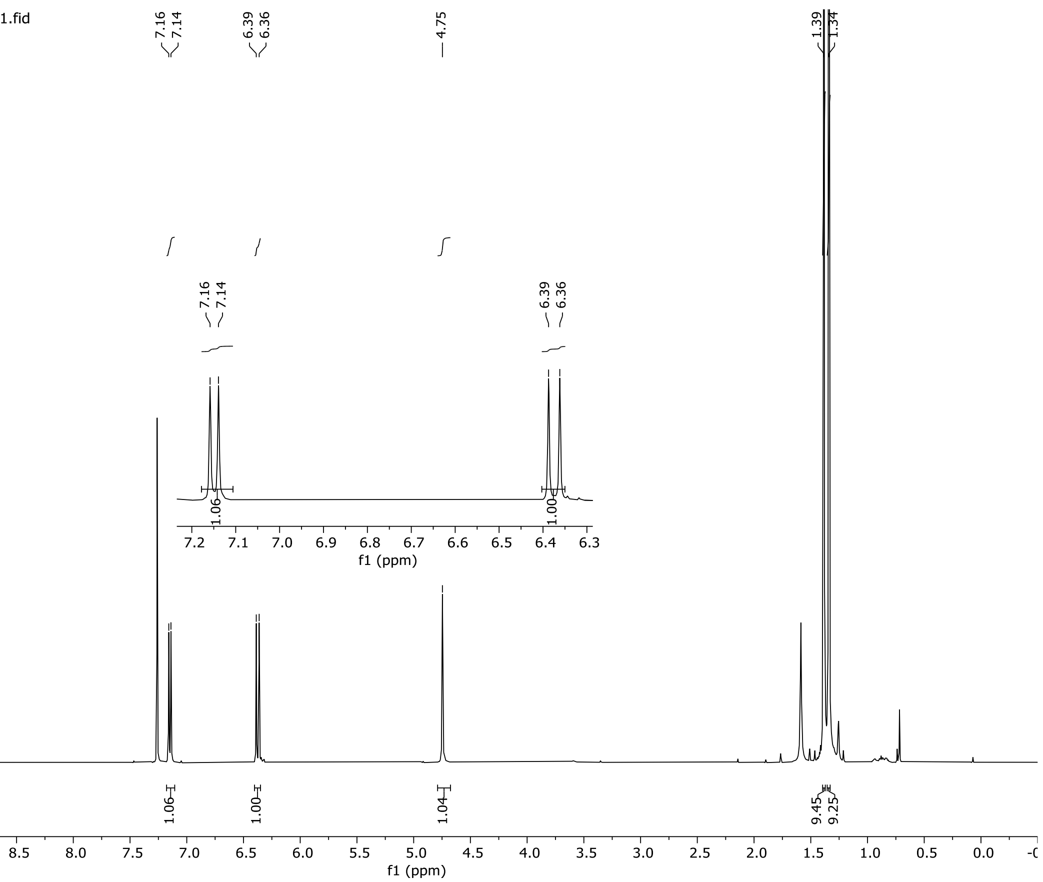

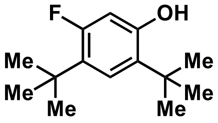

3jaa
(^{13}C, $CDCl_3$)

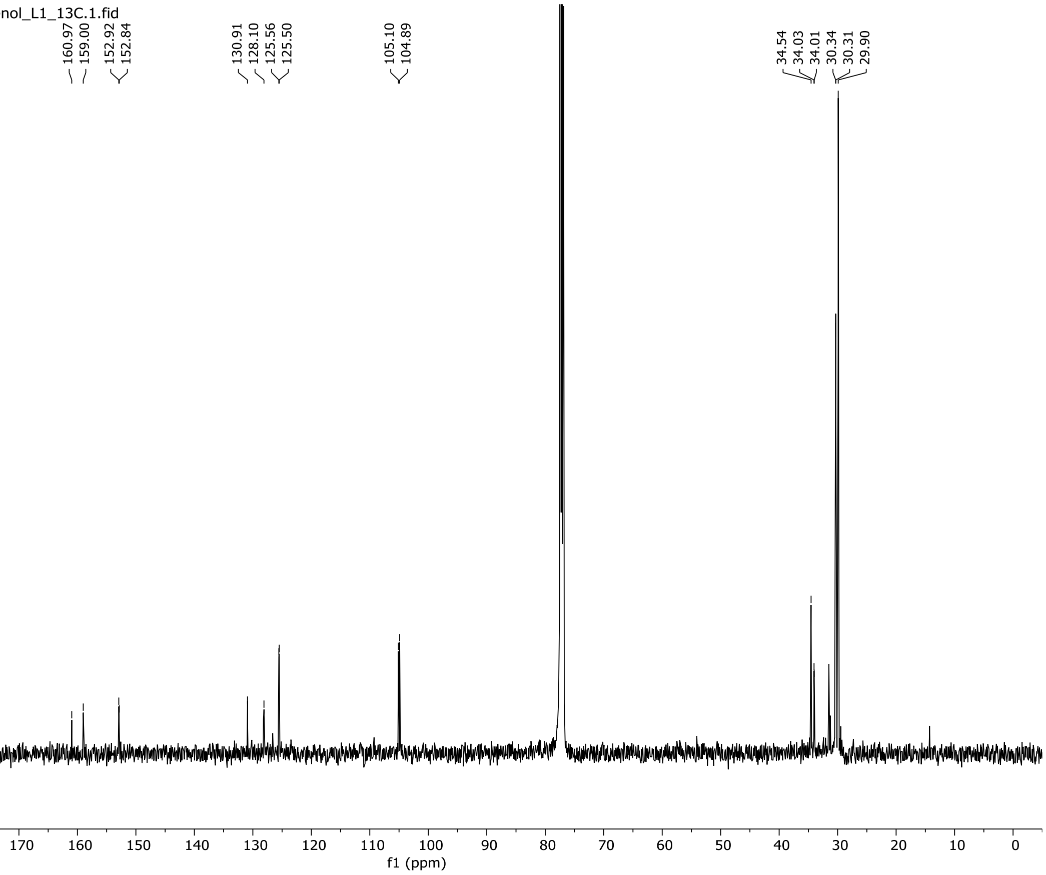

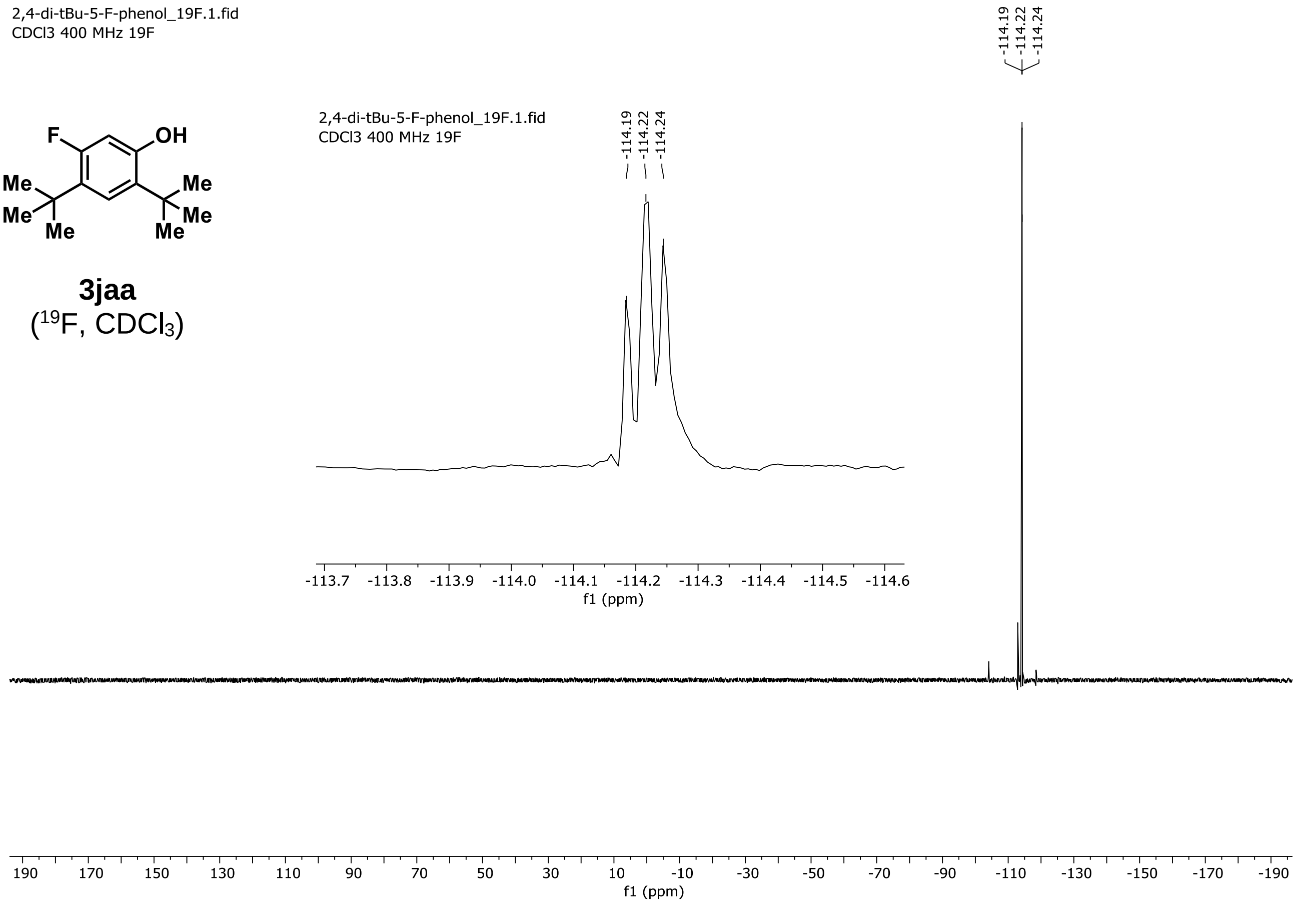

3jaa
(^{19}F, $CDCl_3$)

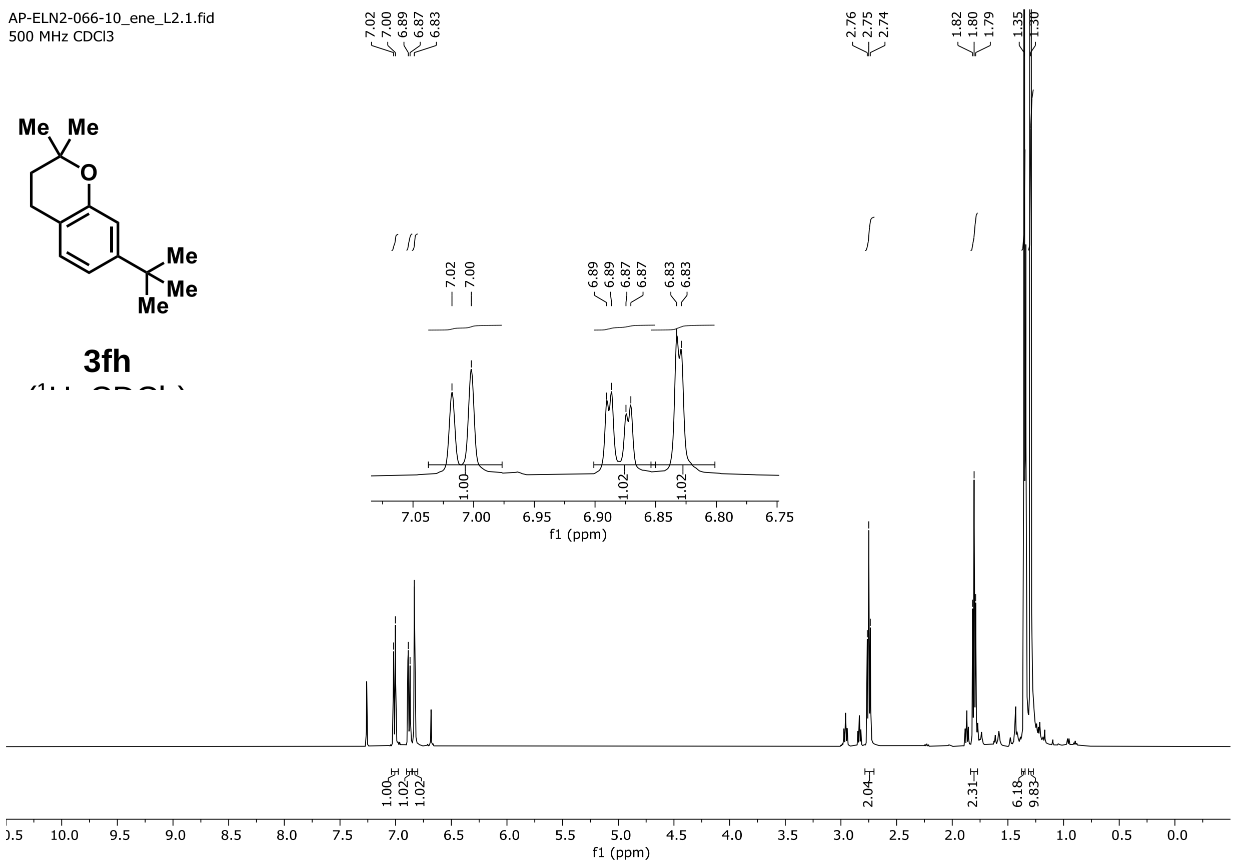

AP-ELN2-066-10_ene_L2.1.fid
500 MHz CDCl3
7.02
7.00
6.89
6.87
6.83
2.76
2.75
2.74
1.82
1.80
1.79
1.35
1.30
Me
Me
O
Me
Me
Me
3fh
1.00
1.02
1.02
2.04
2.31
6.18
9.83
f1 (ppm)

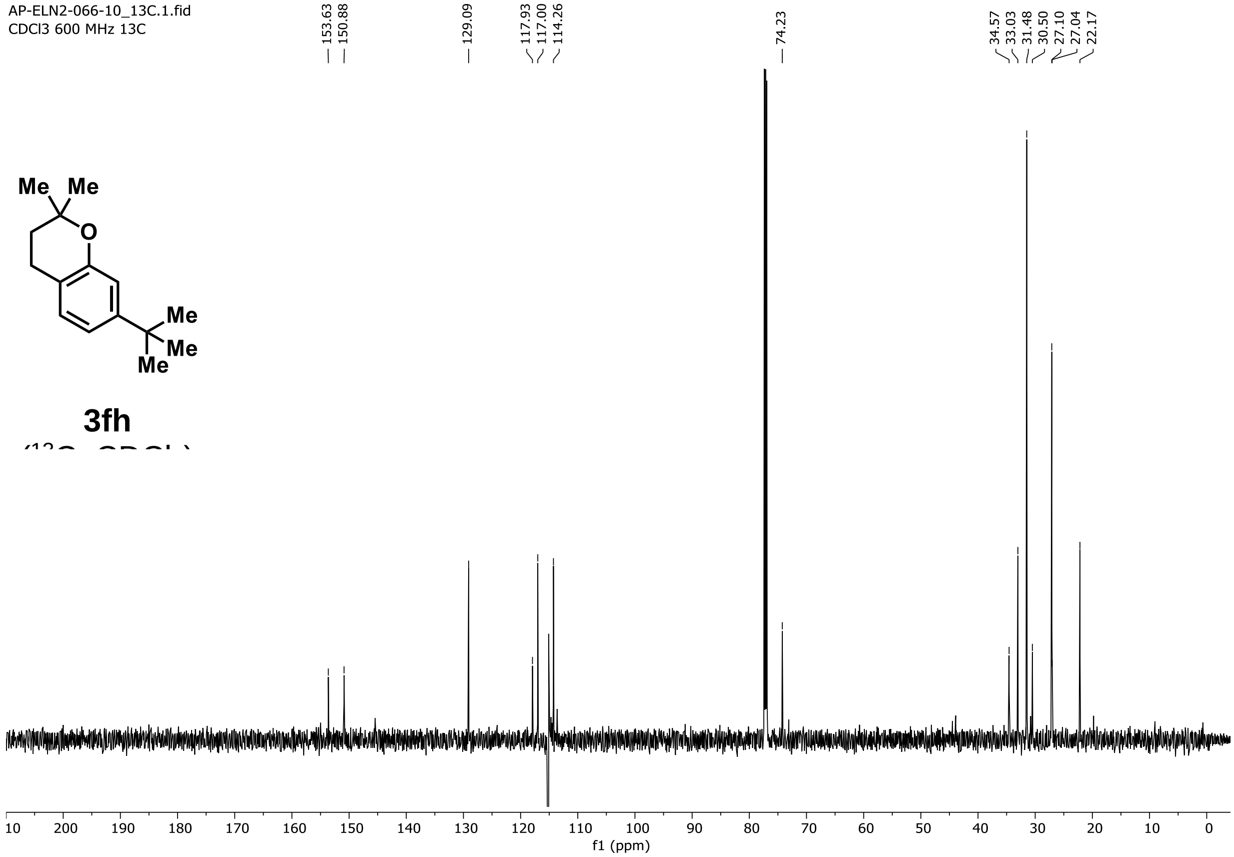

AP-ELN2-066-10_13C.1.fid
CDCl3 600 MHz 13C
153.63
150.88
129.09
117.93
117.00
114.26
74.23
34.57
33.03
31.48
30.50
27.10
27.04
22.17
Me
Me
O
Me
Me
Me
3fh
f1 (ppm)

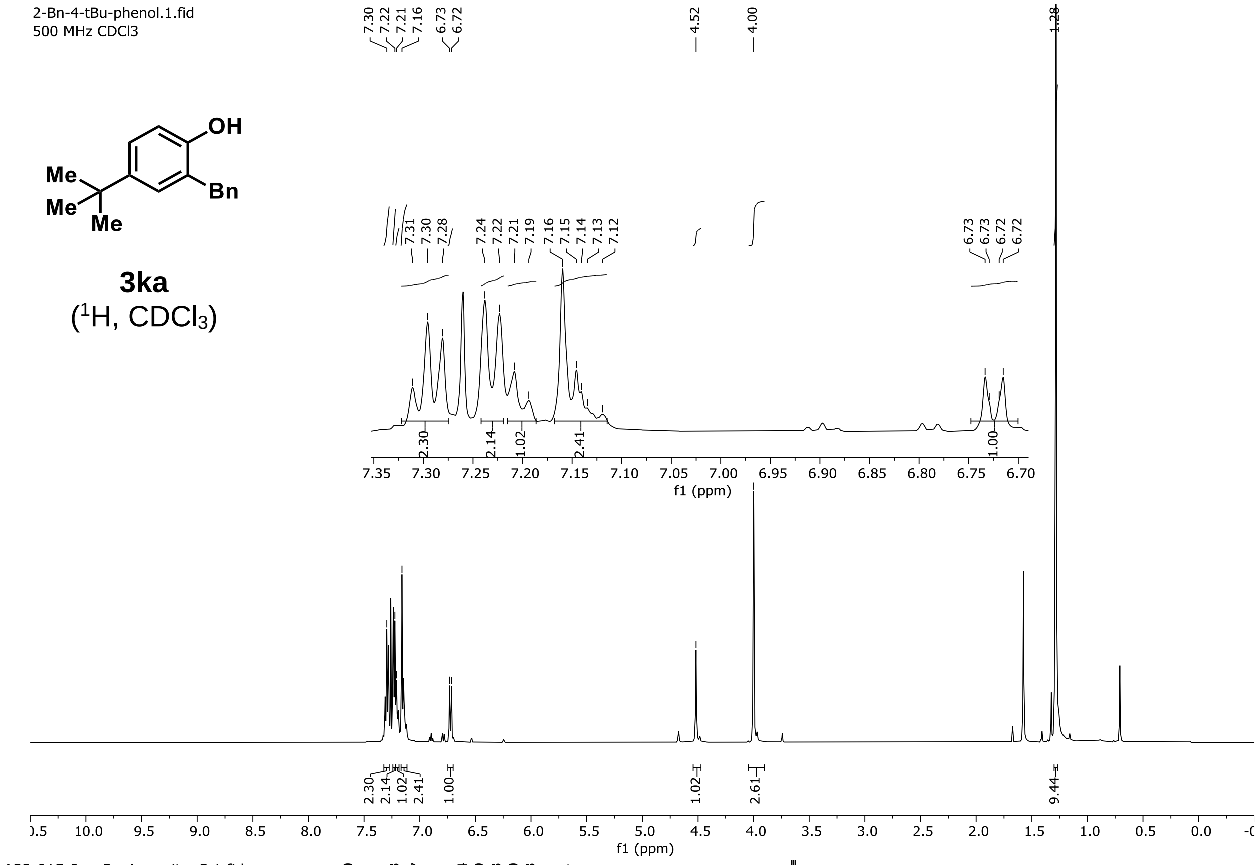

3ka
(^{1}H, $CDCl_3$)

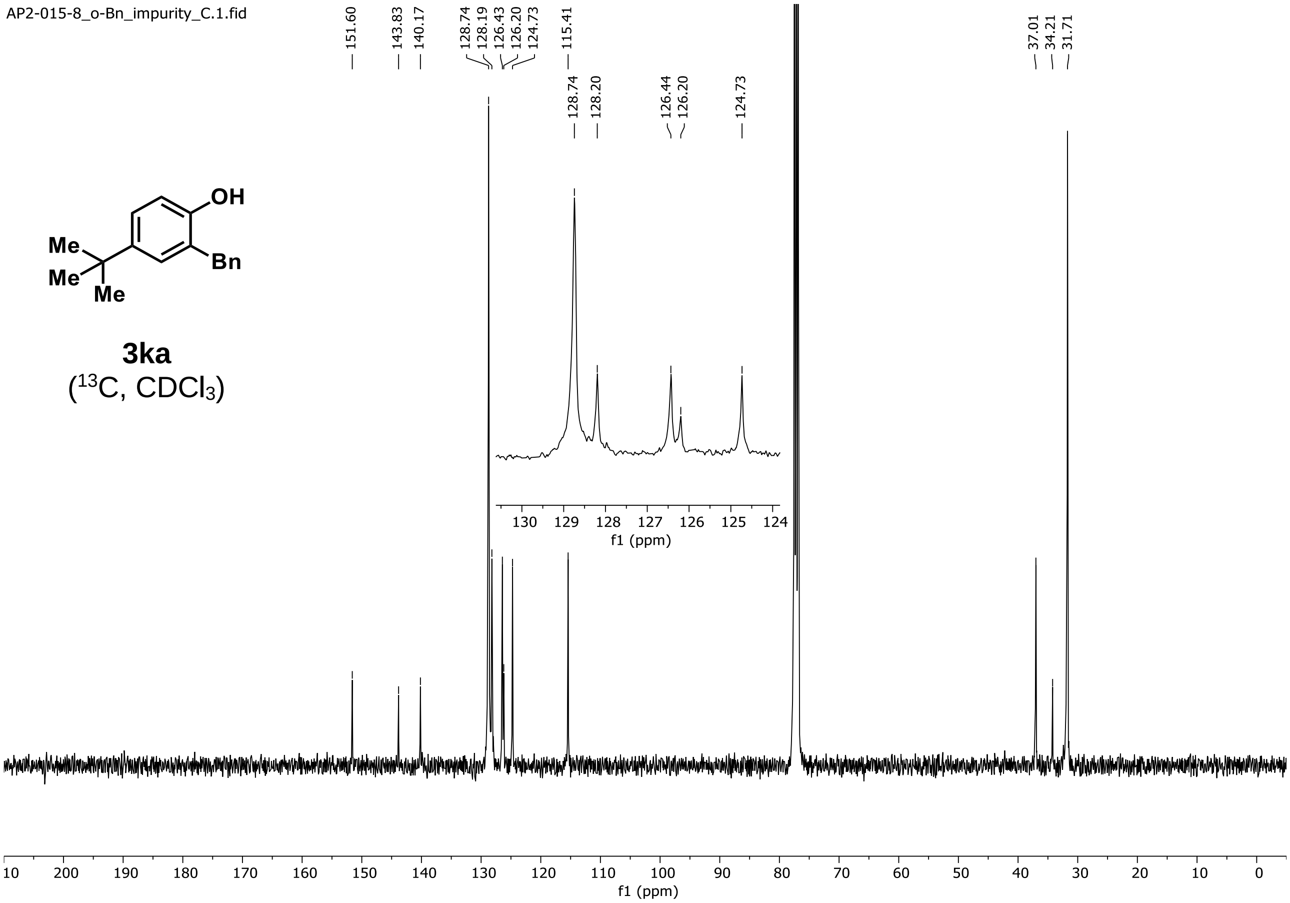

3ka
(^{13}C, $CDCl_3$)

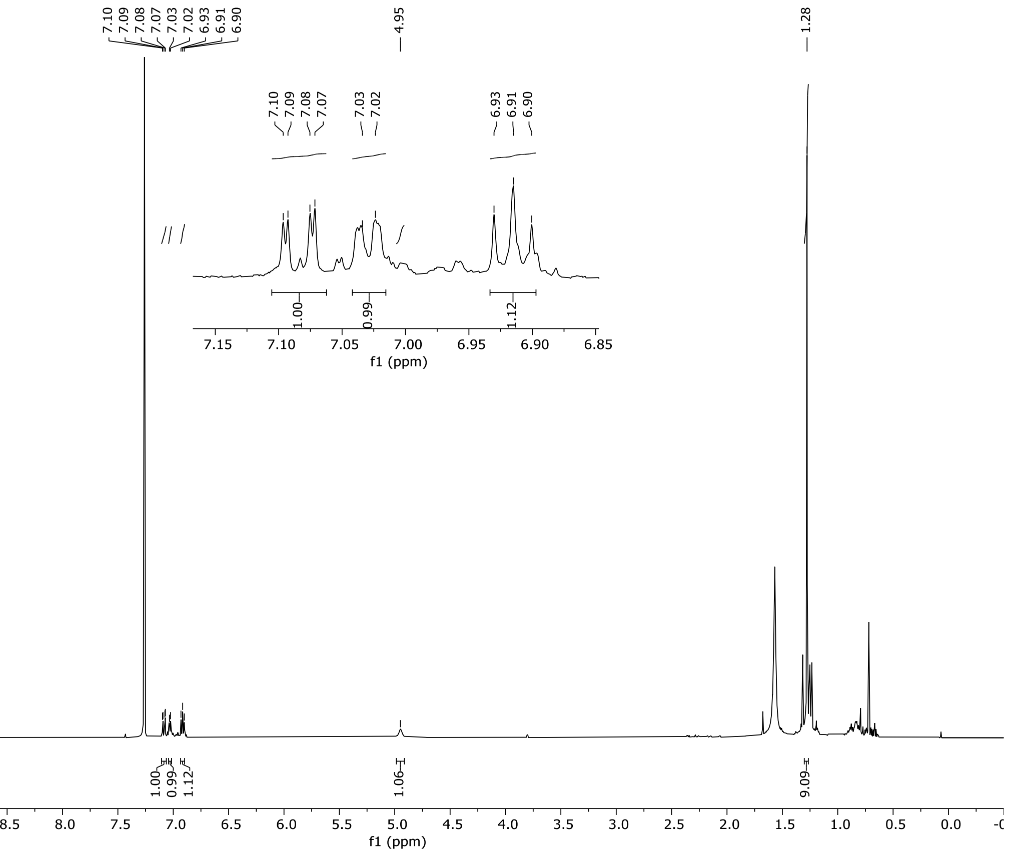

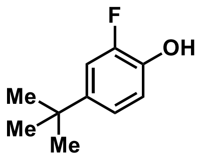

3la
(^{1}H, $CDCl_3$)

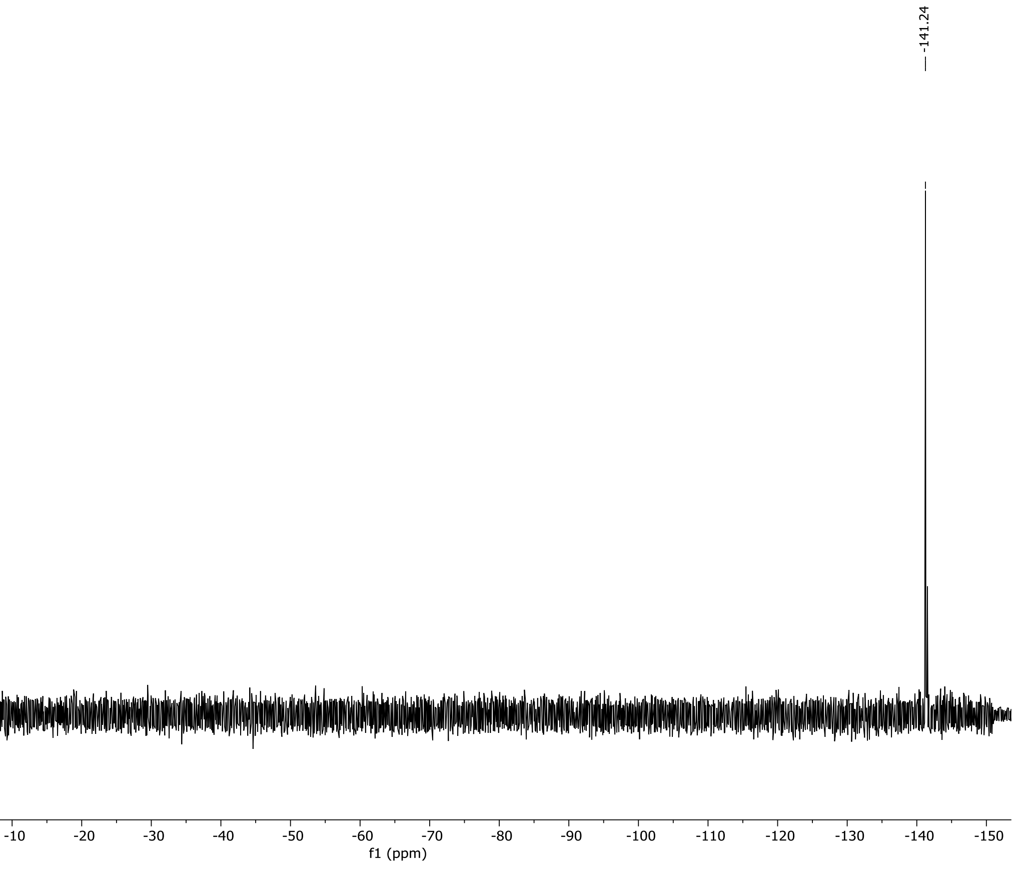

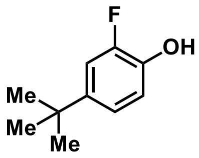

3la
(^{19}F, $CDCl_3$)

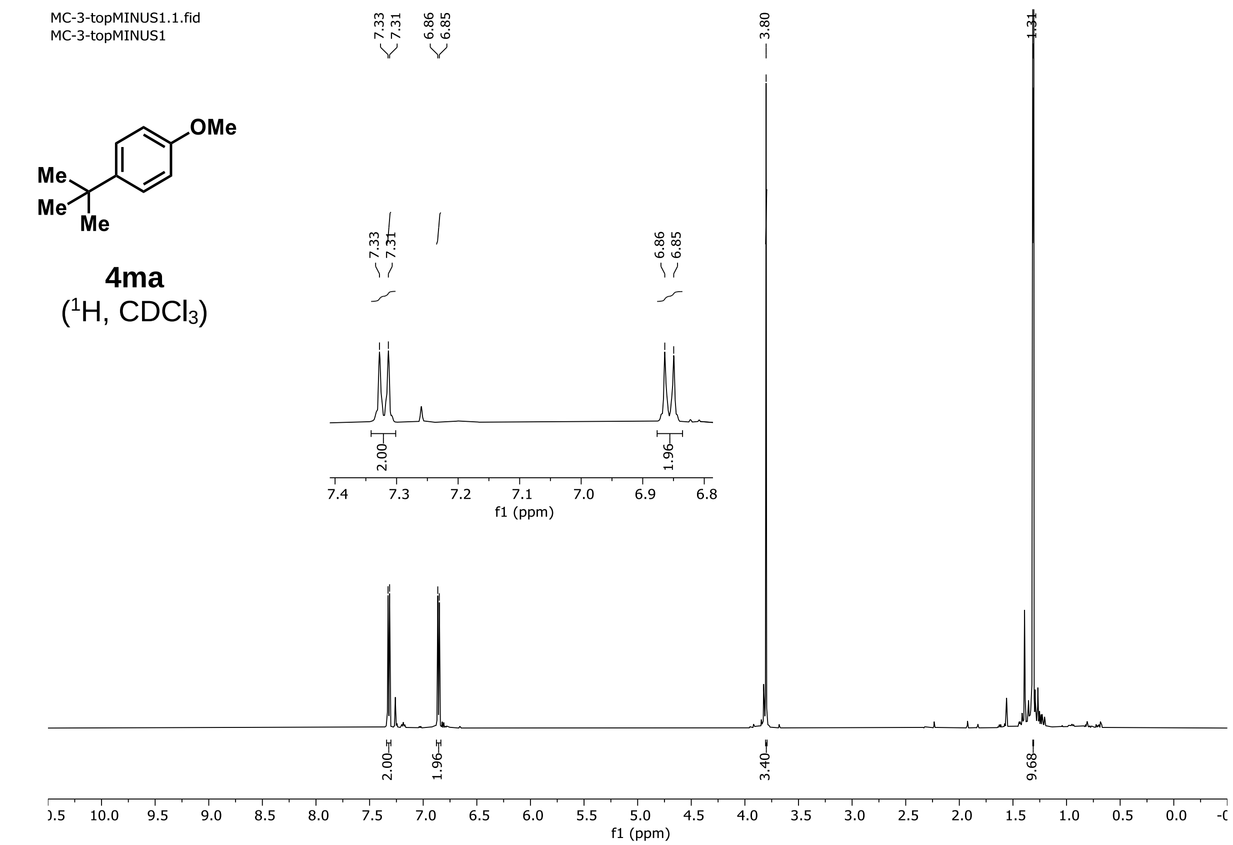

4ma
(^{1}H, $CDCl_3$)

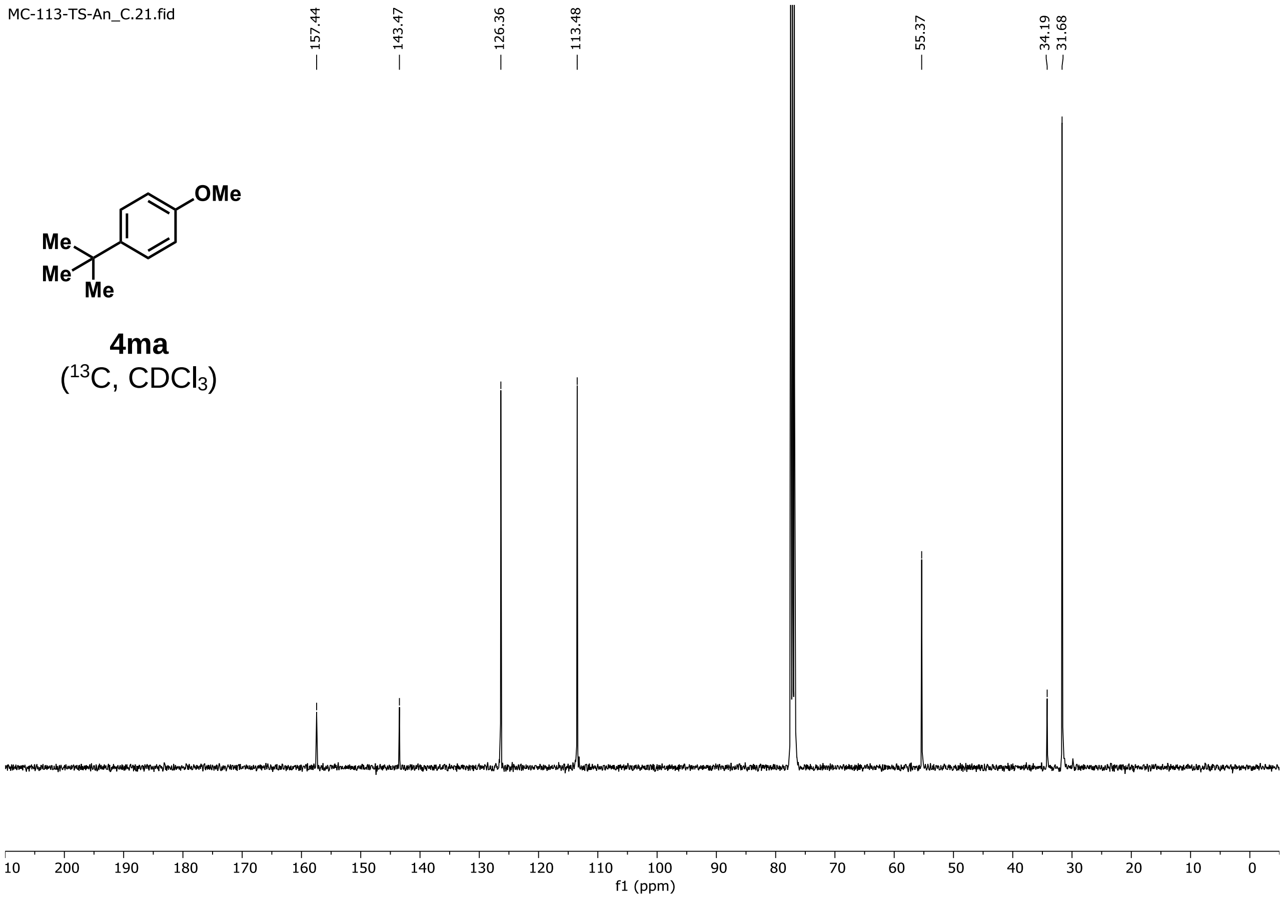

4ma
(^{13}C, $CDCl_3$)

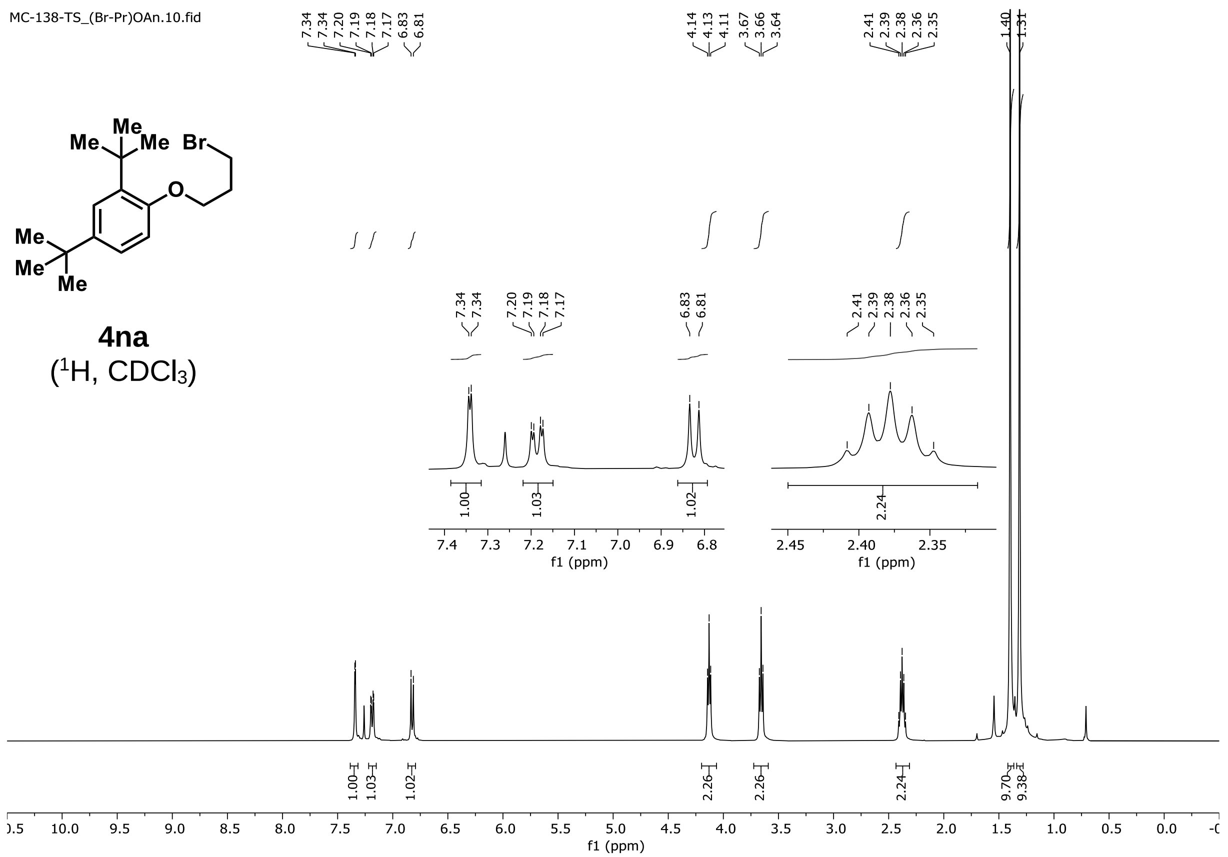
MC-138-TS_(Br-Pr)OAn.10.fid
7.34 7.34 7.20 7.19 7.18 7.17 6.83 6.81
4.14 4.13 4.11 3.67 3.66 3.64
2.41 2.39 2.38 2.36 2.35
1.40 1.31
Me Me Me Br O
Me Me Me
4na
(^{1}H, $CDCl_3$)
1.00 1.03 1.02 2.24
f1 (ppm)
1.00 1.03 1.02 2.26 2.26 2.24 9.70 9.38
f1 (ppm)

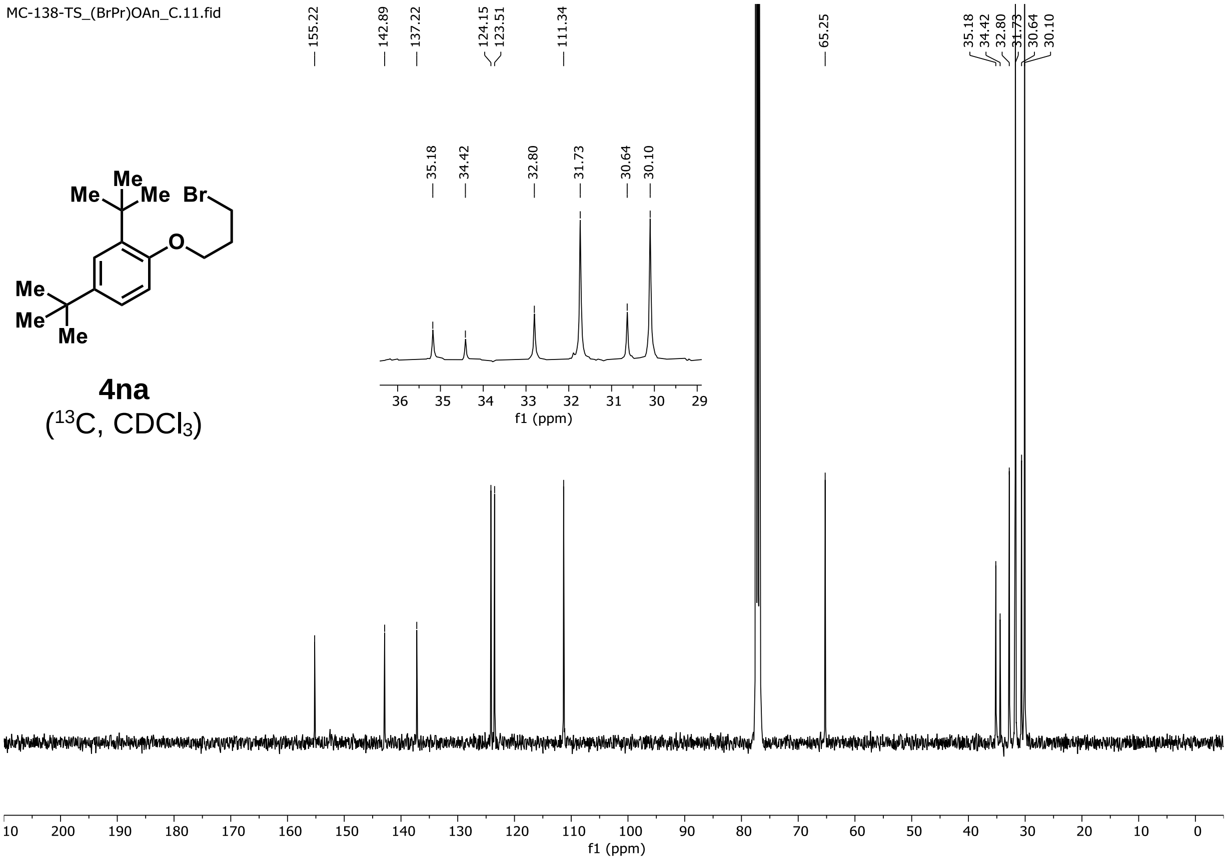
MC-138-TS_(BrPr)OAn_C.11.fid
155.22 142.89 137.22 124.15 123.51 111.34
65.25
35.18 34.42 32.80 31.73 30.64 30.10
Me Me Me Br O
Me Me Me
4na
(^{13}C, $CDCl_3$)
f1 (ppm)
f1 (ppm)

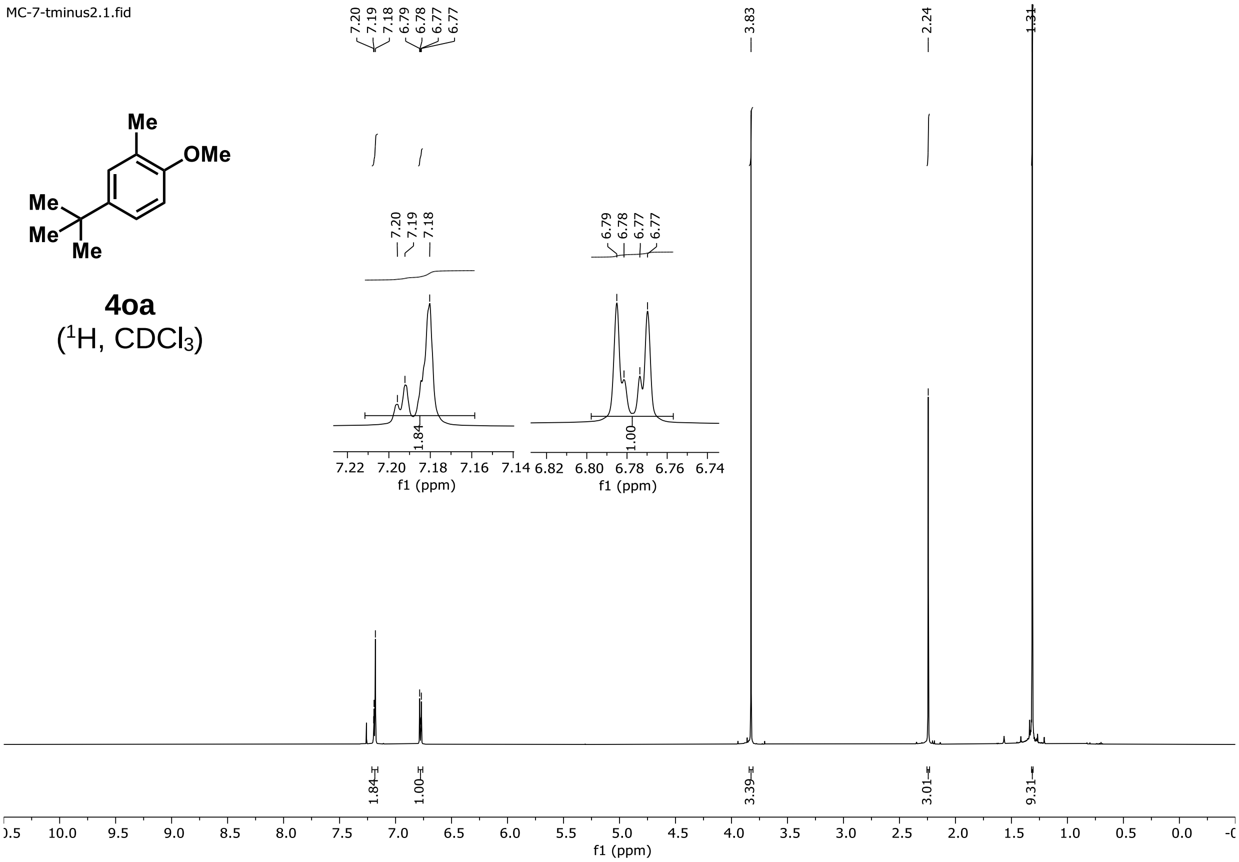
MC-7-tminus2.1.fid
7.20 7.19 7.18 6.79 6.78 6.77 6.77
3.83
2.24
1.31
Me
OMe
Me
Me
Me
4oa
(^{1}H, $CDCl_3$)
1.84
1.00
3.39
3.01
9.31
f1 (ppm)

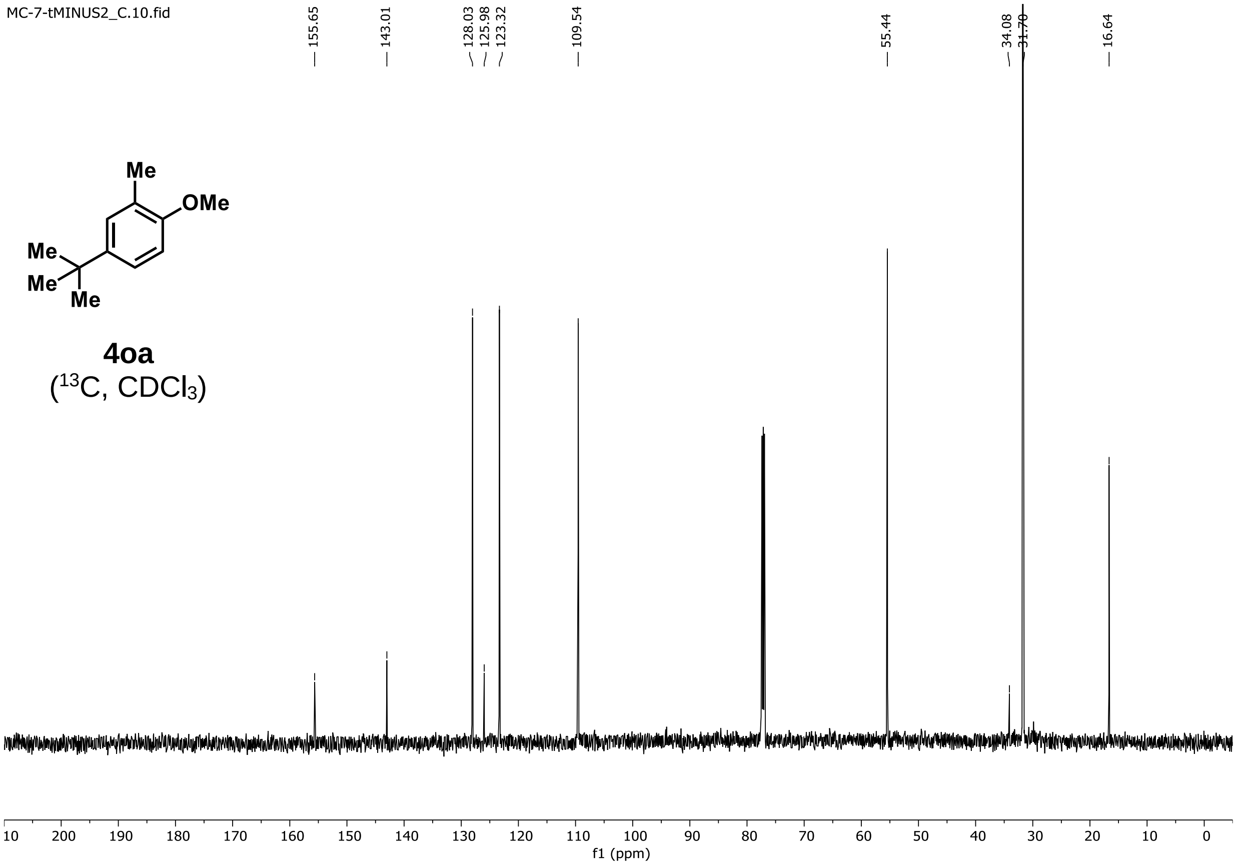
MC-7-tMINUS2_C.10.fid
155.65
143.01
128.03 125.98 123.32
109.54
55.44
34.08 31.70
16.64
Me
OMe
Me
Me
Me
4oa
(^{13}C, $CDCl_3$)
f1 (ppm)

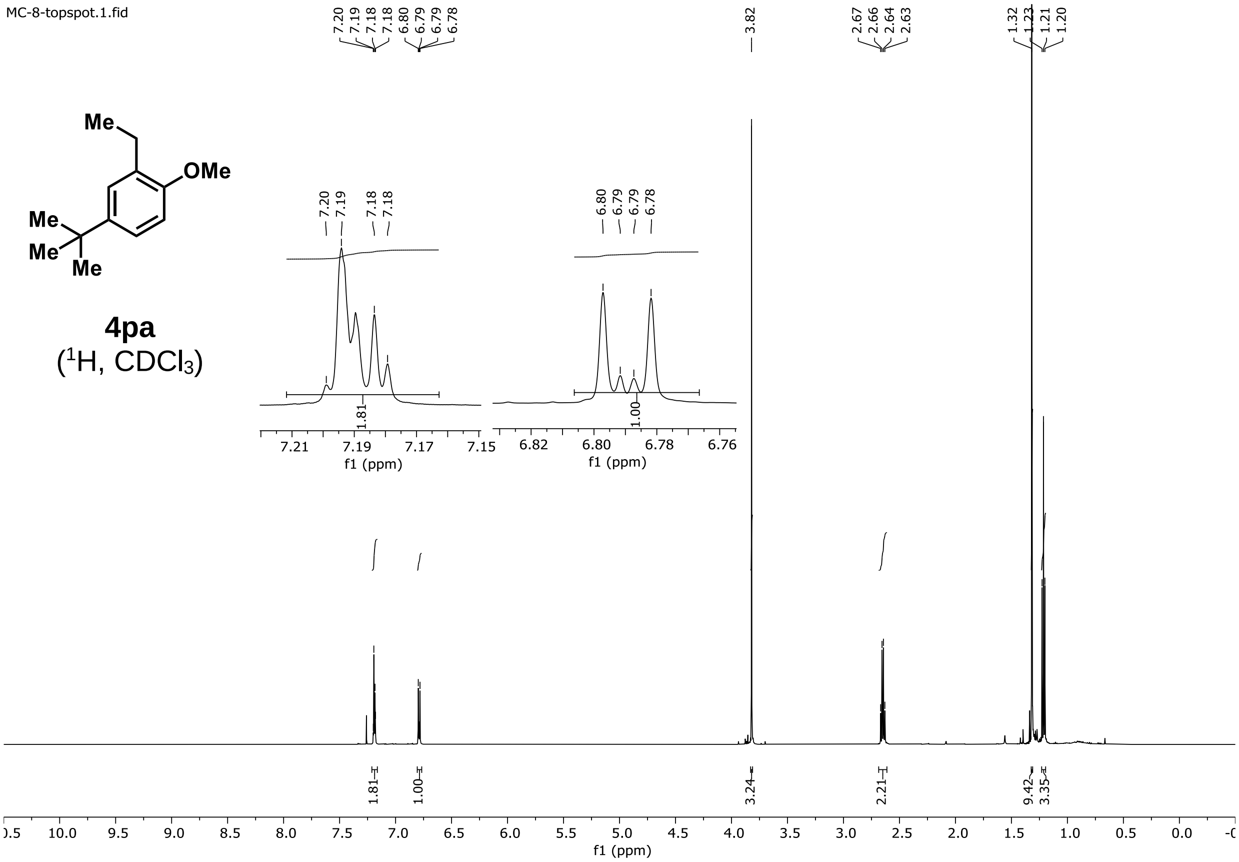
MC-8-topspot.1.fid
7.20
7.19
7.18
7.18
6.80
6.79
6.79
6.78
3.82
2.67
2.66
2.64
2.63
1.32
1.23
1.21
1.20
Me
OMe
Me
Me
Me
4pa
(^{1}H, CDCl$_3$)
1.81
1.00
3.24
2.21
9.42
3.35
f1 (ppm)

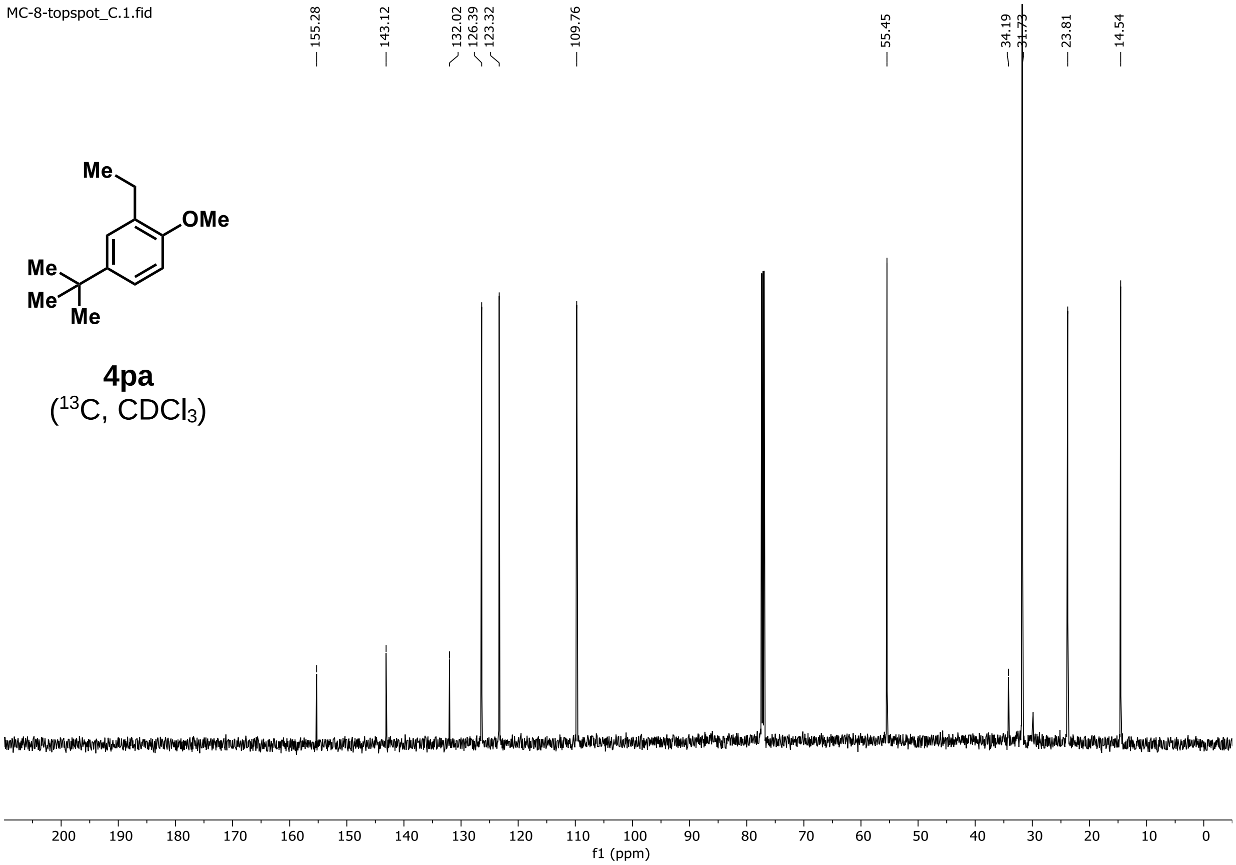
MC-8-topspot_C.1.fid
155.28
143.12
132.02
126.39
123.32
109.76
55.45
34.19
31.73
23.81
14.54
Me
OMe
Me
Me
Me
4pa
(^{13}C, CDCl$_3$)
f1 (ppm)

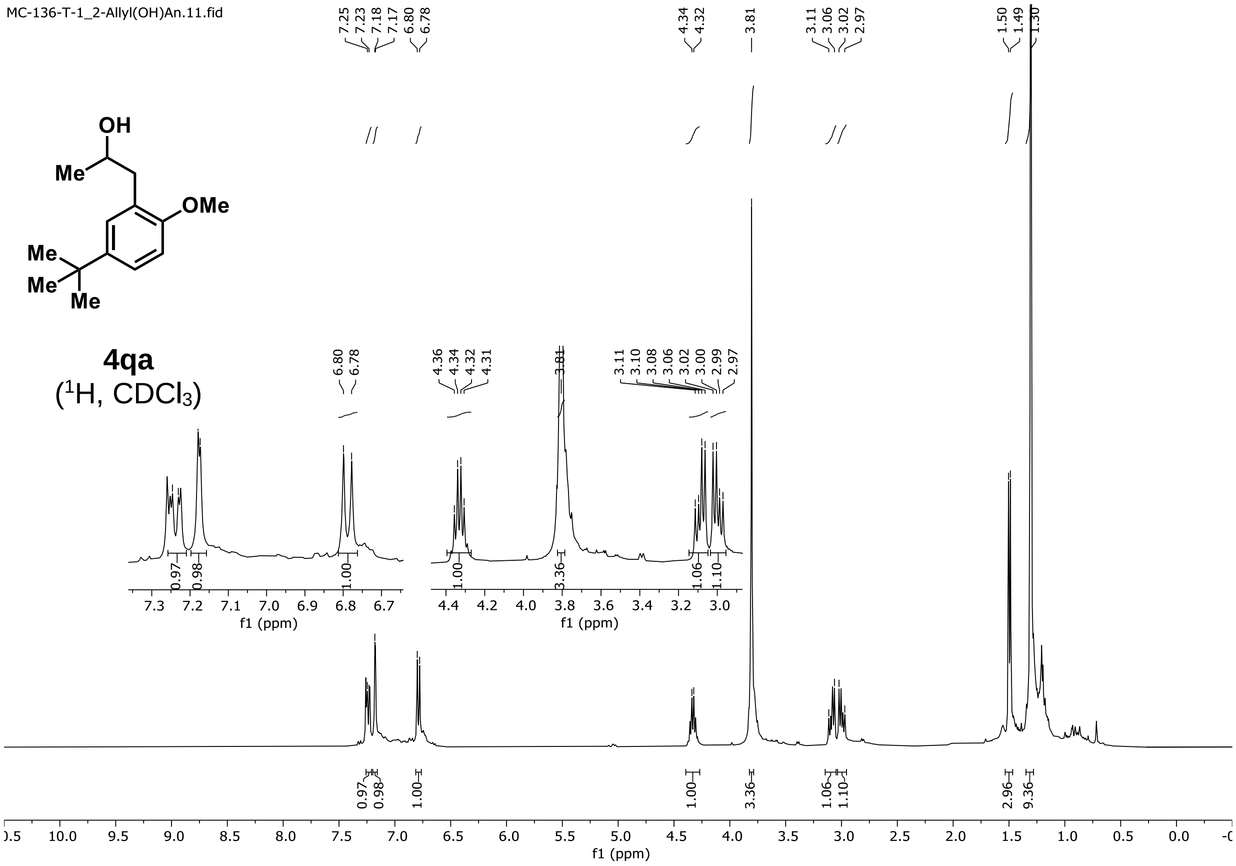

4qa
(^{1}H, $CDCl_3$)

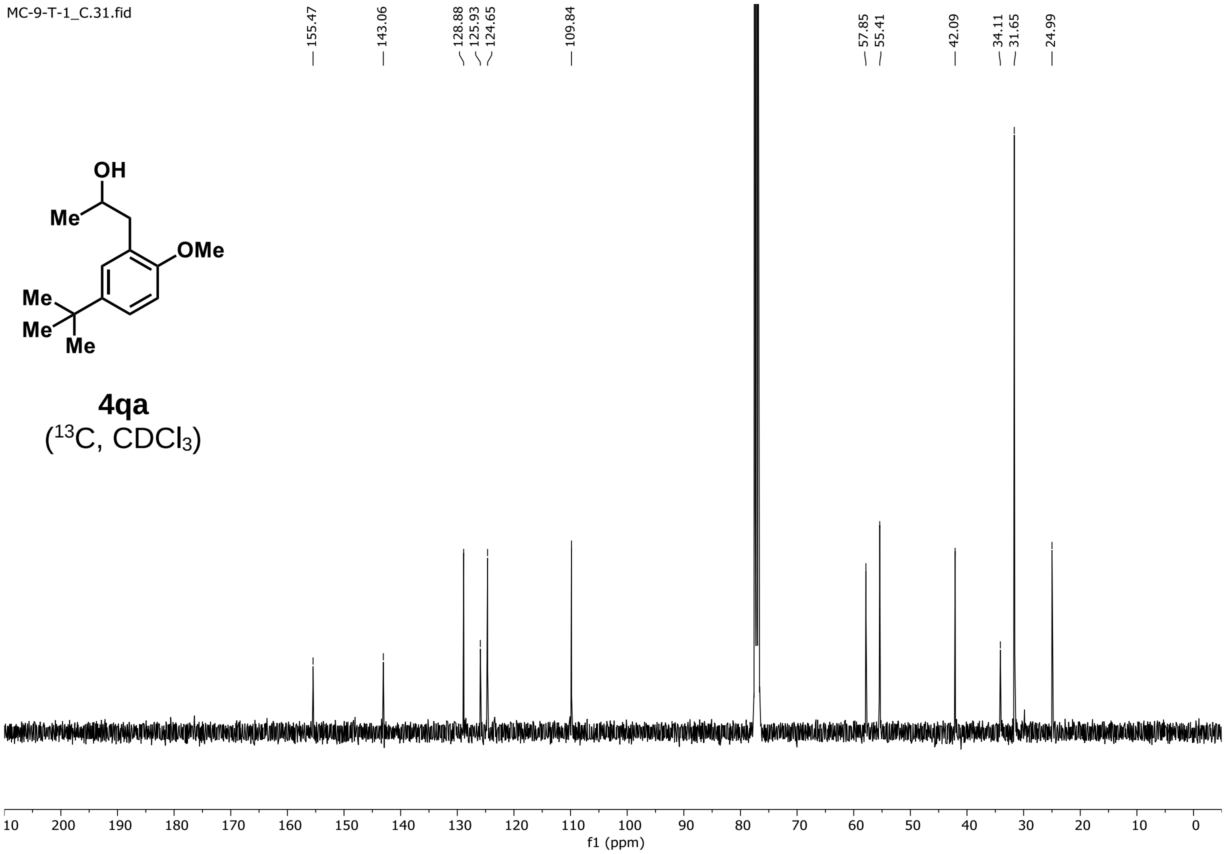

4qa
(^{13}C, $CDCl_3$)

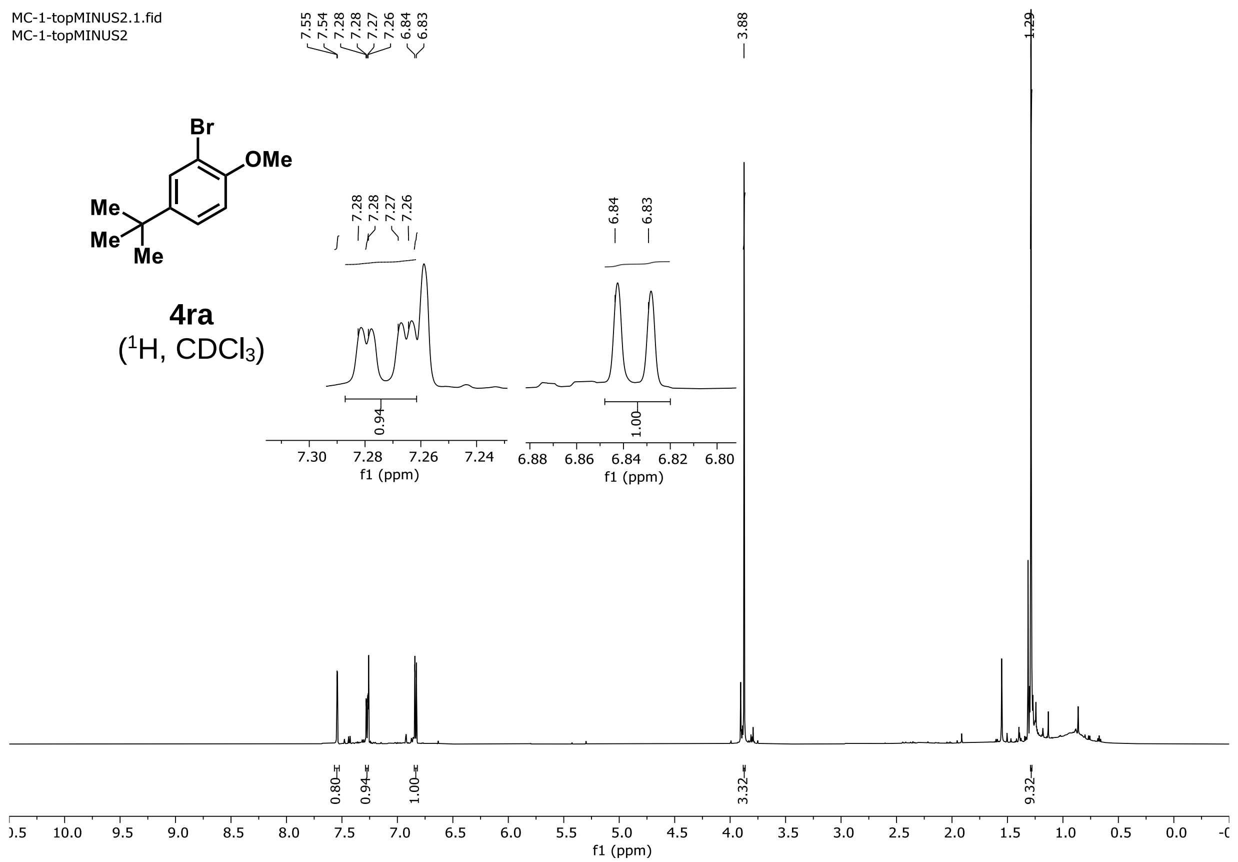
MC-1-topMINUS2.1.fid
MC-1-topMINUS2
7.55
7.54
7.28
7.28
7.27
7.26
6.84
6.83
3.88
1.29
Br
OMe
Me
Me
Me
4ra
(1H, CDCl3)
0.94
1.00
0.80
3.32
9.32
f1 (ppm)

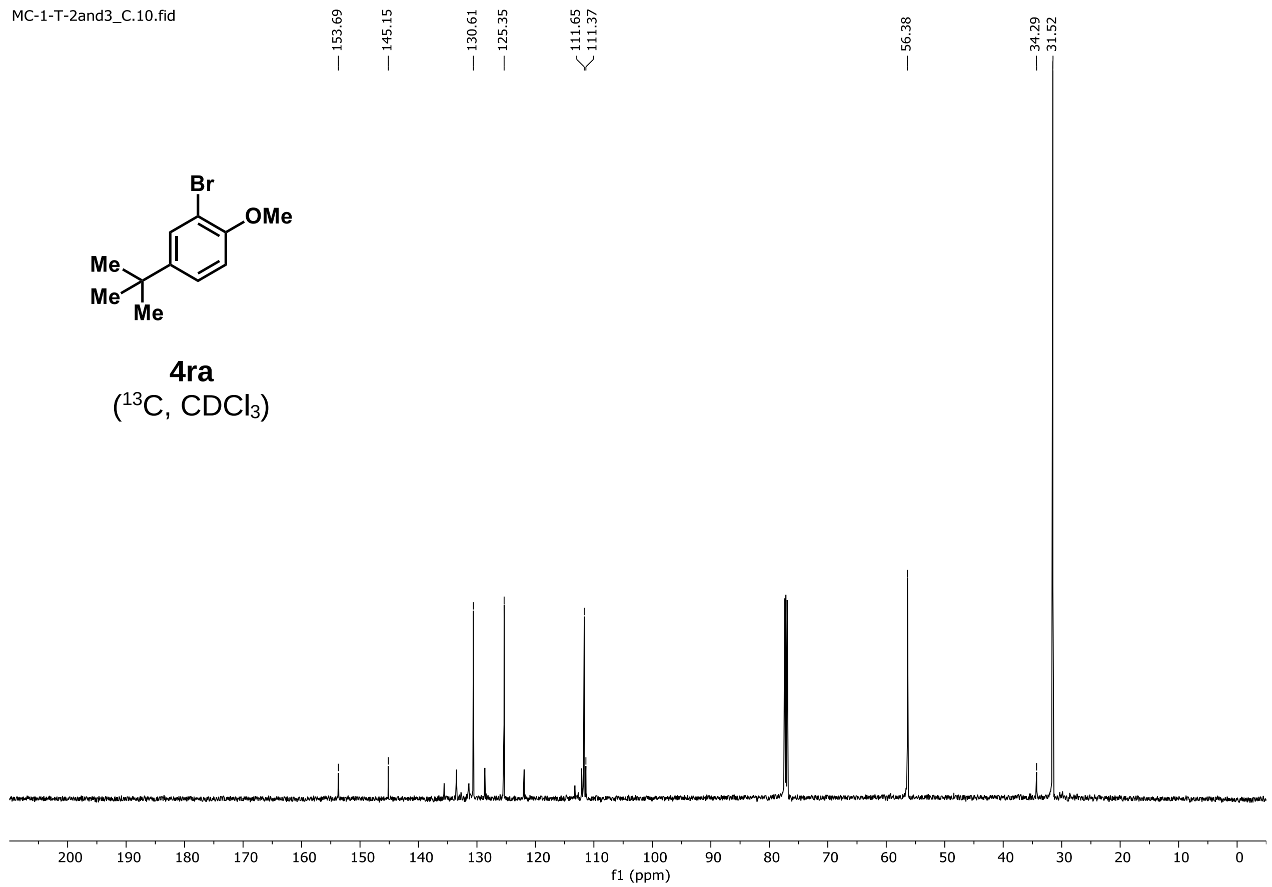
MC-1-T-2and3_C.10.fid
153.69
145.15
130.61
125.35
111.65
111.37
56.38
34.29
31.52
Br
OMe
Me
Me
Me
4ra
(13C, CDCl3)
f1 (ppm)

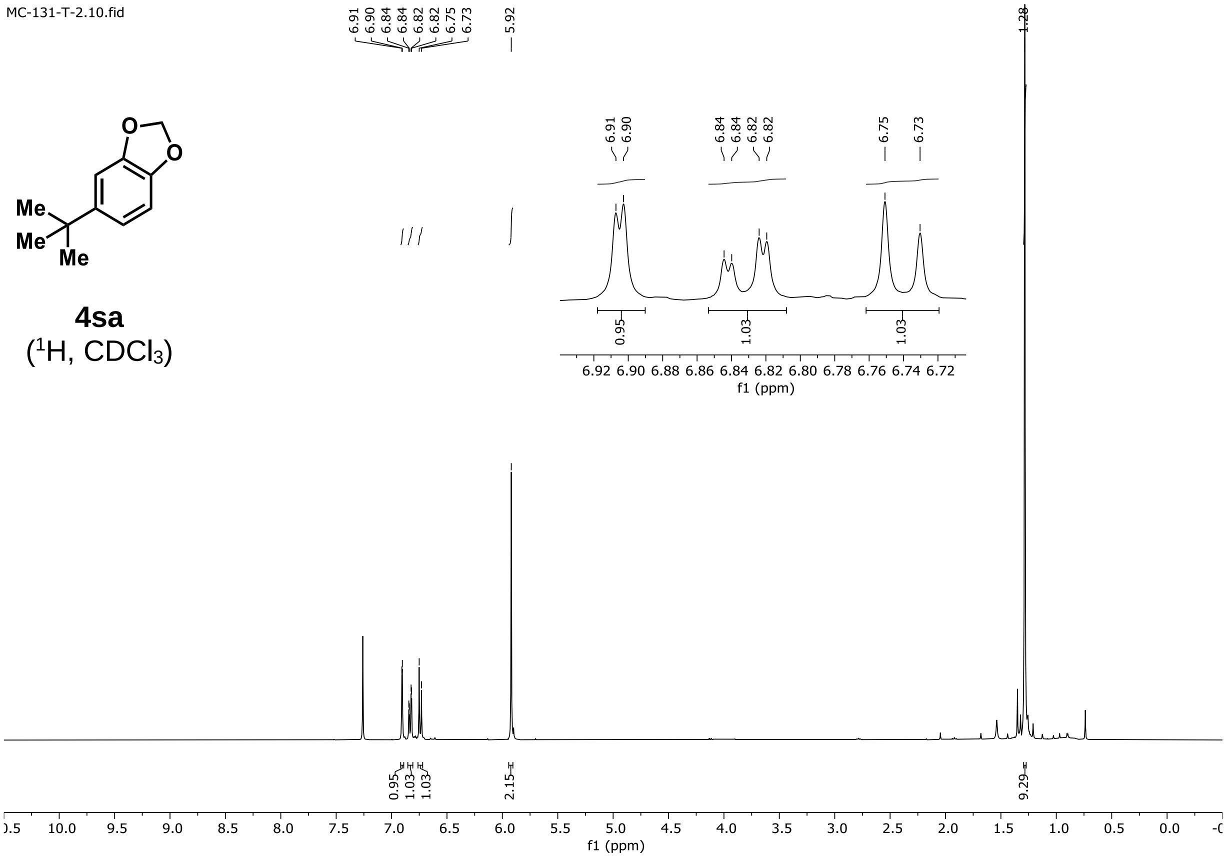

4sa
(^{1}H, $CDCl_3$)

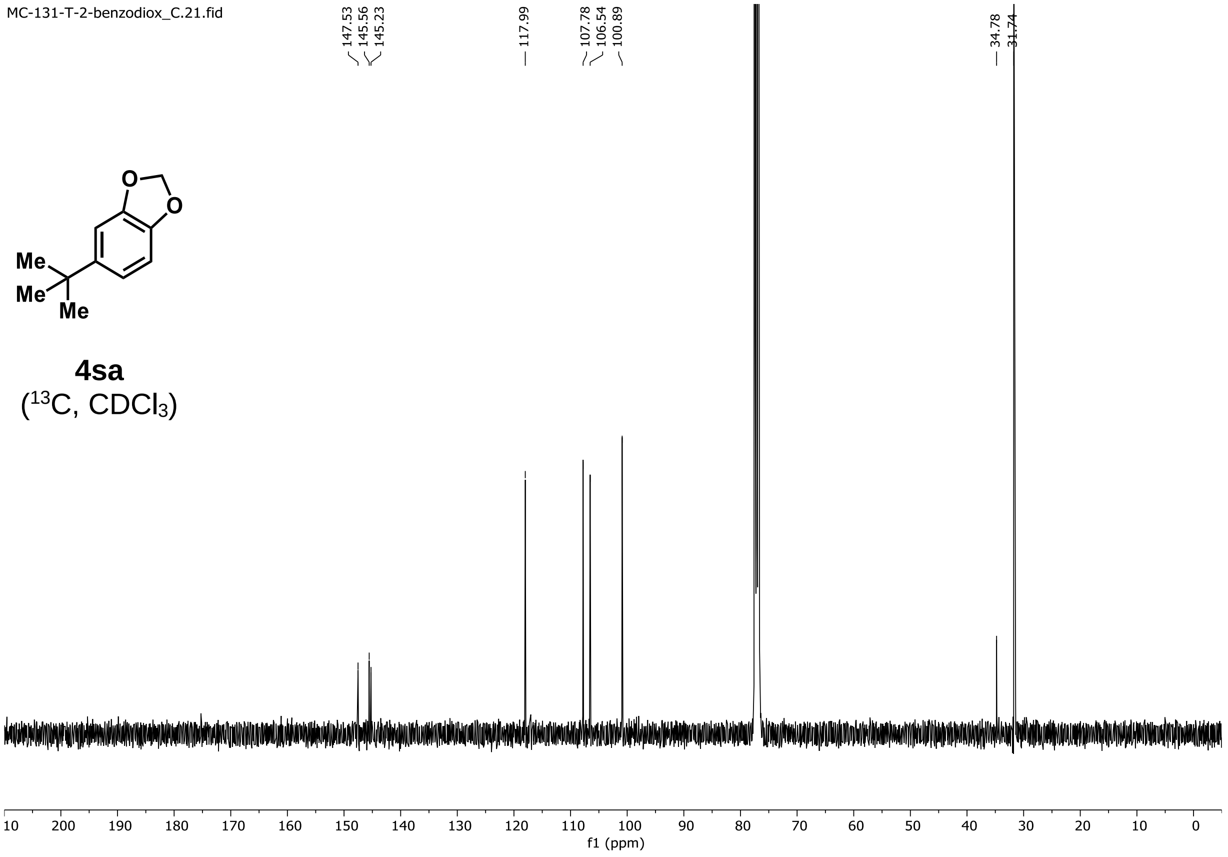

4sa
(^{13}C, $CDCl_3$)

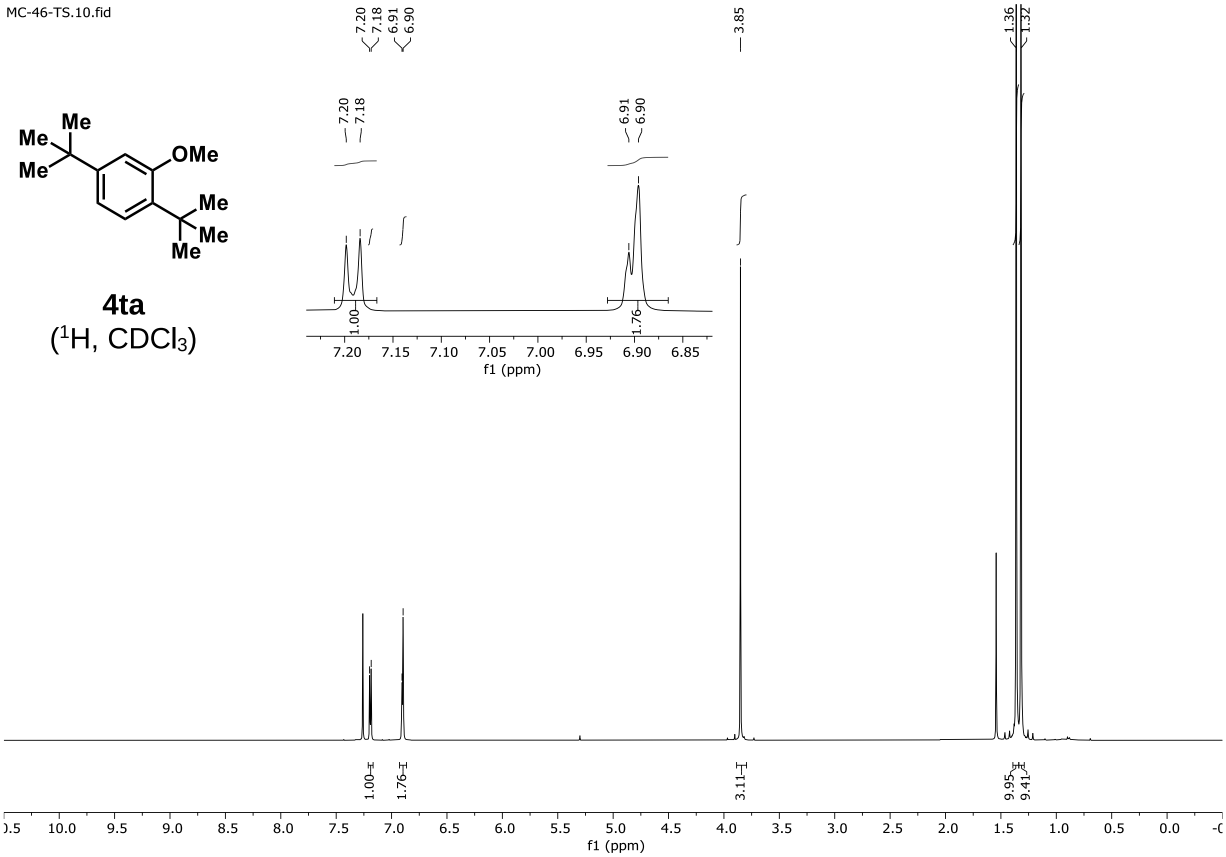
MC-46-TS.10.fid
4ta
(1H, CDCl3)
7.20
7.18
6.91
6.90
3.85
1.36
1.32
1.00
1.76
3.11
9.95
9.41
f1 (ppm)

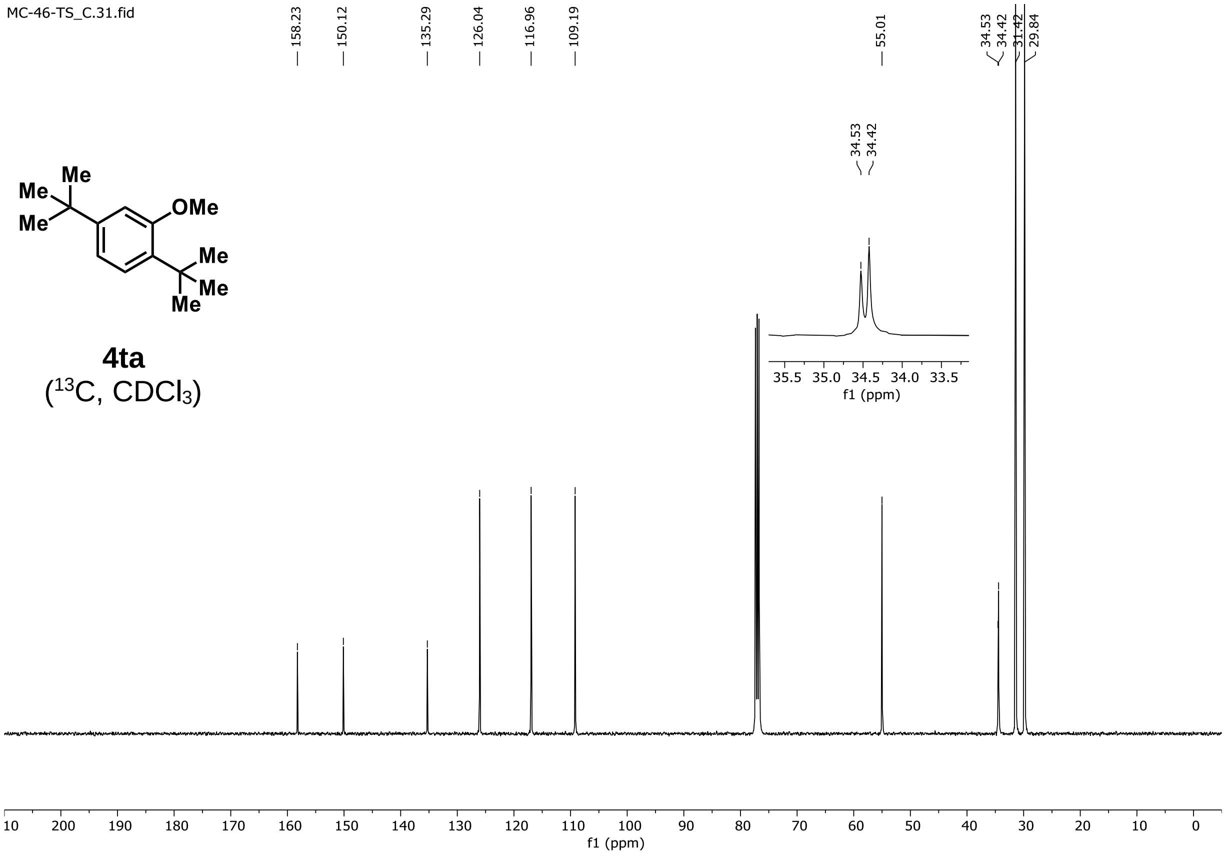
MC-46-TS_C.31.fid
4ta
(13C, CDCl3)
158.23
150.12
135.29
126.04
116.96
109.19
55.01
34.53
34.42
31.42
29.84
f1 (ppm)

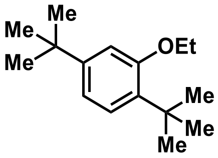

4ua
(^{1}H, $CDCl_3$)

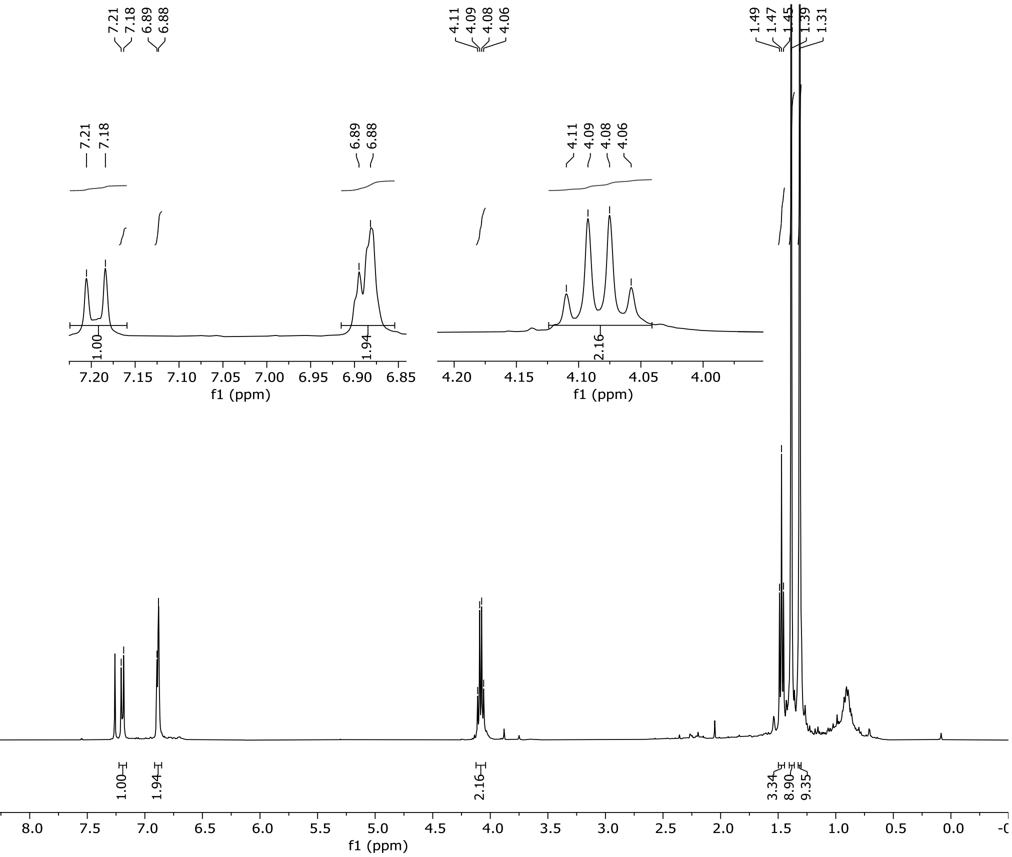

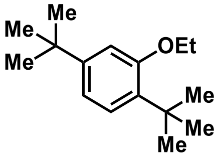

4ua
(^{1}H, $CDCl_3$)

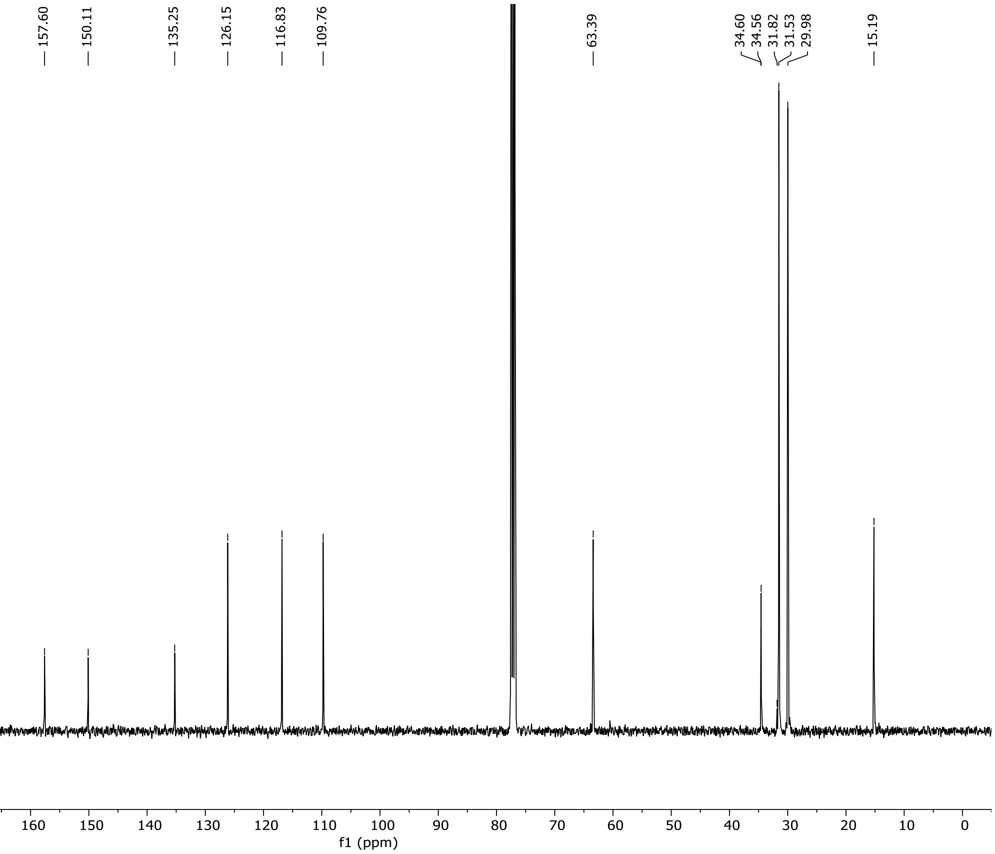

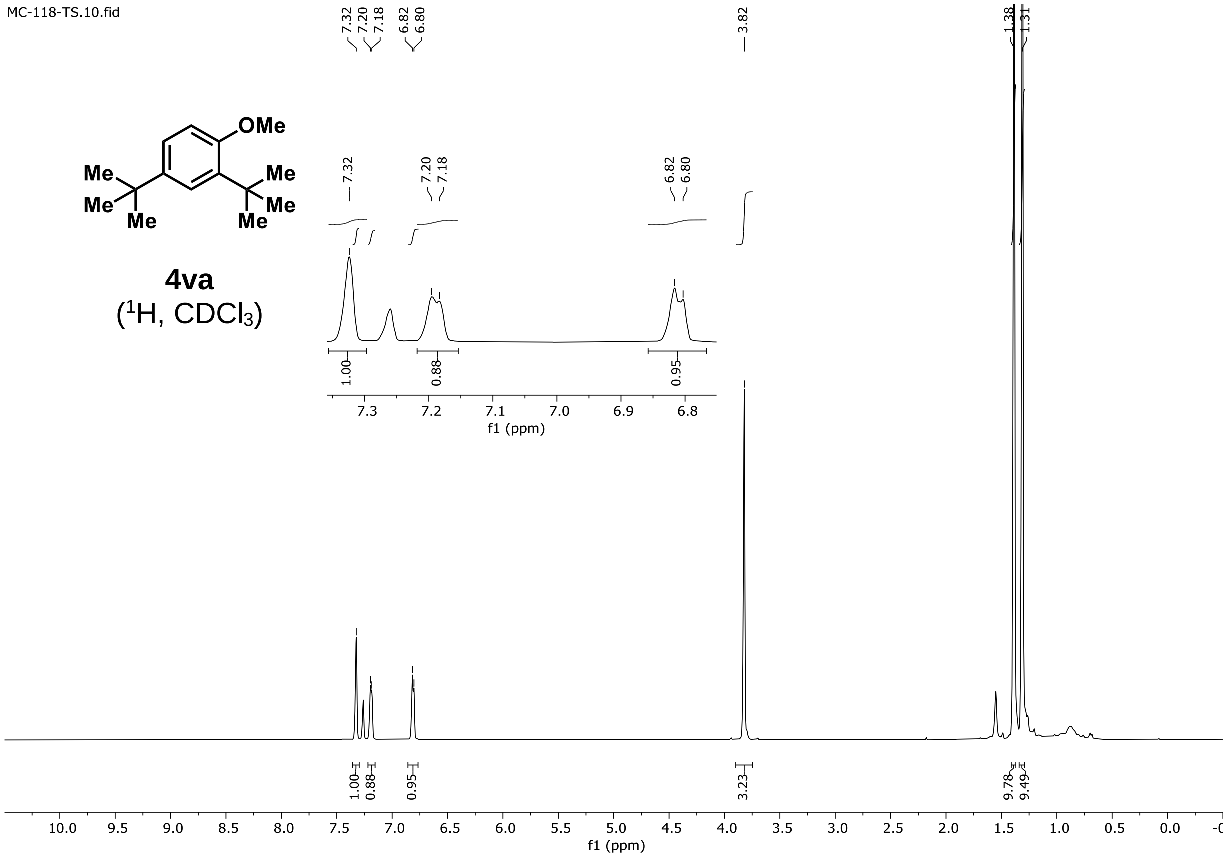
MC-118-TS.10.fid
4va
(^{1}H, $CDCl_3$)
7.32 7.20 7.18 6.82 6.80
3.82
1.38 1.31
1.00 0.88 0.95 3.23 9.78 9.49
f1 (ppm)

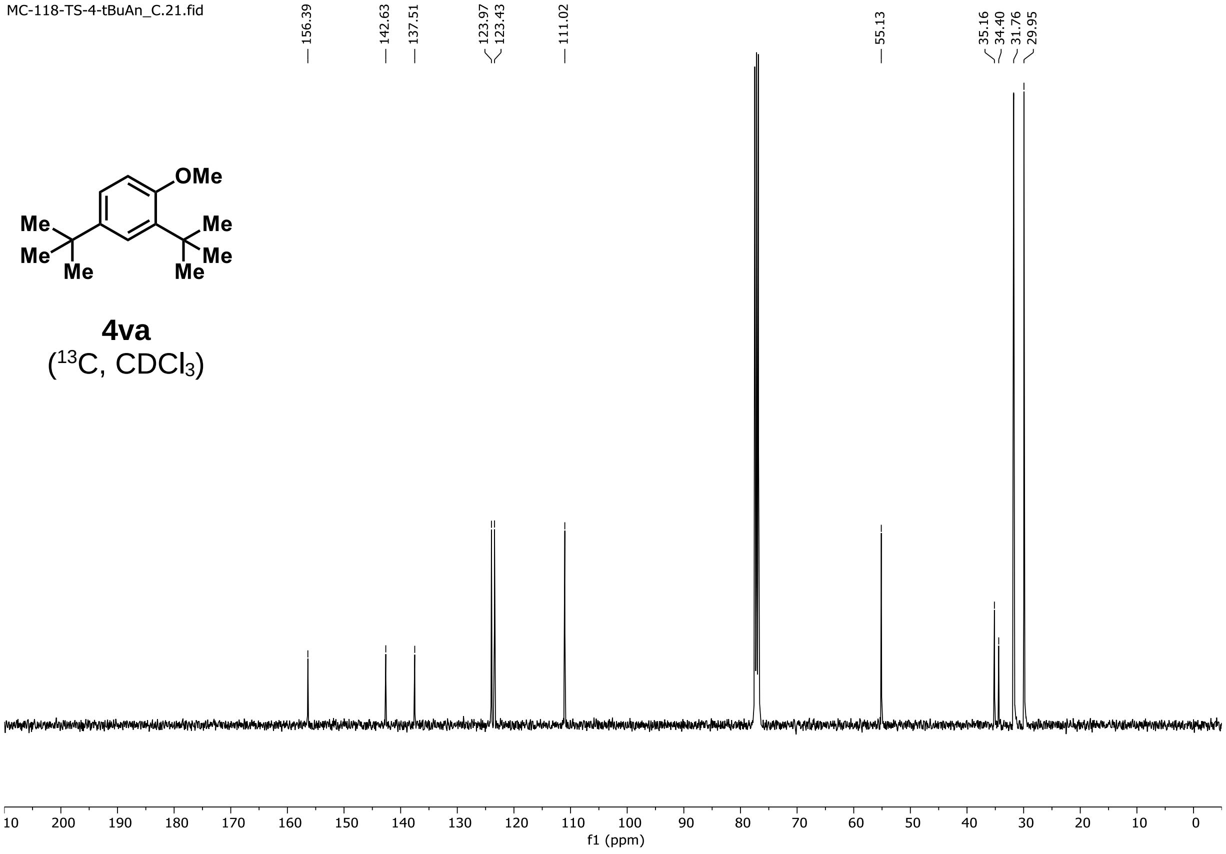
MC-118-TS-4-tBuAn_C.21.fid
4va
(^{13}C, $CDCl_3$)
156.39 142.63 137.51 123.97 123.43 111.02
55.13
35.16 34.40 31.76 29.95
f1 (ppm)

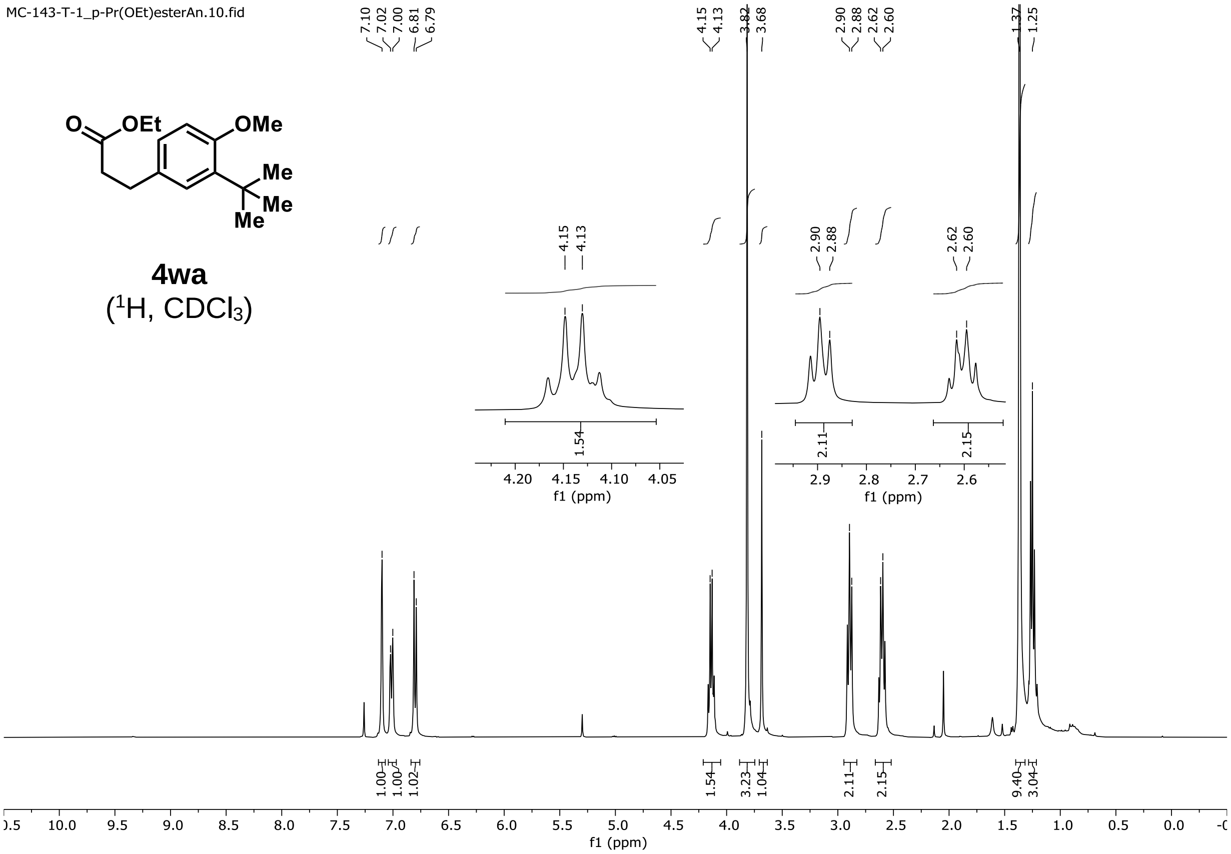

4wa
(^{1}H, $CDCl_3$)

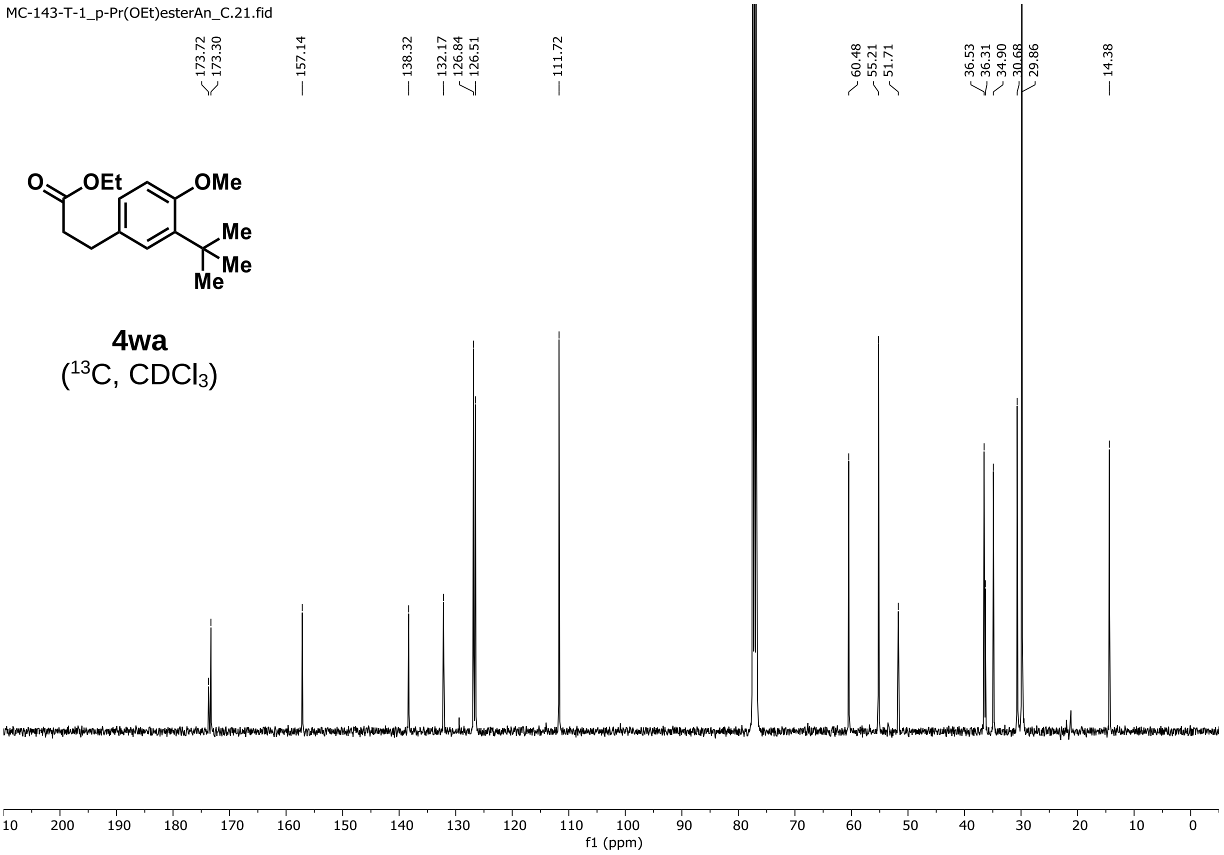

4wa
(^{13}C, $CDCl_3$)

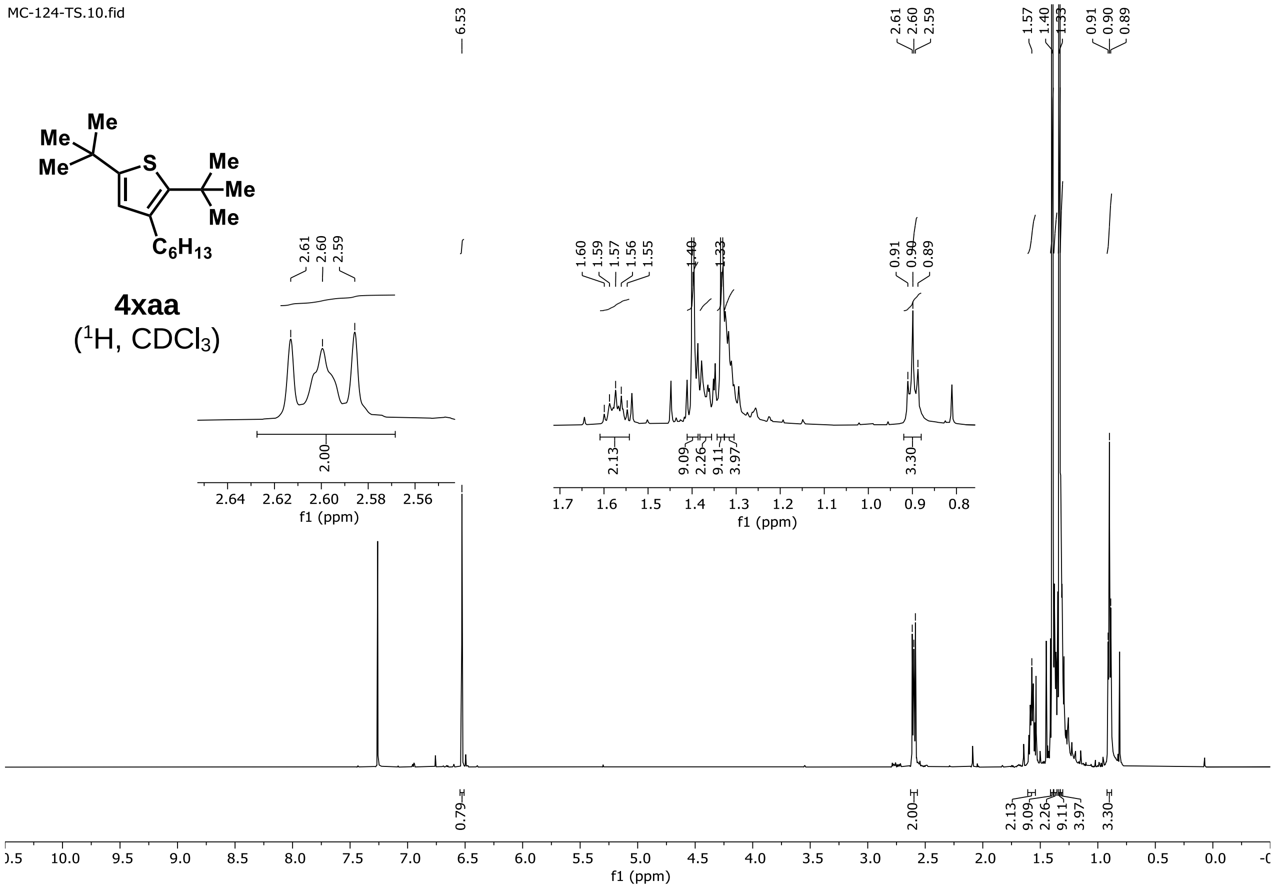

4xaa
(^{1}H, $CDCl_3$)

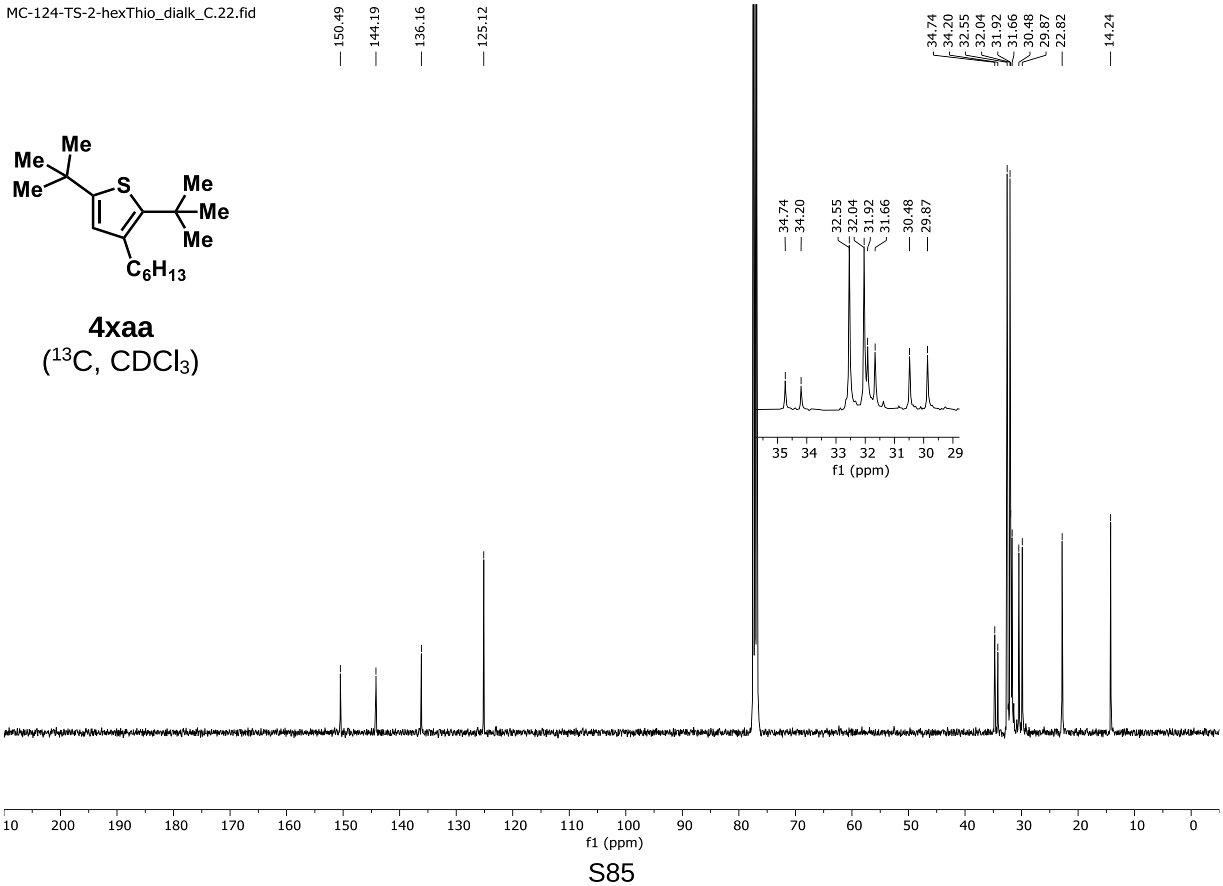

4xaa
(^{13}C, $CDCl_3$)

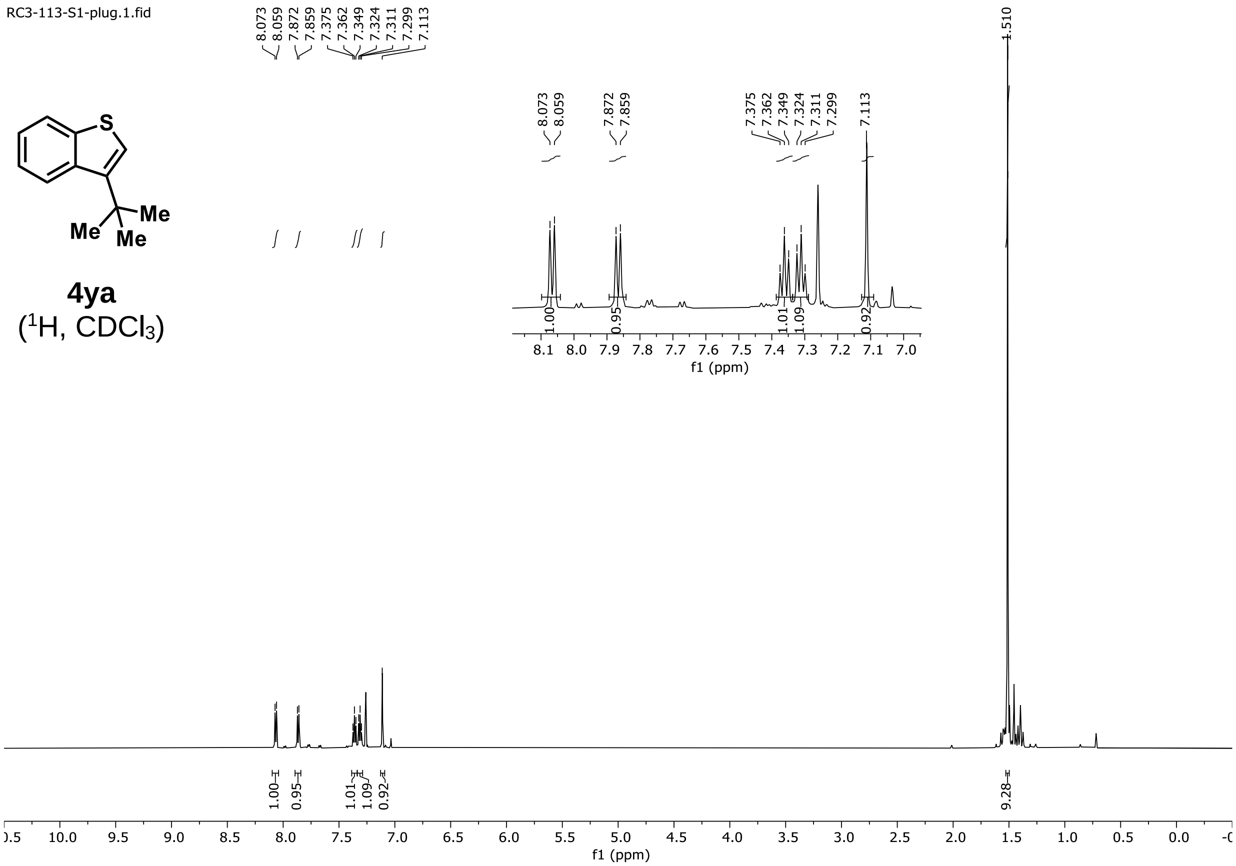
RC3-113-S1-plug.1.fid
4ya
(1H, CDCl3)
8.073
8.059
7.872
7.859
7.375
7.362
7.349
7.324
7.311
7.299
7.113
1.510
1.00
0.95
1.01
1.09
0.92
9.28
f1 (ppm)

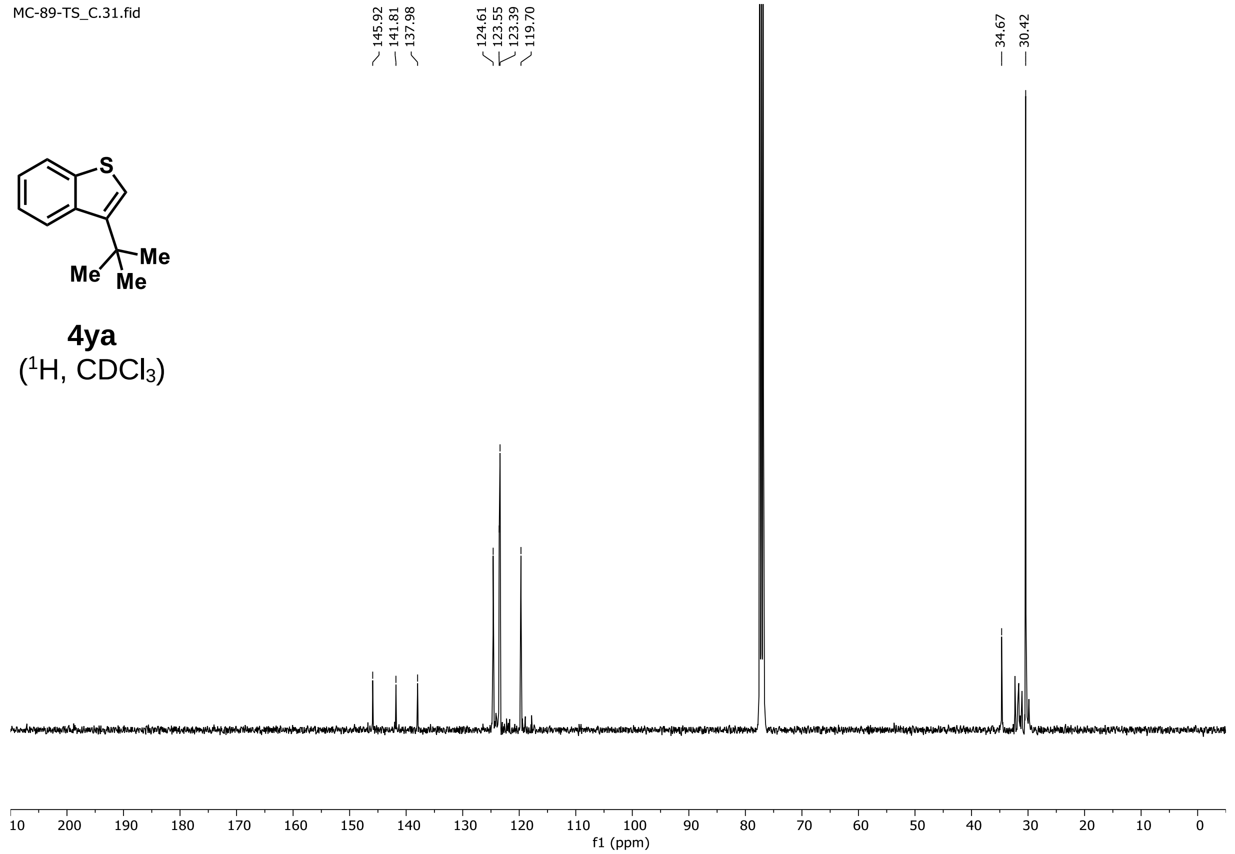
MC-89-TS_C.31.fid
4ya
(1H, CDCl3)
145.92
141.81
137.98
124.61
123.55
123.39
119.70
34.67
30.42
f1 (ppm)

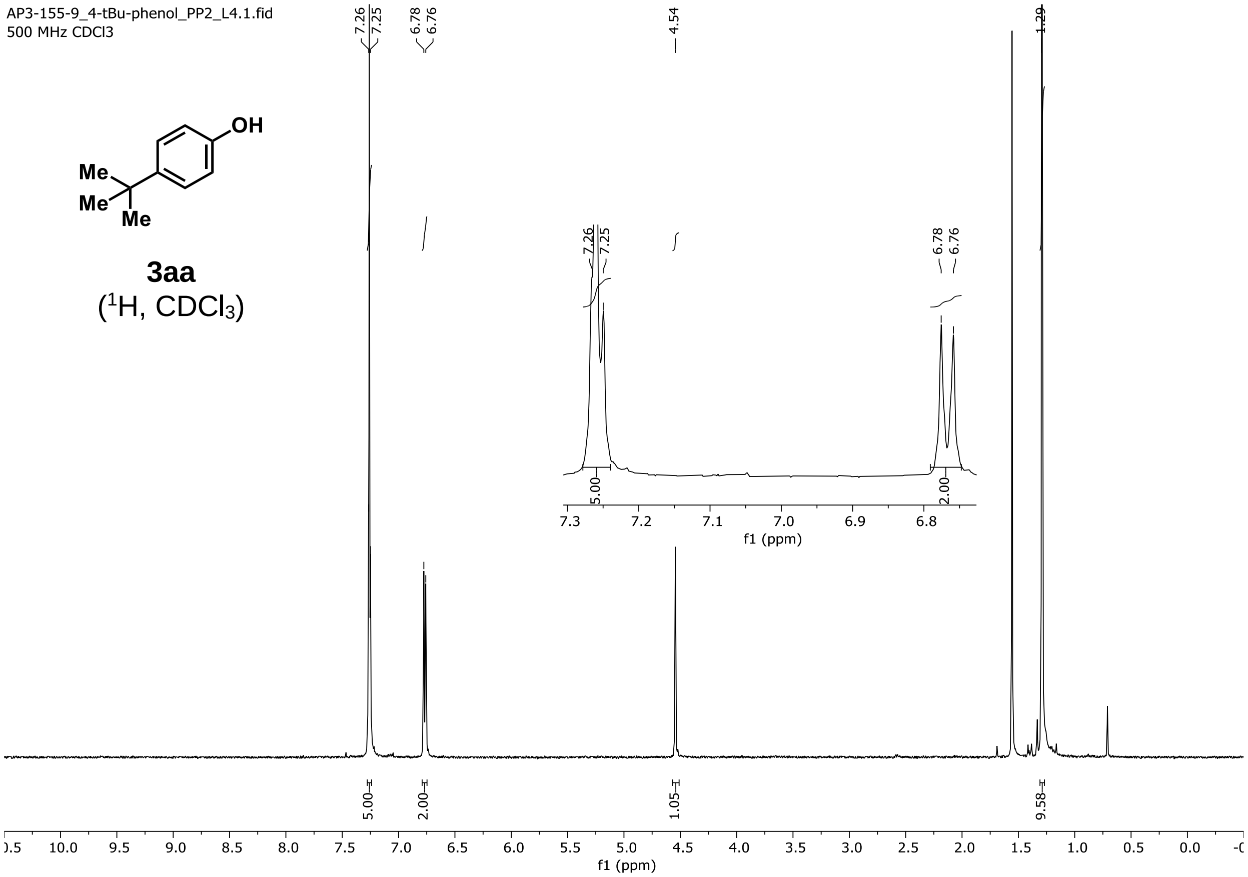
AP3-155-9_4-tBu-phenol_PP2_L4.1.fid
500 MHz CDCl3
OH
Me
Me
Me
3aa
(1H, CDCl3)
7.26
7.25
6.78
6.76
4.54
1.29
5.00
2.00
1.05
9.58
f1 (ppm)

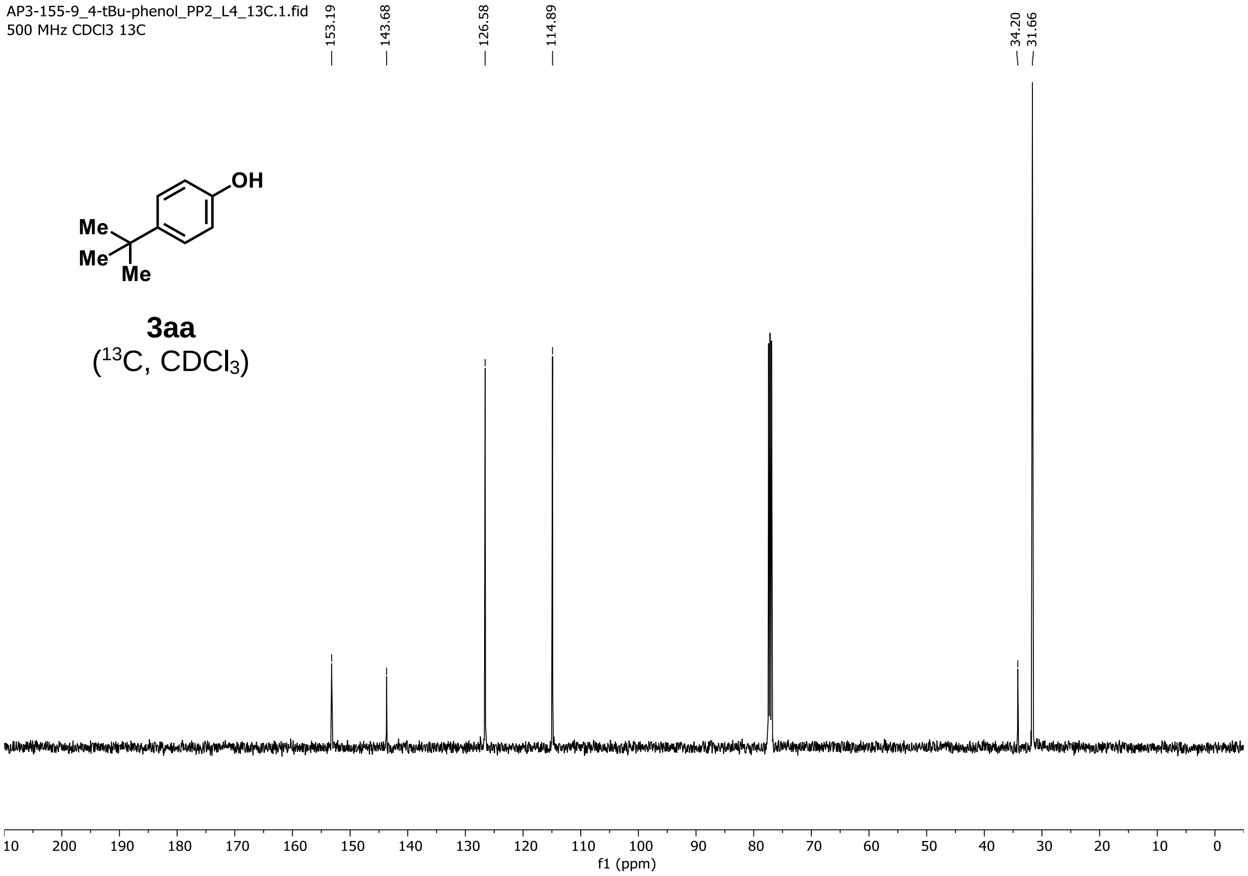
AP3-155-9_4-tBu-phenol_PP2_L4_13C.1.fid
500 MHz CDCl3 13C
153.19
143.68
126.58
114.89
34.20
31.66
OH
Me
Me
Me
3aa
(13C, CDCl3)
f1 (ppm)

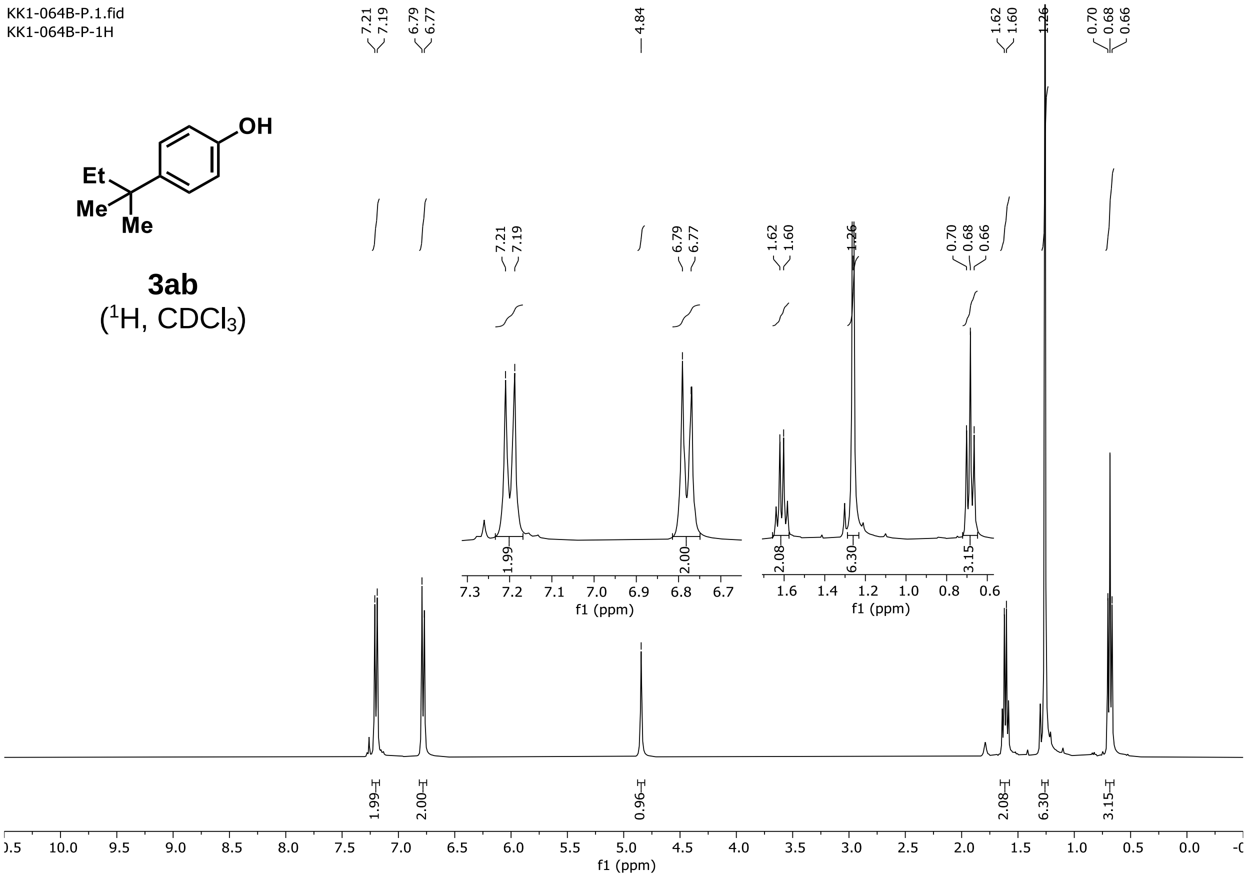
KK1-064B-P.1.fid
KK1-064B-P-1H
7.21
7.19
6.79
6.77
4.84
1.62
1.60
1.26
0.70
0.68
0.66
OH
Et
Me
Me
3ab
(^{1}H, $CDCl_3$)
1.99
2.00
2.08
6.30
3.15
0.96
f1 (ppm)

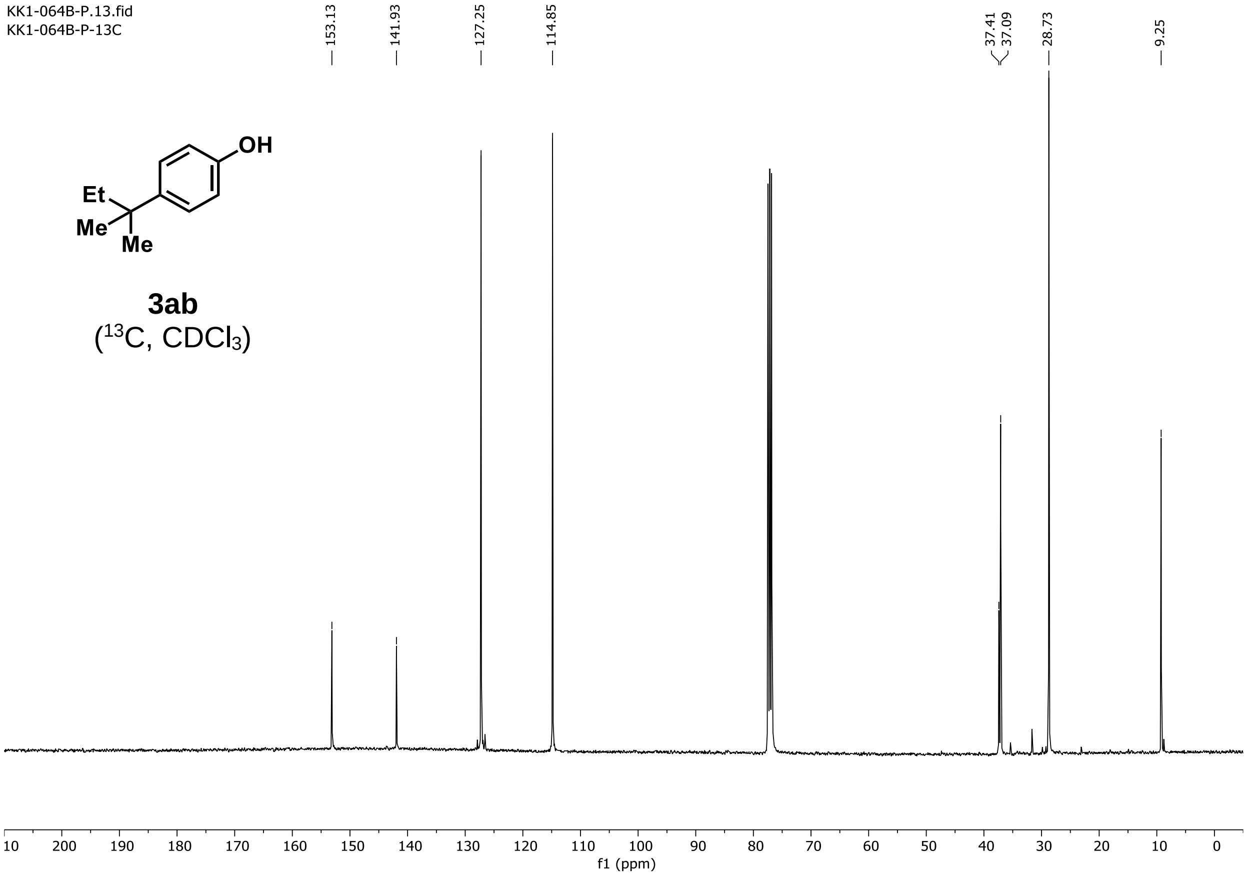
KK1-064B-P.13.fid
KK1-064B-P-13C
153.13
141.93
127.25
114.85
37.41
37.09
28.73
9.25
OH
Et
Me
Me
3ab
(^{13}C, $CDCl_3$)
f1 (ppm)

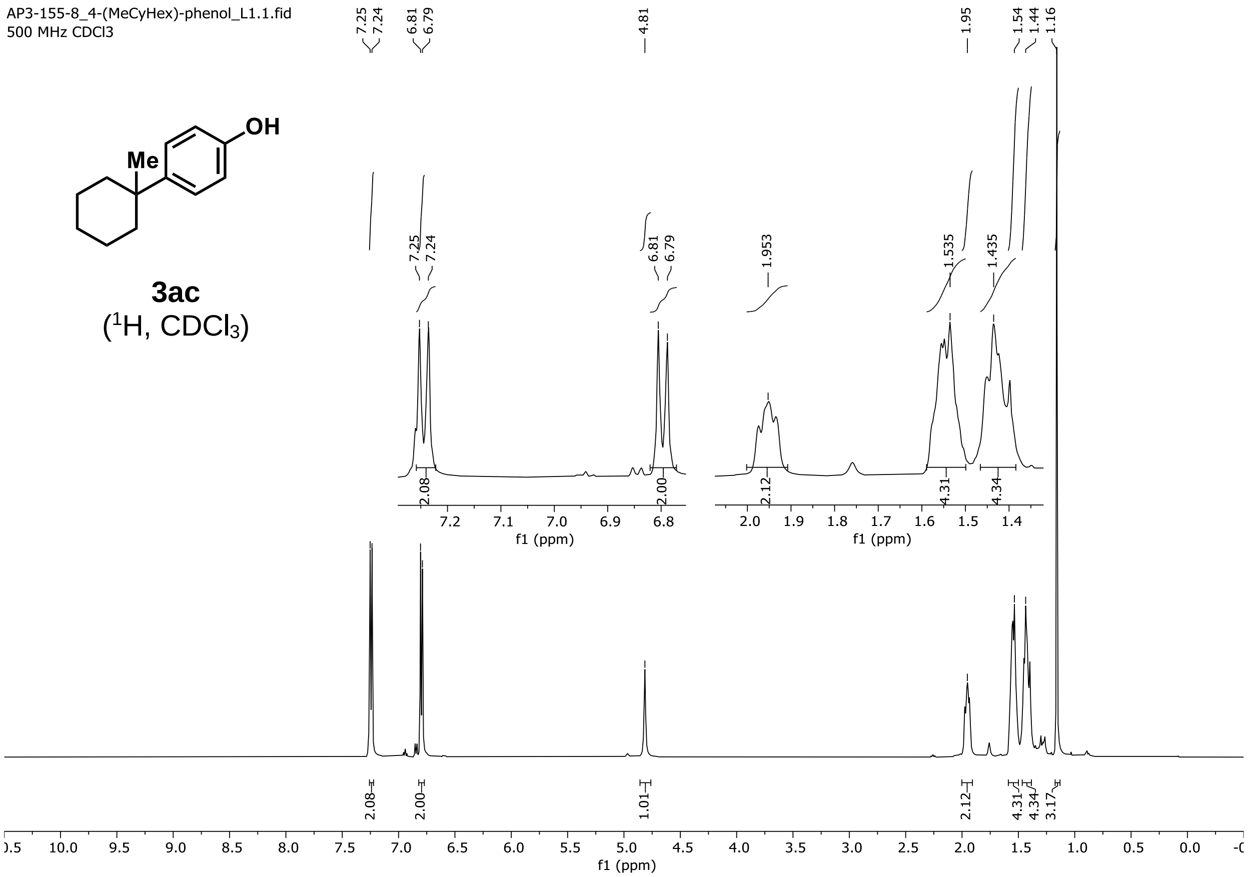
AP3-155-8_4-(MeCyHex)-phenol_L1.1.fid
500 MHz CDCl3
7.25
7.24
6.81
6.79
4.81
1.95
1.54
1.44
1.16
1.953
1.535
1.435
Me
OH
3ac
(1H, CDCl3)
2.08
2.00
2.12
4.31
4.34
1.01
3.17
f1 (ppm)

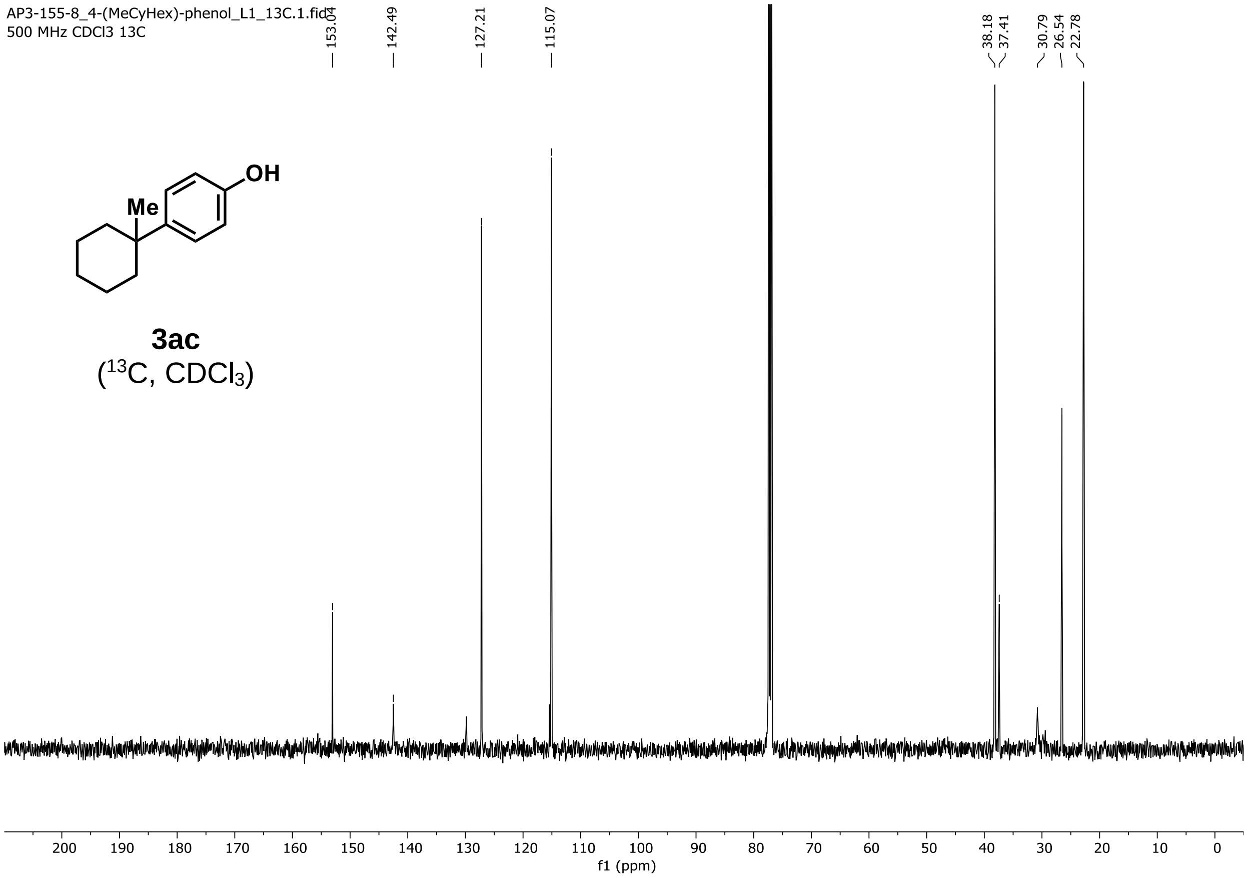
AP3-155-8_4-(MeCyHex)-phenol_L1_13C.1.fid
500 MHz CDCl3 13C
153.04
142.49
127.21
115.07
38.18
37.41
30.79
26.54
22.78
Me
OH
3ac
(13C, CDCl3)
f1 (ppm)

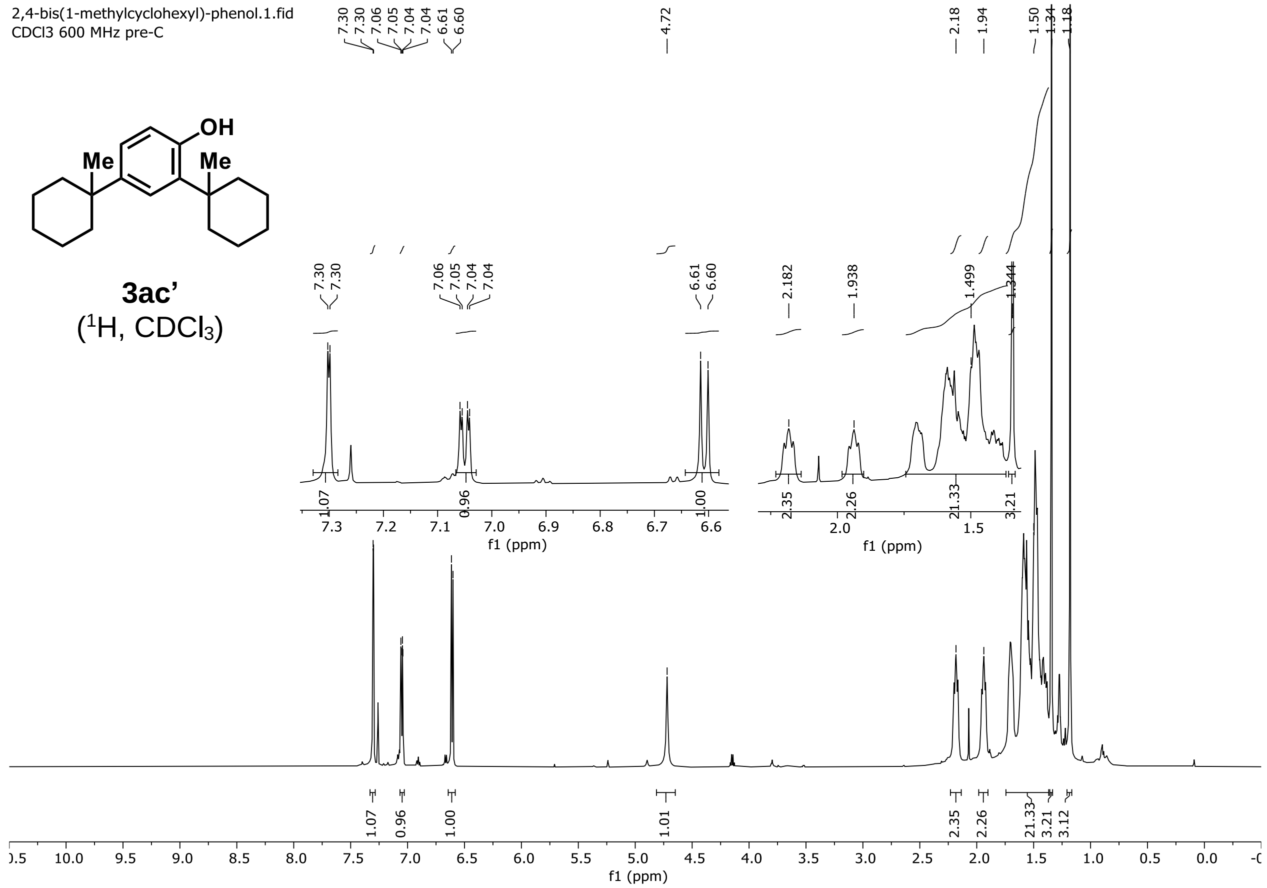
2,4-bis(1-methylcyclohexyl)-phenol.1.fid
CDCl3 600 MHz pre-C
7.30 7.30 7.06 7.05 7.04 7.04 6.61 6.60
4.72
2.18 1.94 1.50 1.34 1.18
OH
Me
Me
3ac’
(1H, CDCl3)
1.07 0.96 1.00 1.01 2.35 2.26 21.33 3.21 3.12
f1 (ppm)

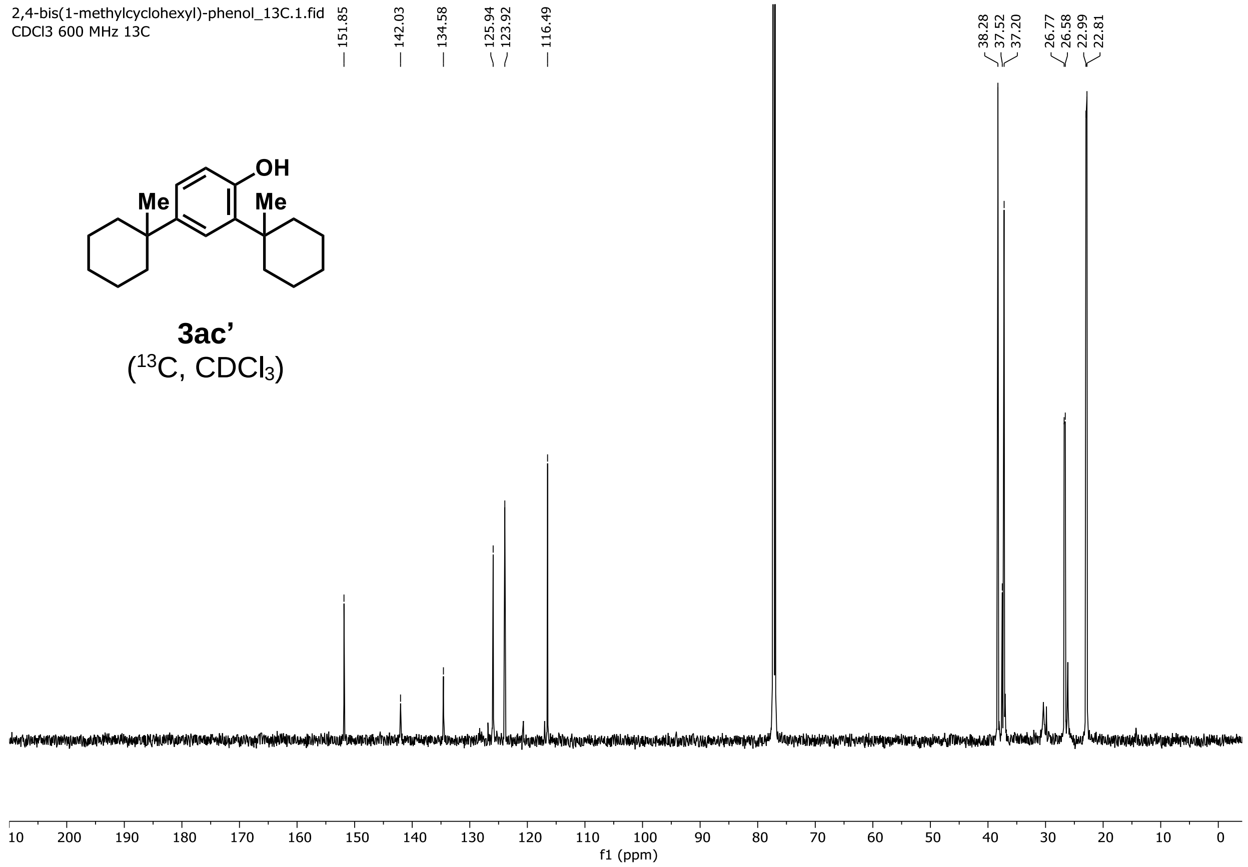
2,4-bis(1-methylcyclohexyl)-phenol_13C.1.fid
CDCl3 600 MHz 13C
151.85 142.03 134.58 125.94 123.92 116.49
38.28 37.52 37.20 26.77 26.58 22.99 22.81
OH
Me
Me
3ac’
(13C, CDCl3)
f1 (ppm)

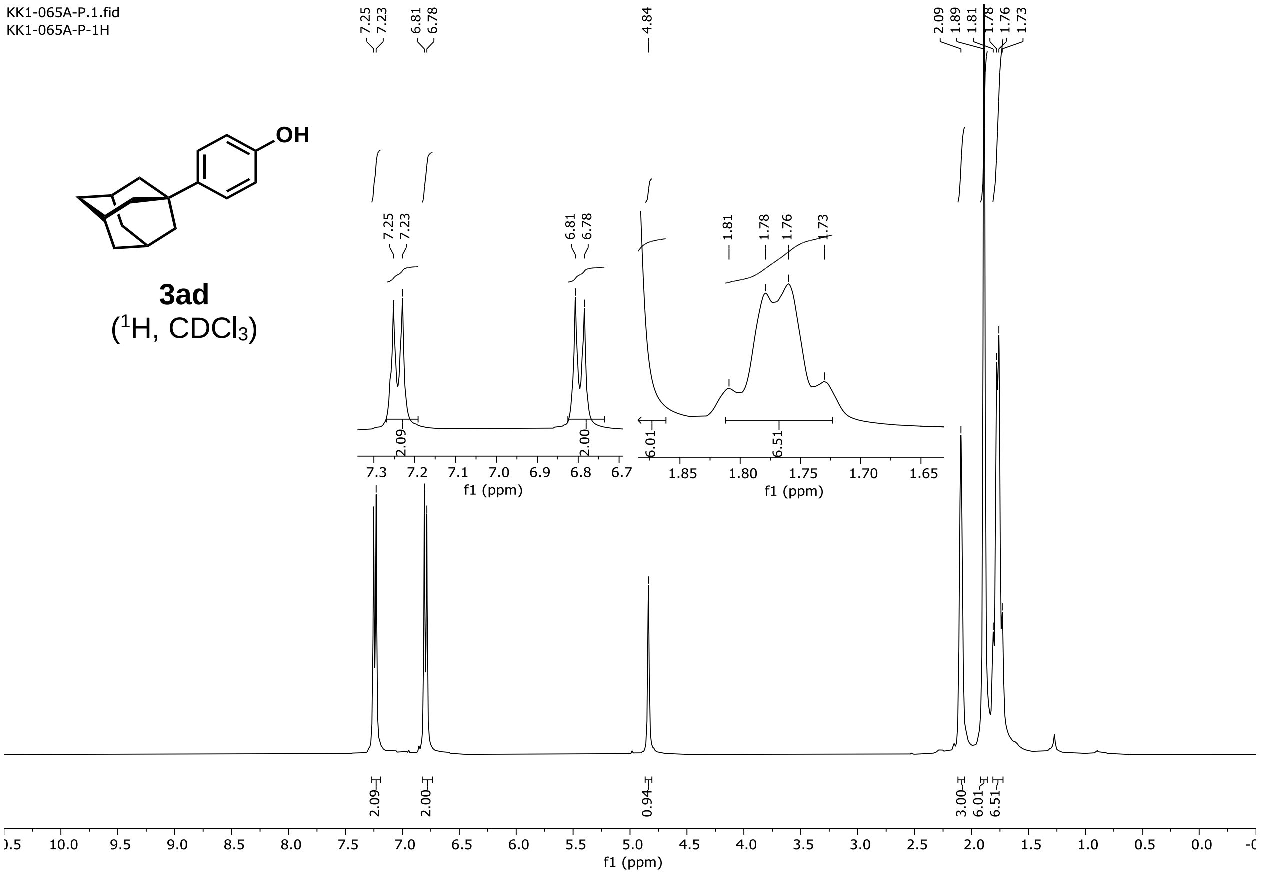
KK1-065A-P.1.fid
KK1-065A-P-1H
7.25
7.23
6.81
6.78
4.84
2.09
1.89
1.81
1.78
1.76
1.73
OH
3ad
(¹H, CDCl₃)
2.09
2.00
6.01
6.51
0.94
3.00
f1 (ppm)

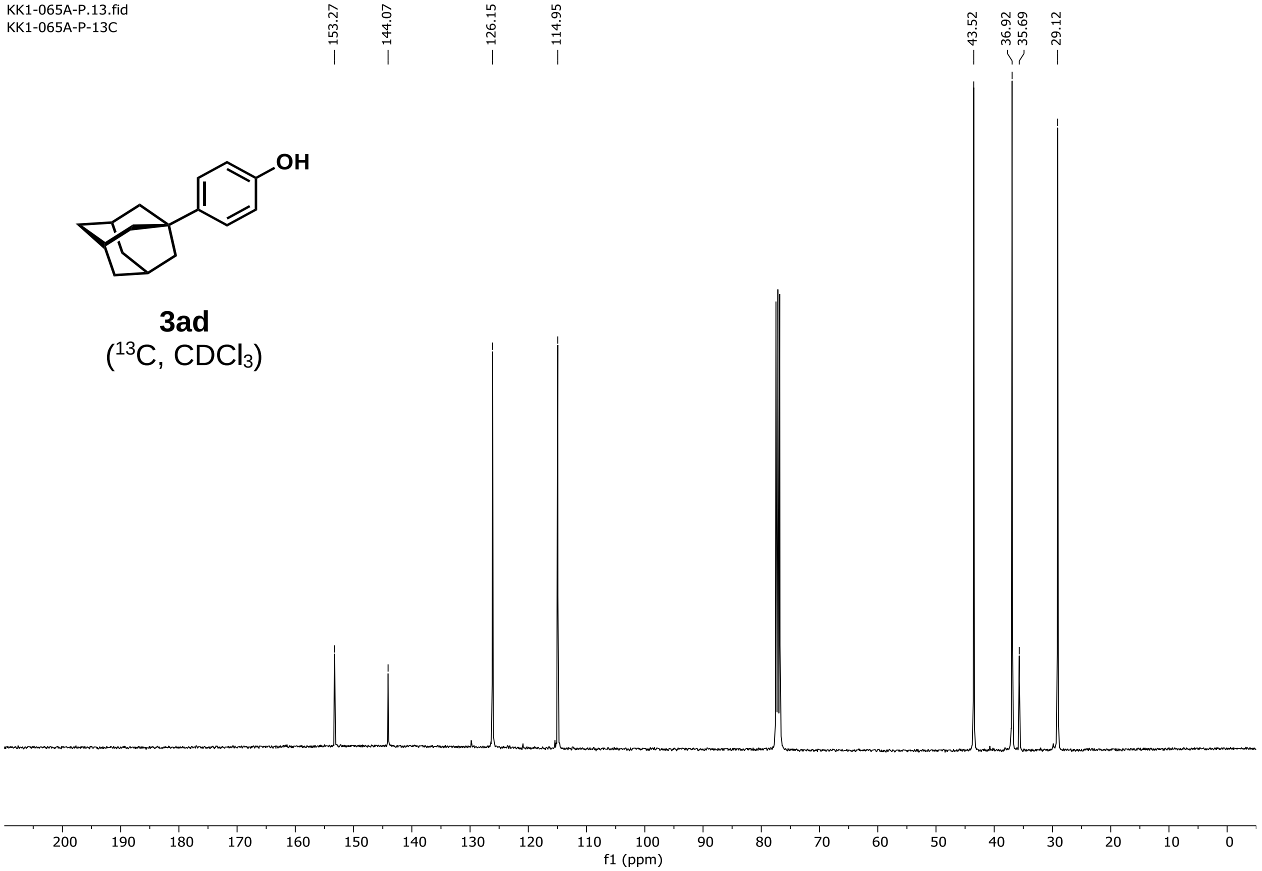
KK1-065A-P.13.fid
KK1-065A-P-13C
153.27
144.07
126.15
114.95
43.52
36.92
35.69
29.12
OH
3ad
(¹³C, CDCl₃)
f1 (ppm)

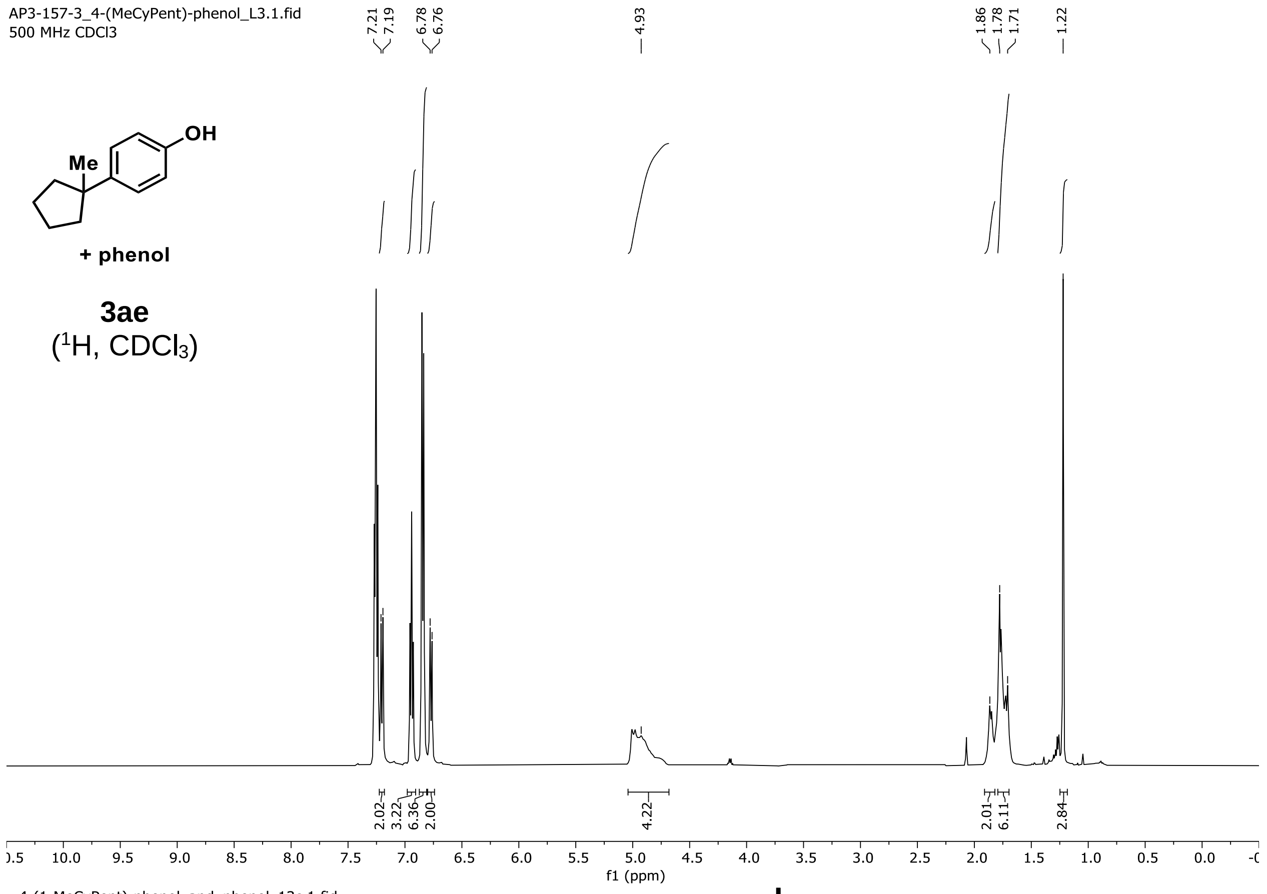

AP3-157-3_4-(MeCyPent)-phenol_L3.1.fid
500 MHz CDCl3
7.21
7.19
6.78
6.76
4.93
1.86
1.78
1.71
1.22
OH
Me
+ phenol
3ae
(¹H, CDCl₃)
2.02
3.22
6.36
2.00
4.22
2.01
6.11
2.84
f1 (ppm)

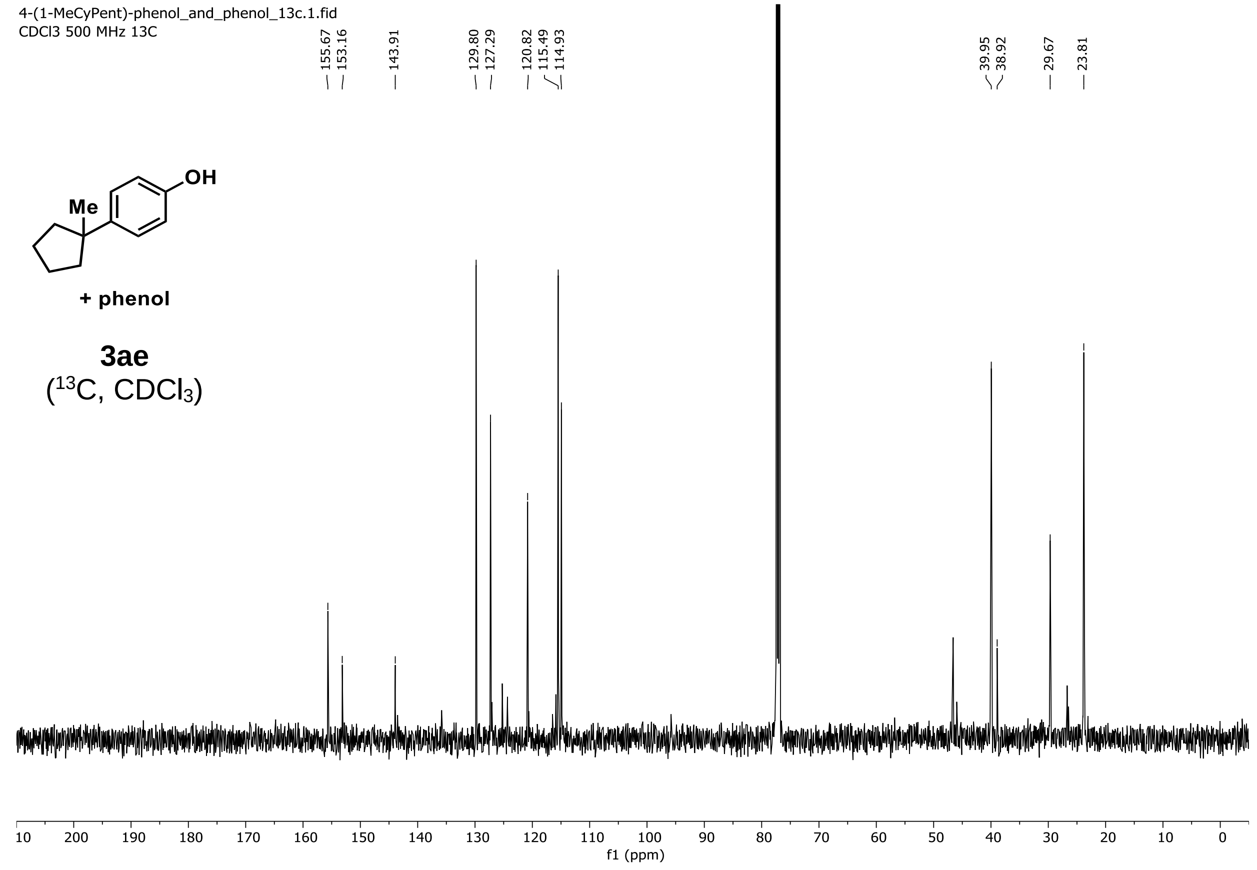

4-(1-MeCyPent)-phenol_and_phenol_13c.1.fid
CDCl3 500 MHz 13C
155.67
153.16
143.91
129.80
127.29
120.82
115.49
114.93
39.95
38.92
29.67
23.81
OH
Me
+ phenol
3ae
(¹³C, CDCl₃)
f1 (ppm)

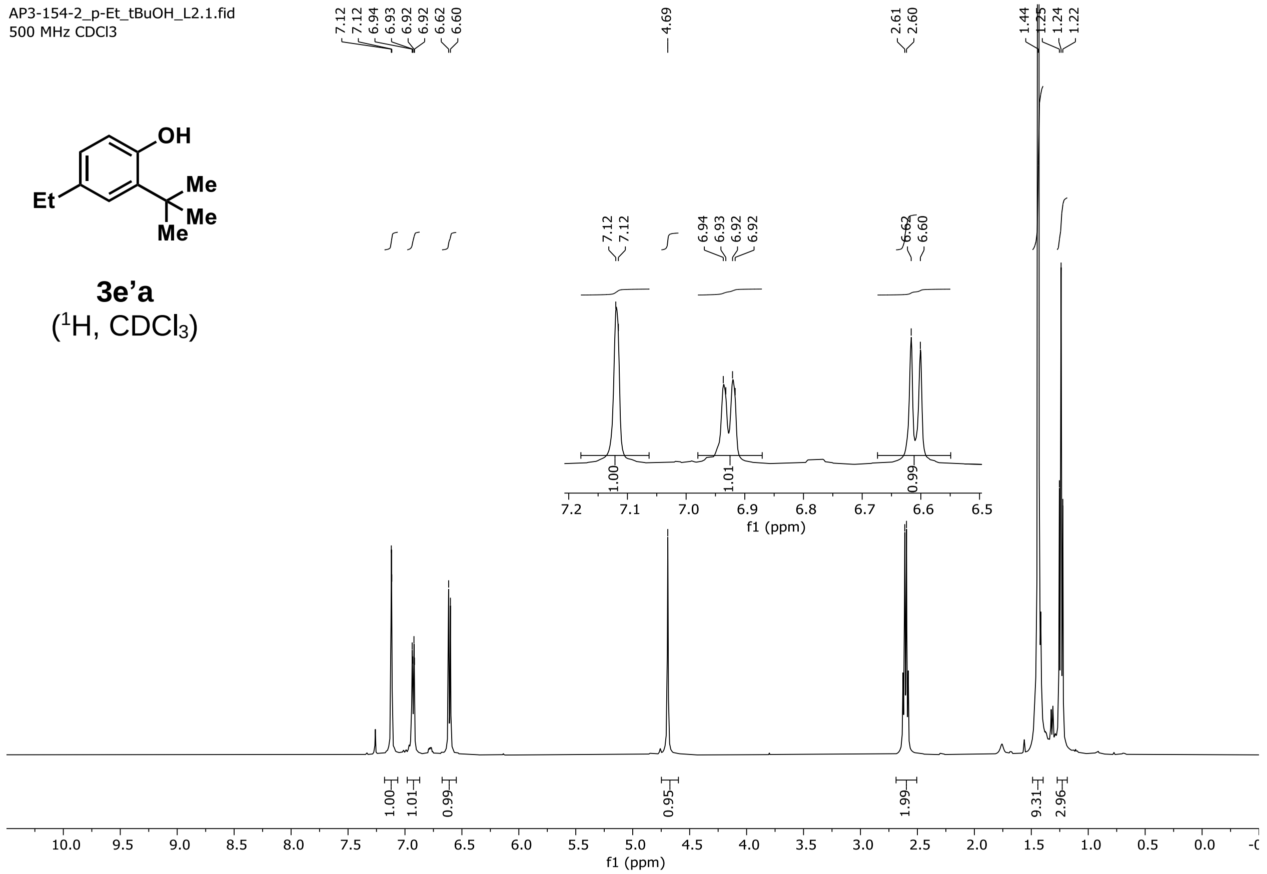

3e'a
(^{1}H, $CDCl_3$)

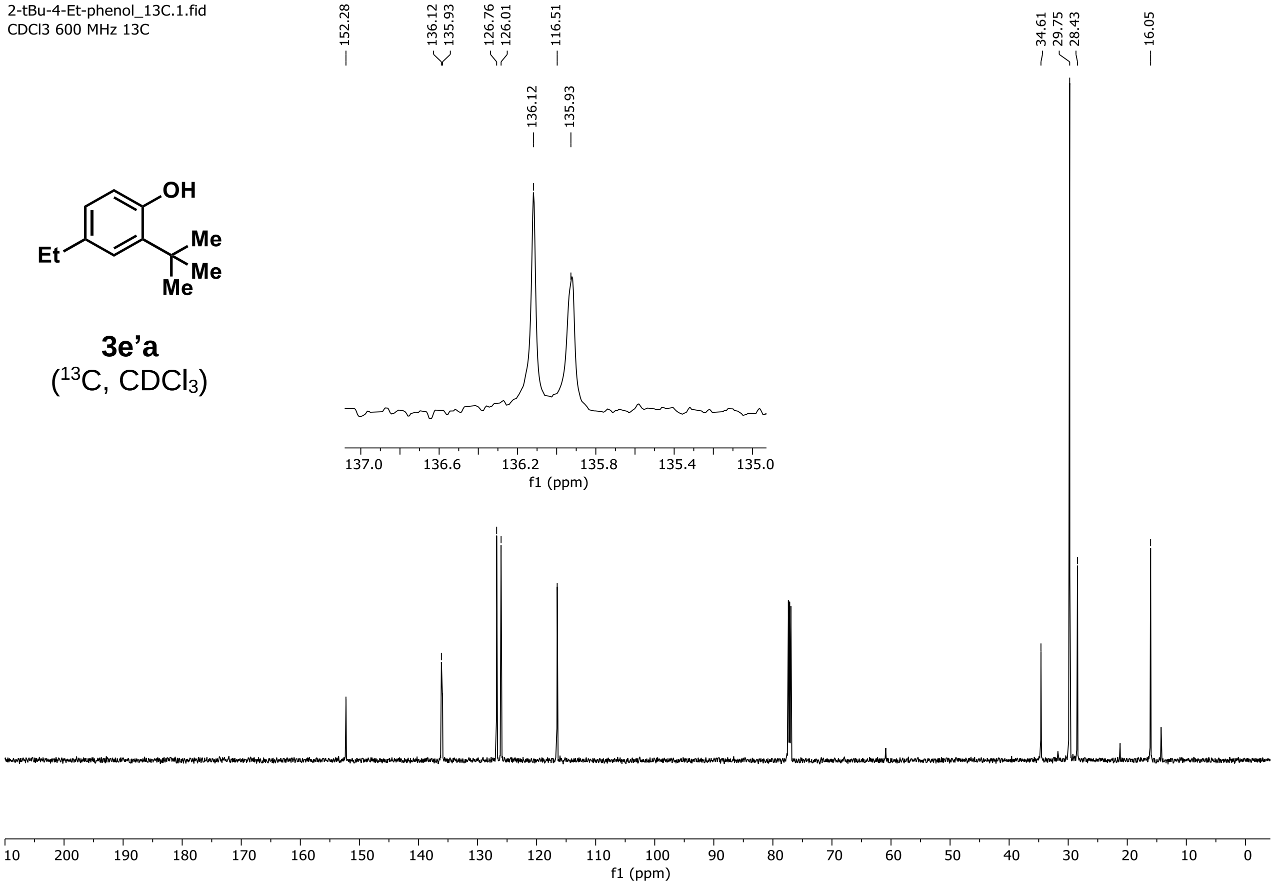

3e'a
(^{13}C, $CDCl_3$)

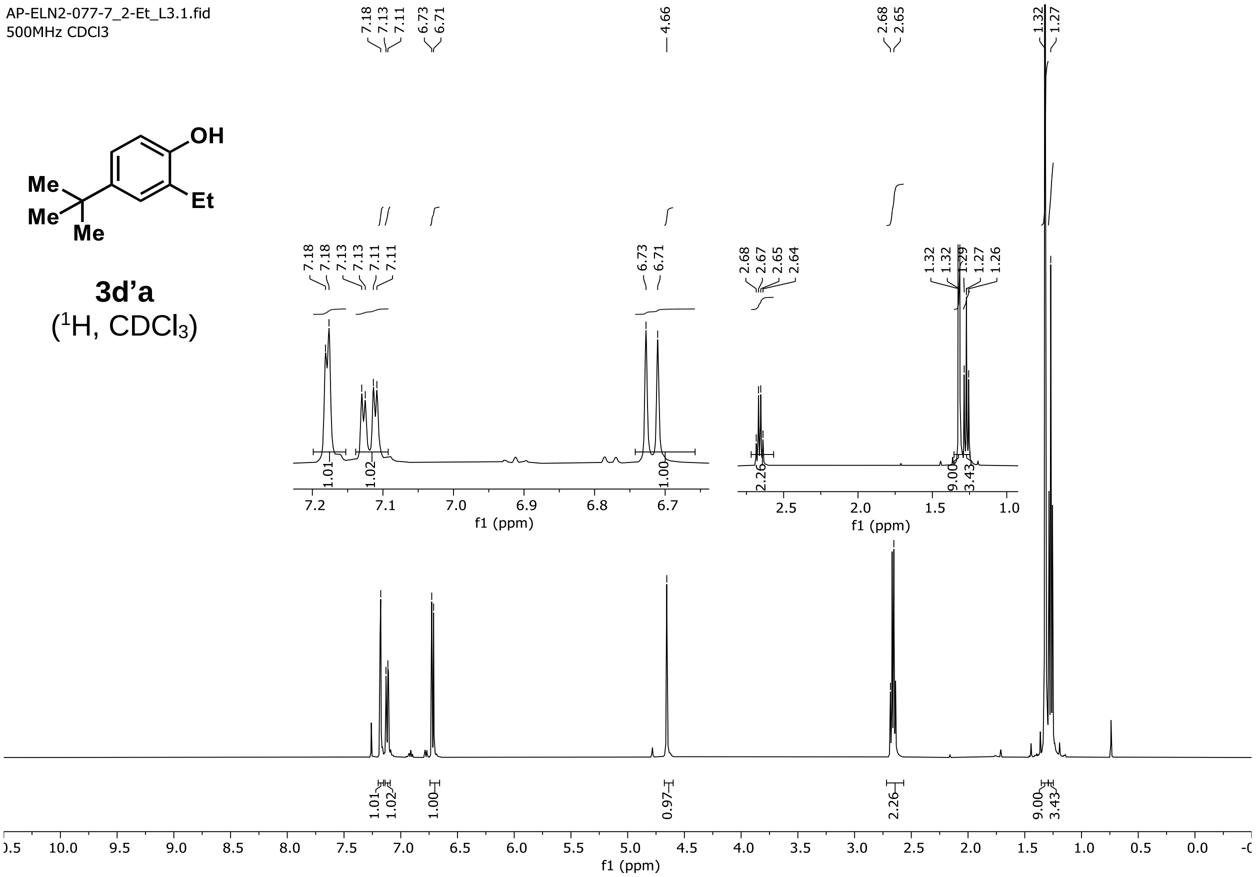
AP-ELN2-077-7_2-Et_L3.1.fid
500MHz CDCl3
OH
Et
Me
Me
Me
3d'a
(1H, CDCl3)
f1 (ppm)

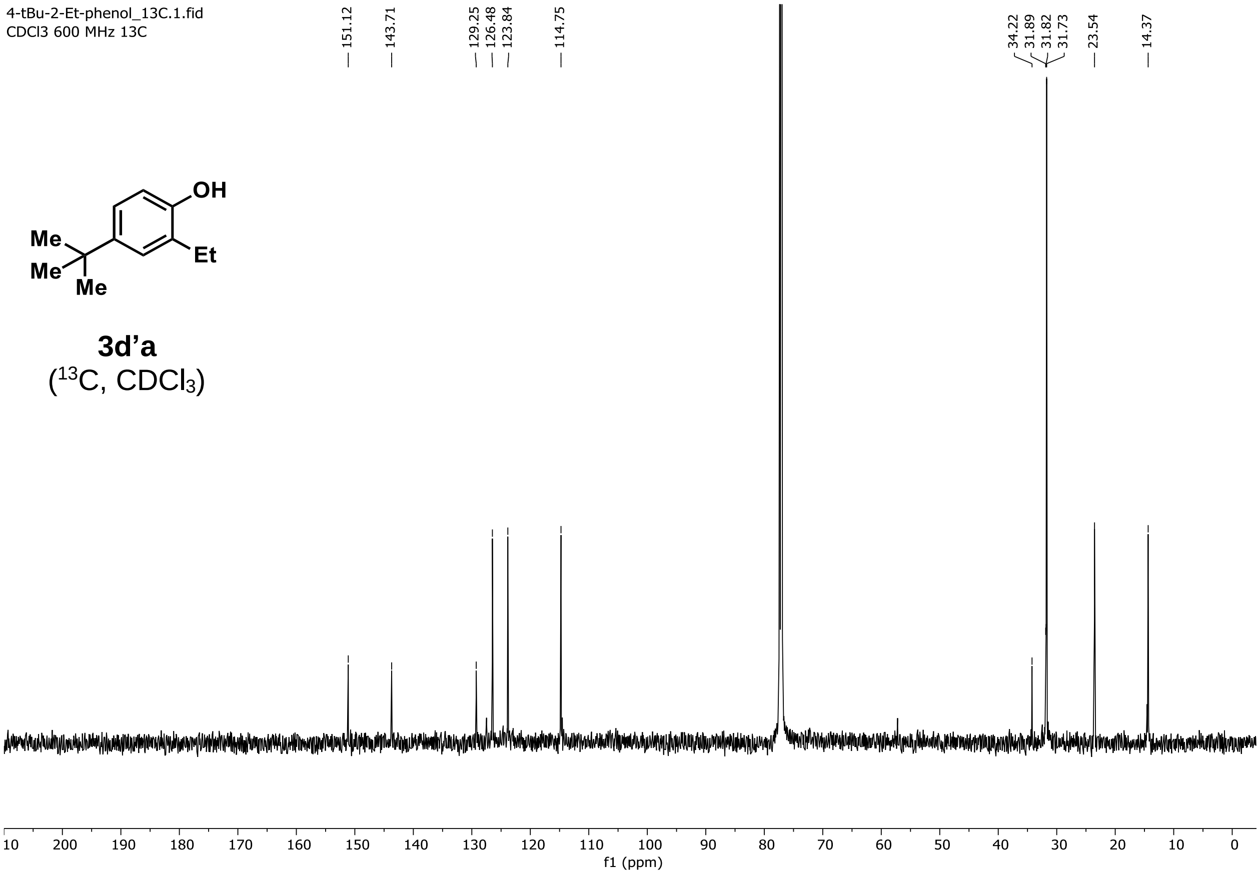
4-tBu-2-Et-phenol_13C.1.fid
CDCl3 600 MHz 13C
151.12
143.71
129.25
126.48
123.84
114.75
34.22
31.89
31.82
31.73
23.54
14.37
OH
Et
Me
Me
Me
3d'a
(13C, CDCl3)
f1 (ppm)

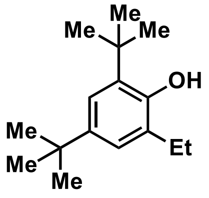

3d’aa
(^{1}H, $CDCl_3$)

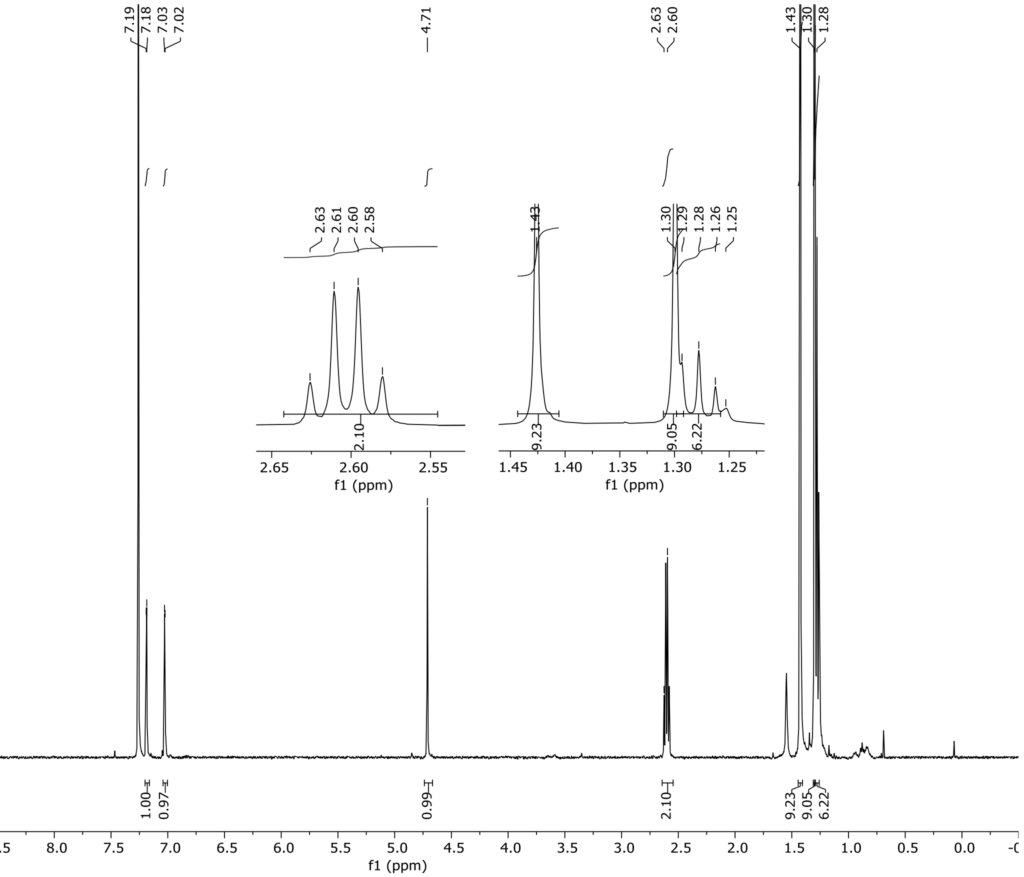

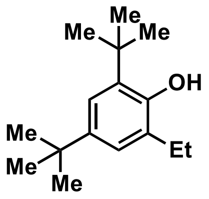

3d’aa
(^{13}C, $CDCl_3$)

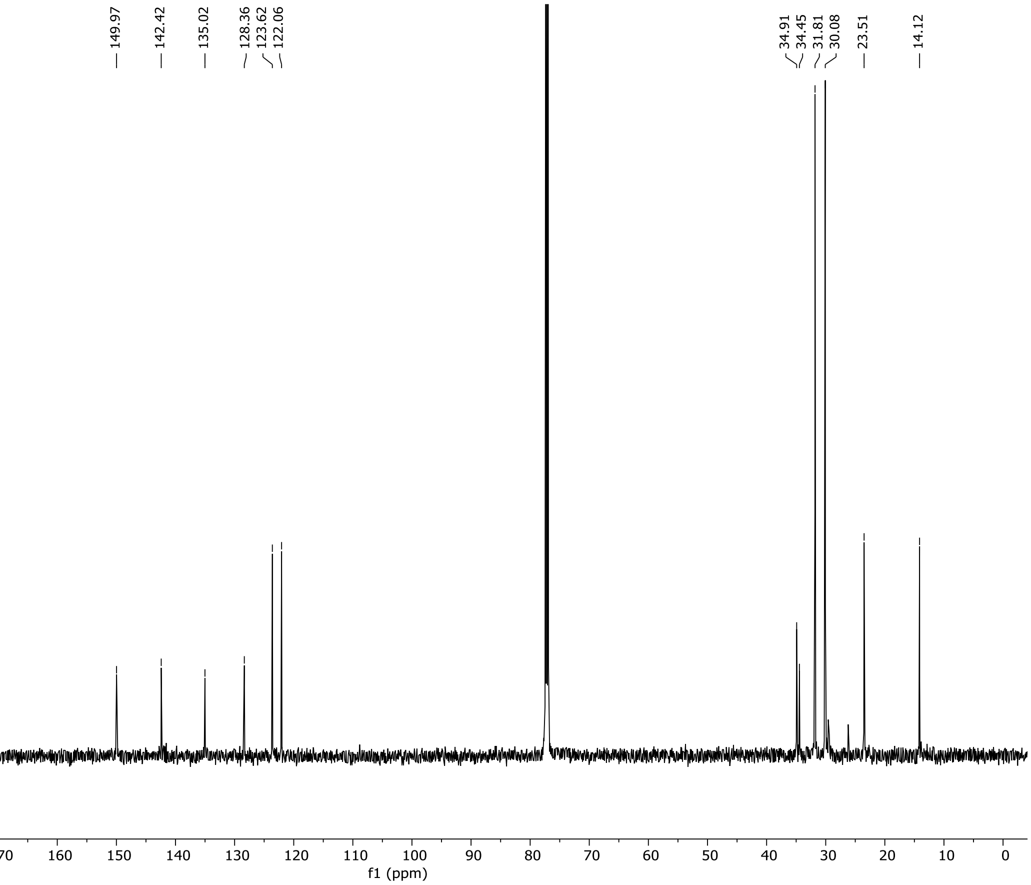

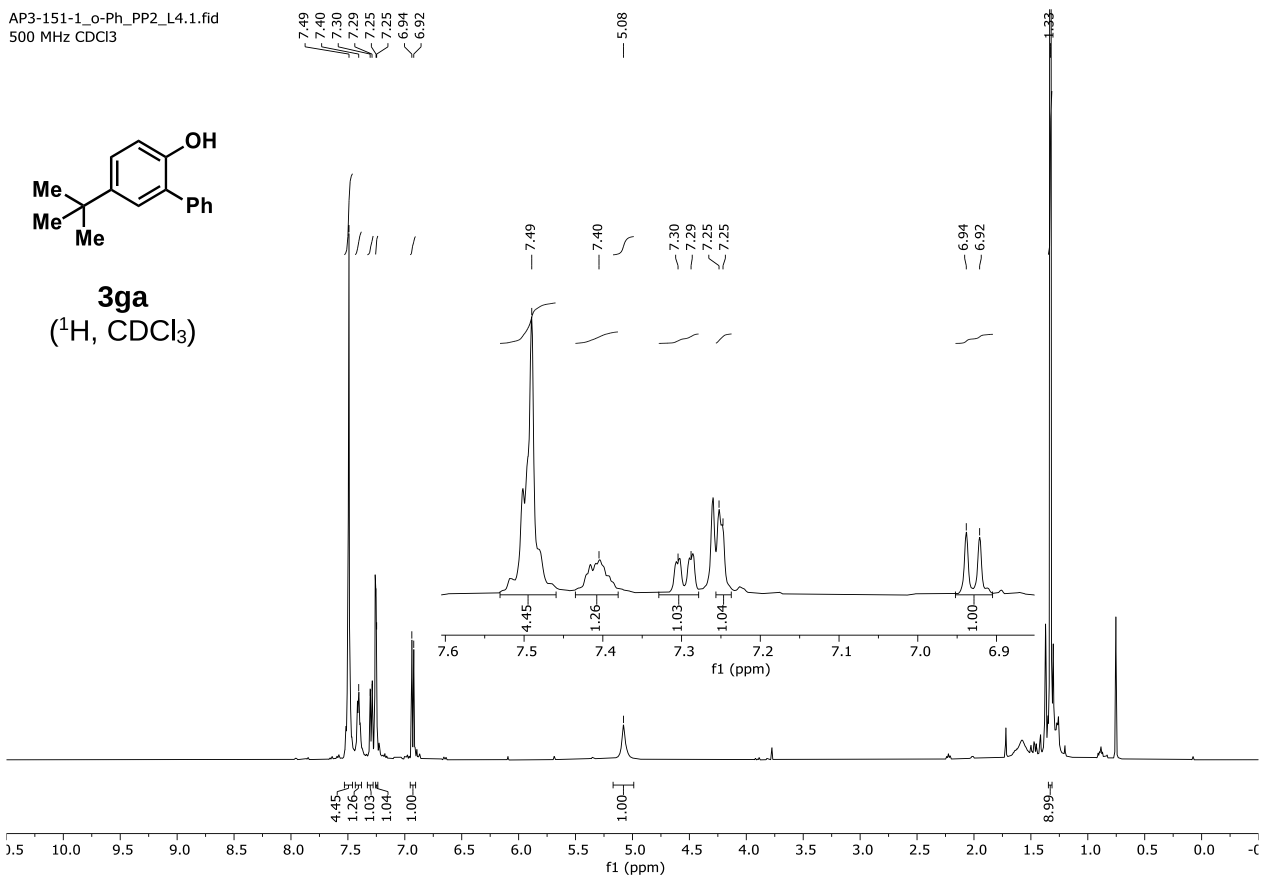

AP3-151-1_o-Ph_PP2_L4.1.fid
500 MHz CDCl3
OH
Me
Me
Me
Ph
3ga
(1H, CDCl3)
7.49
7.40
7.30
7.29
7.25
7.25
6.94
6.92
5.08
1.33
4.45
1.26
1.03
1.04
1.00
1.00
8.99
f1 (ppm)

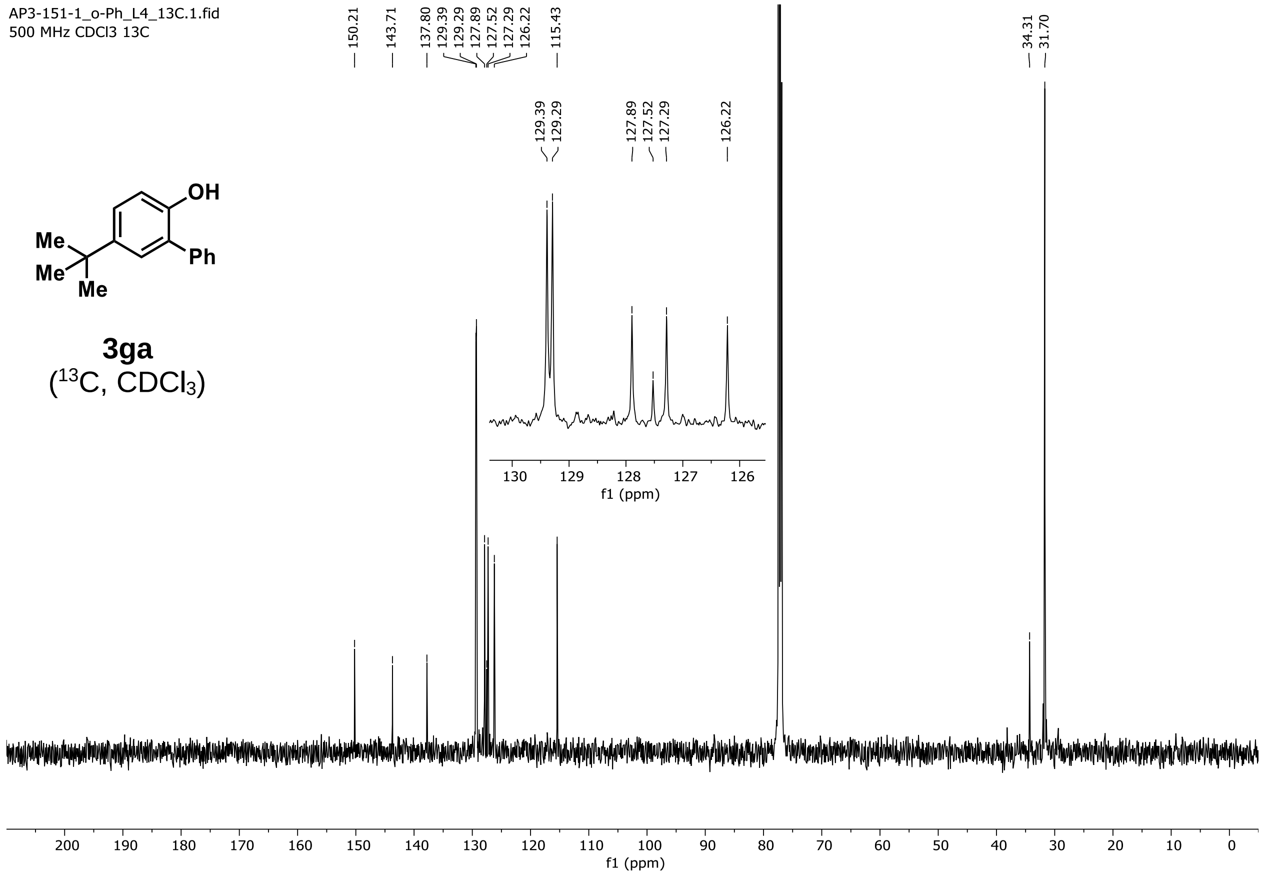

AP3-151-1_o-Ph_L4_13C.1.fid
500 MHz CDCl3 13C
150.21
143.71
137.80
129.39
129.29
127.89
127.52
127.29
126.22
115.43
34.31
31.70
OH
Me
Me
Me
Ph
3ga
(13C, CDCl3)
f1 (ppm)

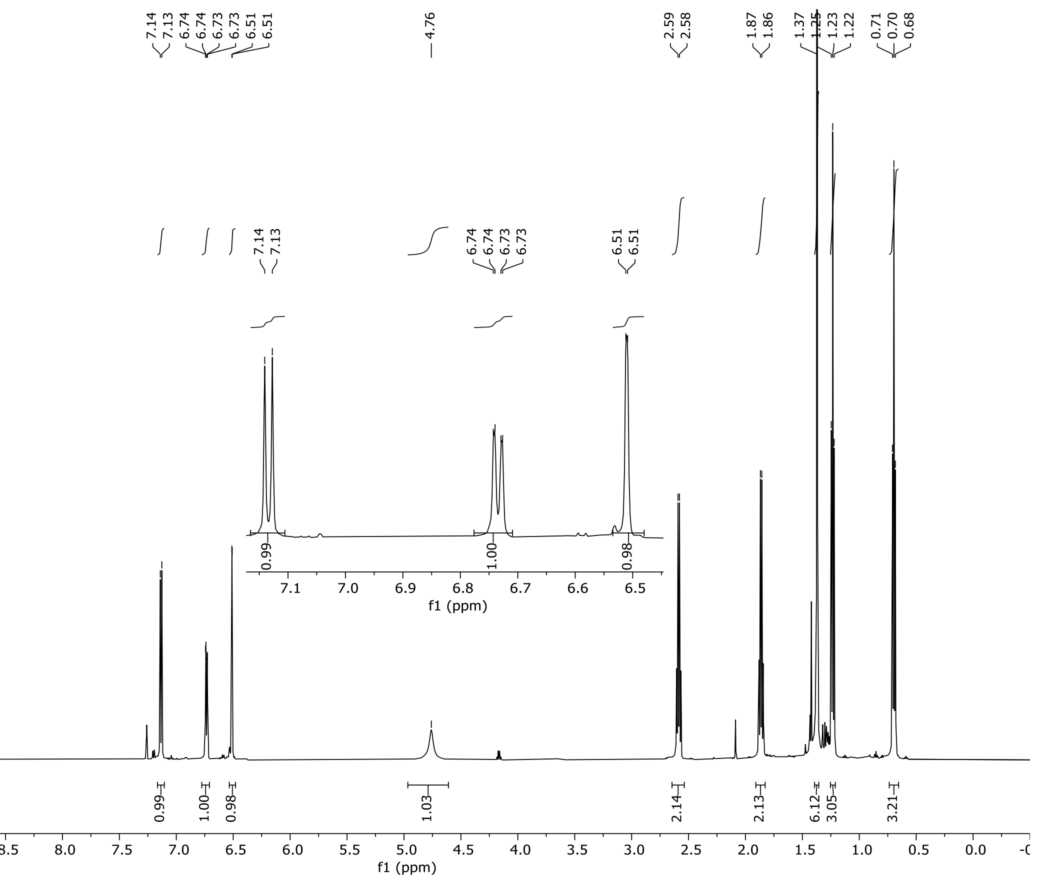

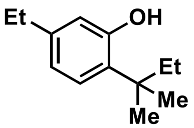

3eb
(^{1}H, $CDCl_3$)

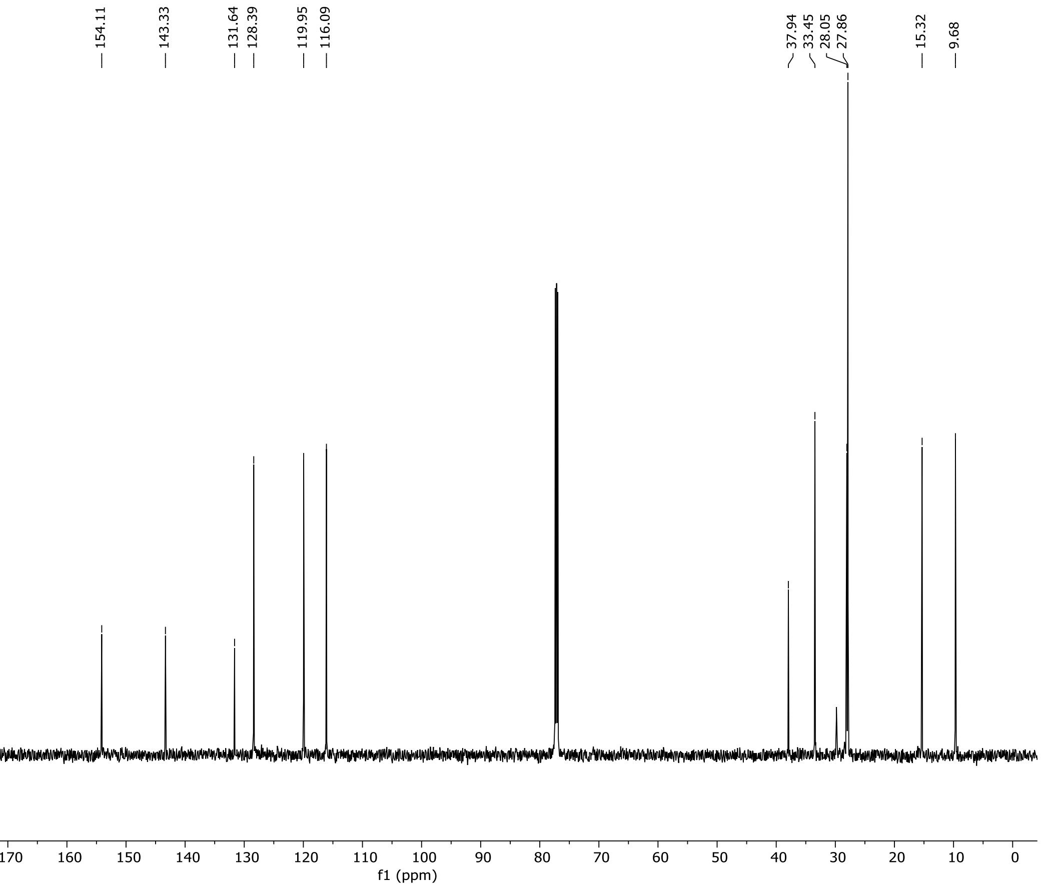

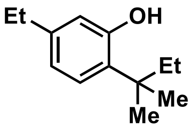

3eb
(^{13}C, $CDCl_3$)

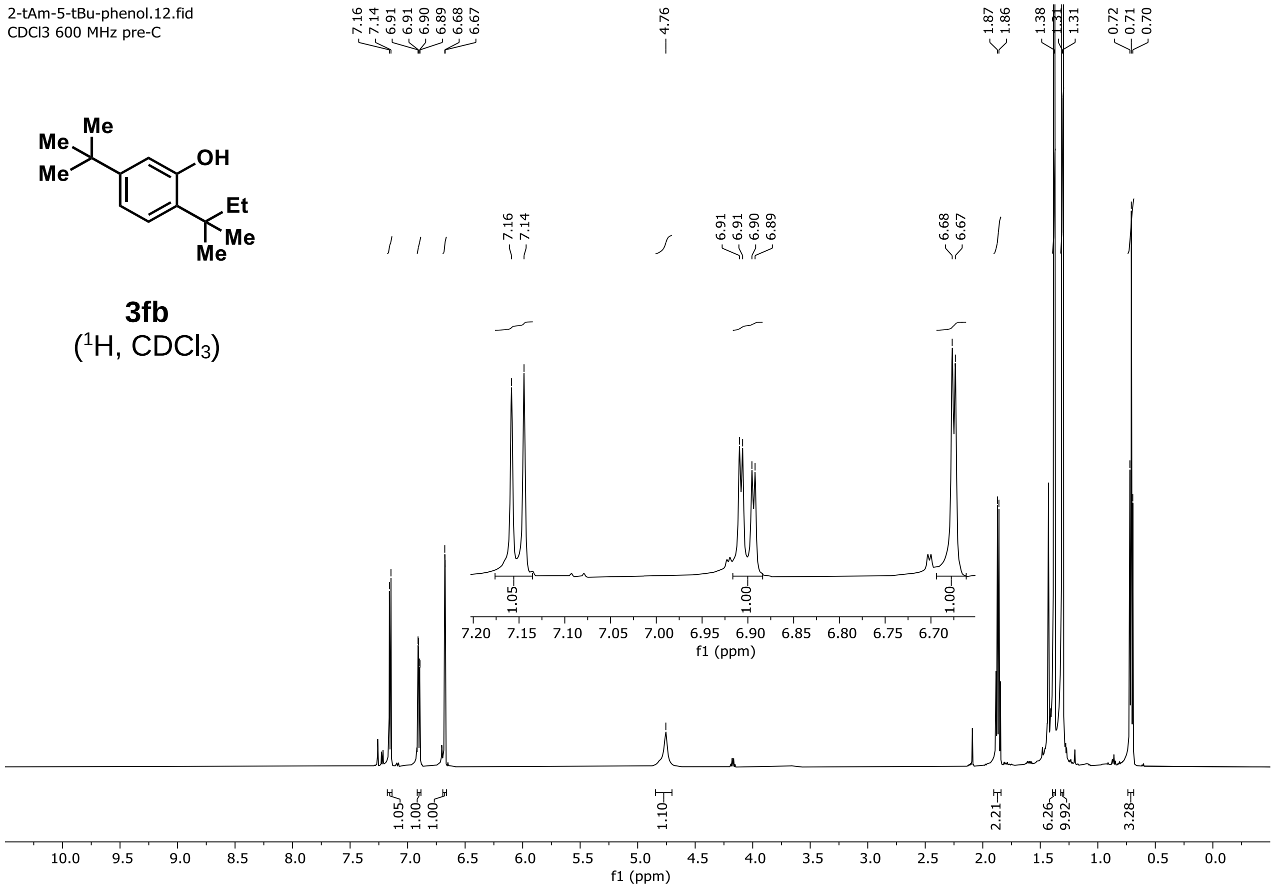

3fb
(^{1}H, $CDCl_3$)

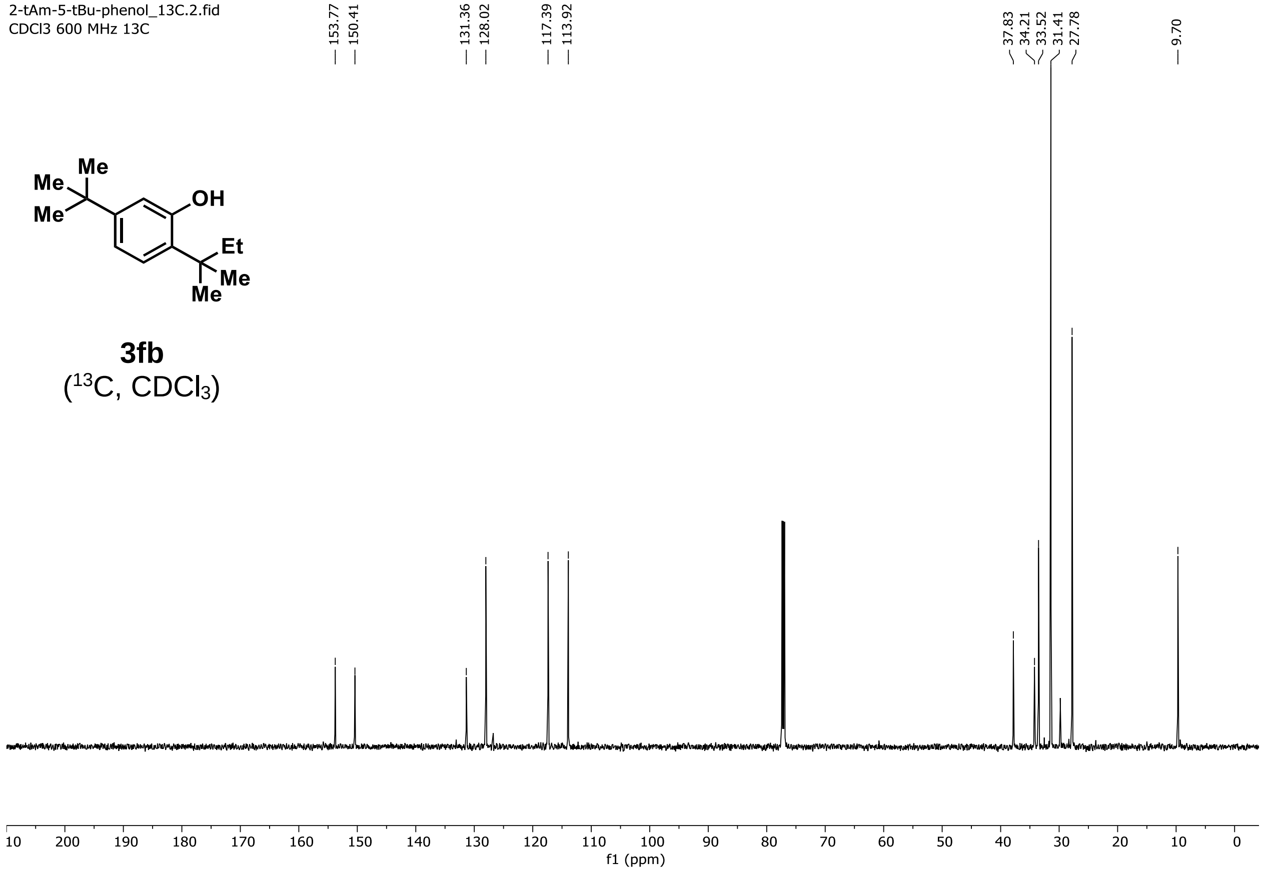

3fb
(^{13}C, $CDCl_3$)

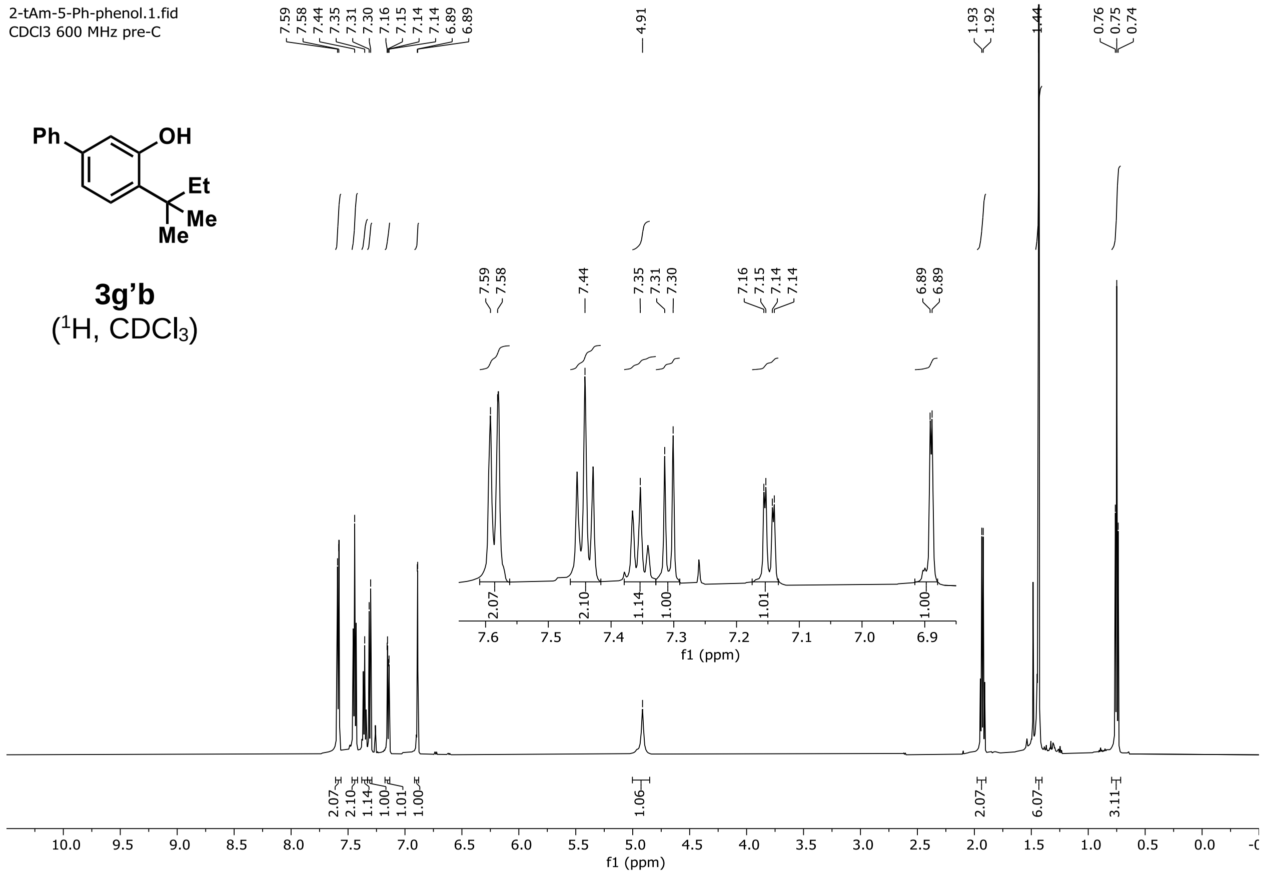
2-tAm-5-Ph-phenol.1.fid
CDCl3 600 MHz pre-C
7.59 7.58 7.44 7.35 7.31 7.30 7.16 7.15 7.14 7.14 6.89 6.89
4.91
1.93 1.92
1.44
0.76 0.75 0.74
Ph
OH
Et
Me
Me
3g'b
(1H, CDCl3)
2.07 2.10 1.14 1.00 1.01 1.00
1.06
2.07
6.07
3.11
f1 (ppm)

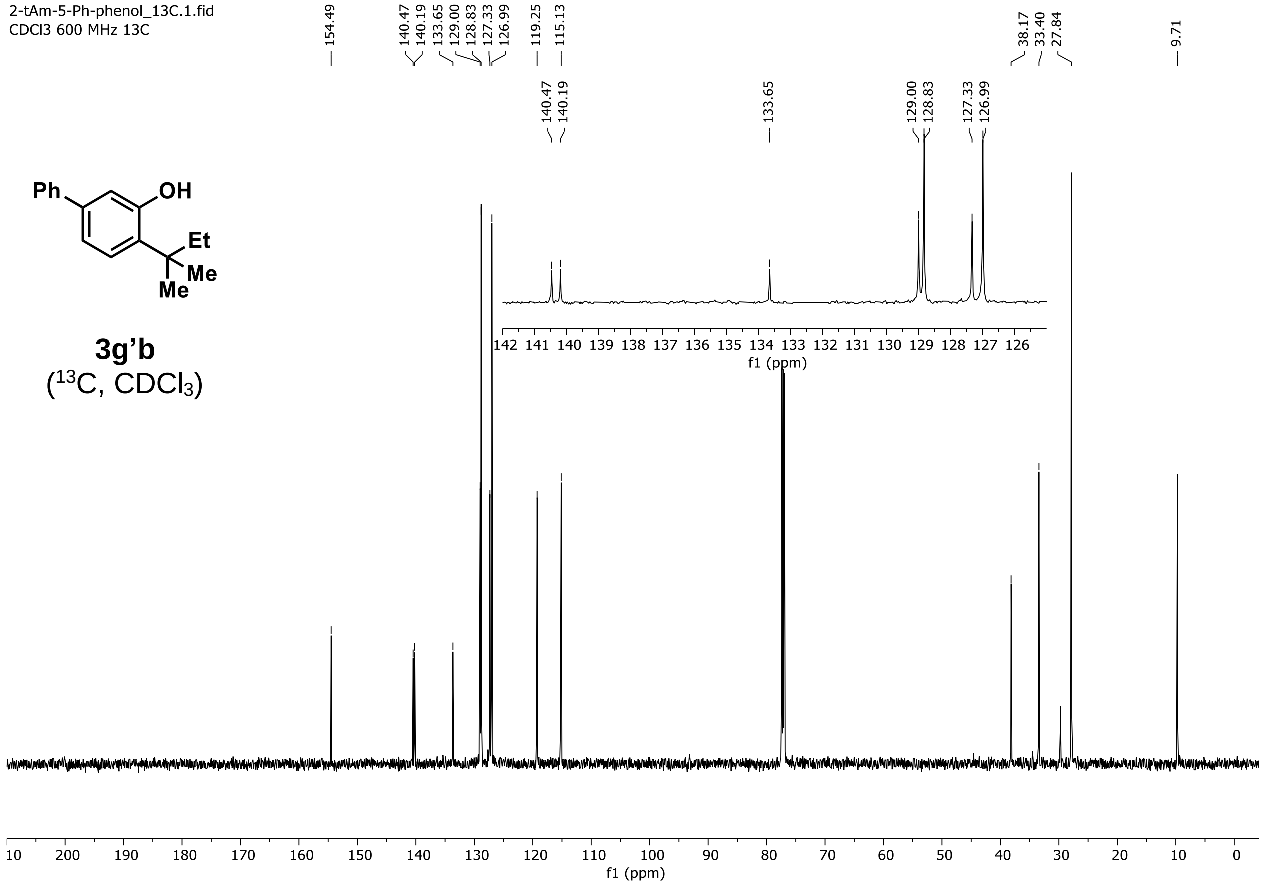
2-tAm-5-Ph-phenol_13C.1.fid
CDCl3 600 MHz 13C
154.49
140.47 140.19 133.65 129.00 128.83 127.33 126.99
119.25 115.13
38.17 33.40 27.84
9.71
Ph
OH
Et
Me
Me
3g'b
(13C, CDCl3)
f1 (ppm)

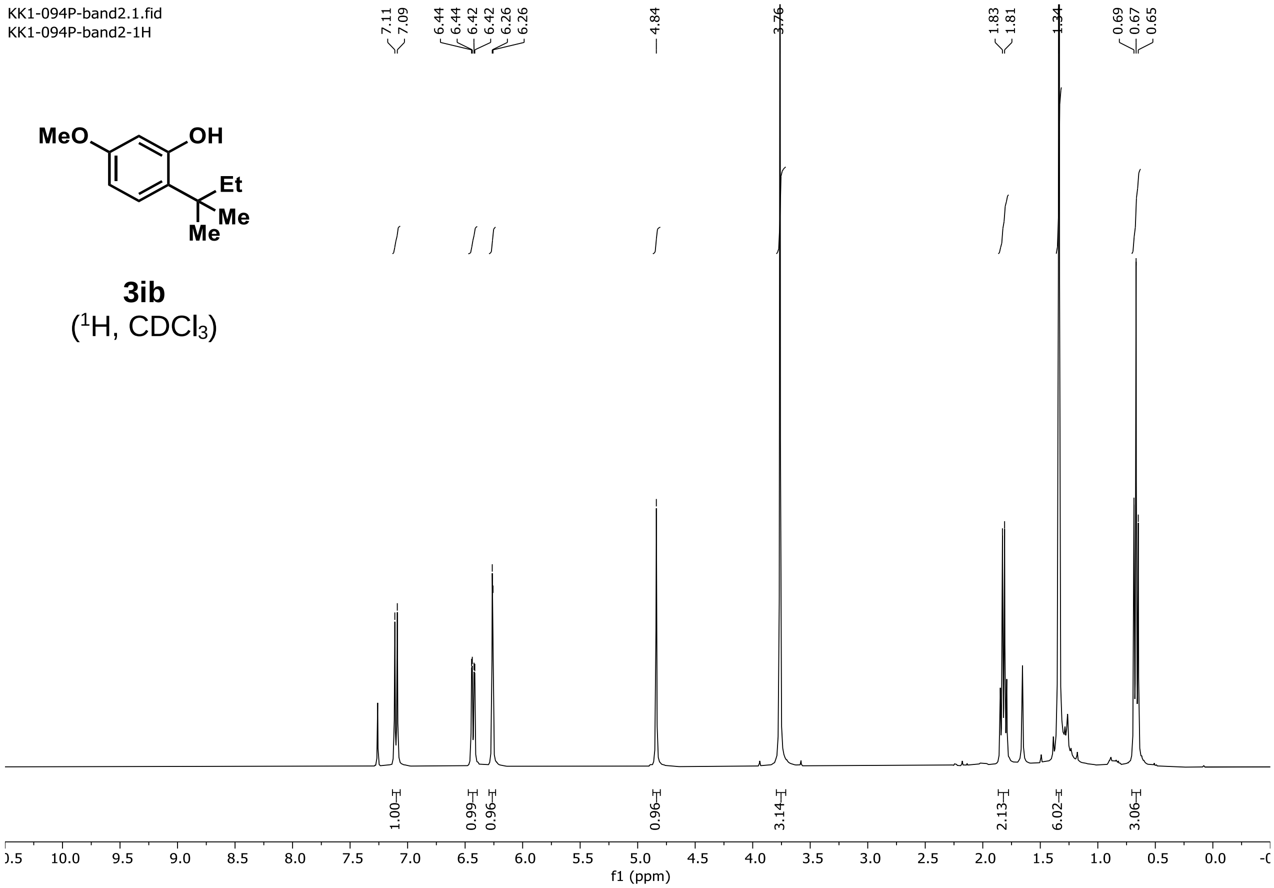
KK1-094P-band2.1.fid
KK1-094P-band2-1H
7.11
7.09
6.44
6.44
6.42
6.42
6.26
6.26
4.84
3.76
1.83
1.81
1.34
0.69
0.67
0.65
MeO
OH
Et
Me
Me
3ib
(1H, CDCl3)
1.00
0.99
0.96
0.96
3.14
2.13
6.02
3.06
f1 (ppm)

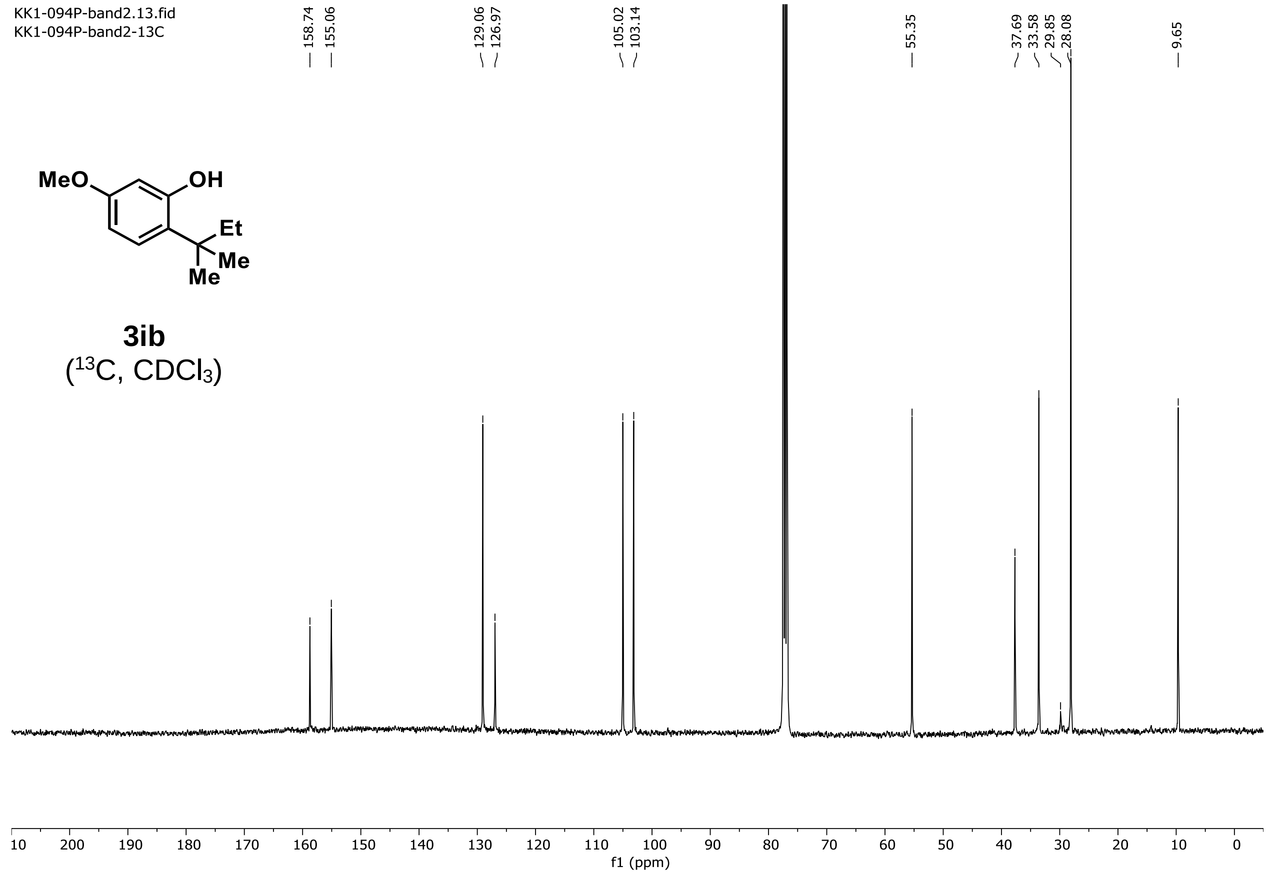
KK1-094P-band2.13.fid
KK1-094P-band2-13C
158.74
155.06
129.06
126.97
105.02
103.14
55.35
37.69
33.58
29.85
28.08
9.65
MeO
OH
Et
Me
Me
3ib
(13C, CDCl3)
f1 (ppm)

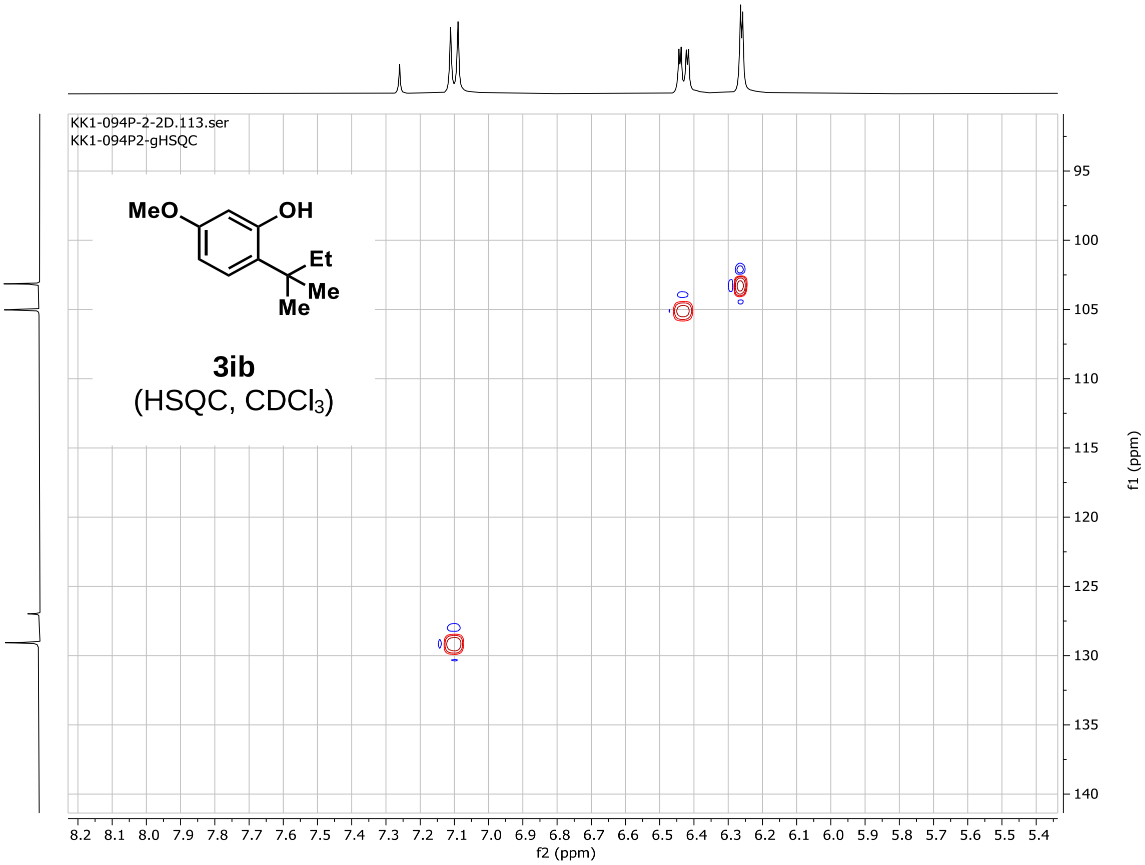
KK1-094P-2-2D.113.ser
KK1-094P2-gHSQC
MeO
OH
Et
Me
Me
3ib
(HSQC, CDCl3)
f1 (ppm)
f2 (ppm)

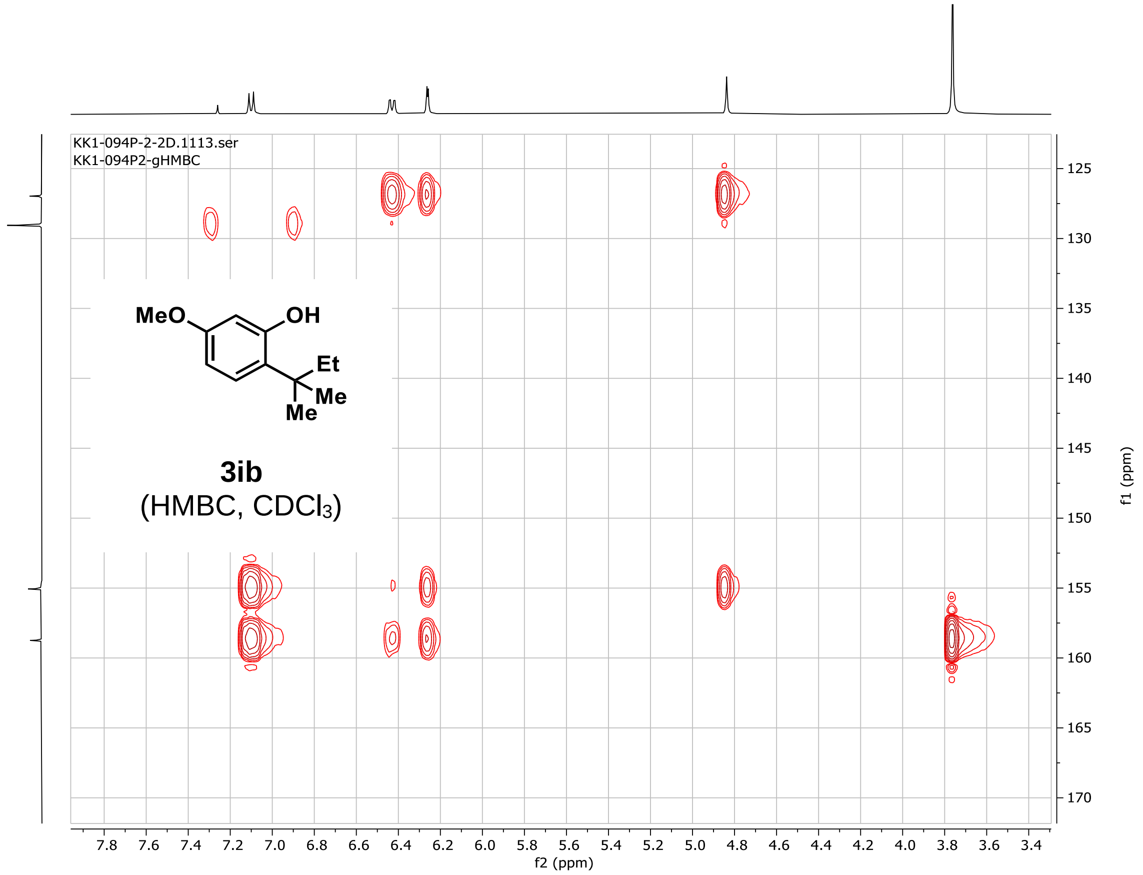
KK1-094P-2-2D.1113.ser
KK1-094P2-gHMBC
MeO
OH
Et
Me
Me
3ib
(HMBC, CDCl3)
f1 (ppm)
f2 (ppm)

3mb
(^{1}H, $CDCl_3$)

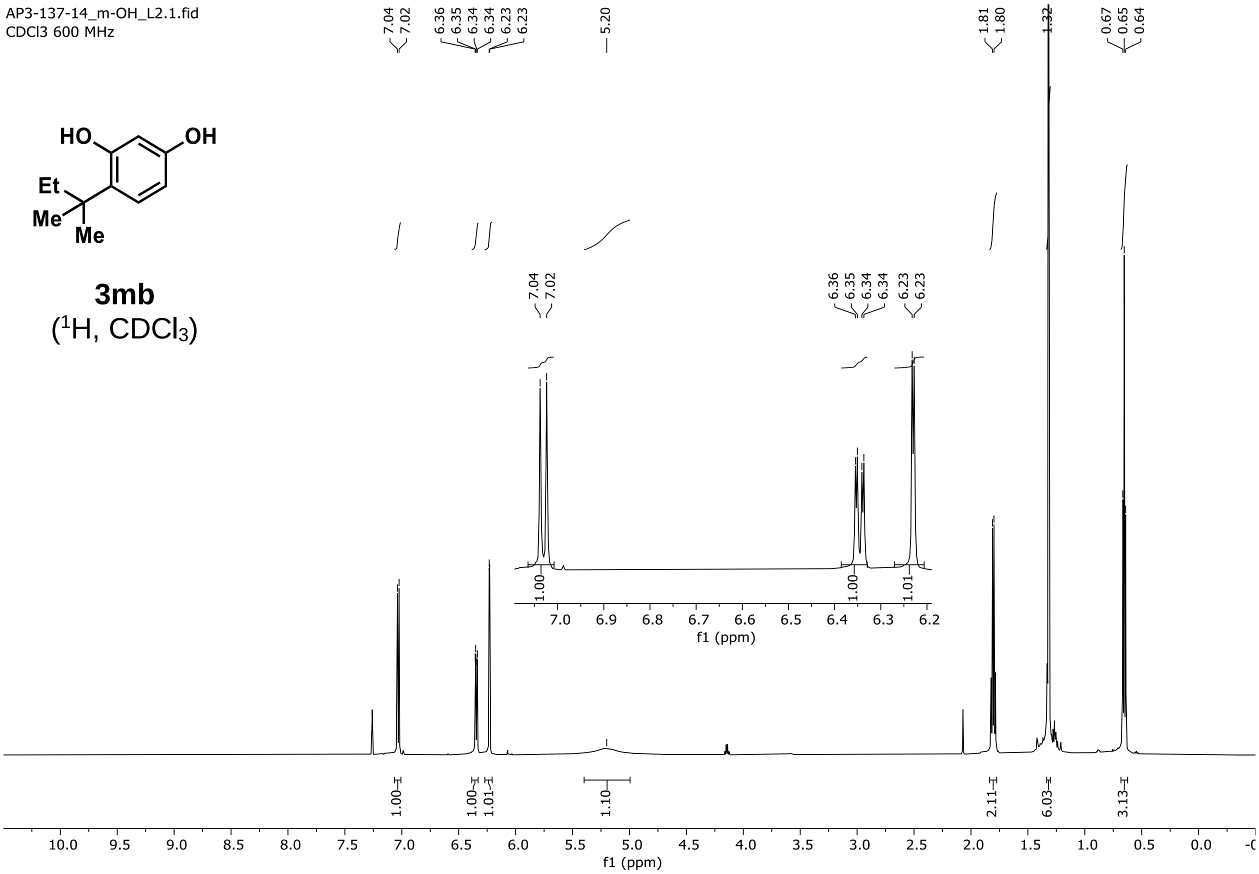

3mb
(^{13}C, $CDCl_3$)

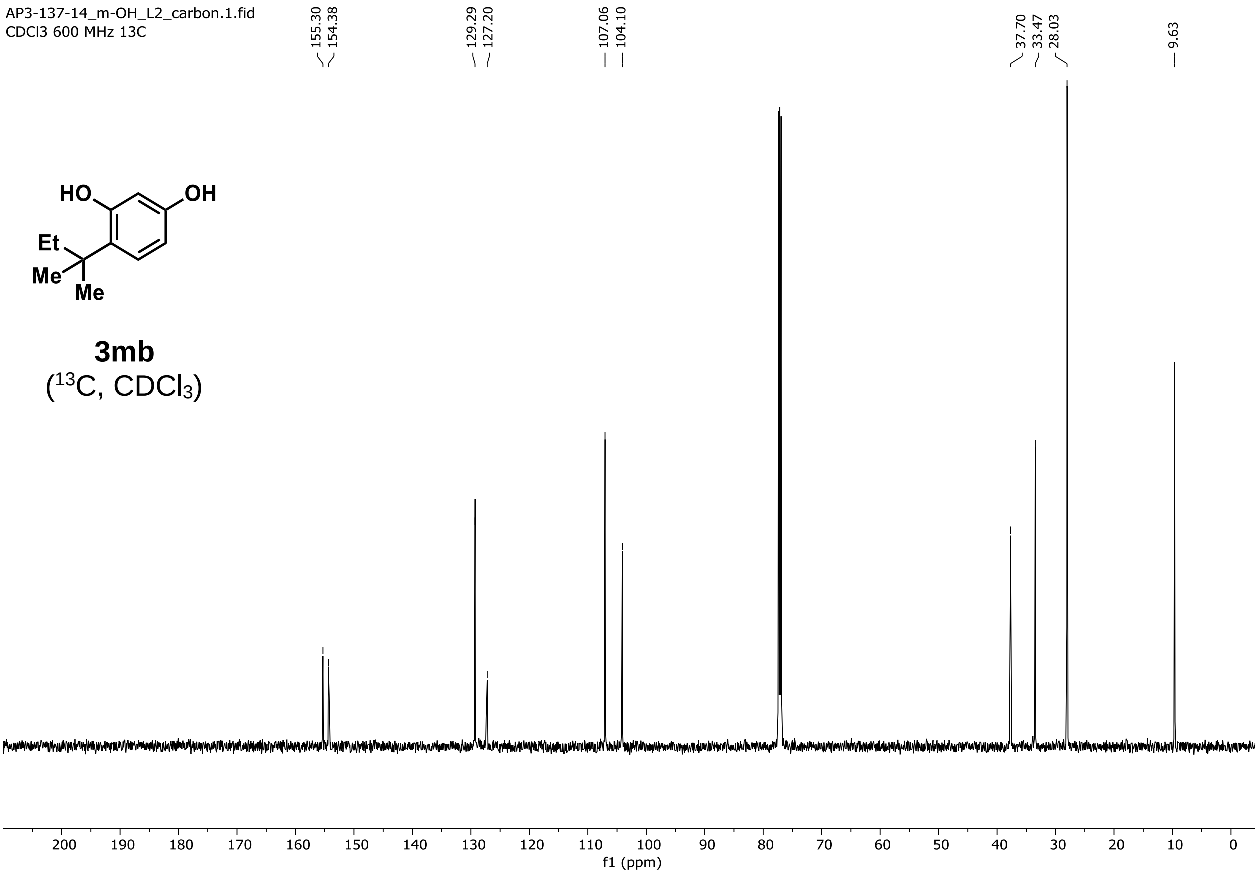

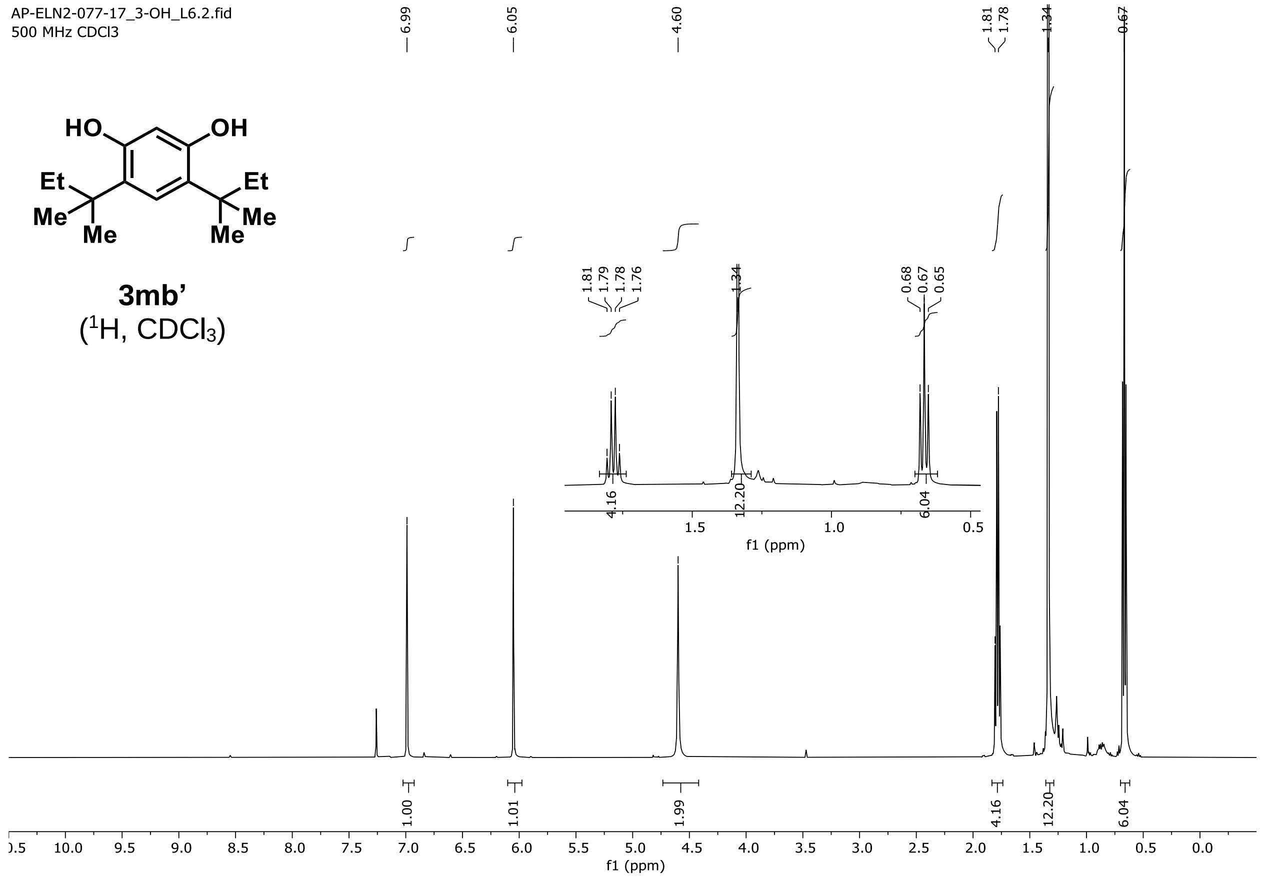

3mb'
(^{1}H, $CDCl_3$)

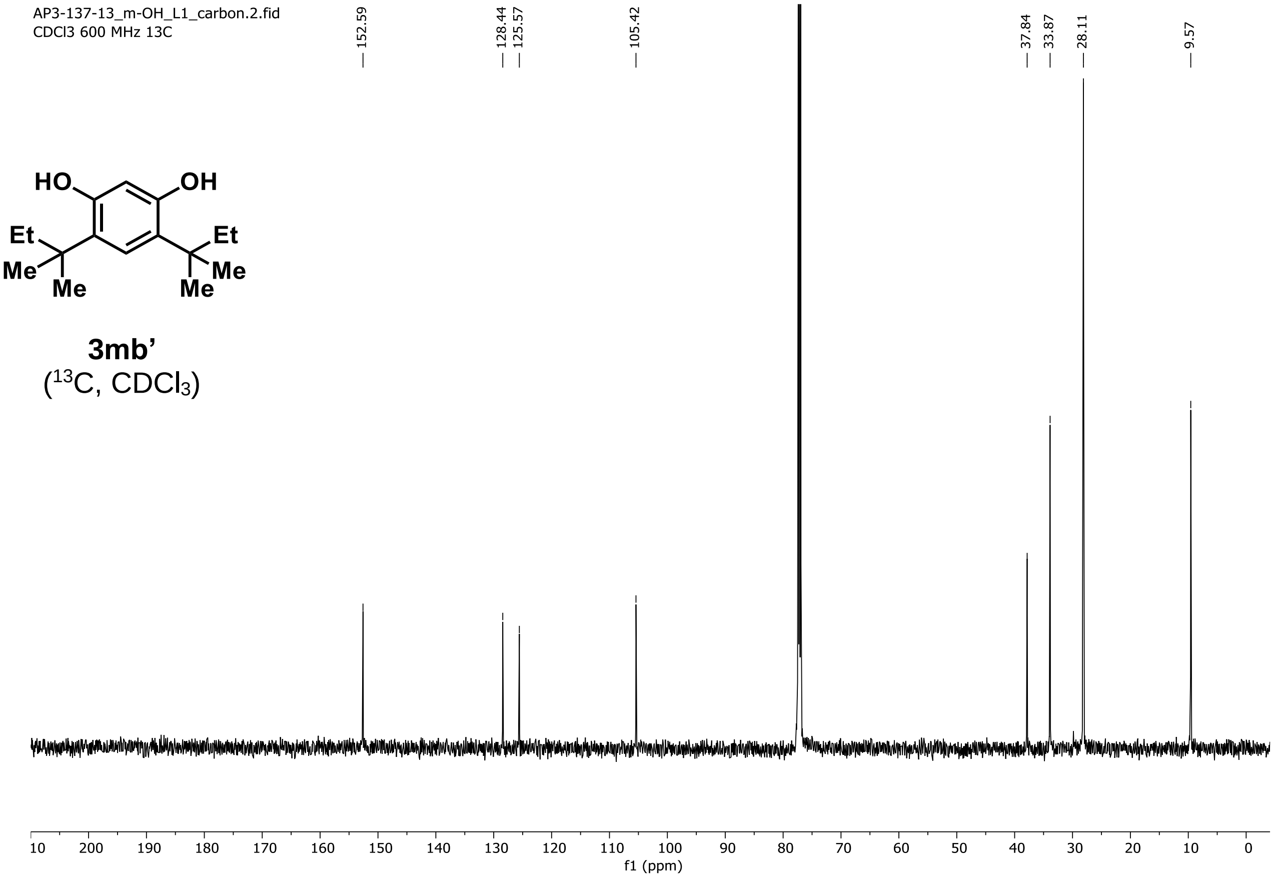

3mb'
(^{13}C, $CDCl_3$)

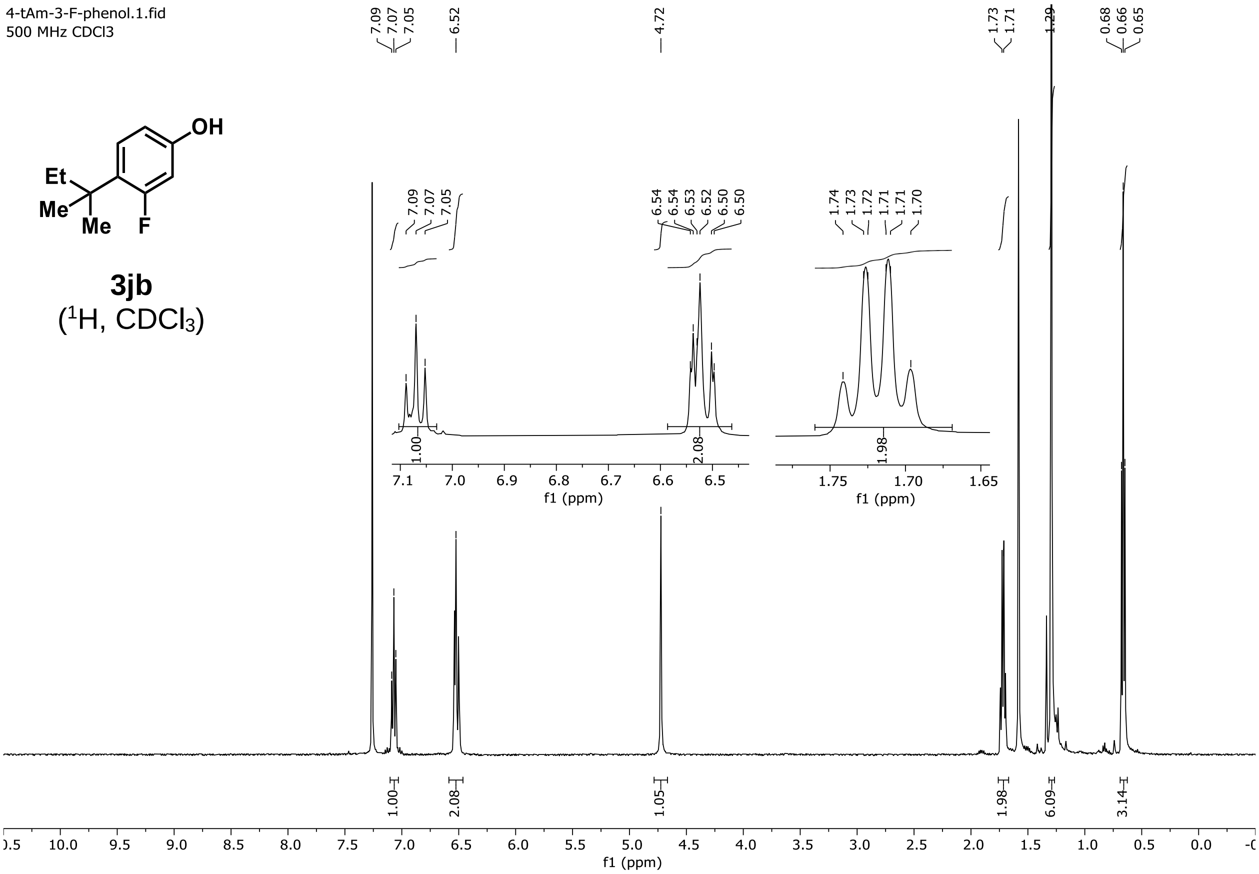
4-tAm-3-F-phenol.1.fid
500 MHz CDCl3
7.09
7.07
7.05
6.52
4.72
1.73
1.71
1.29
0.68
0.66
0.65
OH
Et
Me
Me
F
3jb
(^{1}H, $CDCl_3$)
7.09
7.07
7.05
6.54
6.54
6.53
6.52
6.50
6.50
1.74
1.73
1.72
1.71
1.71
1.70
1.00
2.08
1.98
f1 (ppm)
f1 (ppm)
1.00
2.08
1.05
1.98
6.09
3.14
f1 (ppm)

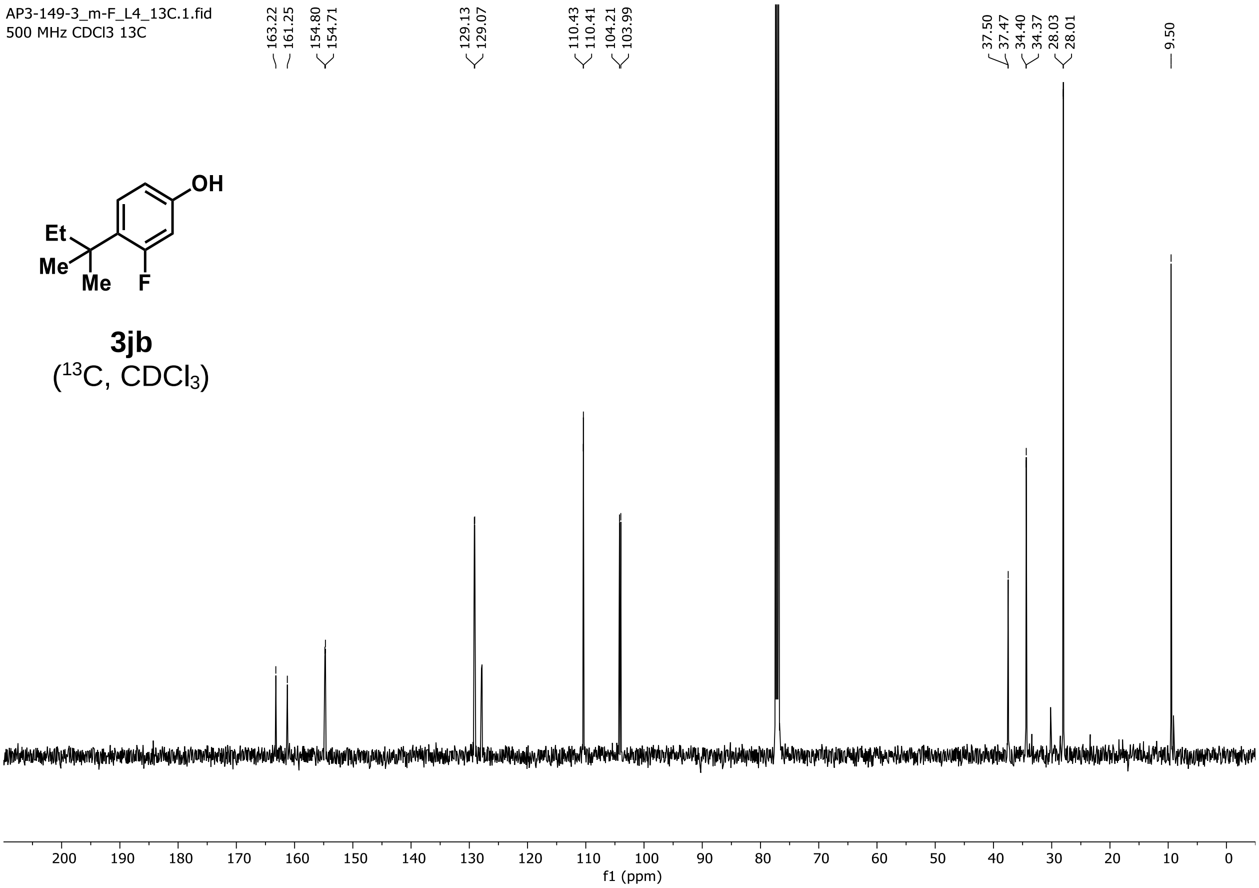
AP3-149-3_m-F_L4_13C.1.fid
500 MHz CDCl3 13C
163.22
161.25
154.80
154.71
129.13
129.07
110.43
110.41
104.21
103.99
37.50
37.47
34.40
34.37
28.03
28.01
9.50
OH
Et
Me
Me
F
3jb
(^{13}C, $CDCl_3$)
f1 (ppm)

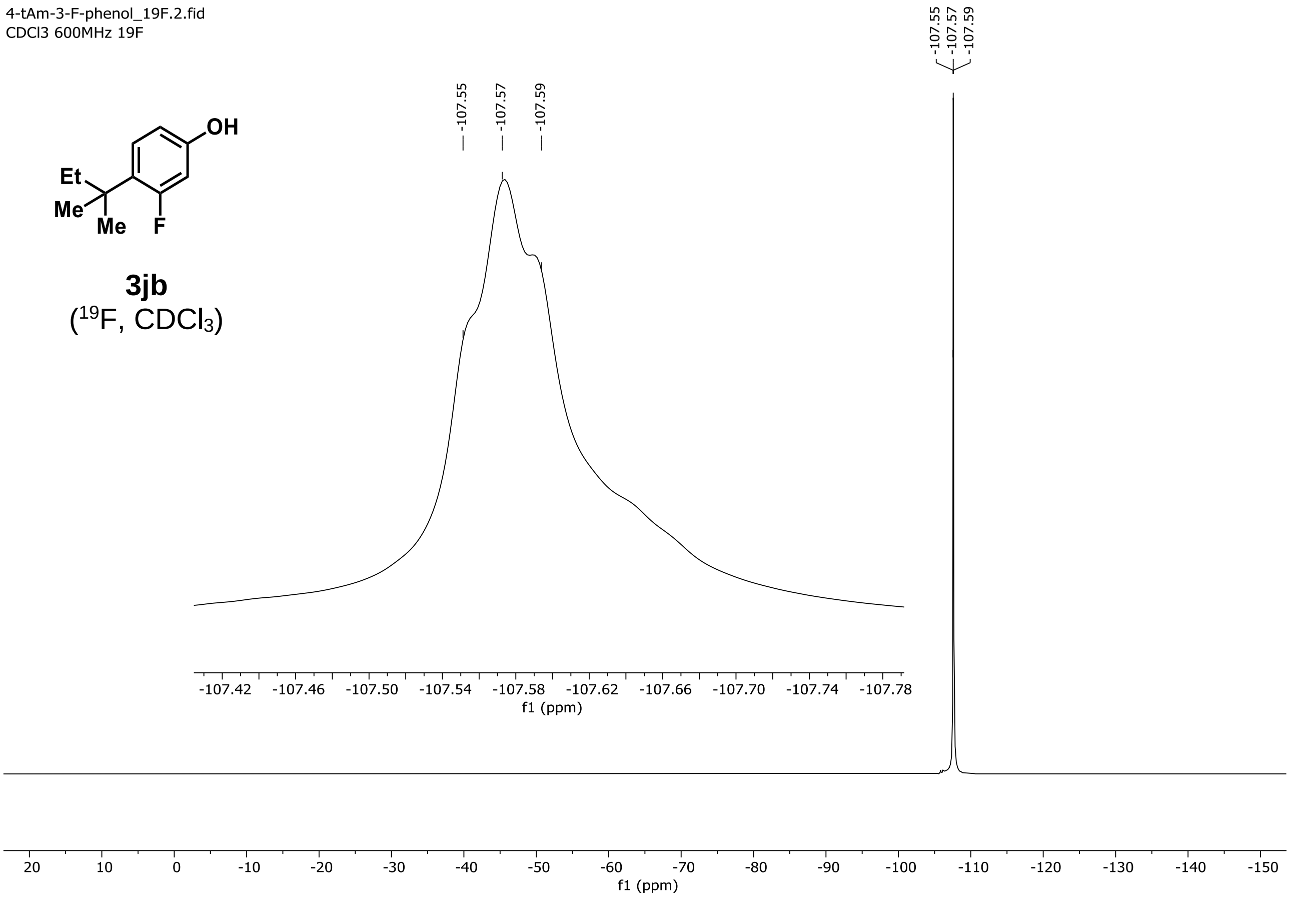

4-tAm-3-F-phenol_19F.2.fid
CDCl3 600MHz 19F
OH
Et
Me
Me
F
3jb
(19F, CDCl3)
-107.55
-107.57
-107.59
-107.42
-107.46
-107.50
-107.54
-107.58
-107.62
-107.66
-107.70
-107.74
-107.78
f1 (ppm)
20
10
0
-10
-20
-30
-40
-50
-60
-70
-80
-90
-100
-110
-120
-130
-140
-150
f1 (ppm)

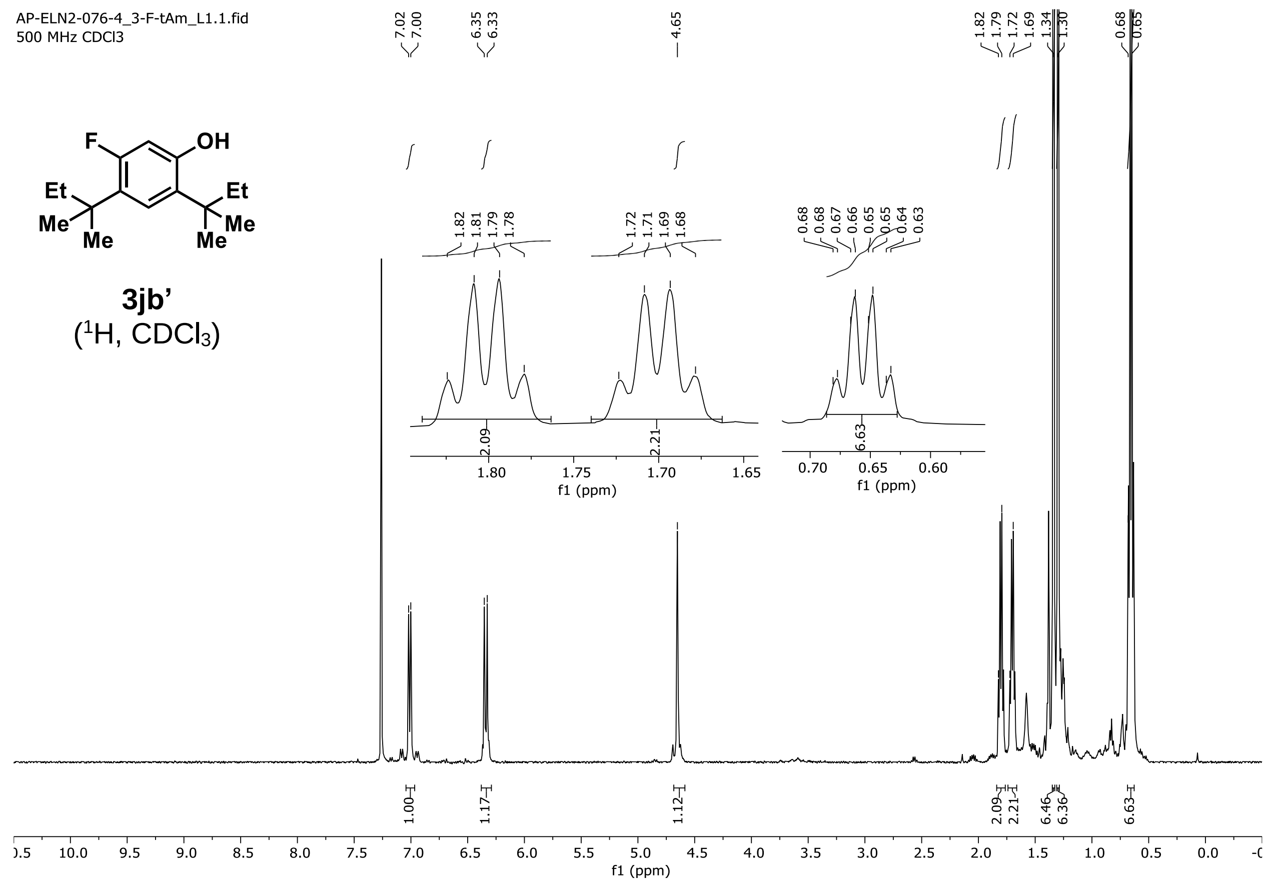
AP-ELN2-076-4_3-F-tAm_L1.1.fid
500 MHz CDCl3
7.02
7.00
6.35
6.33
4.65
1.82
1.79
1.72
1.69
1.34
1.30
0.68
0.65
F
OH
Et
Et
Me
Me
Me
Me
3jb’
(1H, CDCl3)
1.82
1.81
1.79
1.78
1.72
1.71
1.69
1.68
0.68
0.68
0.67
0.66
0.65
0.65
0.64
0.63
2.09
2.21
6.63
f1 (ppm)
1.00
1.17
1.12
2.09
2.21
6.46
6.36
6.63
f1 (ppm)

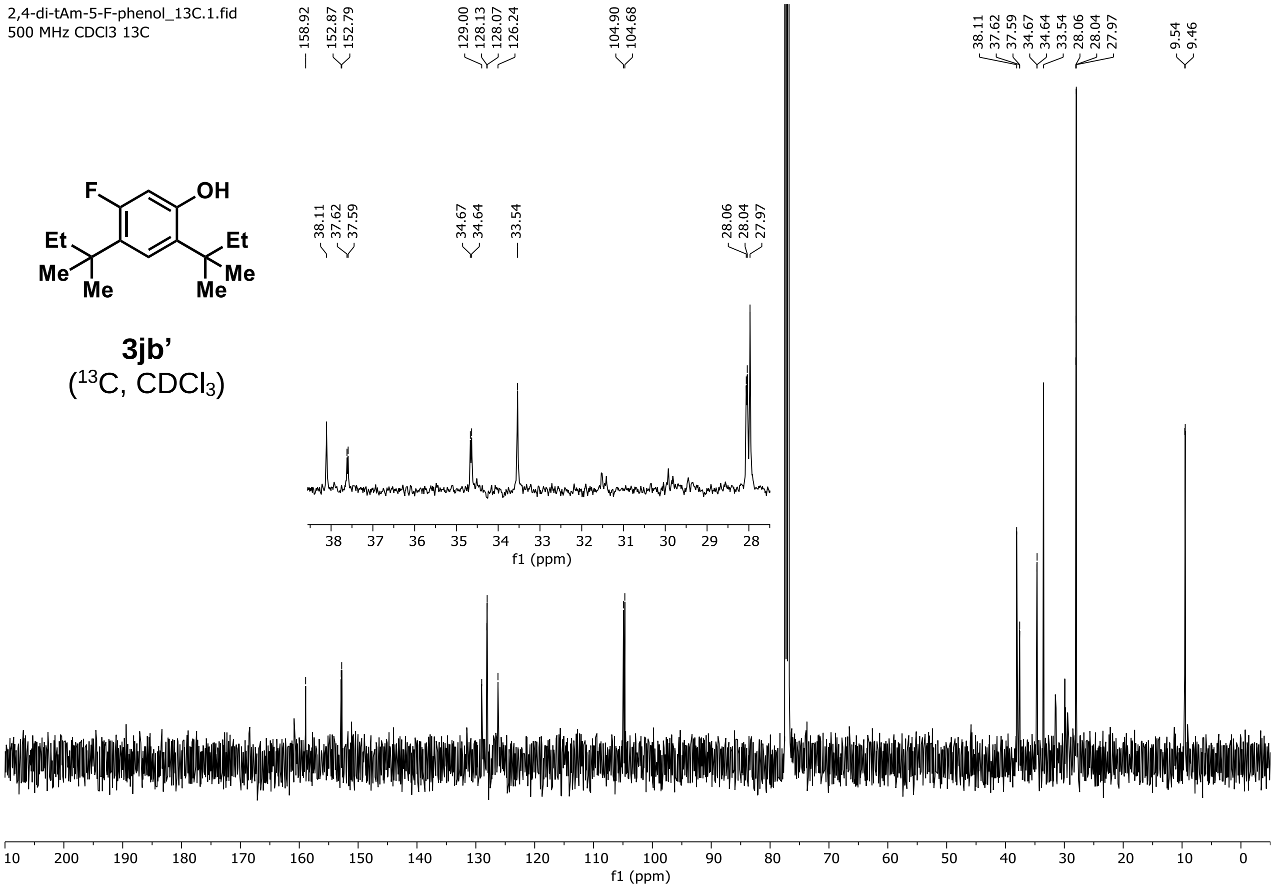
2,4-di-tAm-5-F-phenol_13C.1.fid
500 MHz CDCl3 13C
158.92
152.87
152.79
129.00
128.13
128.07
126.24
104.90
104.68
38.11
37.62
37.59
34.67
34.64
33.54
28.06
28.04
27.97
9.54
9.46
F
OH
Et
Et
Me
Me
Me
Me
3jb’
(13C, CDCl3)
38.11
37.62
37.59
34.67
34.64
33.54
28.06
28.04
27.97
f1 (ppm)
f1 (ppm)

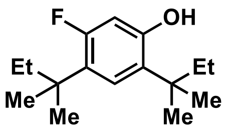

3jb'
(^{19}F, $CDCl_3$)

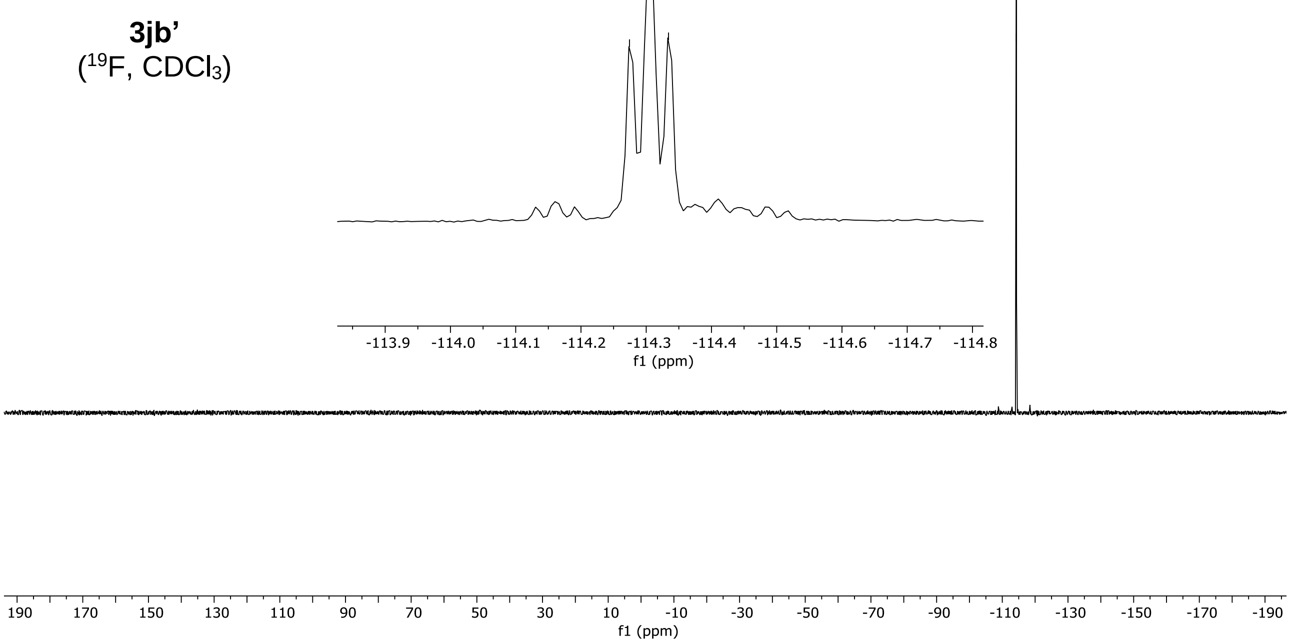

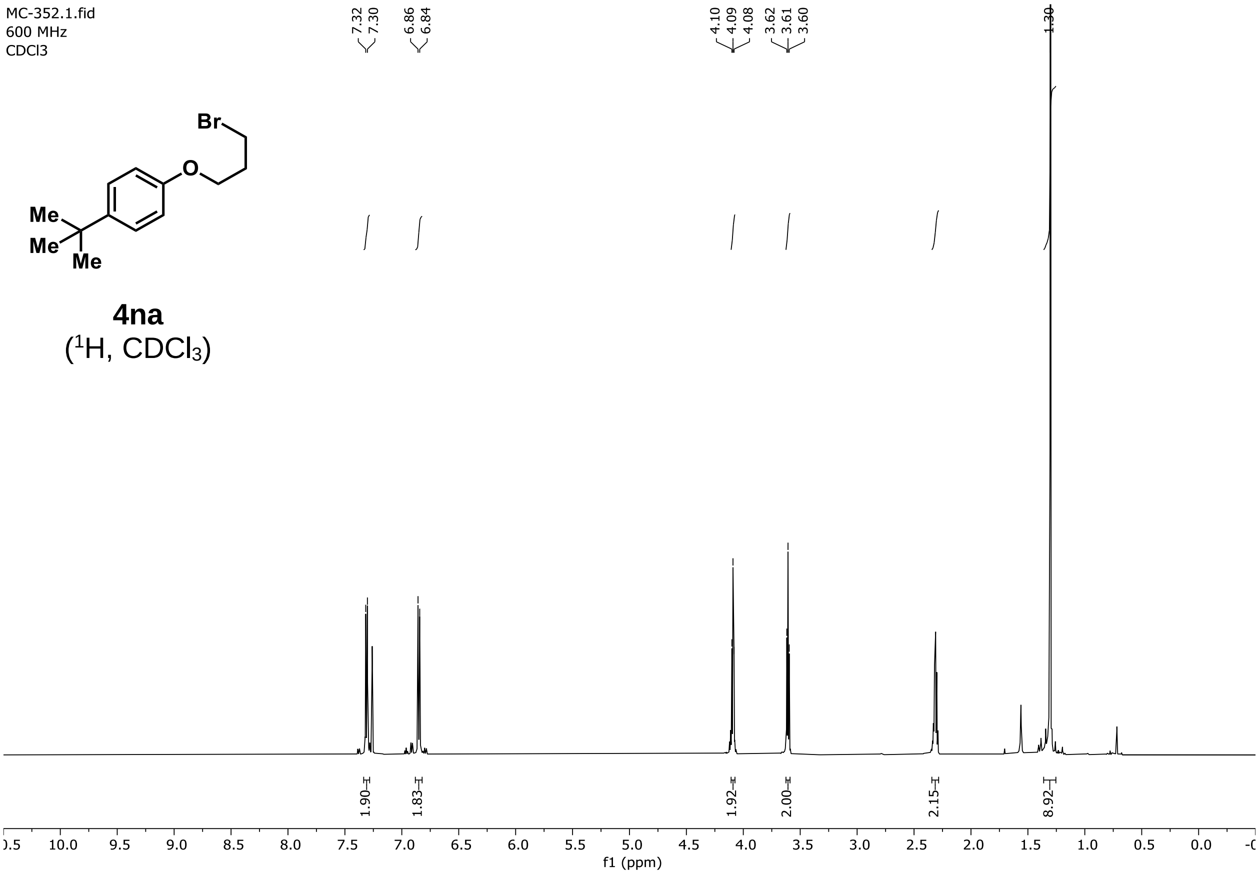

MC-352.1.fid
600 MHz
CDCl3
7.32
7.30
6.86
6.84
4.10
4.09
4.08
3.62
3.61
3.60
1.30
Br
O
Me
Me
Me
4na
(1H, CDCl3)
1.90
1.83
1.92
2.00
2.15
8.92
f1 (ppm)

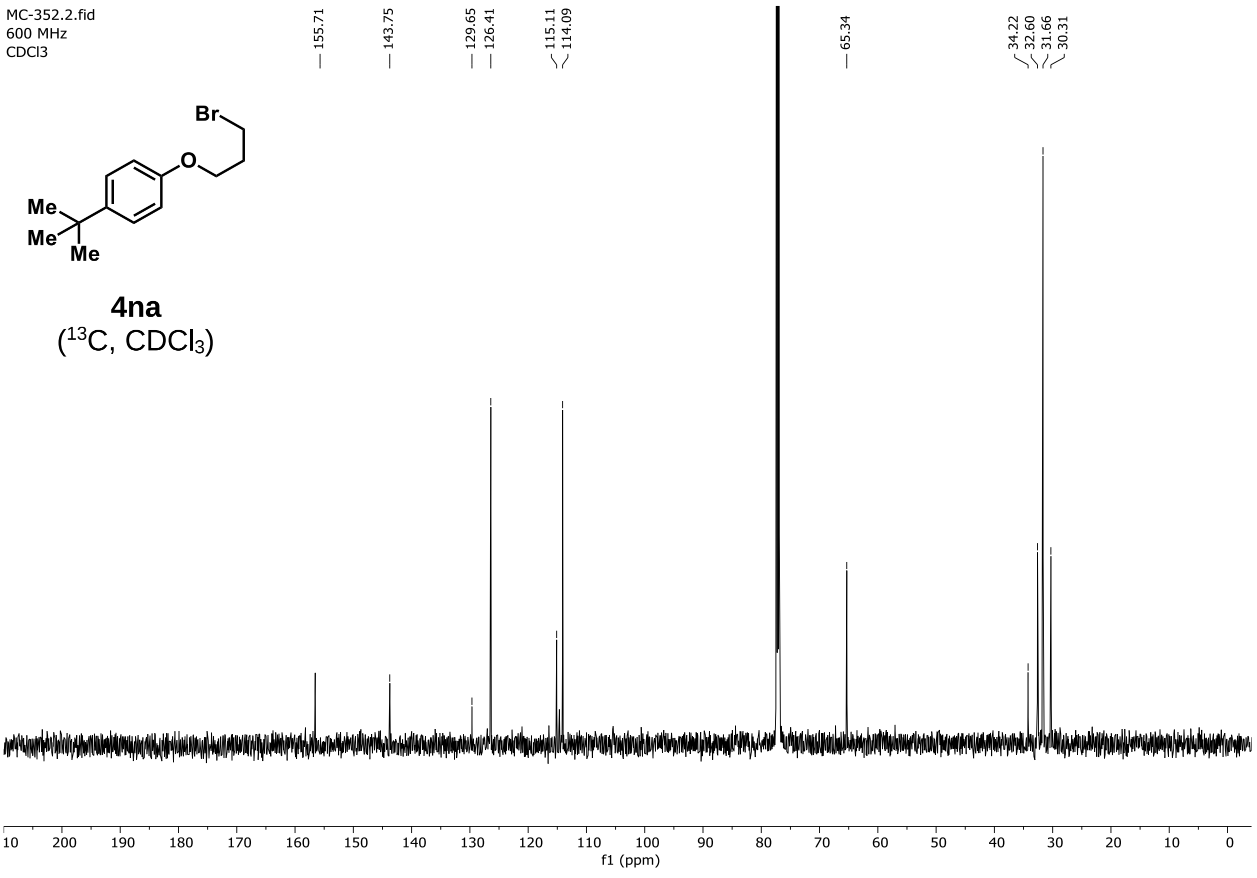

MC-352.2.fid
600 MHz
CDCl3
155.71
143.75
129.65
126.41
115.11
114.09
65.34
34.22
32.60
31.66
30.31
Br
O
Me
Me
Me
4na
(13C, CDCl3)
f1 (ppm)

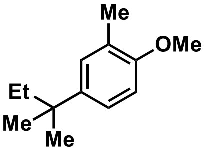

4ob
(^{1}H, $CDCl_3$)

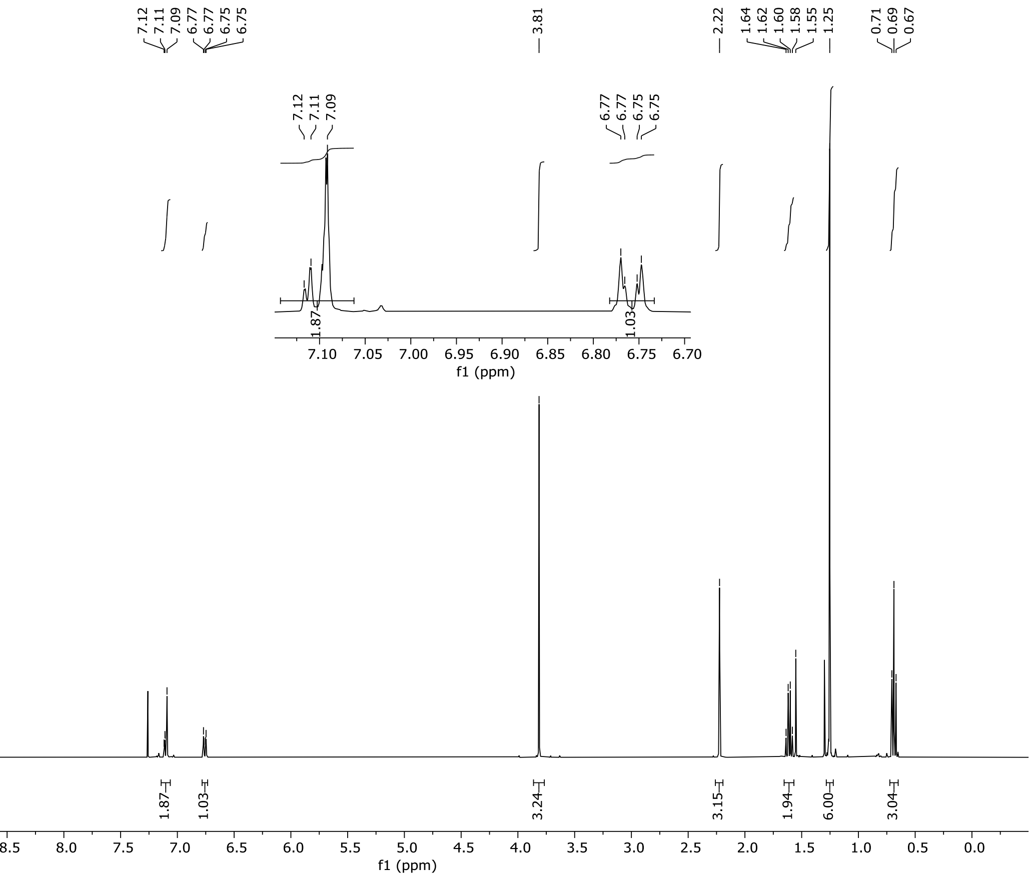

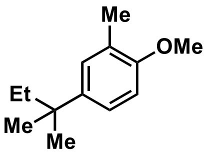

4ob
(^{13}C, $CDCl_3$)

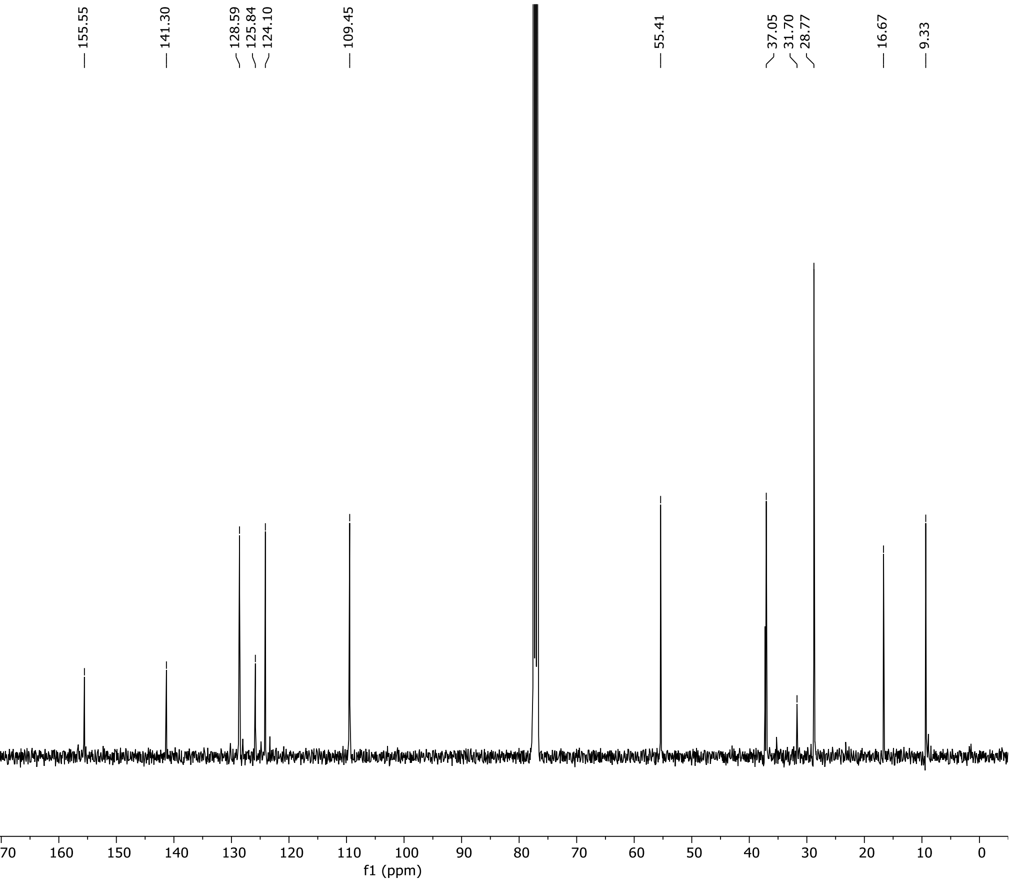

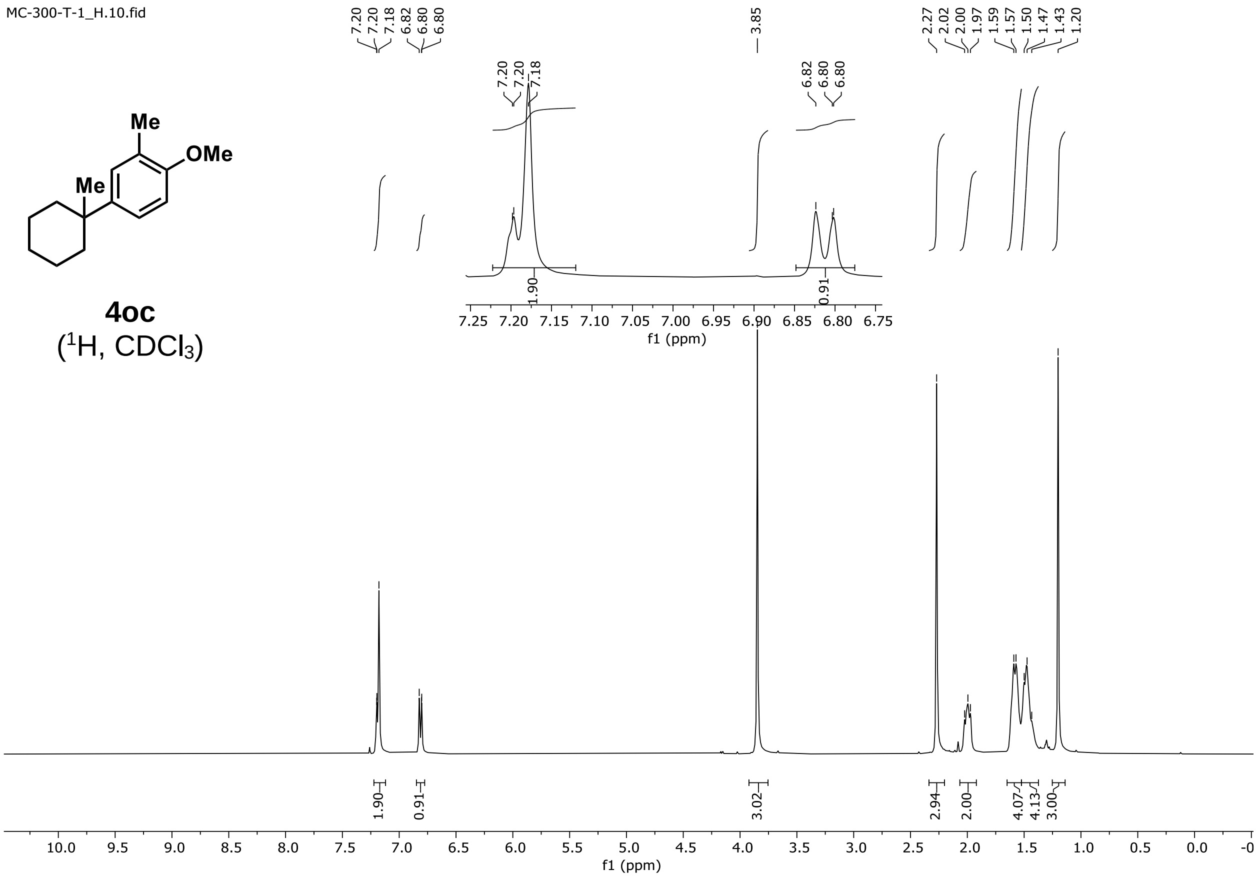

4oc
(^{1}H, $CDCl_3$)

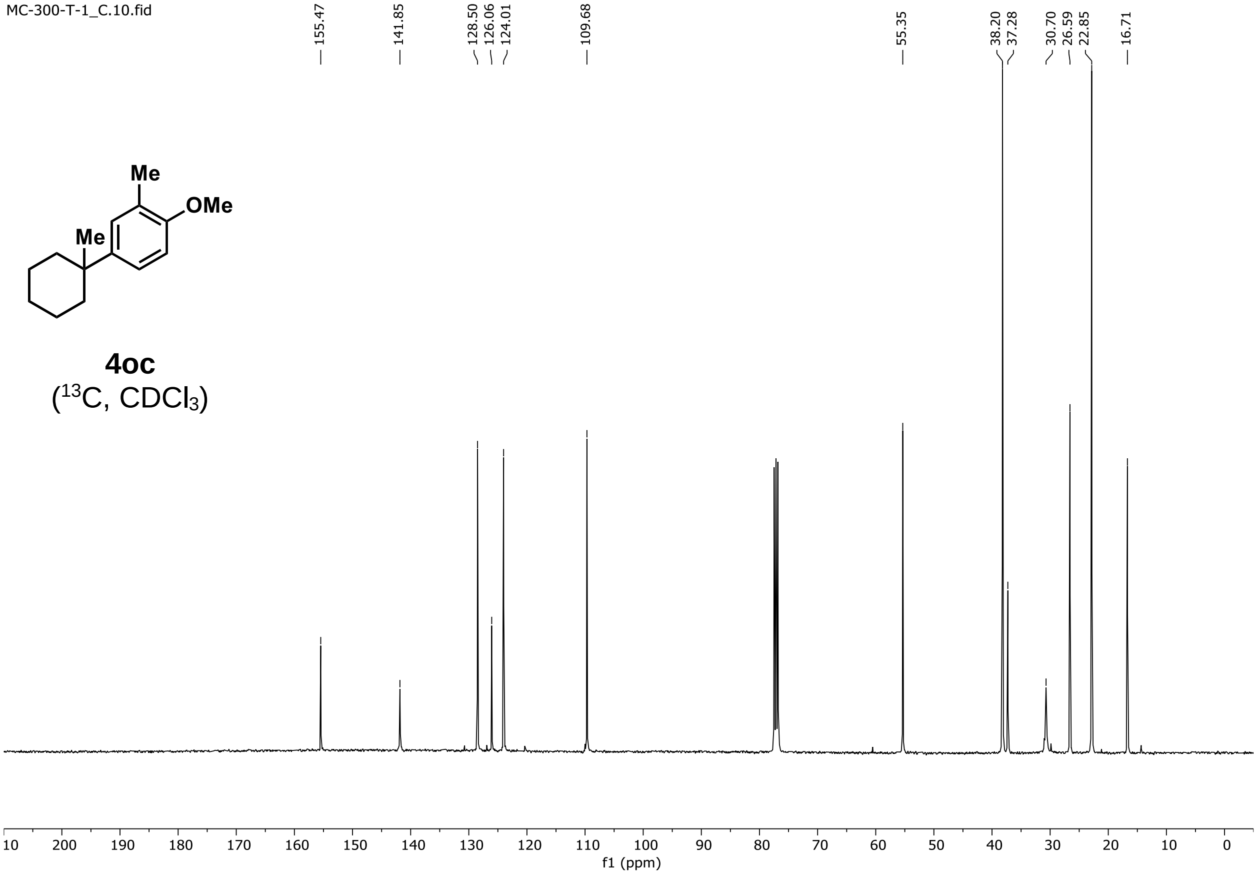

4oc
(^{13}C, $CDCl_3$)

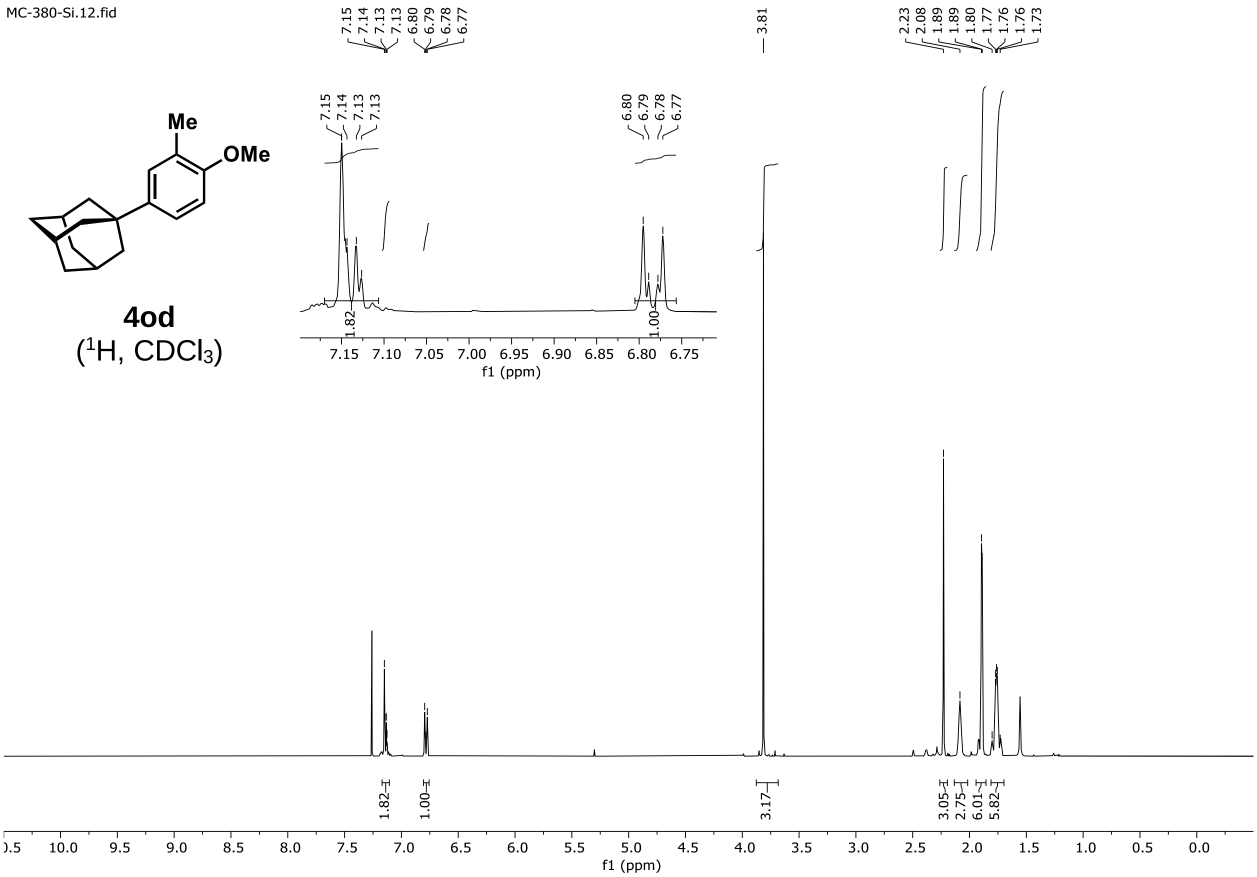
MC-380-Si.12.fid
7.15 7.14 7.13 7.13 6.80 6.79 6.78 6.77
3.81
2.23 2.08 1.89 1.89 1.80 1.77 1.76 1.76 1.73
Me
OMe
4od
(^{1}H, CDCl$_3$)
1.82
1.00
f1 (ppm)
1.82
1.00
3.17
3.05
2.75
6.01
5.82
f1 (ppm)

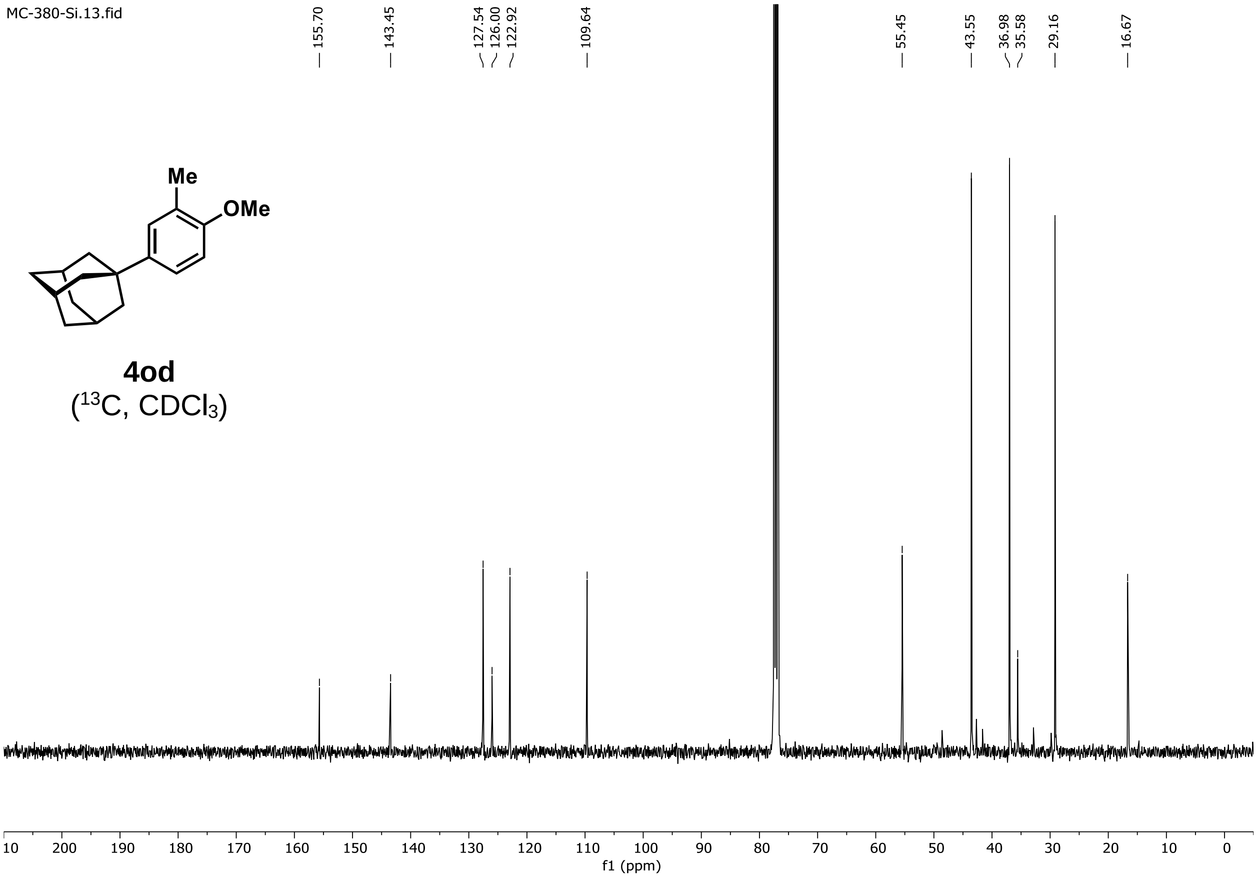
MC-380-Si.13.fid
155.70
143.45
127.54 126.00 122.92
109.64
55.45
43.55
36.98 35.58
29.16
16.67
Me
OMe
4od
(^{13}C, CDCl$_3$)
f1 (ppm)

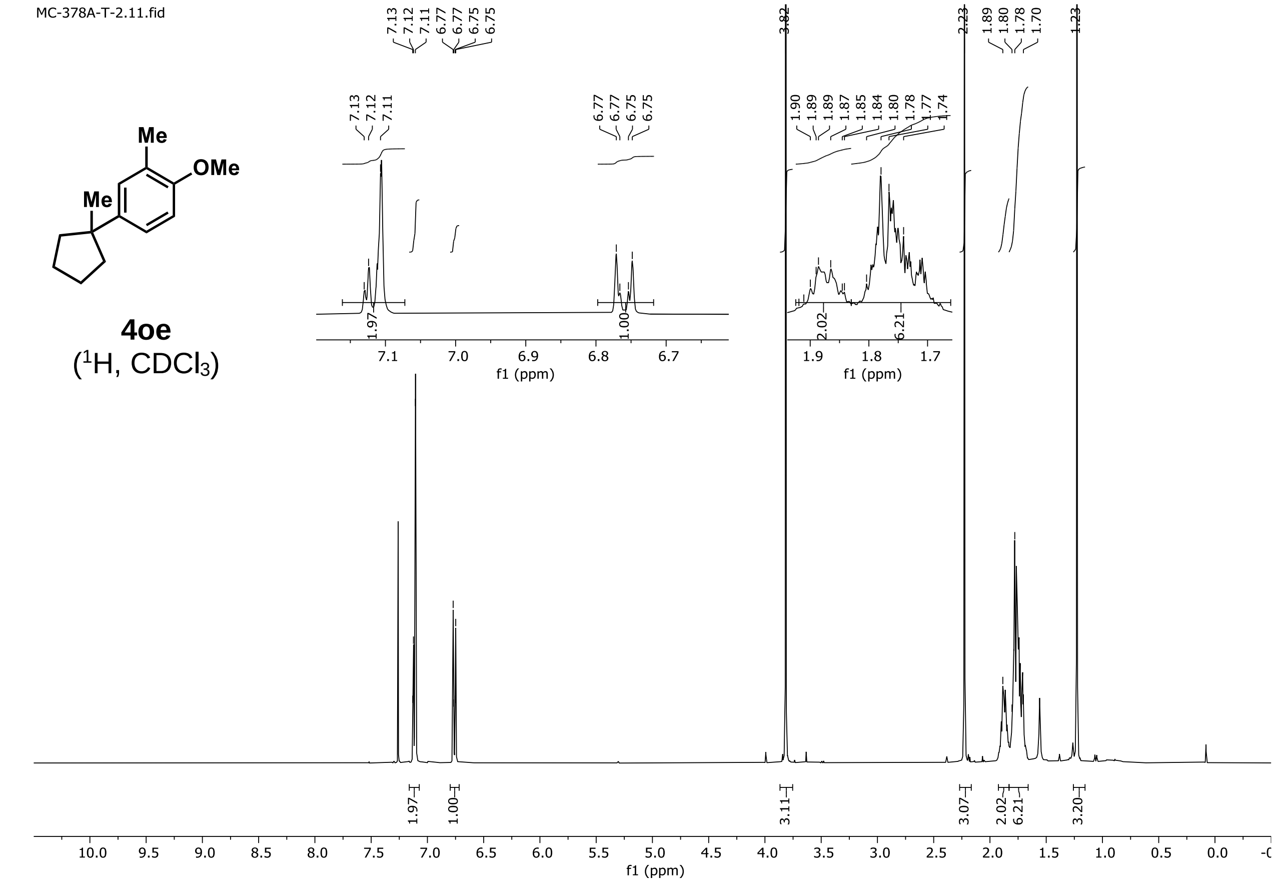

4oe
(^{1}H, $CDCl_3$)

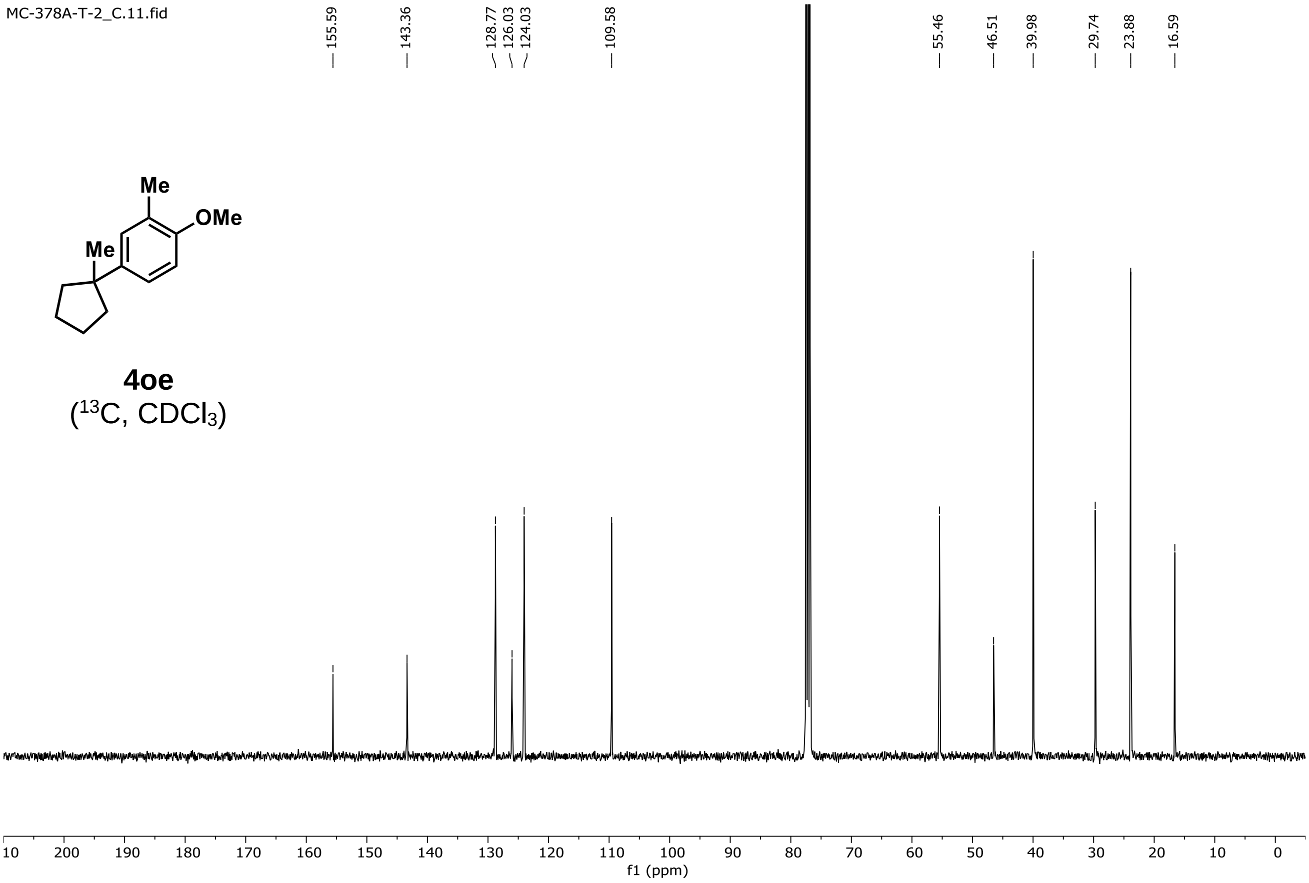

4oe
(^{13}C, $CDCl_3$)

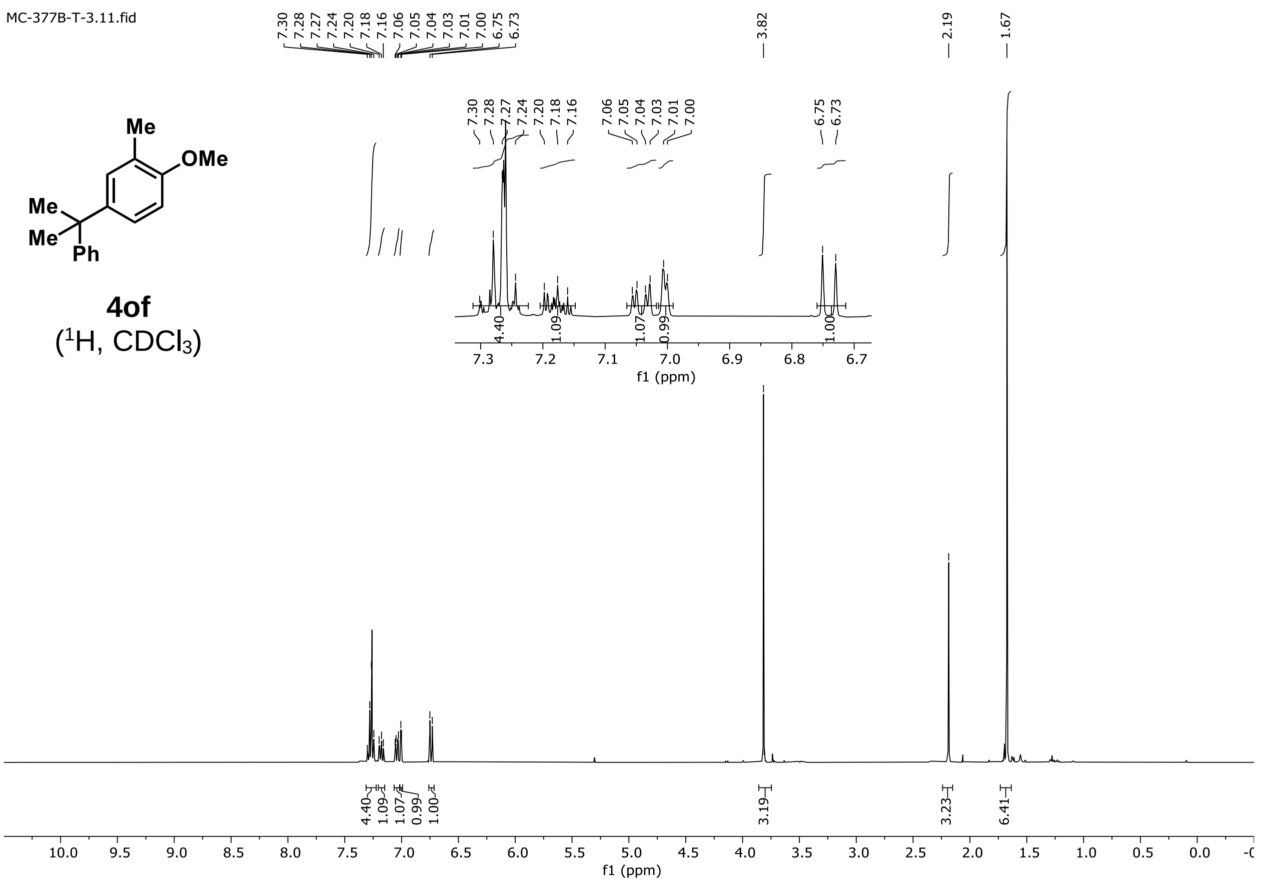
MC-377B-T-3.11.fid
7.30 7.28 7.27 7.24 7.20 7.18 7.16 7.06 7.05 7.04 7.03 7.01 7.00 6.75 6.73
3.82
2.19
1.67
Me
OMe
Me
Me
Ph
4of
(^{1}H, CDCl$_3$)
4.40 1.09 1.07 0.99 1.00
3.19 3.23 6.41
f1 (ppm)

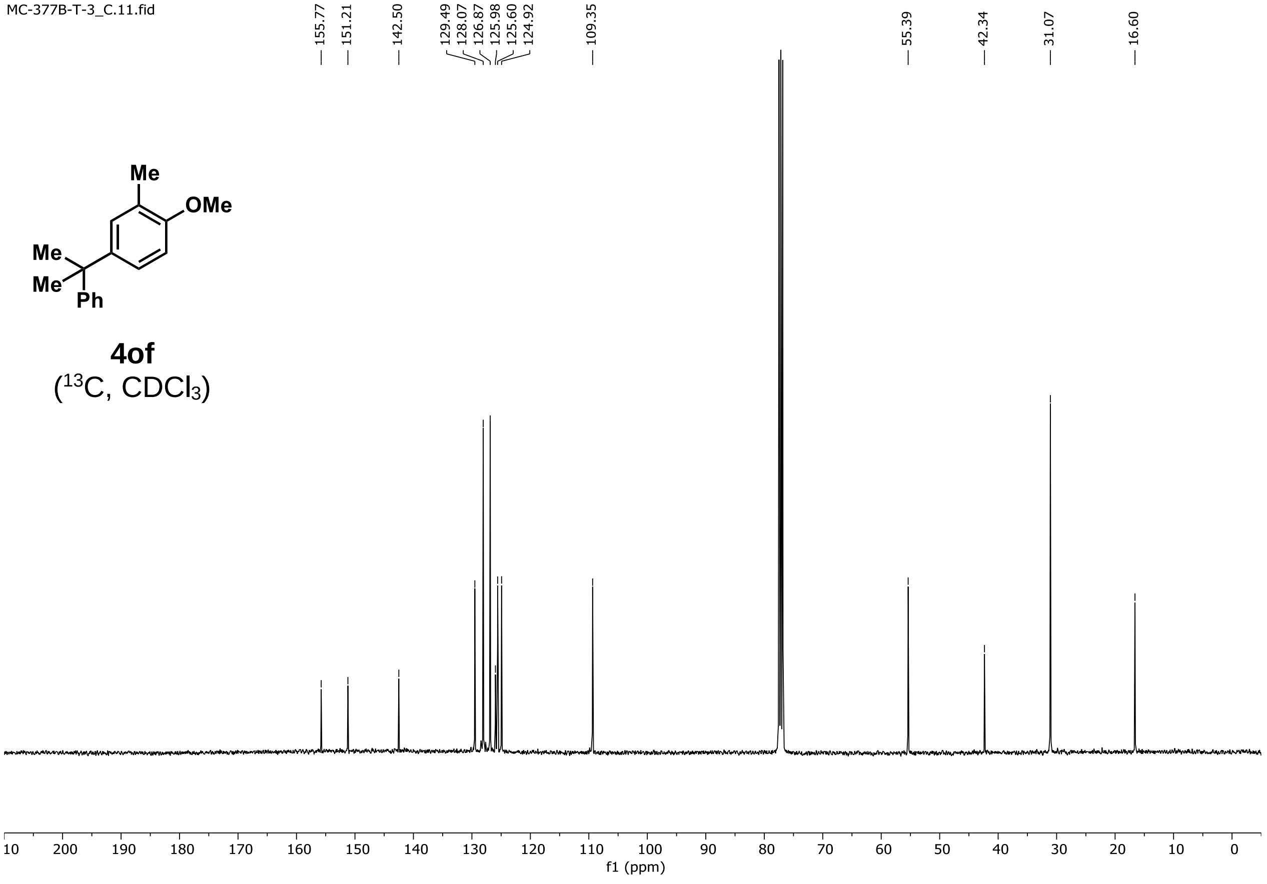
MC-377B-T-3_C.11.fid
155.77
151.21
142.50
129.49 128.07 126.87 125.98 125.60 124.92
109.35
55.39
42.34
31.07
16.60
Me
OMe
Me
Me
Ph
4of
(^{13}C, CDCl$_3$)
f1 (ppm)

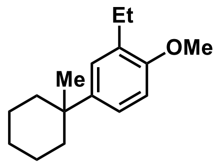

4pc
(^{1}H, $CDCl_3$)

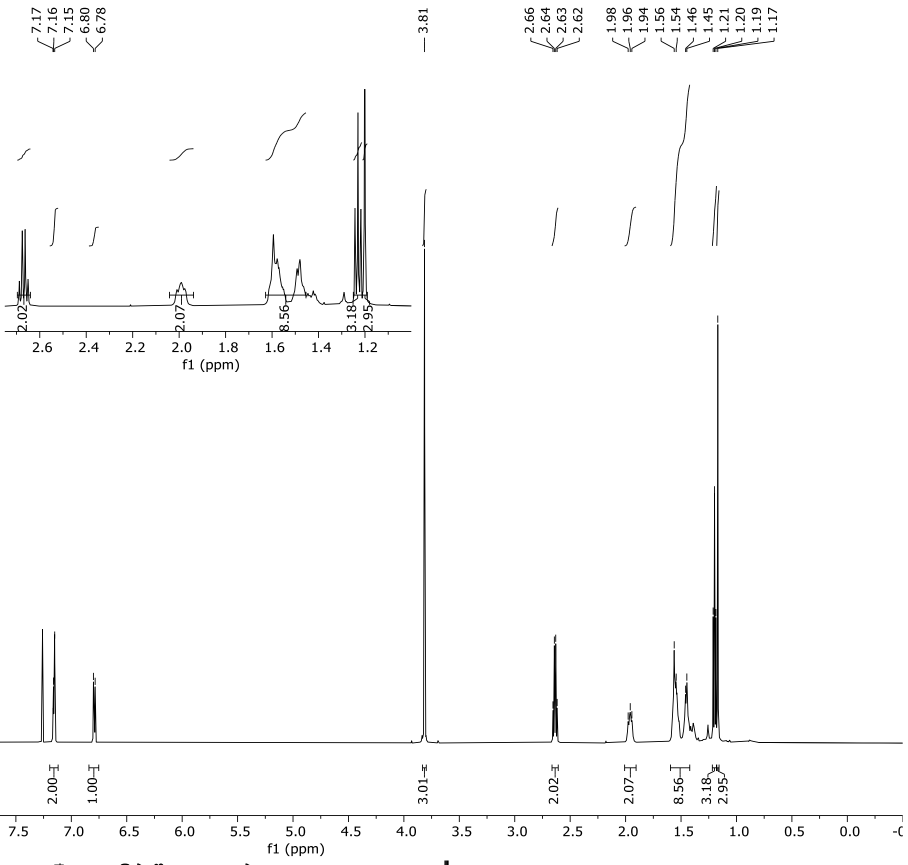

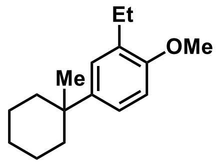

4pc
(^{13}C, $CDCl_3$)

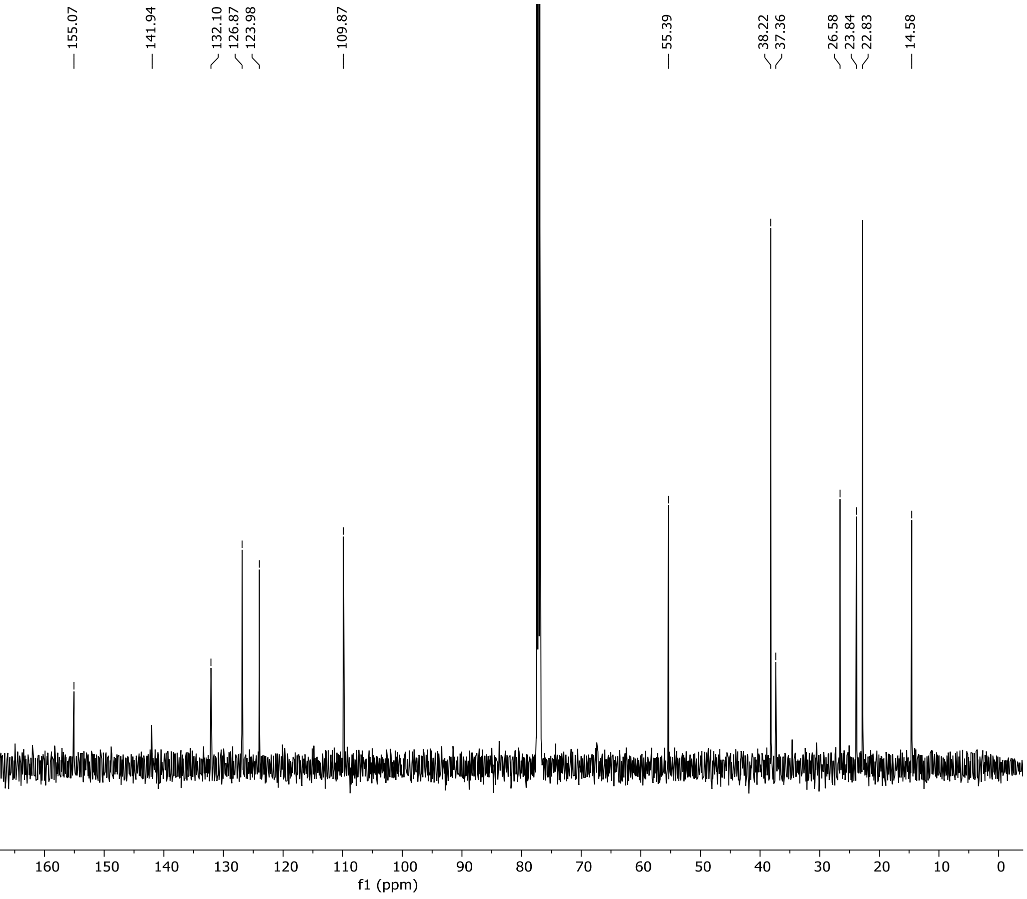

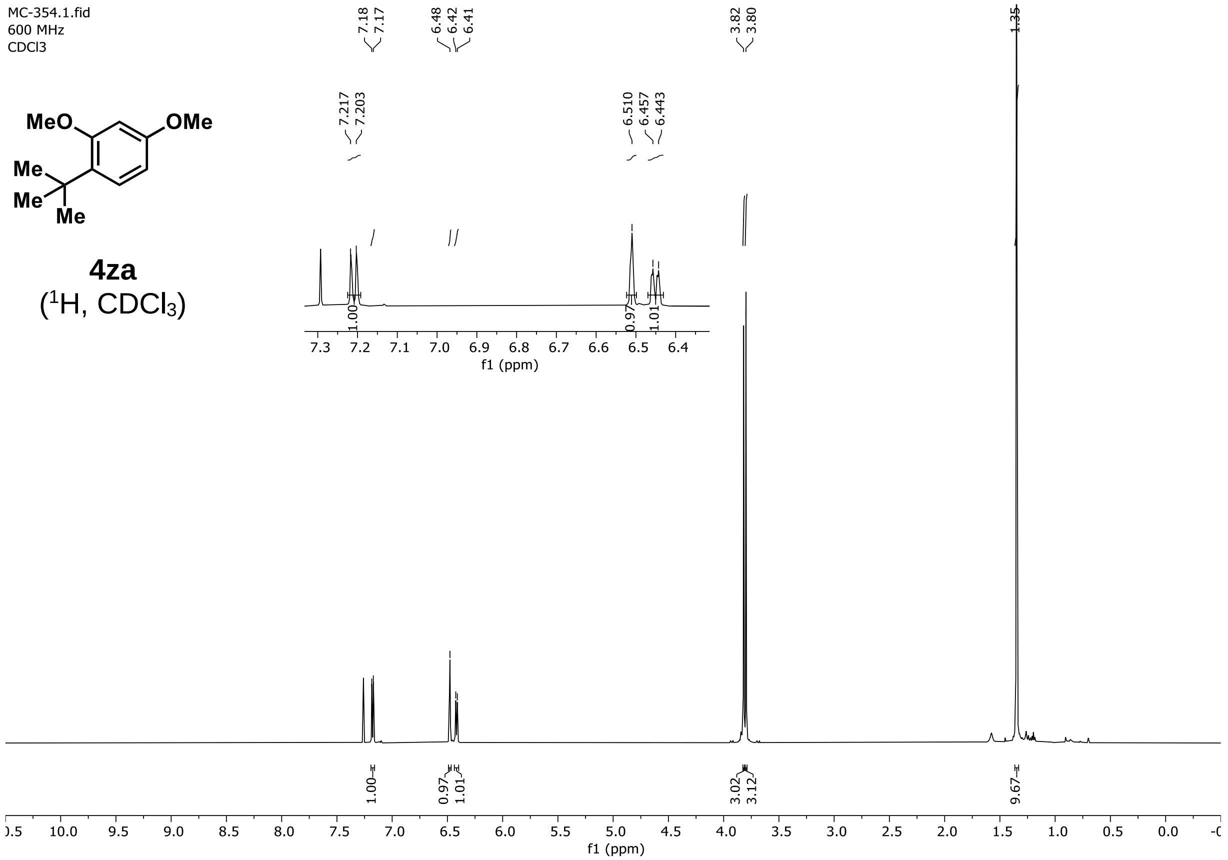

4za
(^{1}H, $CDCl_3$)

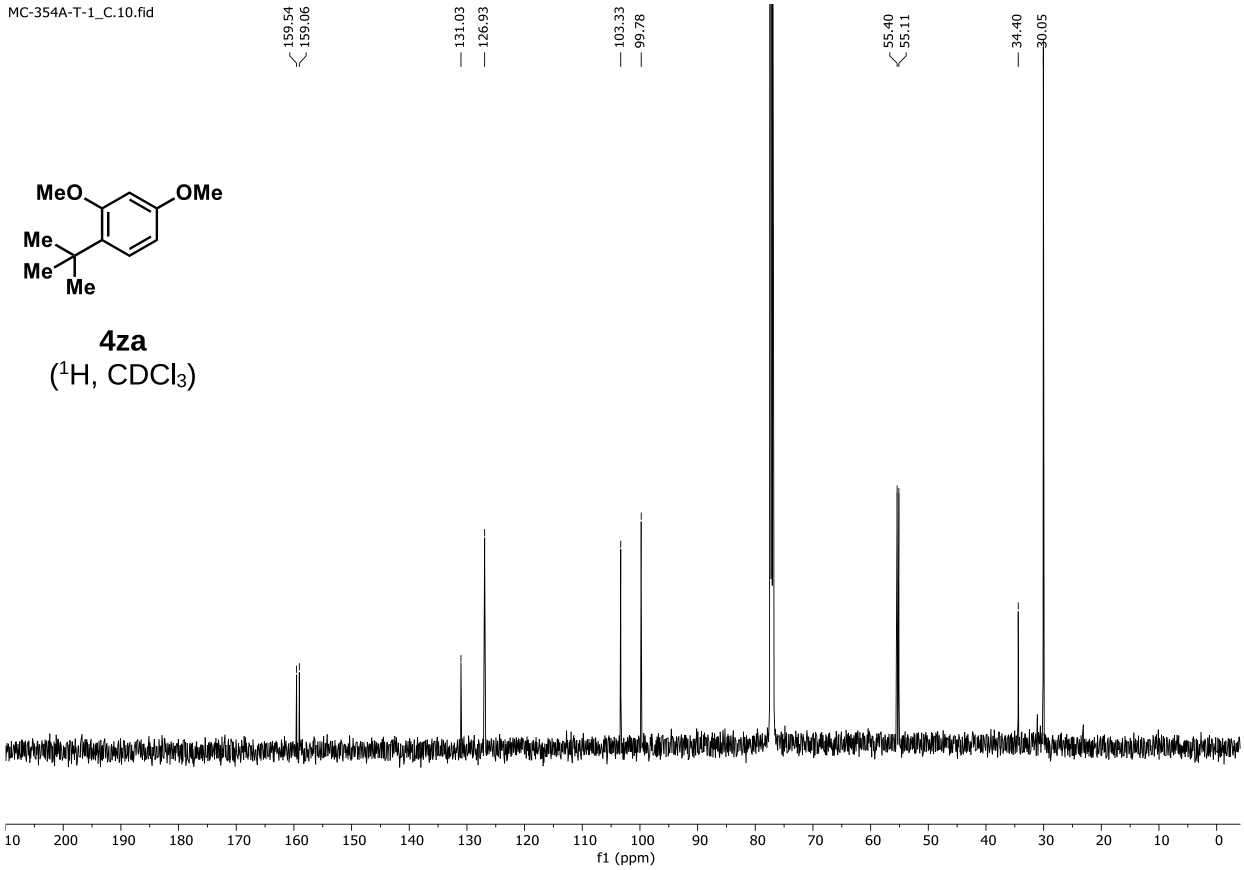

4za
(^{1}H, $CDCl_3$)

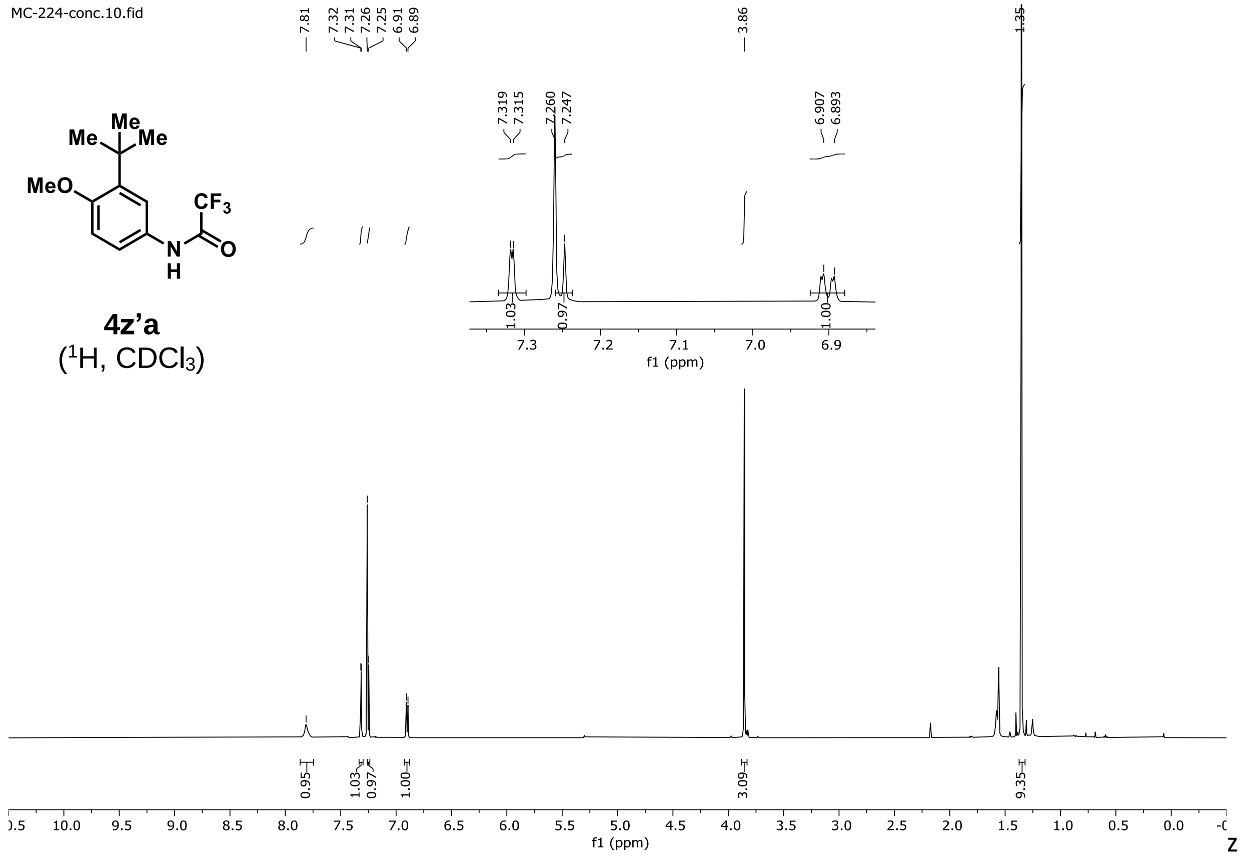

4z’a
(^{1}H, $CDCl_3$)

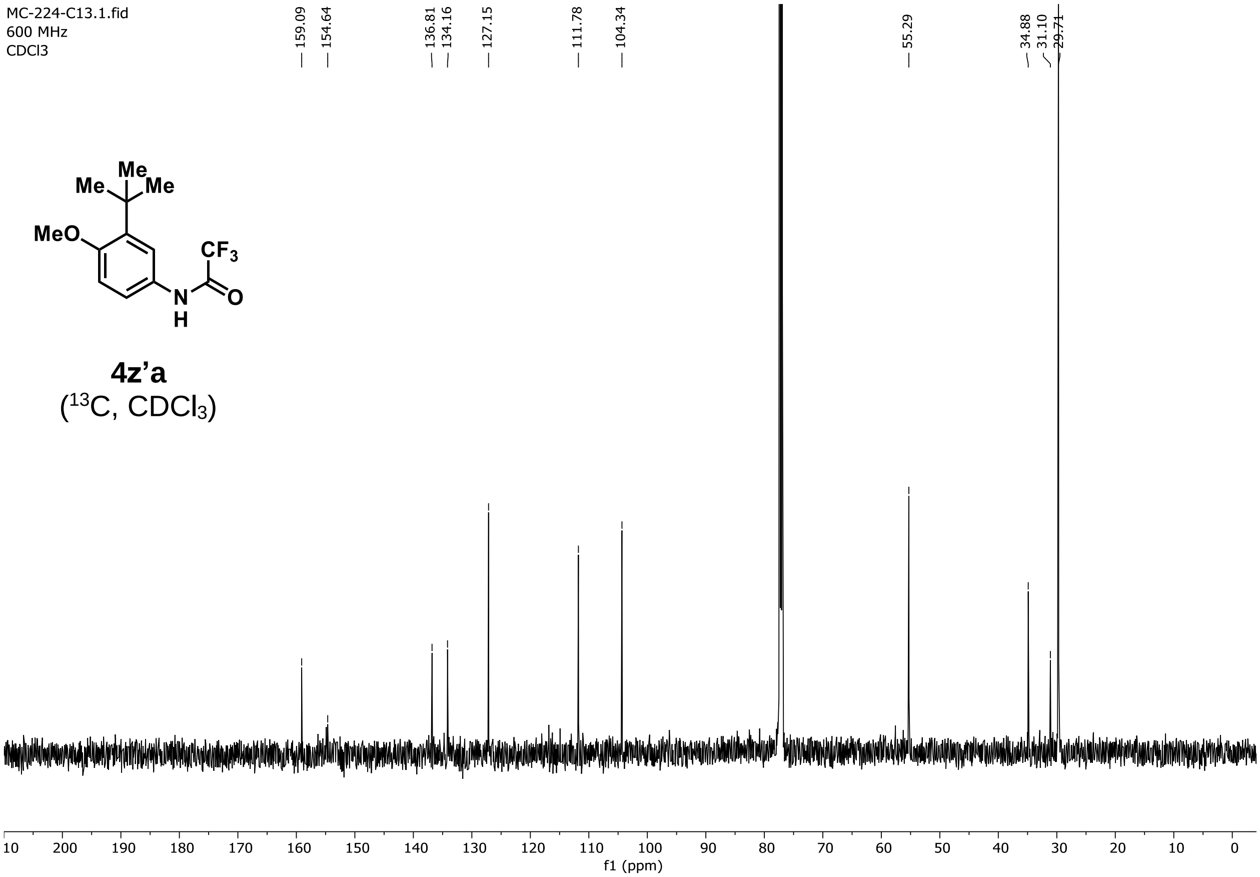

4z’a
(^{13}C, $CDCl_3$)

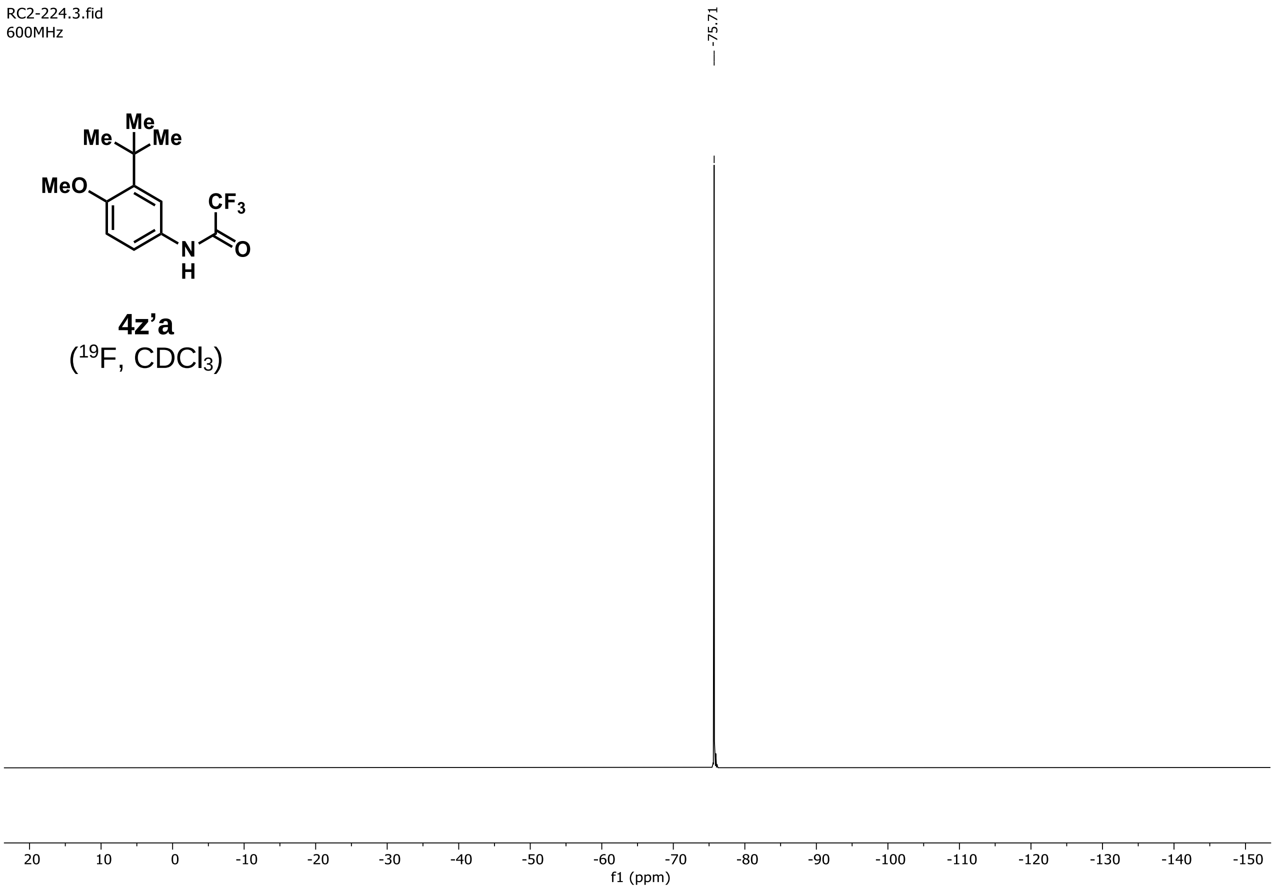
RC2-224.3.fid
600MHz
-75.71
Me
Me
Me
MeO
CF3
N
H
O
4z'a
(19F, CDCl3)
20
10
0
-10
-20
-30
-40
-50
-60
-70
-80
-90
-100
-110
-120
-130
-140
-150
f1 (ppm)

MC-355B-T-1.13.fid

4z"a
(^{1}H, $CDCl_3$)

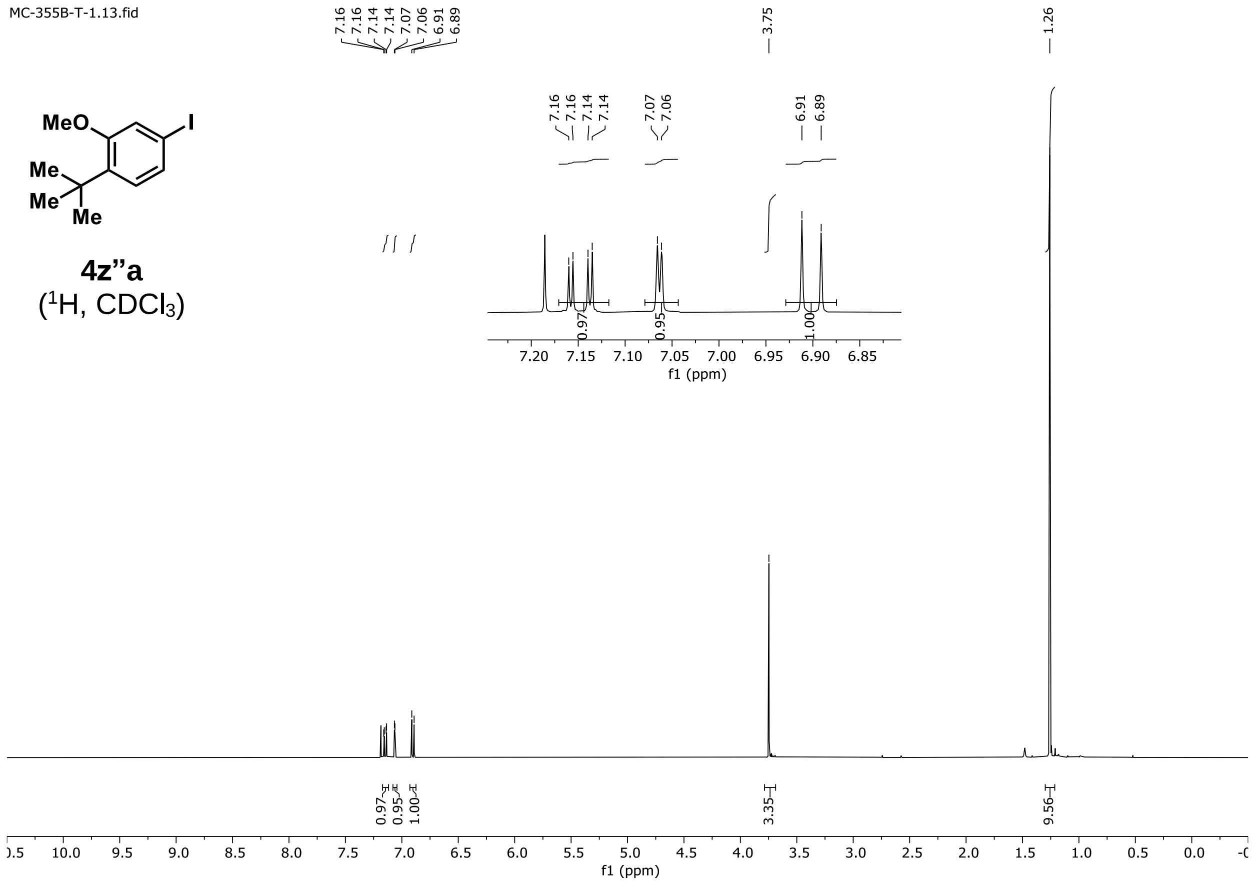

MC-355B-T-1.14.fid

4z"a
(^{13}C, $CDCl_3$)

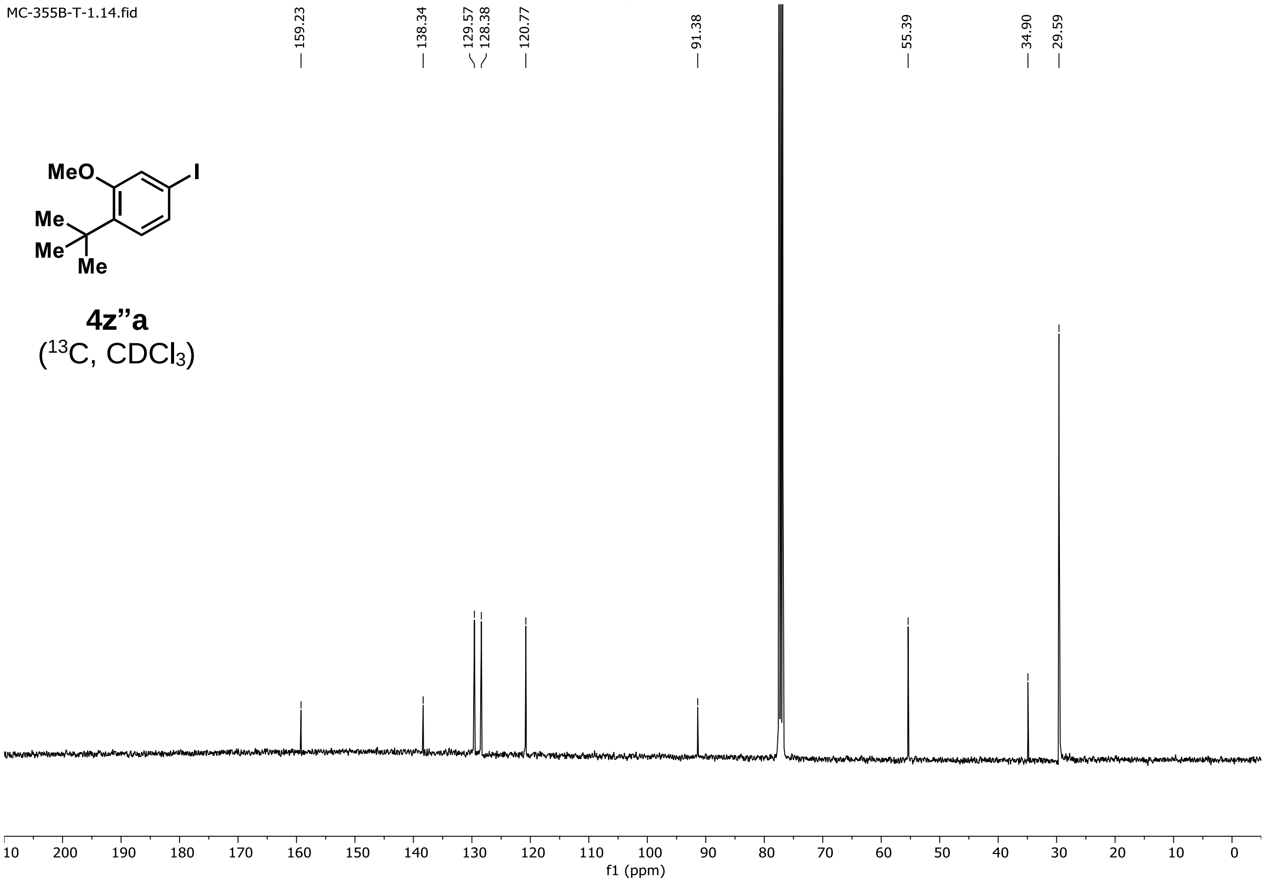

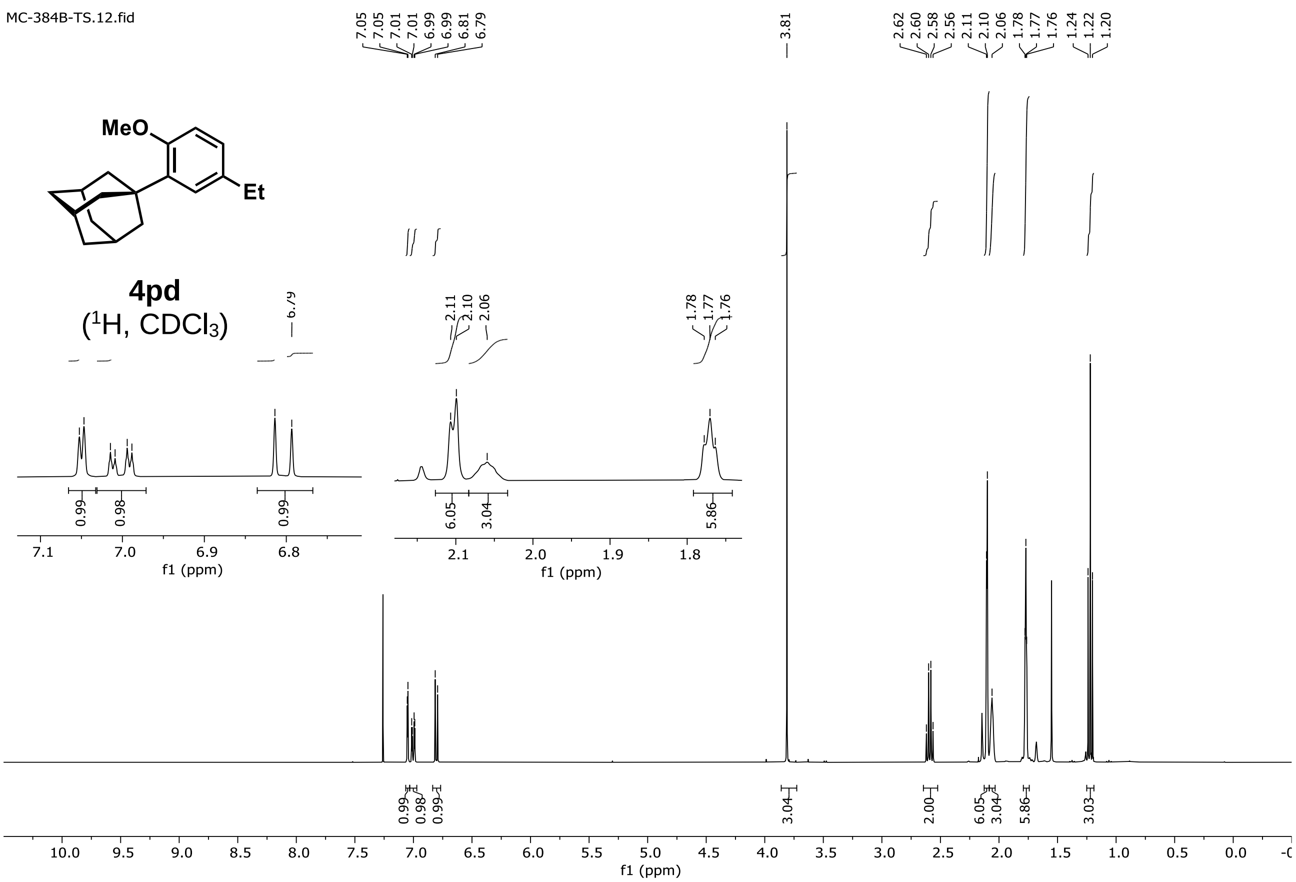
MC-384B-TS.12.fid
MeO
Et
4pd
(^{1}H, CDCl$_3$)
7.05 7.05 7.01 7.01 6.99 6.99 6.81 6.79
3.81
2.62 2.60 2.58 2.56 2.11 2.10 2.06 1.78 1.77 1.76 1.24 1.22 1.20
0.99 0.98 0.99 3.04 2.00 6.05 3.04 5.86 3.03
f1 (ppm)

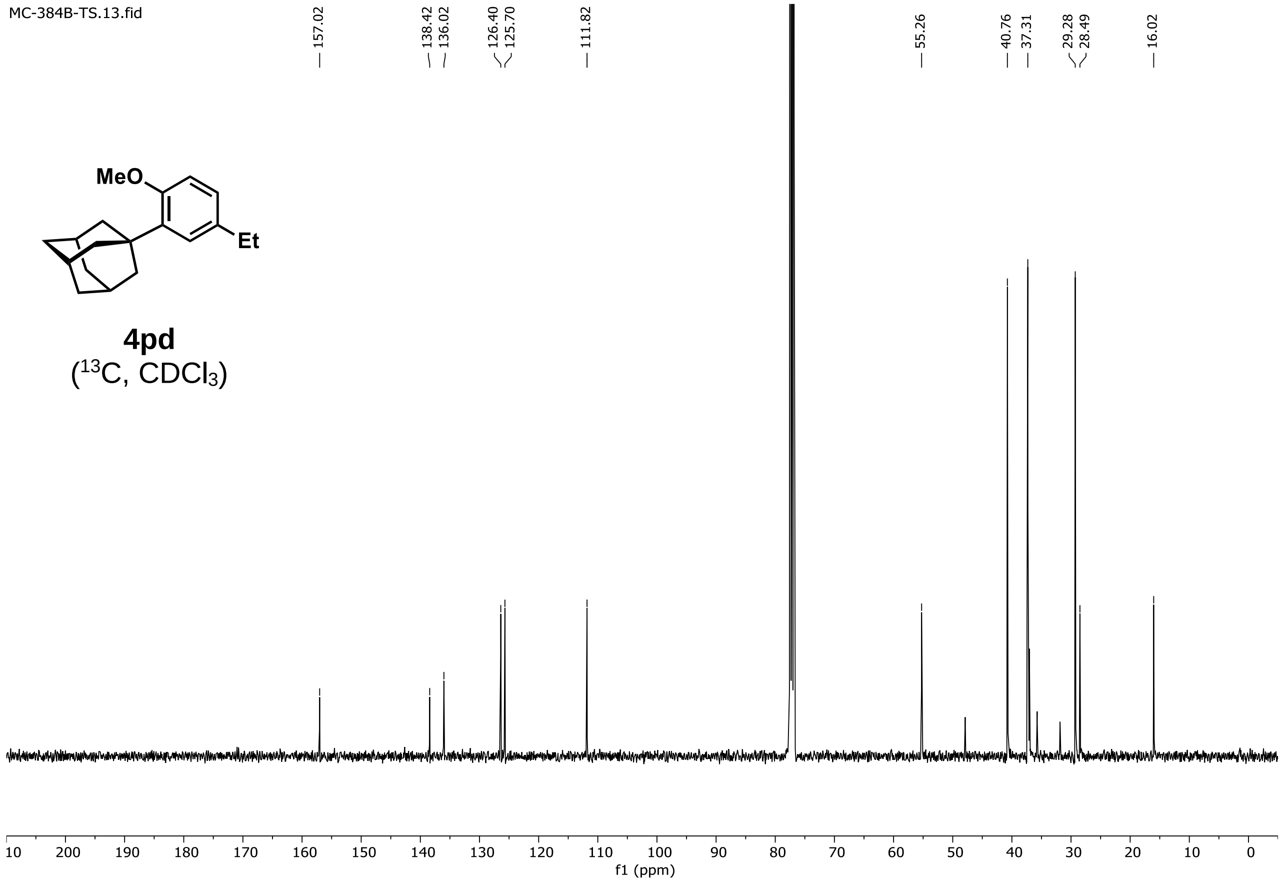
MC-384B-TS.13.fid
157.02
138.42 136.02
126.40 125.70
111.82
55.26
40.76 37.31
29.28 28.49
16.02
MeO
Et
4pd
(^{13}C, CDCl$_3$)
f1 (ppm)

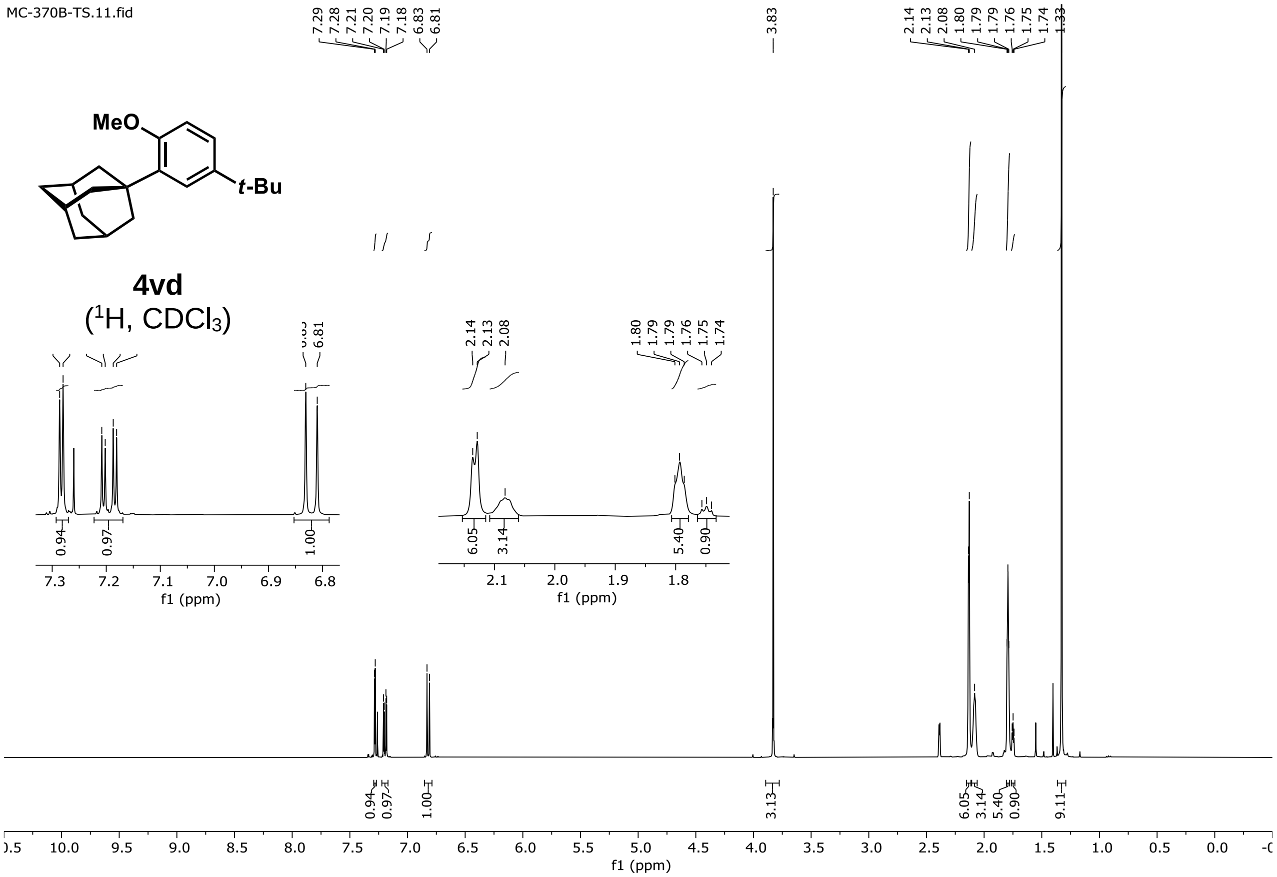
MC-370B-TS.11.fid
MeO
t-Bu
4vd
(1H, CDCl3)
7.29 7.28 7.21 7.20 7.19 7.18 6.83 6.81
3.83
2.14 2.13 2.08 1.80 1.79 1.79 1.76 1.75 1.74 1.33
0.94 0.97 1.00 3.13 6.05 3.14 5.40 0.90 9.11
f1 (ppm)

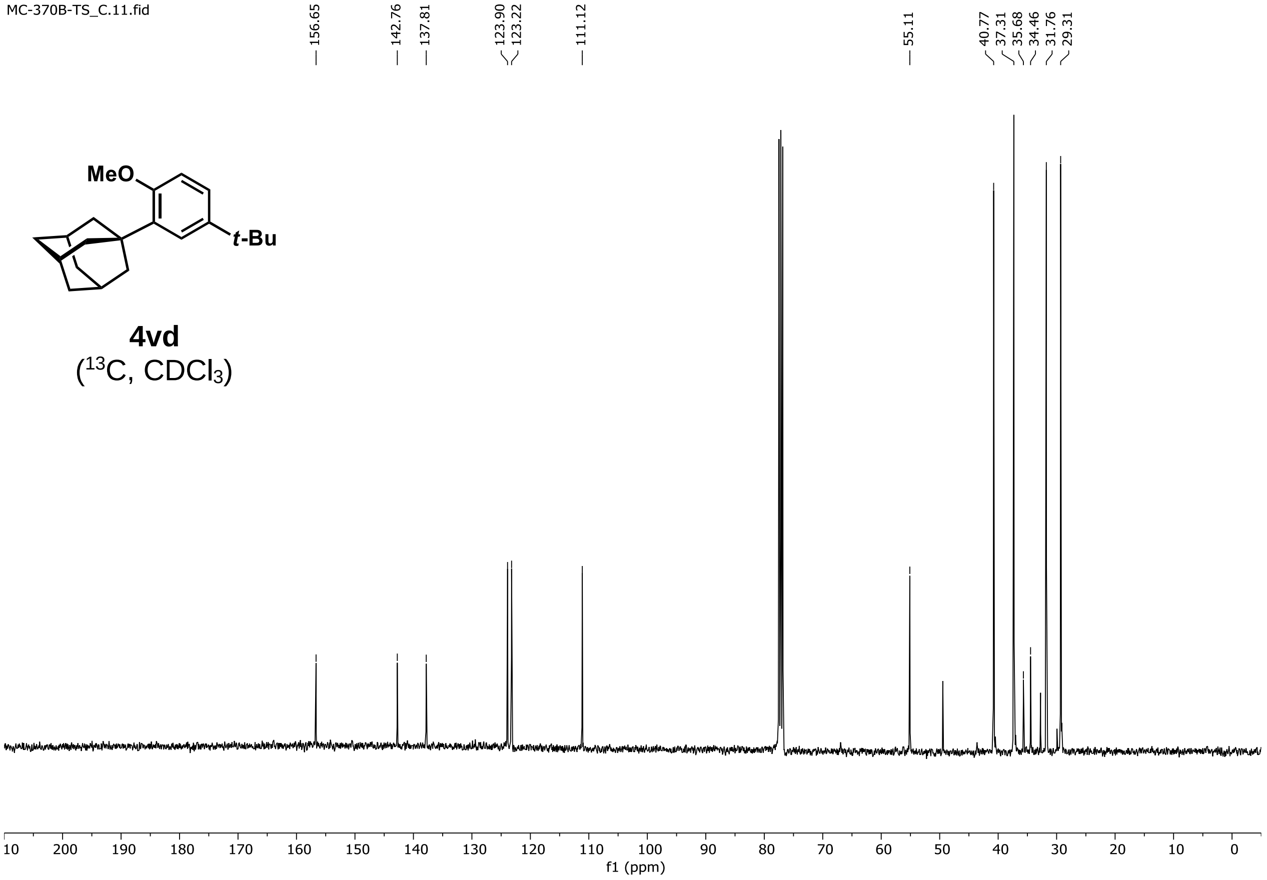
MC-370B-TS_C.11.fid
MeO
t-Bu
4vd
(13C, CDCl3)
156.65 142.76 137.81 123.90 123.22 111.12 55.11 40.77 37.31 35.68 34.46 31.76 29.31
f1 (ppm)

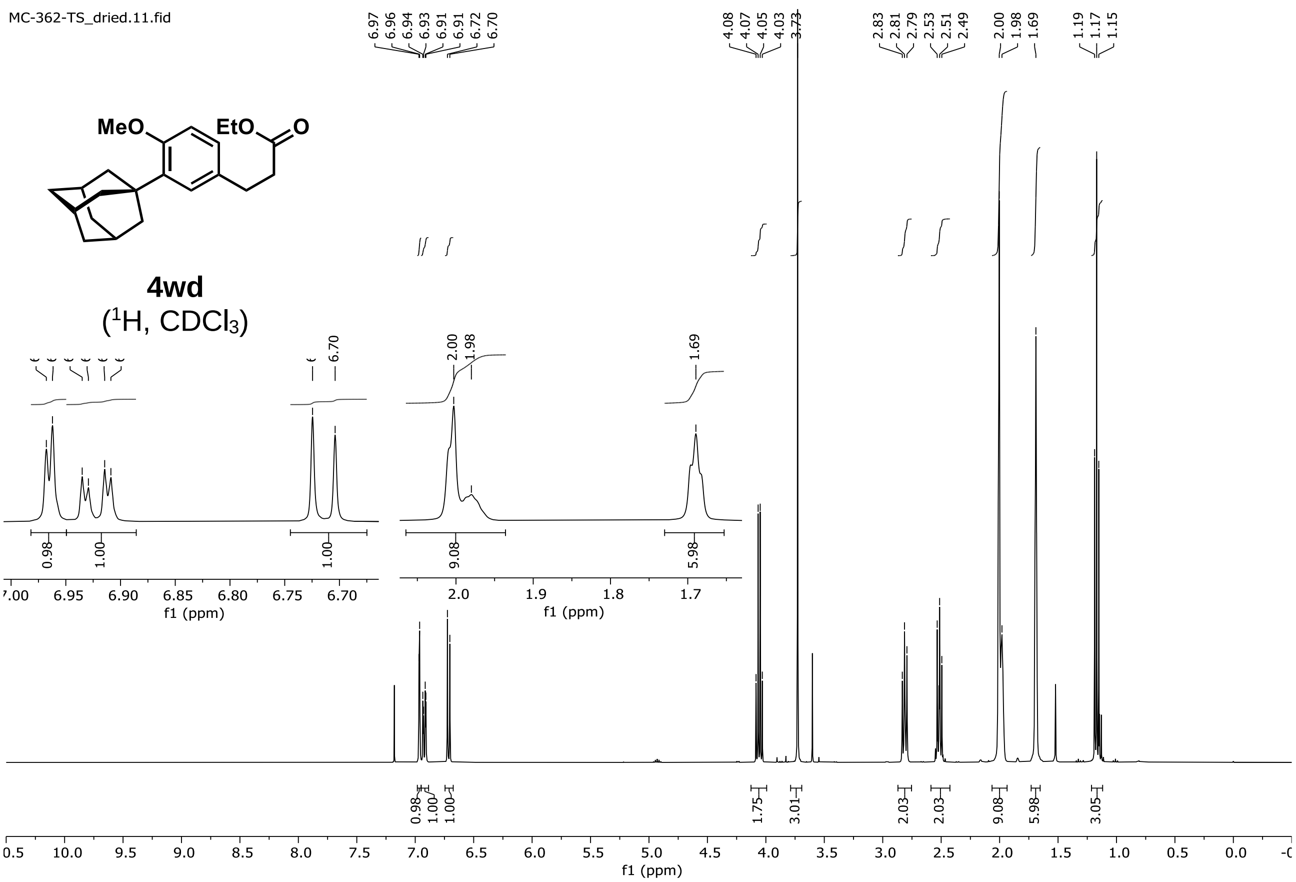
MC-362-TS_dried.11.fid
MeO
EtO
O
4wd
(¹H, CDCl3)
f1 (ppm)

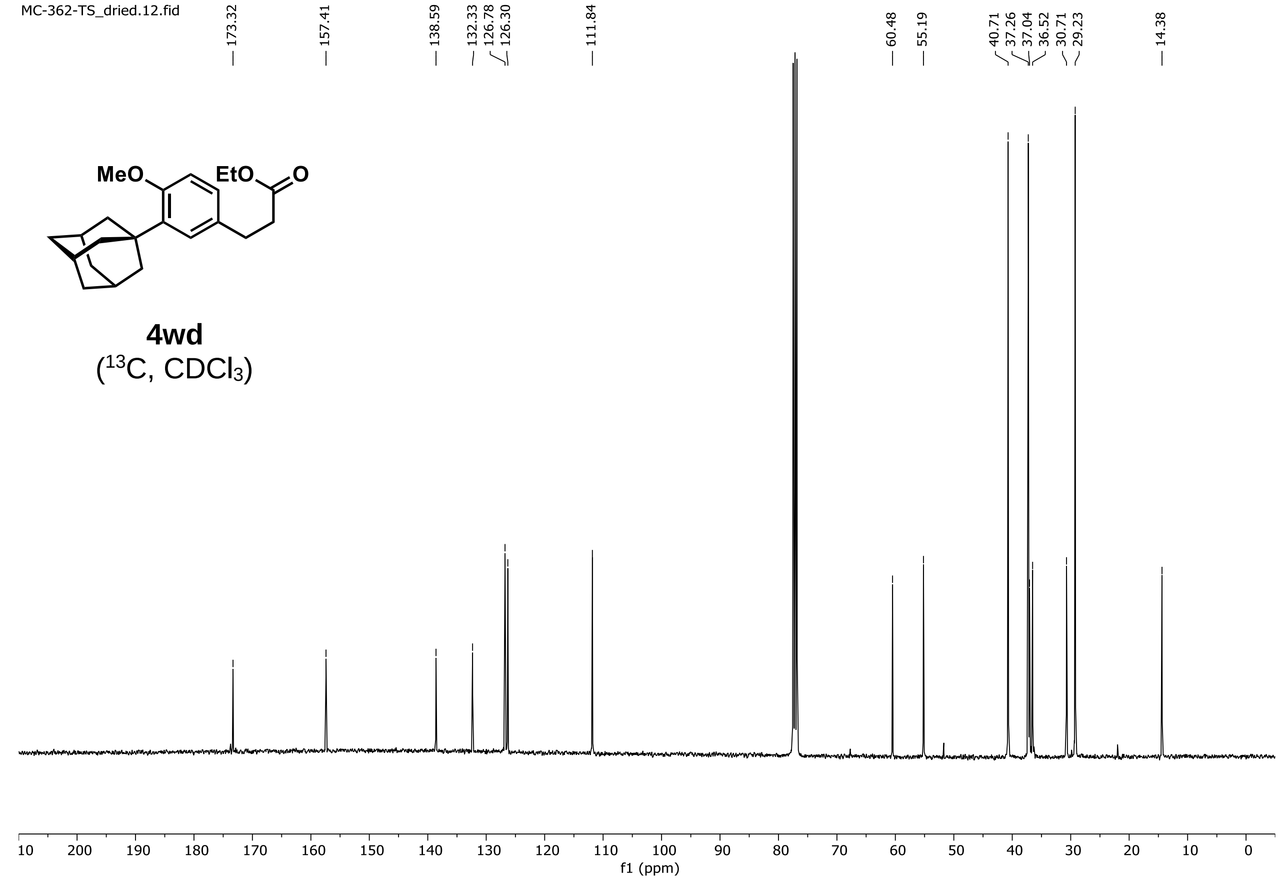
MC-362-TS_dried.12.fid
MeO
EtO
O
4wd
(¹³C, CDCl3)
f1 (ppm)

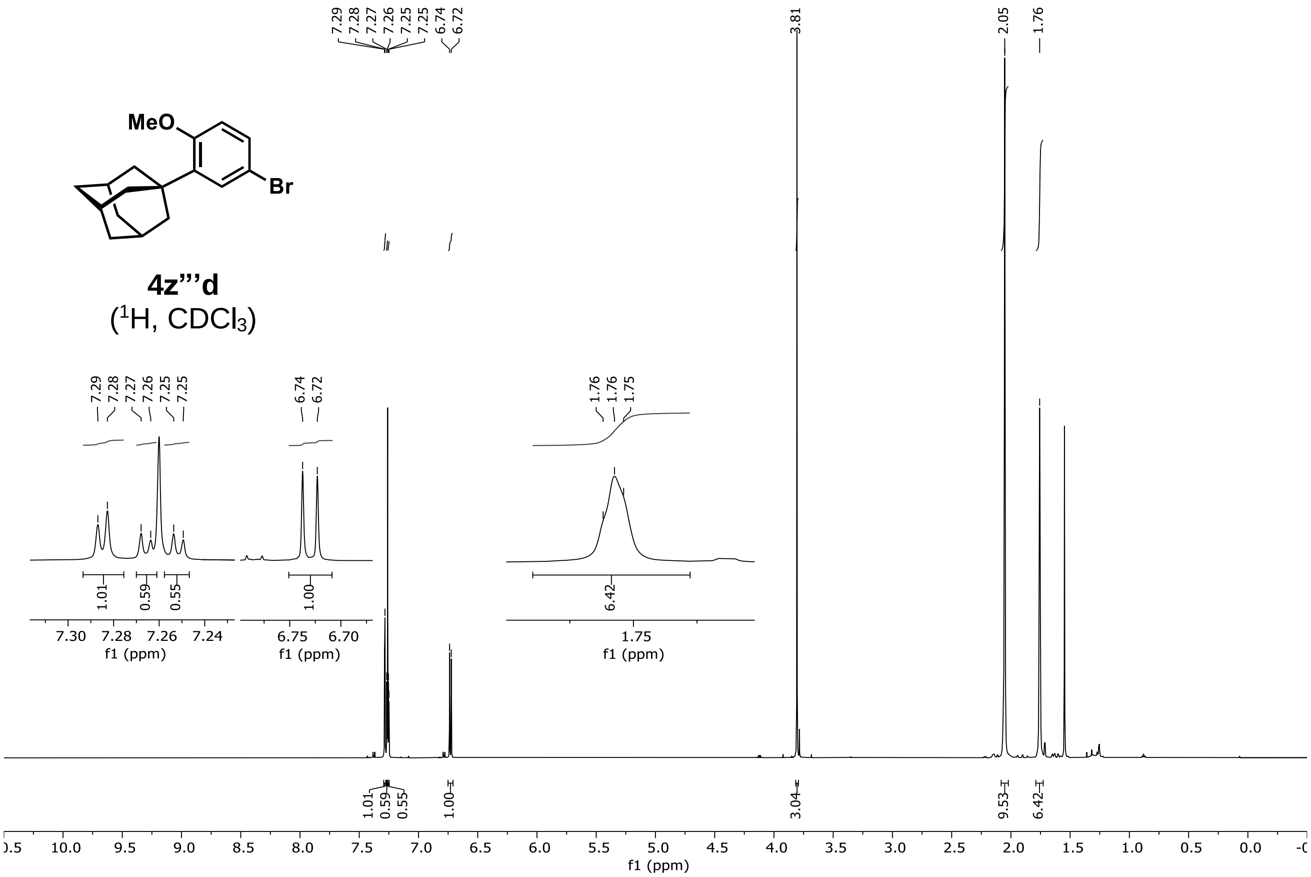

MC-313-TS_C.10.fid

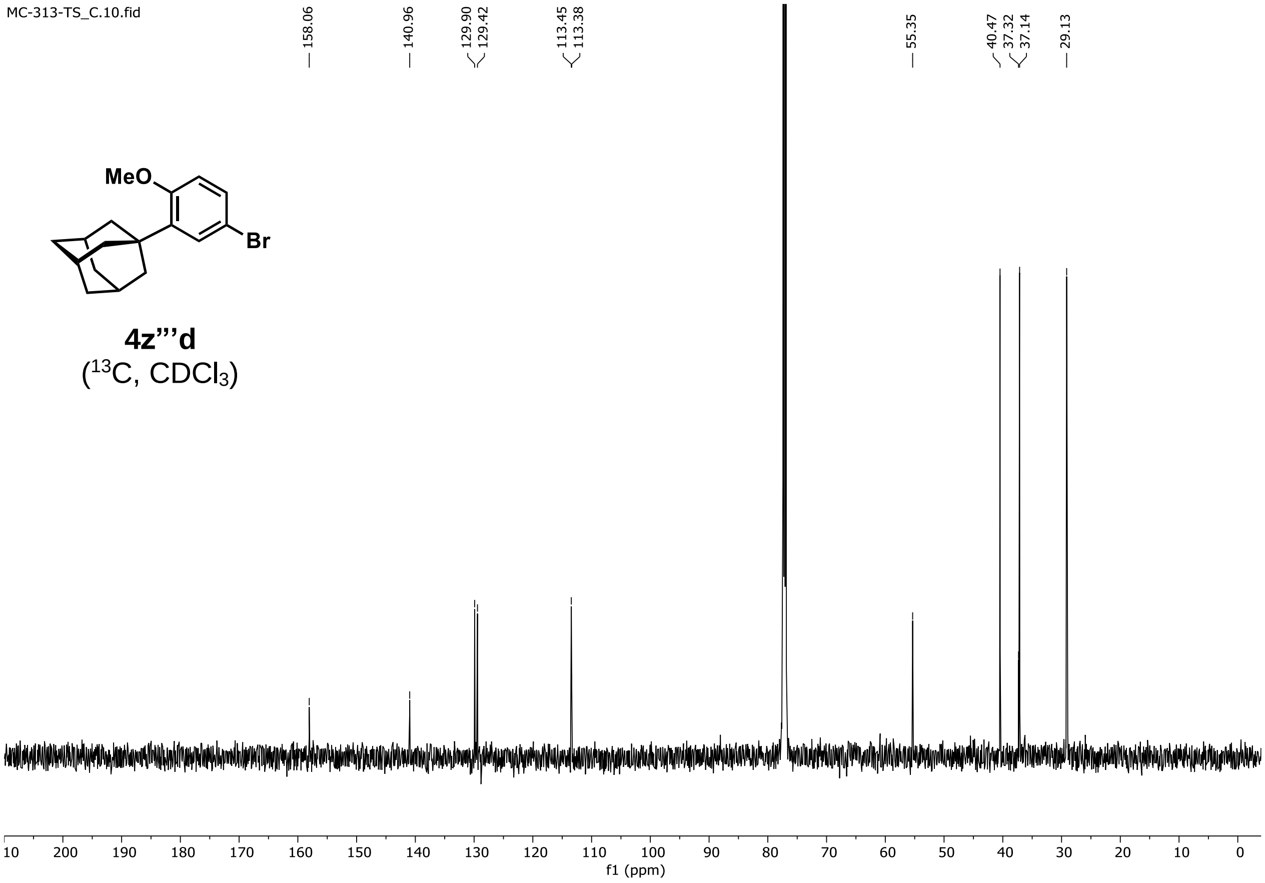

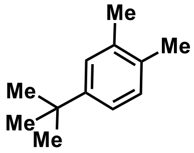

5aa
(^{1}H, $CDCl_3$)

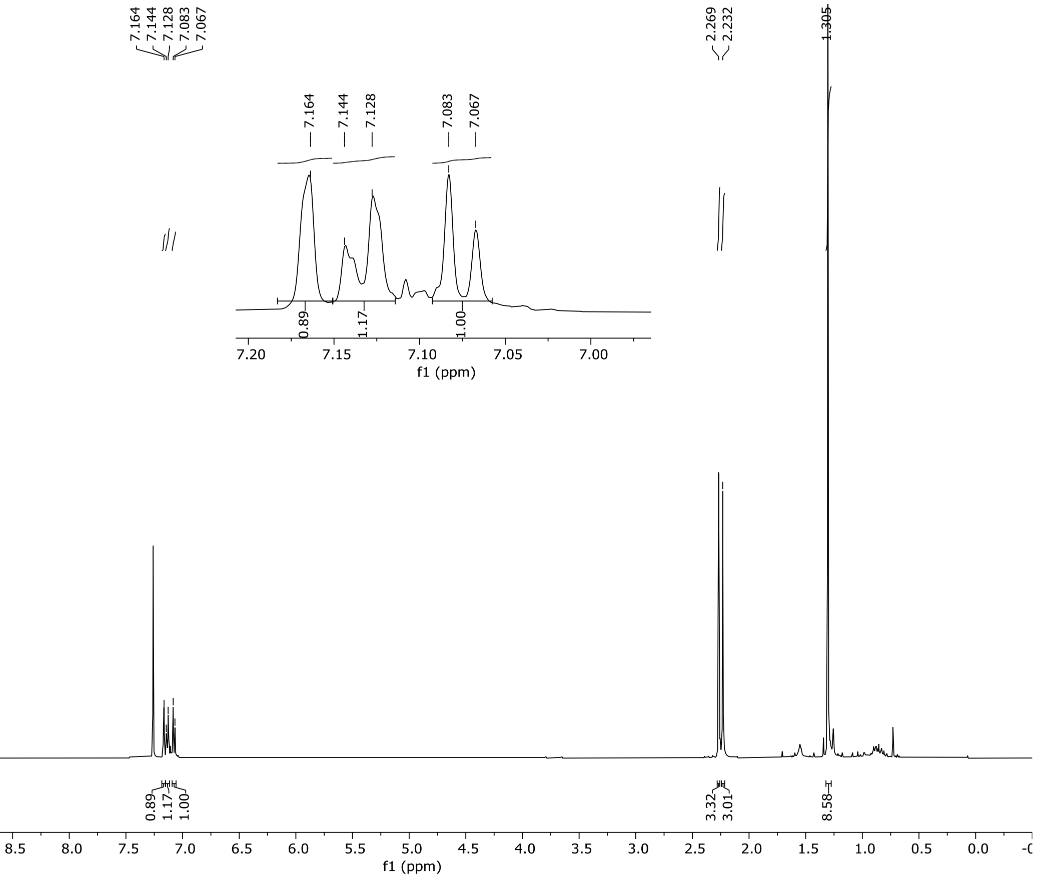

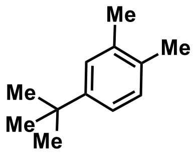

5aa
(^{13}C, $CDCl_3$)

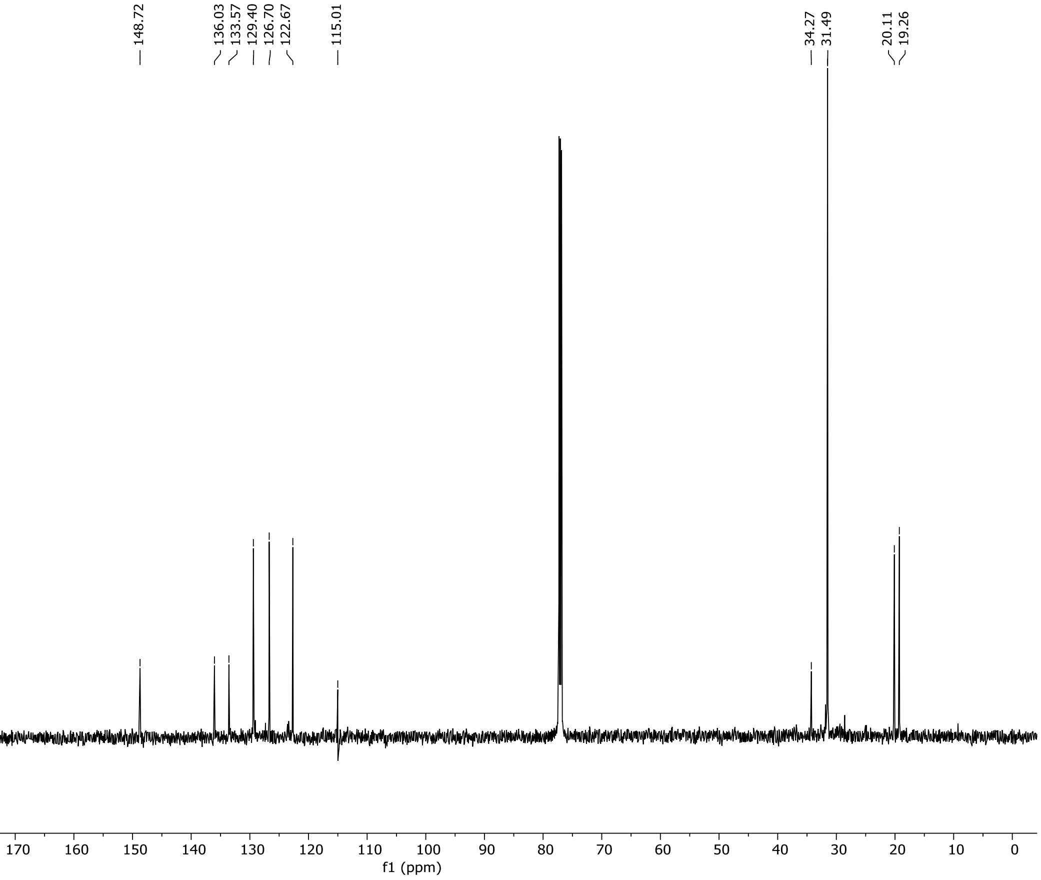

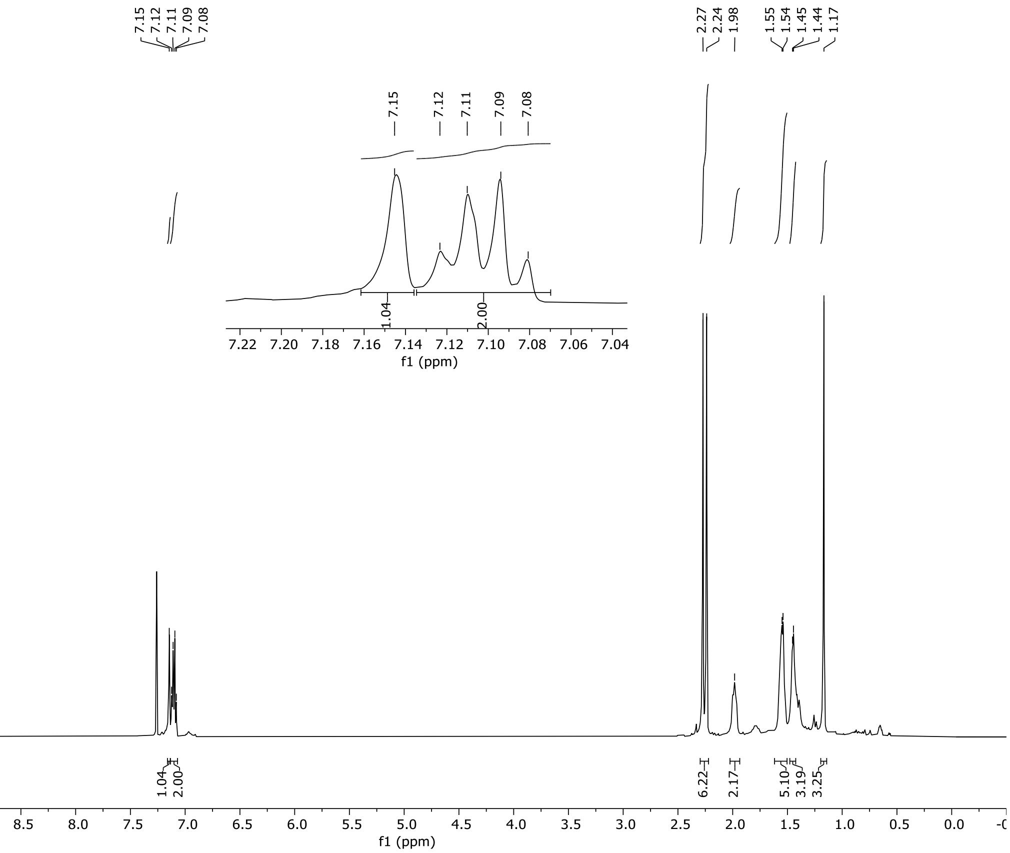

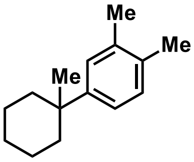

5ac
(^{1}H, $CDCl_3$)

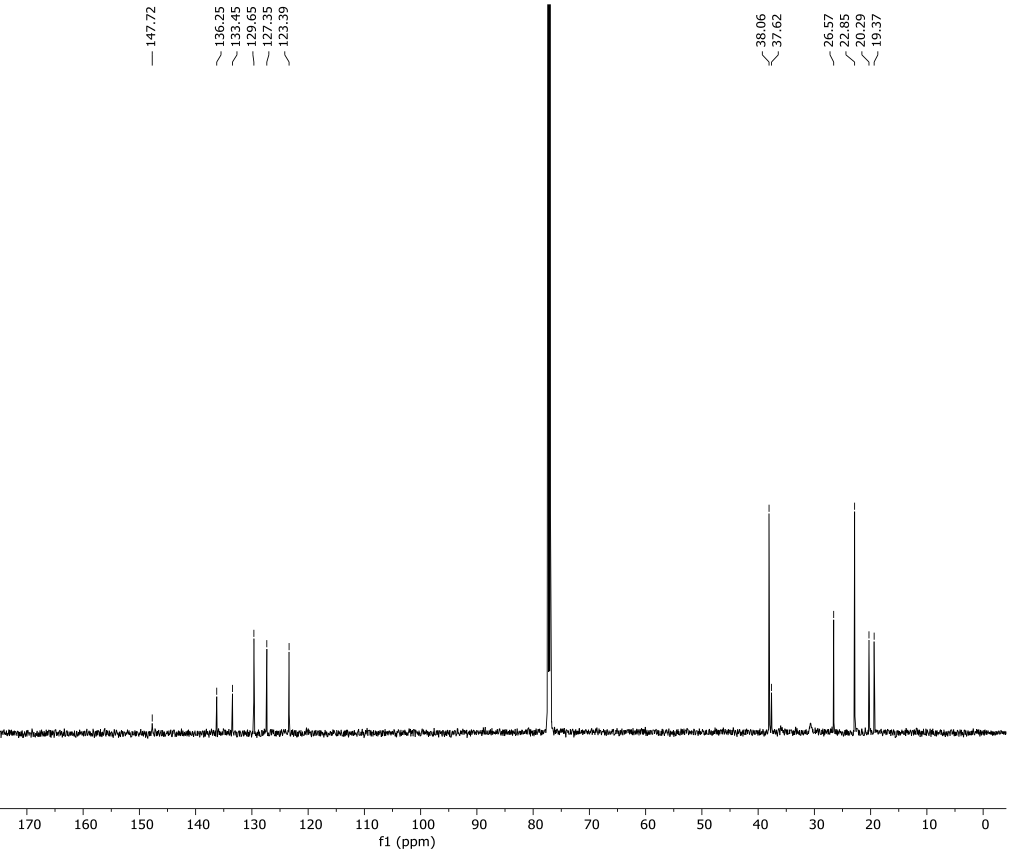

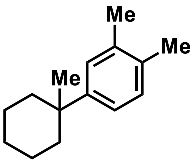

5ac
(^{13}C, $CDCl_3$)

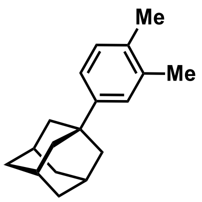

5ad
(^{1}H, $CDCl_3$)

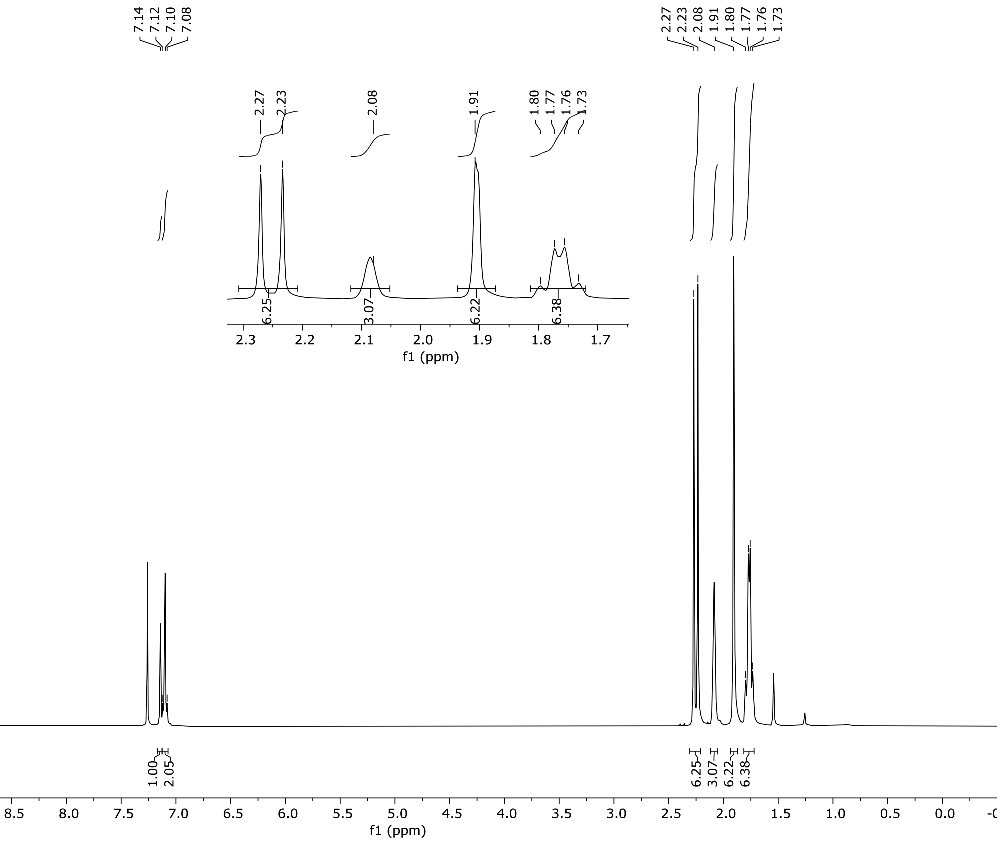

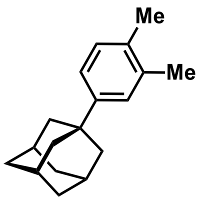

5ad
(^{13}C, $CDCl_3$)

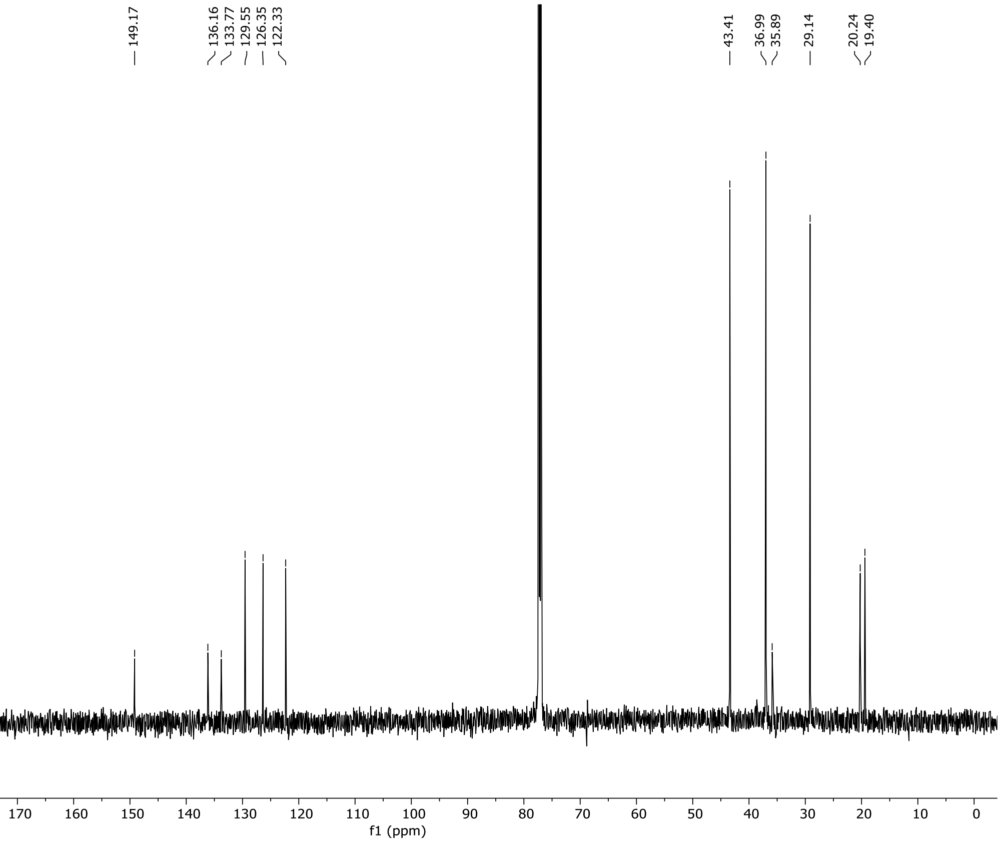

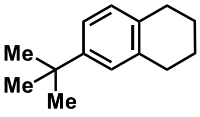

5ba
(^{1}H, $CDCl_3$)

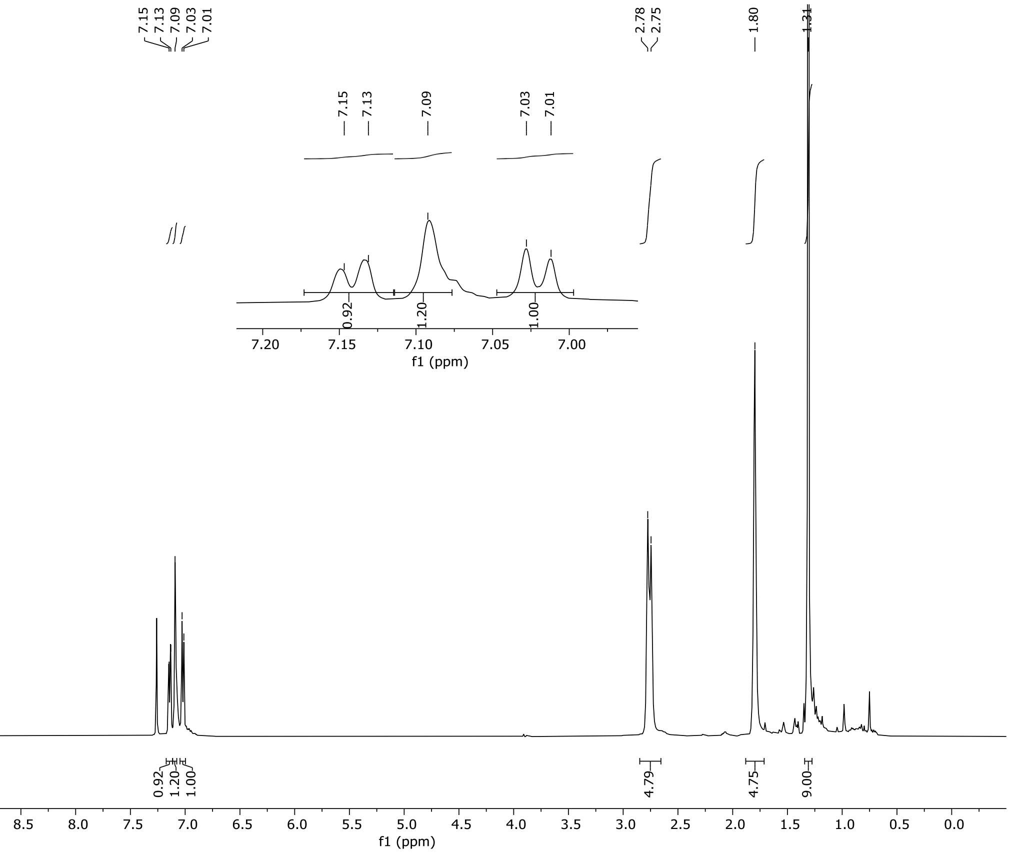

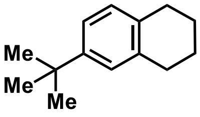

5ba
(^{13}C, $CDCl_3$)

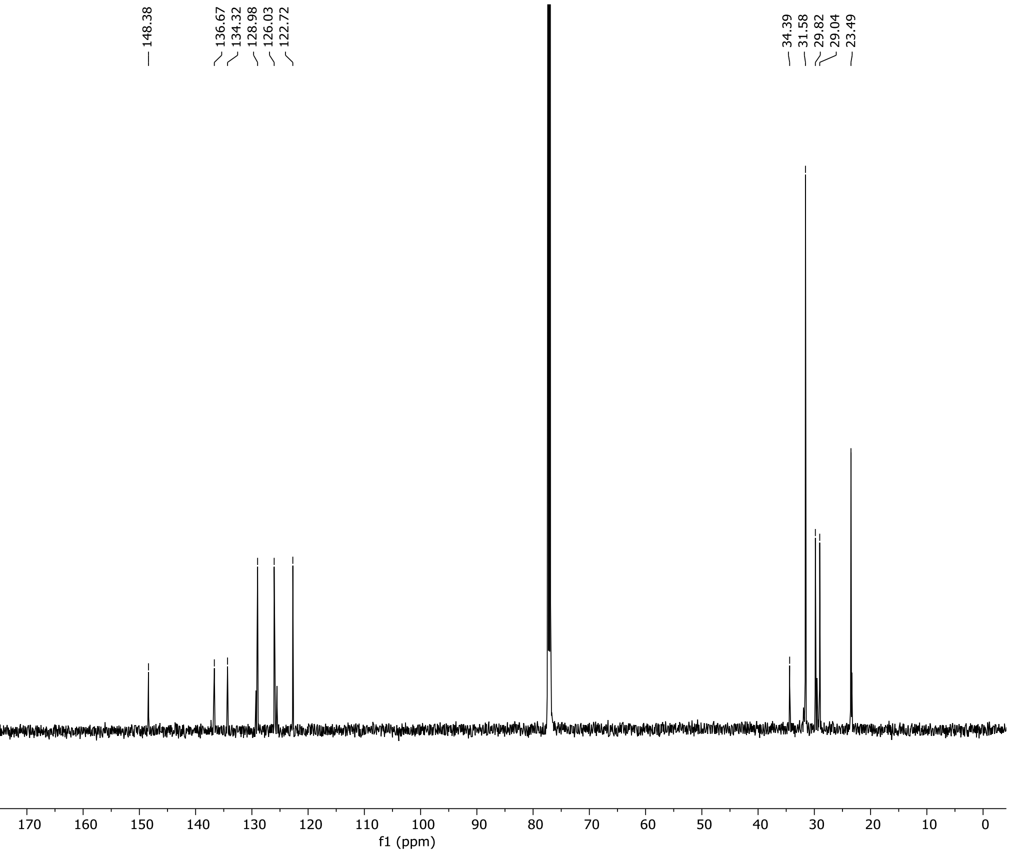

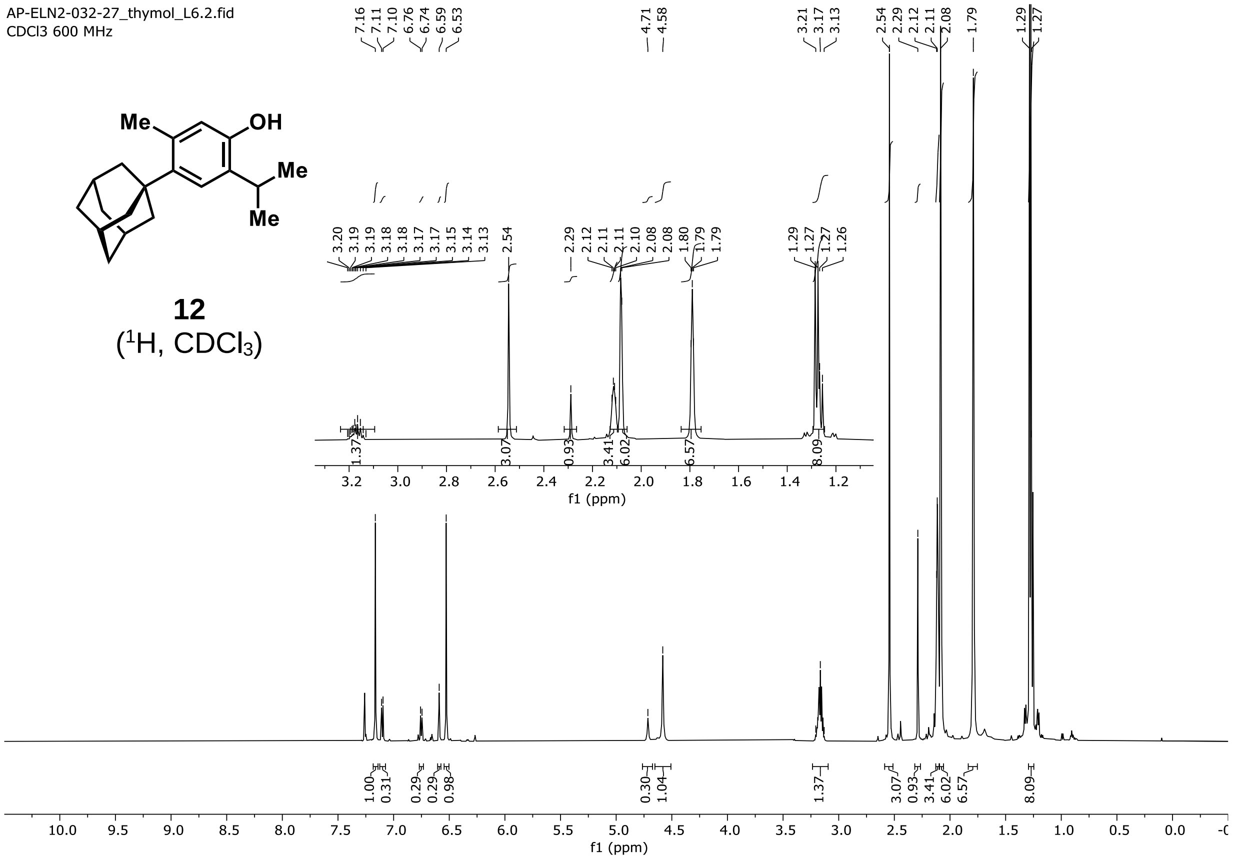

12
(^{1}H, $CDCl_3$)

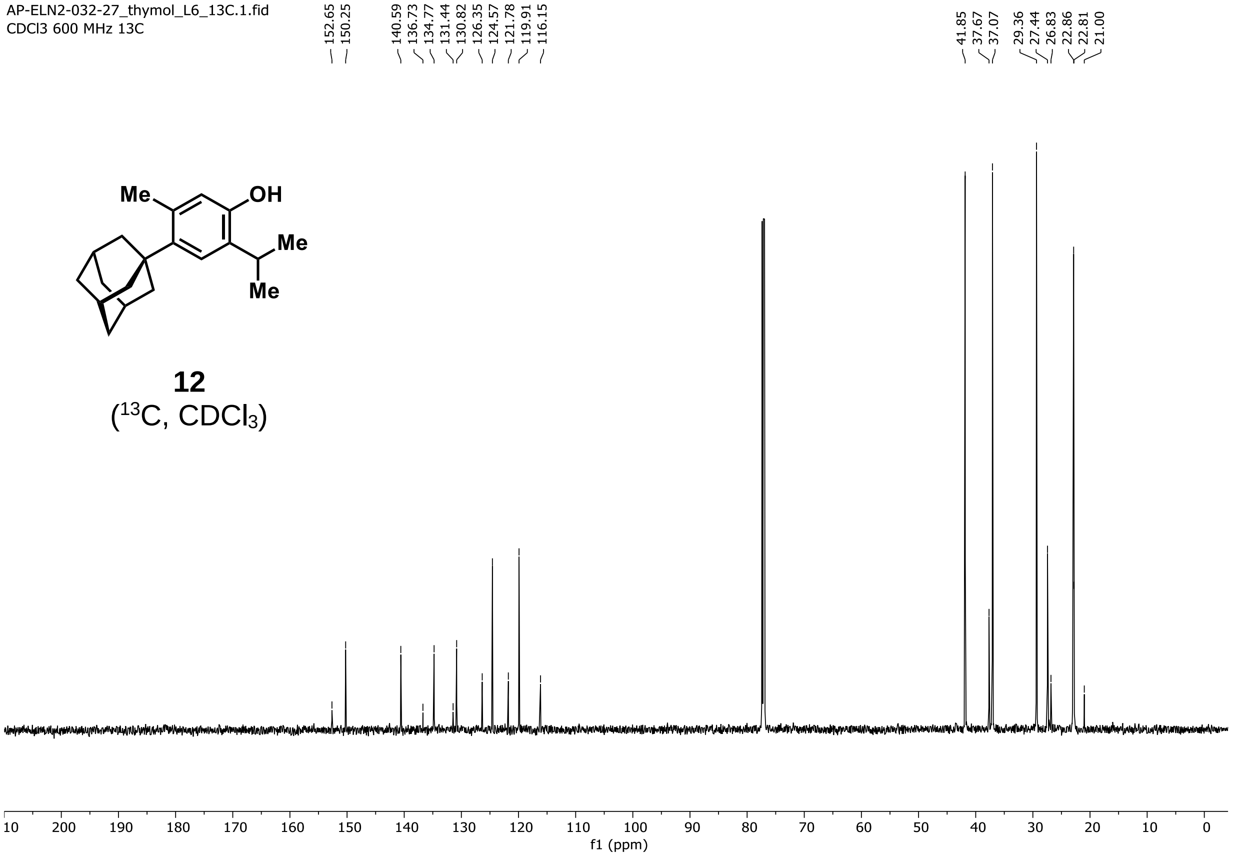

12
(^{13}C, $CDCl_3$)

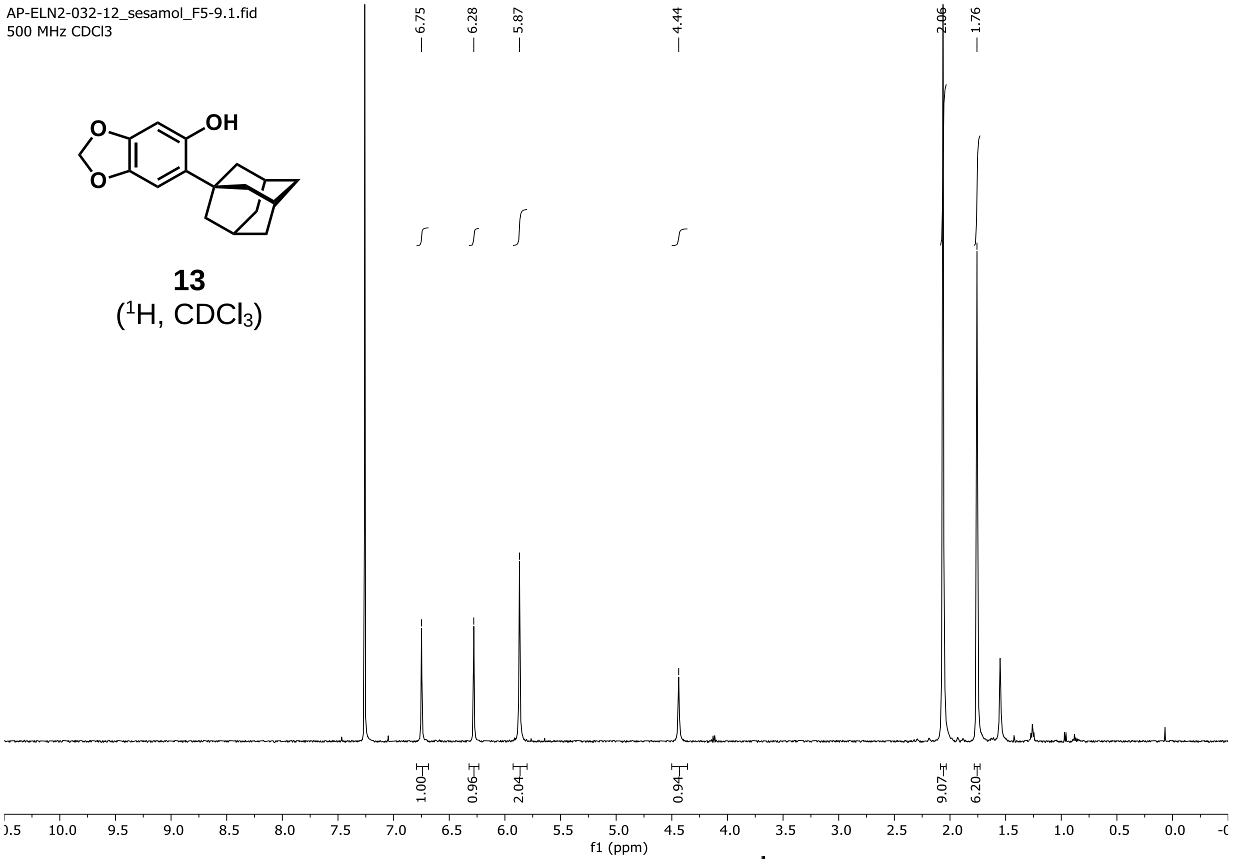
AP-ELN2-032-12_sesamol_F5-9.1.fid
500 MHz CDCl3
OH
O
O
13
(1H, CDCl3)
6.75
6.28
5.87
4.44
2.06
1.76
1.00
0.96
2.04
0.94
9.07
6.20
f1 (ppm)

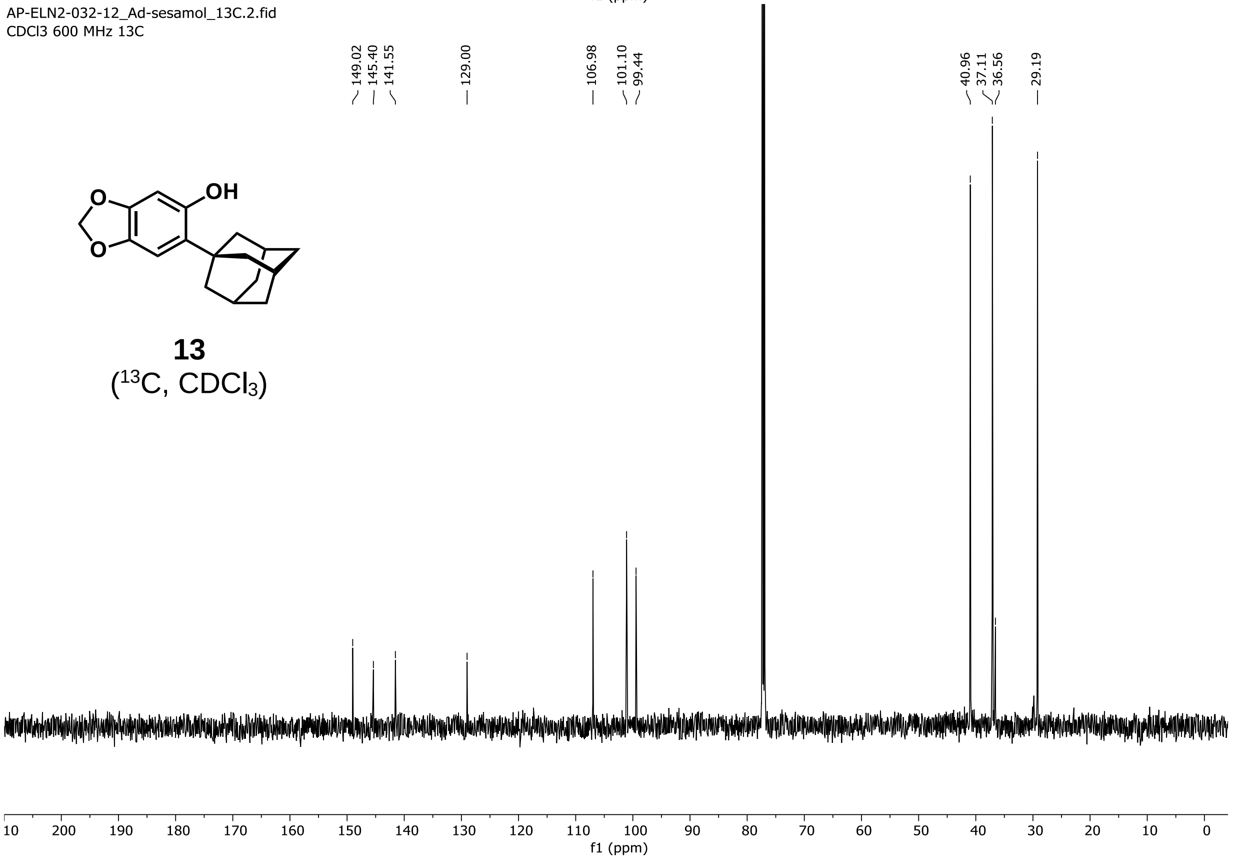
AP-ELN2-032-12_Ad-sesamol_13C.2.fid
CDCl3 600 MHz 13C
OH
O
O
13
(13C, CDCl3)
149.02
145.40
141.55
129.00
106.98
101.10
99.44
40.96
37.11
36.56
29.19
f1 (ppm)

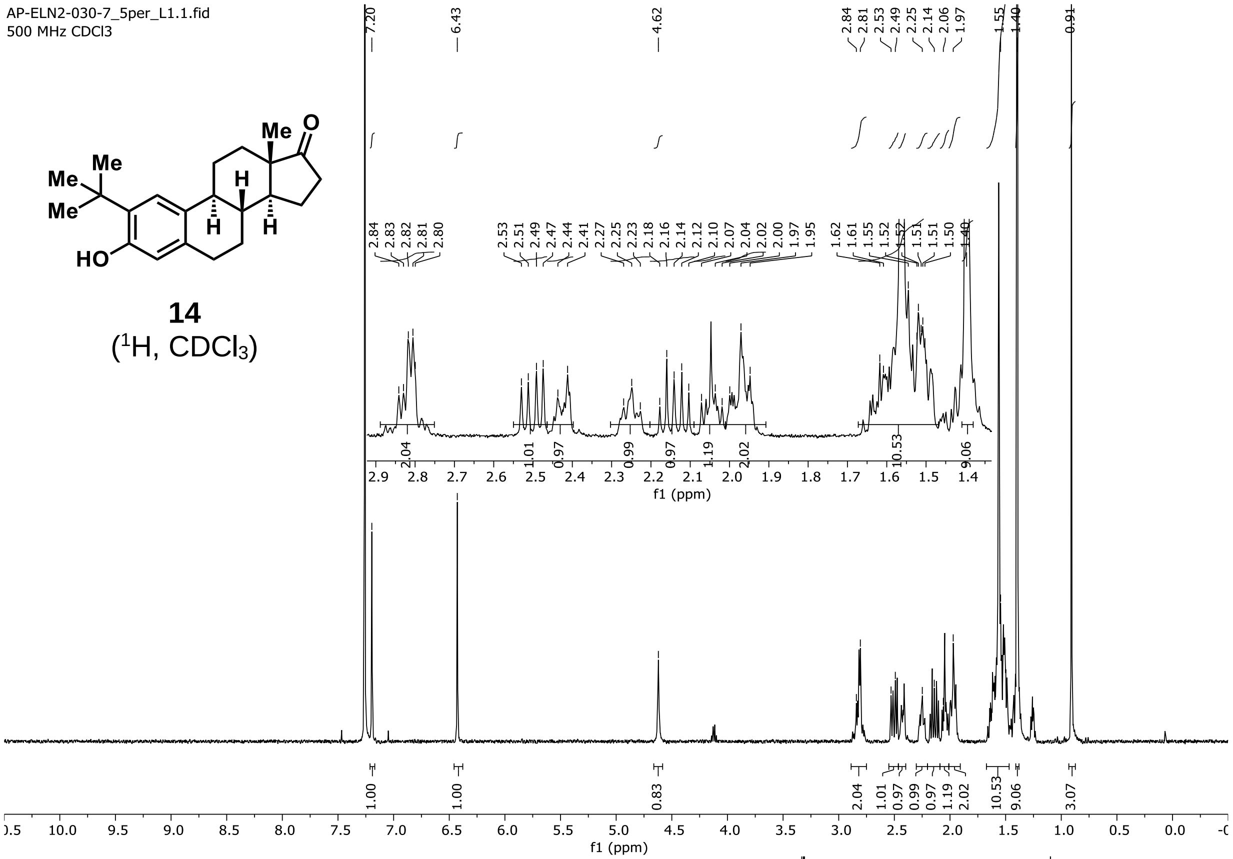

14
(^{1}H, $CDCl_3$)

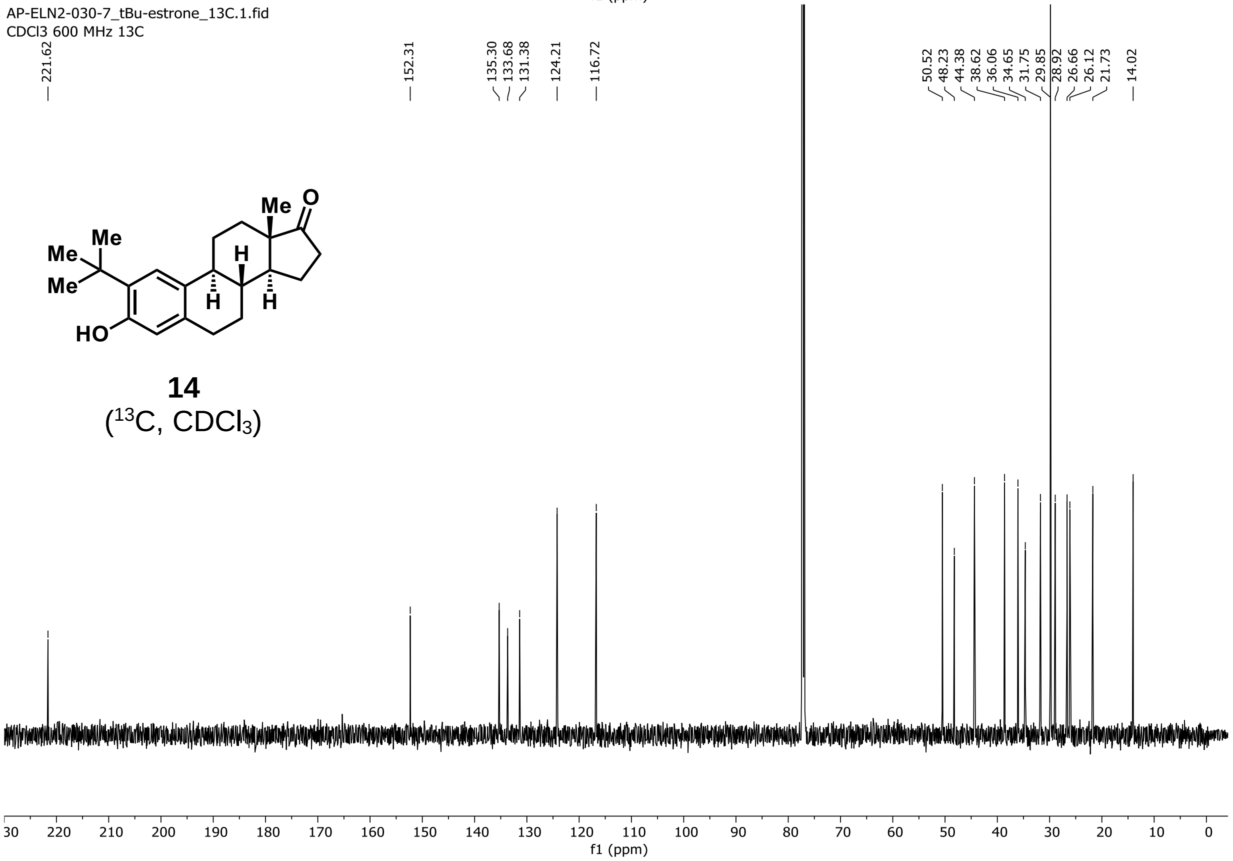

14
(^{13}C, $CDCl_3$)

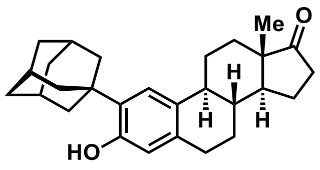

15
(^{1}H, $CDCl_3$)

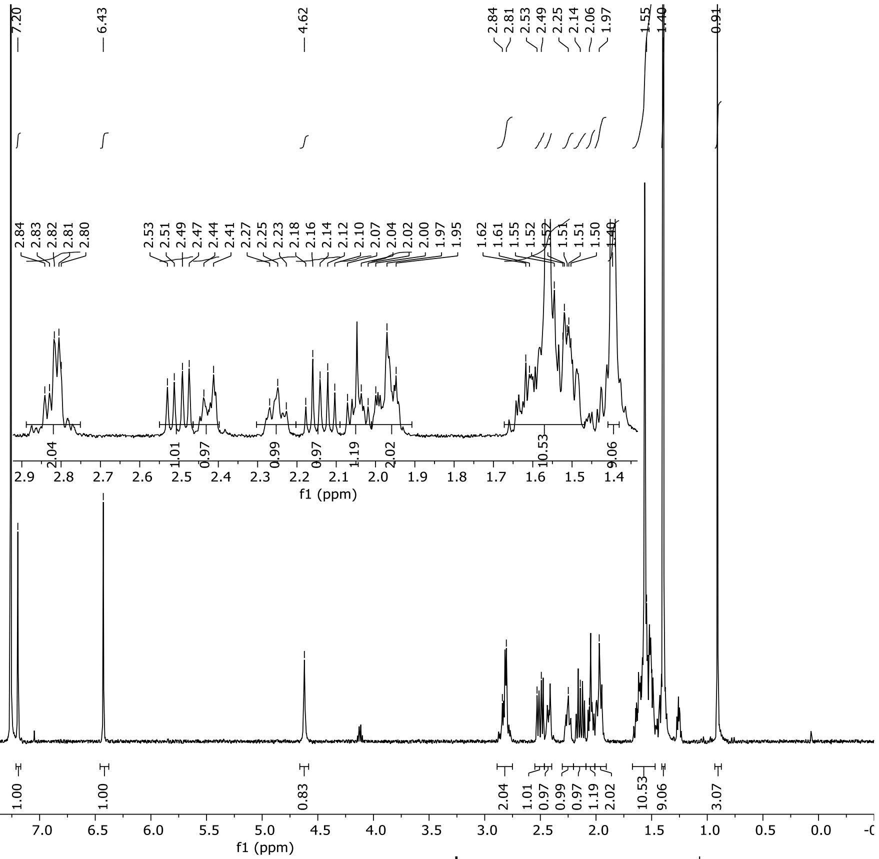

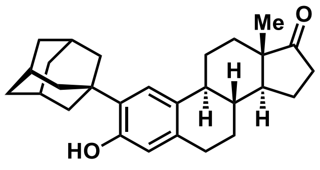

15
(^{13}C, $CDCl_3$)

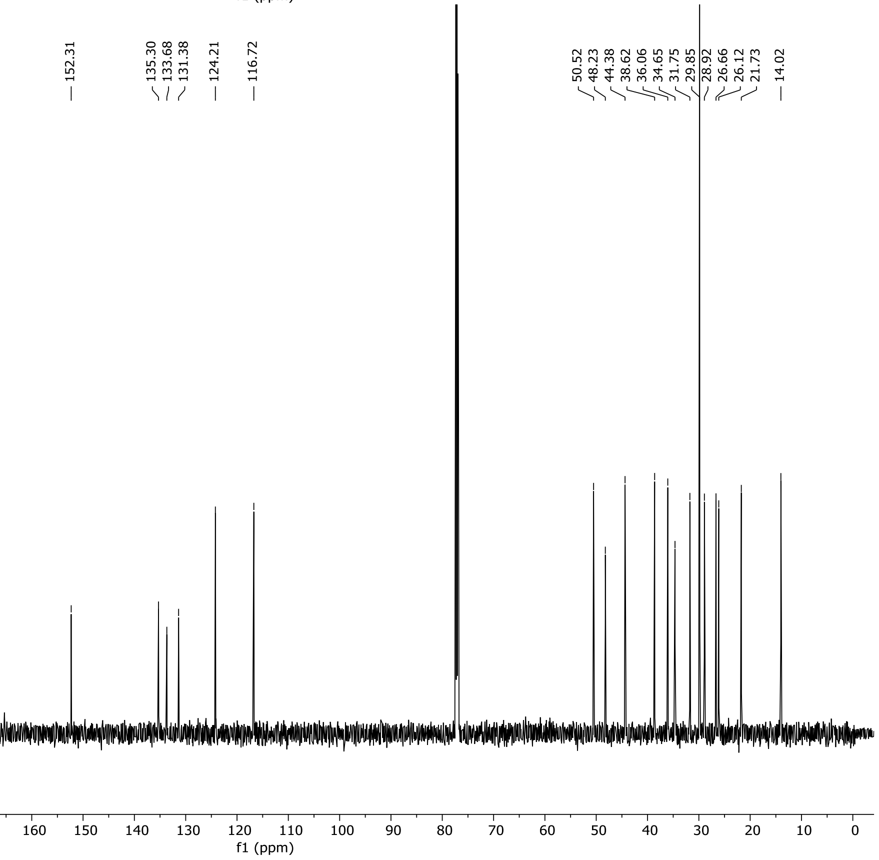

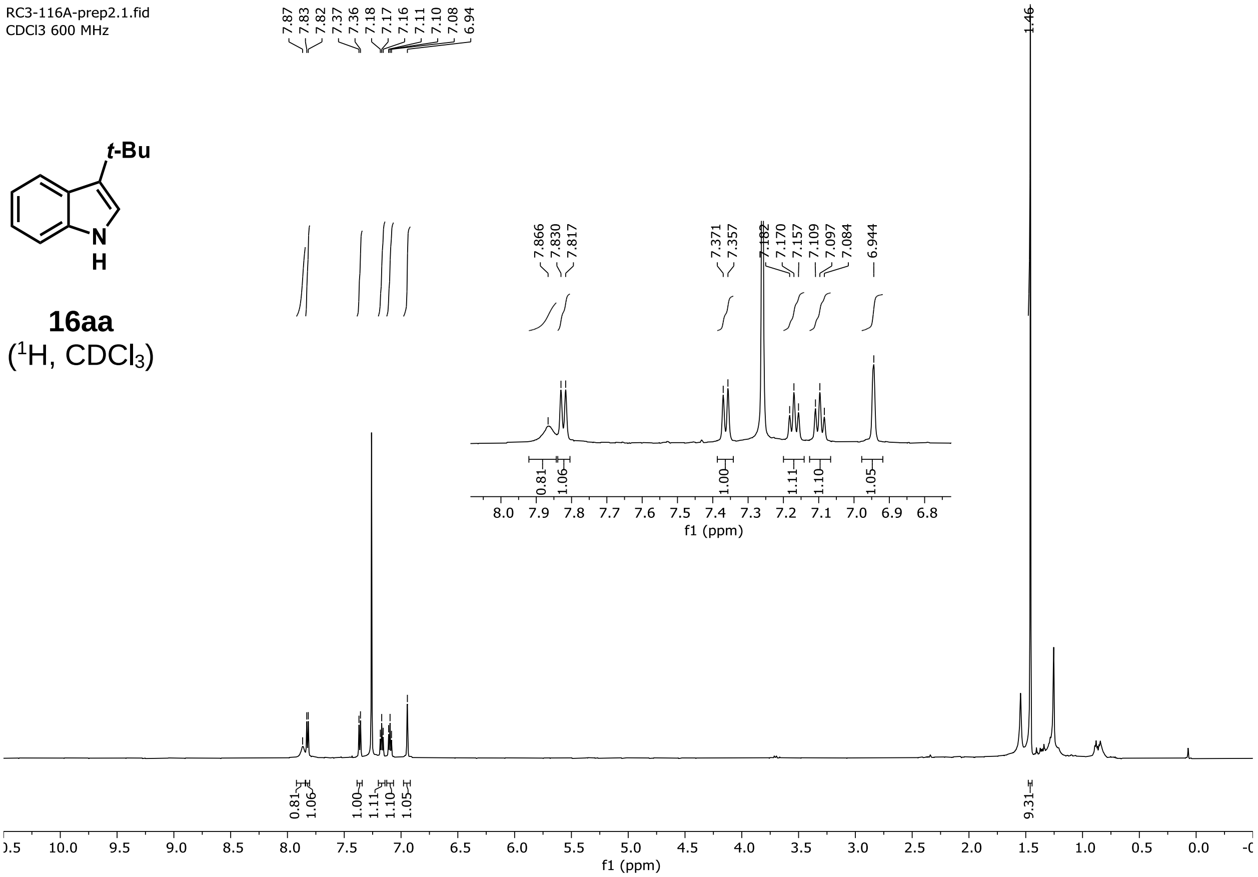
RC3-116A-prep2.1.fid
CDCl3 600 MHz
t-Bu
N
H
16aa
(1H, CDCl3)
f1 (ppm)

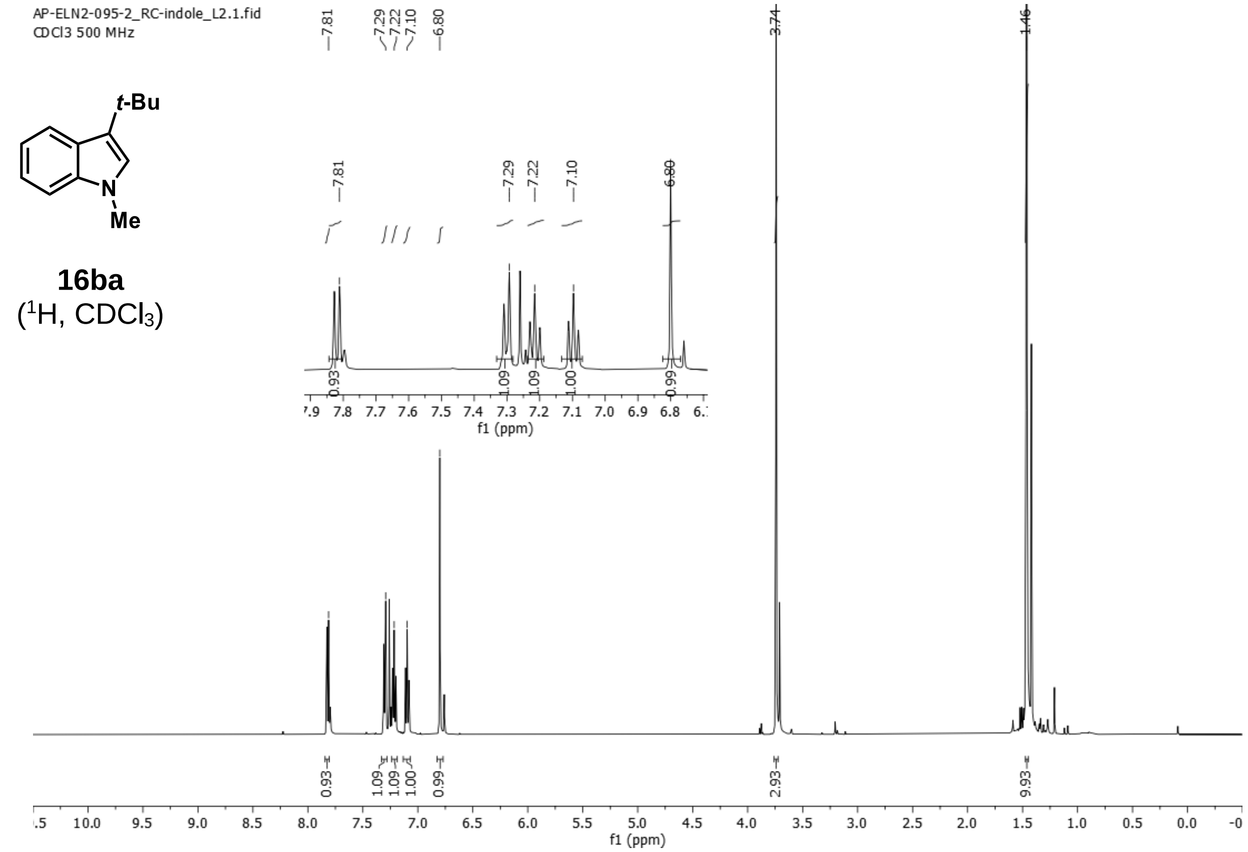

16ba
(^{1}H, $CDCl_3$)

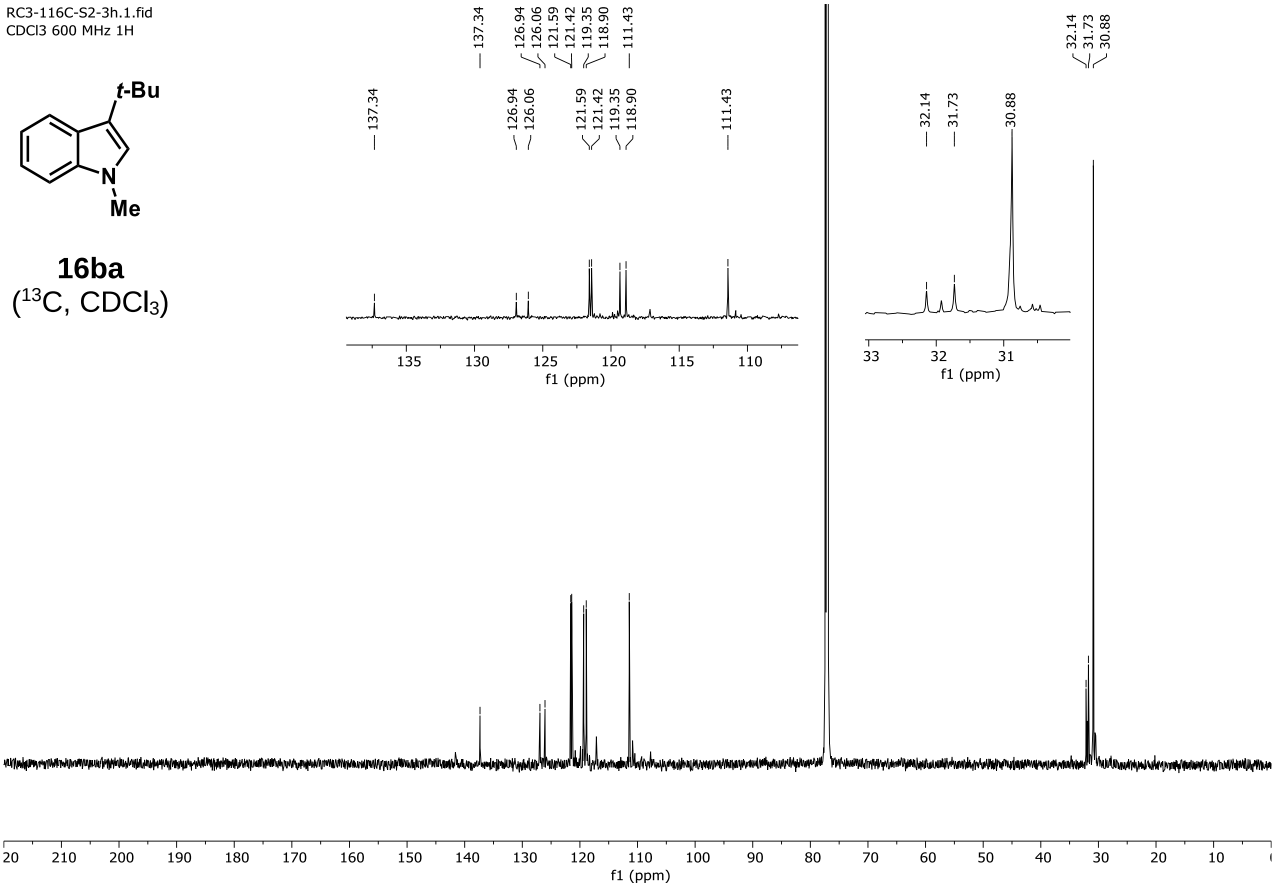

16ba
(^{13}C, $CDCl_3$)

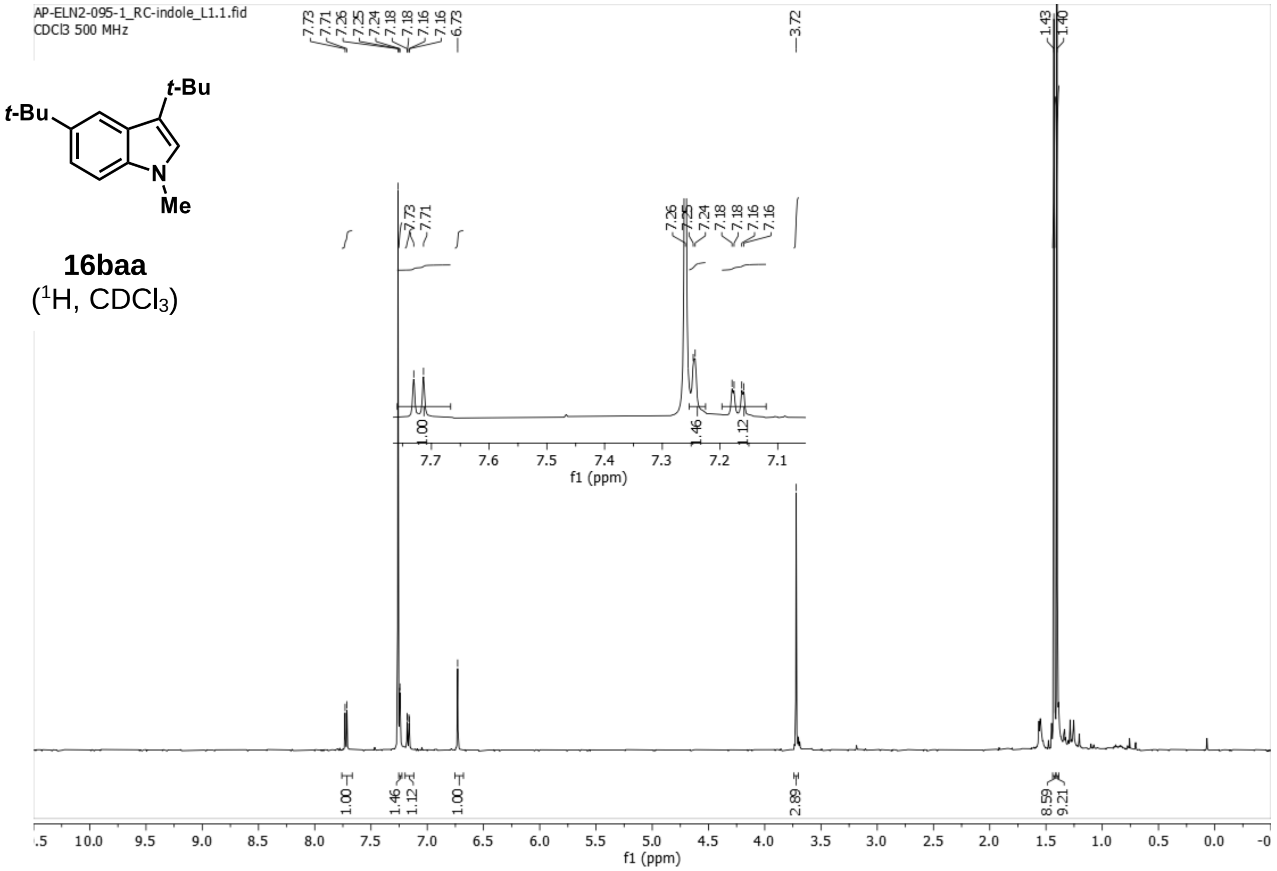

16baa
(^{1}H, $CDCl_3$)

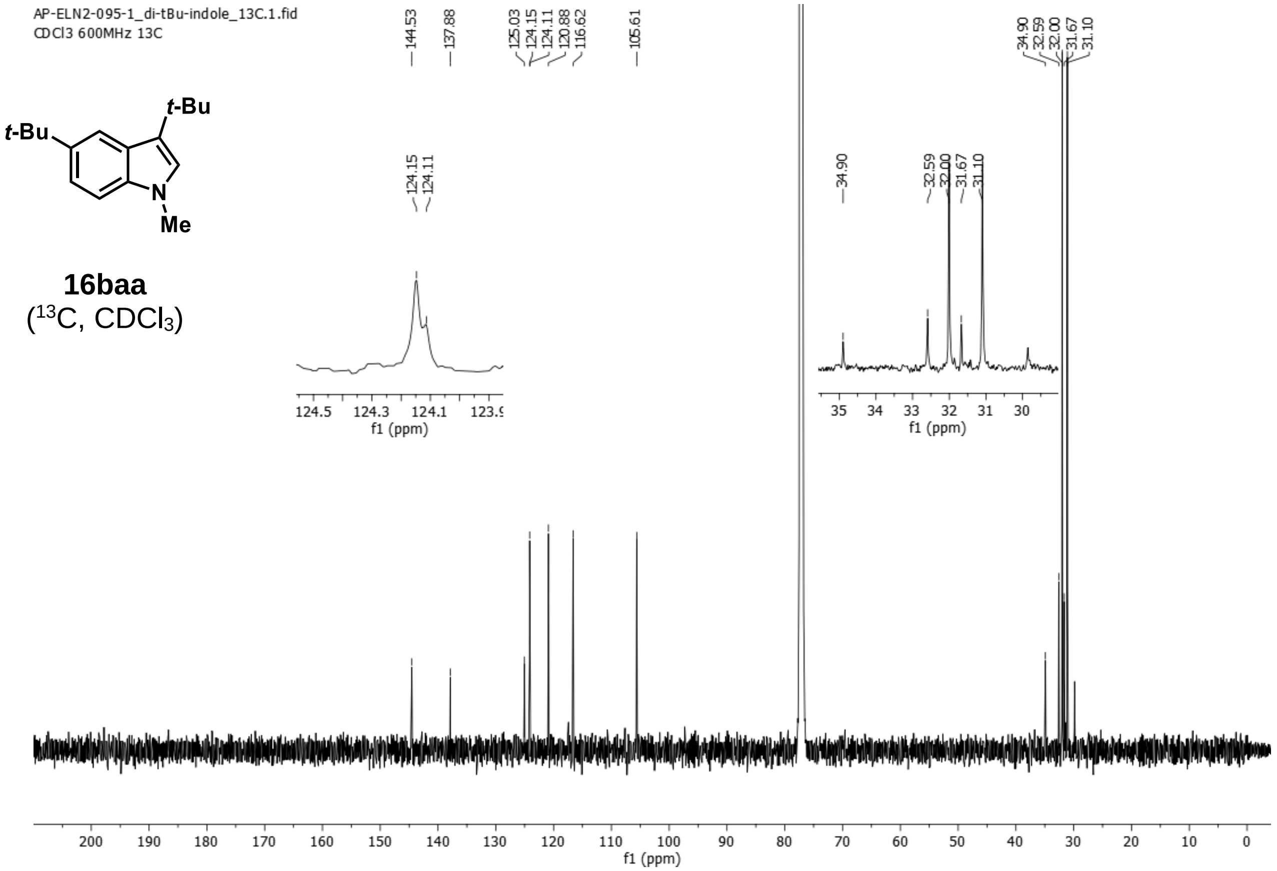

16baa
(^{13}C, $CDCl_3$)

RC1-67-column.1.fid
400 MHz

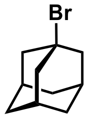

17
(^{1}H, $CDCl_3$)

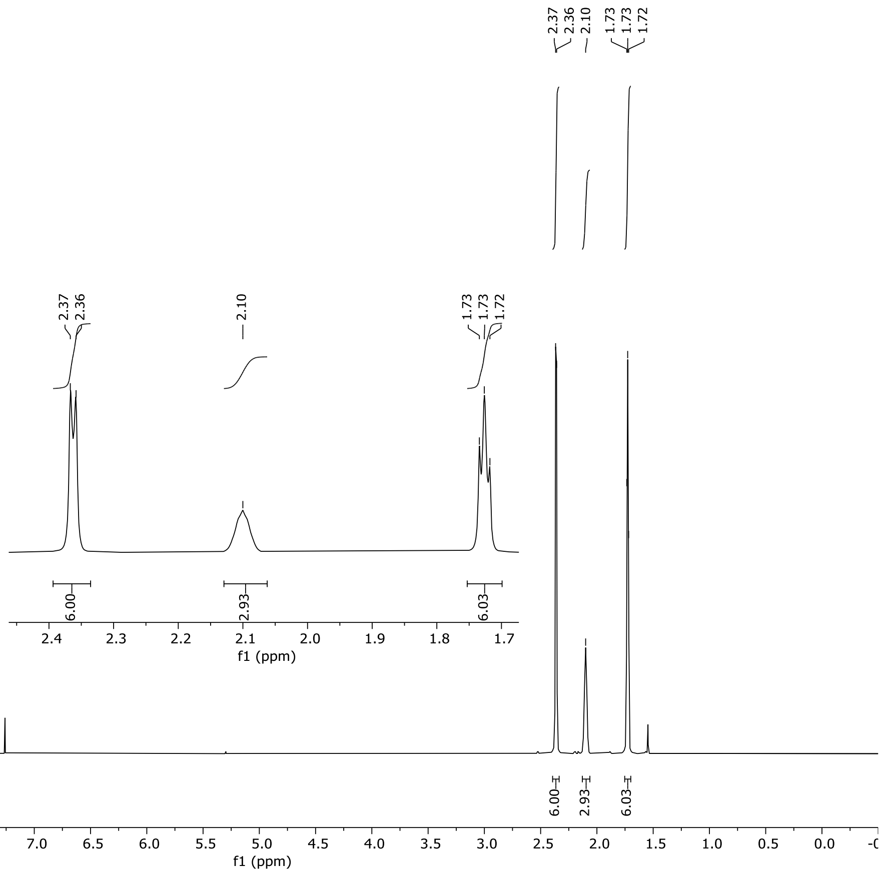

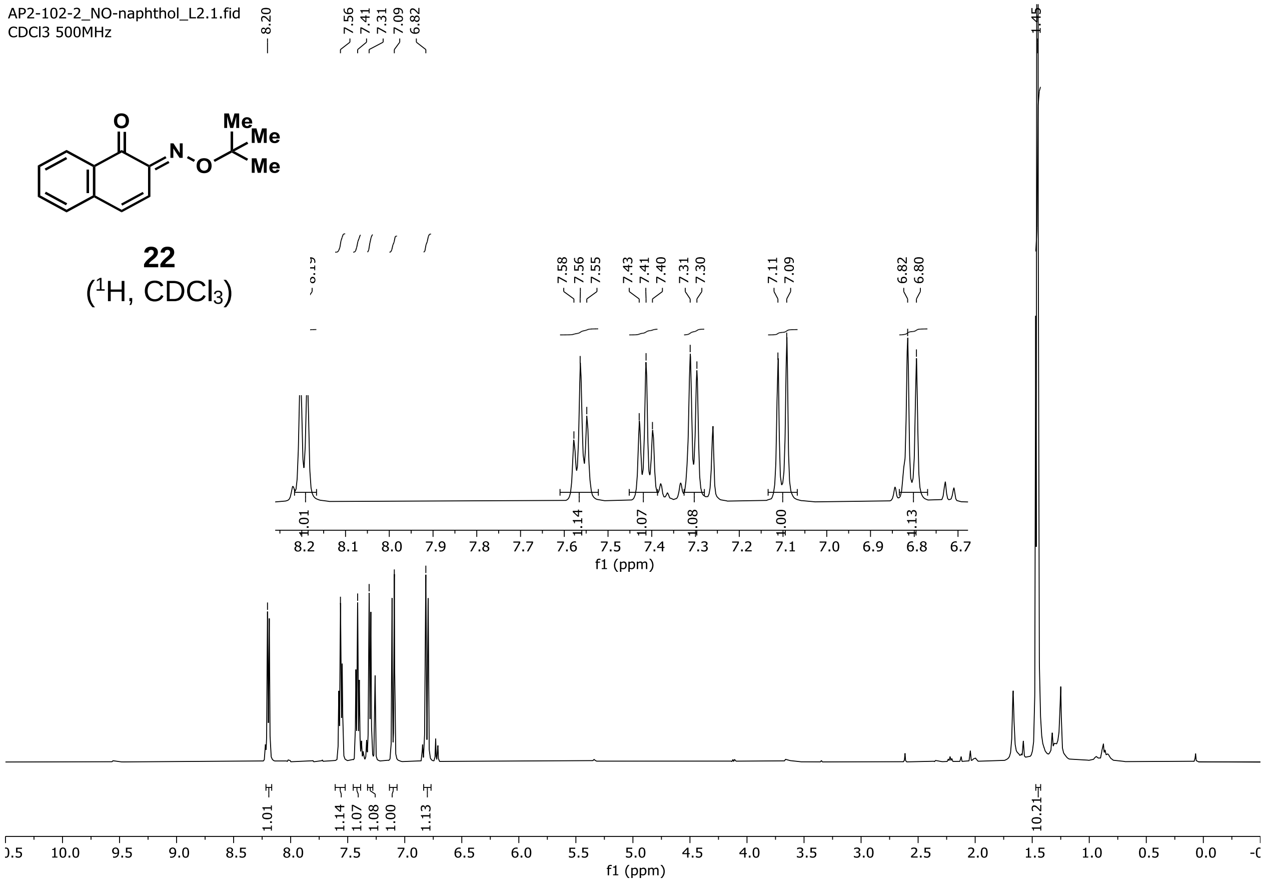
AP2-102-2_NO-naphthol_L2.1.fid
CDCl3 500MHz
8.20
7.56
7.41
7.31
7.09
6.82
1.45
22
(^{1}H, $CDCl_3$)
7.58
7.56
7.55
7.43
7.41
7.40
7.31
7.30
7.11
7.09
6.82
6.80
1.01
1.14
1.07
1.08
1.00
1.13
10.21
f1 (ppm)

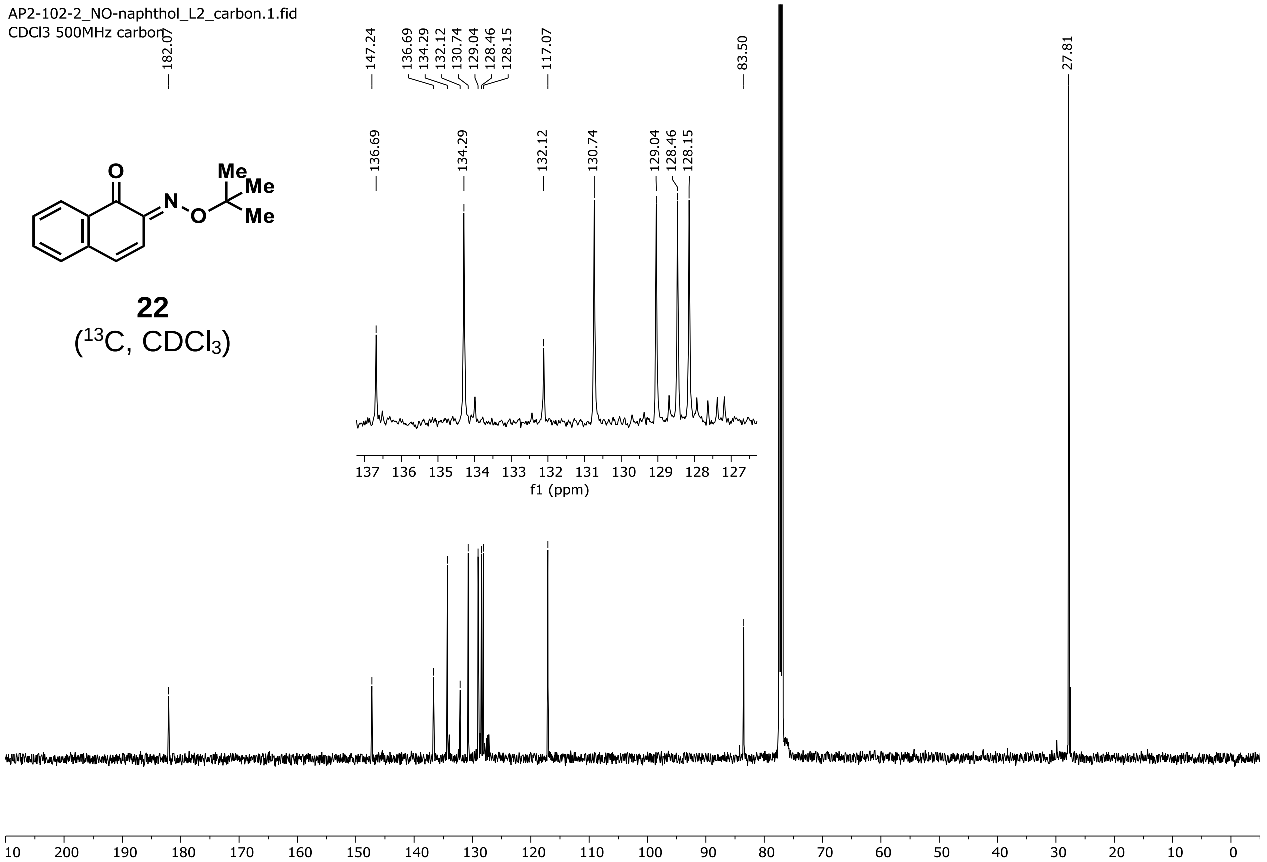
AP2-102-2_NO-naphthol_L2_carbon.1.fid
CDCl3 500MHz carbon
182.07
147.24
136.69
134.29
132.12
130.74
129.04
128.46
128.15
117.07
83.50
27.81
22
(^{13}C, $CDCl_3$)
f1 (ppm)
